# Global analysis of biosynthetic gene clusters reveals conserved and unique natural products in entomopathogenic nematode-symbiotic bacteria

**DOI:** 10.1038/s41557-022-00923-2

**Published:** 2022-04-25

**Authors:** Yi-Ming Shi, Merle Hirschmann, Yan-Ni Shi, Shabbir Ahmed, Desalegne Abebew, Nicholas J. Tobias, Peter Grün, Jan J. Crames, Laura Pöschel, Wolfgang Kuttenlochner, Christian Richter, Jennifer Herrmann, Rolf Müller, Aunchalee Thanwisai, Sacha J. Pidot, Timothy P. Stinear, Michael Groll, Yonggyun Kim, Helge B. Bode

**Affiliations:** 1 Department of Natural Products in Organismic Interactions, Max Planck Institute for Terrestrial Microbiology, Marburg, Germany; 2 Molecular Biotechnology, Department of Biosciences, Goethe University Frankfurt, Frankfurt am Main, Germany; 3 Department of Plant Medicals, College of Life Sciences, Andong National University, Andong, Korea; 4 LOEWE Center for Translational Biodiversity Genomics (TBG), Frankfurt am Main, Germany; 5 Senckenberg Gesellschaft für Naturforschung, Frankfurt am Main, Germany; 6 Center for Protein Assemblies, Department of Chemistry, Technical University of Munich, Garching, Germany; 7 Institute for Organic Chemistry and Chemical Biology, Center for Biomolecular Magnetic Resonance, Goethe University Frankfurt, Frankfurt am Main, Germany; 8 Helmholtz Institute for Pharmaceutical Research Saarland (HIPS), Helmholtz Center for Infection Research & German Center for Infection Research (DZIF), partner site Hannover-Braunschweig, Saarbrücken, Germany; 9 Department of Microbiology and Parasitology, Faculty of Medical Science, Naresuan University, Phitsanulok, Thailand; 10 Department of Microbiology and Immunology, Peter Doherty Institute for Infection and Immunity, University of Melbourne, Melbourne, Victoria Australia

**Keywords:** Symbiosis, Chemical ecology, Microbial ecology, Natural products

## Abstract

Microorganisms contribute to the biology and physiology of eukaryotic hosts and affect other organisms through natural products. *Xenorhabdus* and *Photorhabdus* (*XP*) living in mutualistic symbiosis with entomopathogenic nematodes generate natural products to mediate bacteria–nematode–insect interactions. However, a lack of systematic analysis of the *XP* biosynthetic gene clusters (BGCs) has limited the understanding of how natural products affect interactions between the organisms. Here we combine pangenome and sequence similarity networks to analyse BGCs from 45 *XP* strains that cover all sequenced strains in our collection and represent almost all *XP* taxonomy. The identified 1,000 BGCs belong to 176 families. The most conserved families are denoted by 11 BGC classes. We homologously (over)express the ubiquitous and unique BGCs and identify compounds featuring unusual architectures. The bioactivity evaluation demonstrates that the prevalent compounds are eukaryotic proteasome inhibitors, virulence factors against insects, metallophores and insect immunosuppressants. These findings explain the functional basis of bacterial natural products in this tripartite relationship.

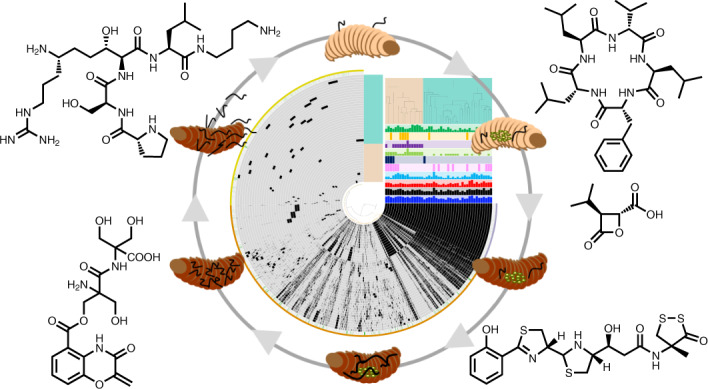

## Abstract (web summary)

Entomopathogenic nematodes carrying *Xenorhabdus* and *Photorhabdus* bacteria prey on insect larvae in the soil. Now, a comprehensive analysis of the bacterial genome has revealed ubiquitous and unique families of biosynthetic gene clusters. Evaluation of the bioactivity of the natural products expressed by the most prevalent cluster families explains the functional basis of bacterial natural products involved in bacteria–nematode–insect interactions.

## Main

Interactions between microorganisms (for example, bacteria) and higher eukaryotes are ubiquitous and have essential medical, environmental and evolutionary significance^1^. Microorganisms supply nutrients^2^, shape immune systems^3^, maintain diverse and productive communities^4^ and drive evolution^5^ for higher eukaryotic hosts. Such microbe–host interactions can be relationships ranging from mutualistic/parasitic to pathogenic symbiosis^6^, in which microorganisms sense and respond to environmental changes with diffusible small molecules. These small molecules are also known as natural products or specialized metabolites, which affect not only the microbial host but also neighbouring microbes and other organisms^7^. However, due to limitations in the genetic tractability of microbial species, as well as formidable obstacles to imitating microbial natural habitats^8^, only a few correlations between microbial natural products (for example, colibactin^9,10^ and tilivalline^11^ produced by the human gut microbiota) and the function with which the microbial natural products endow the producers have been established.

Entomopathogenic *Xenorhabdus* and *Photorhabdus* (*XP*) bacteria live in mutualistic symbiosis with nematodes of the genera *Steinernema* and *Heterorhabditis*, respectively. The dauer-stage nematodes carrying the symbiotic bacteria within their intestines actively search for insect larvae in the soil^12,13^, additionally sensing signals from plant roots infected by insects^14^. When nematodes invade insect prey through natural openings and cuticles, the bacterial symbionts are released into the insect haemolymph, where the bacteria begin to propagate and produce proteins (for example, toxins and lytic enzymes) and natural products that help with killing the insect prey, degrading the insect cadaver, and protecting it against other soil-living organisms. The nematodes then feed on the predigested insect tissues, as well as *XP*, and reproduce within the cadaver. Upon food depletion, a new generation of dauer-stage nematodes re-associates with the symbiotic bacteria, exits the carcass and seeks new prey. Notably, although *XP* strains have yet to be found independently of environmental sources, they can be cultivated and genetically manipulated under standard laboratory conditions^12^. Also, the other two organisms, nematodes and insects, can be established readily in laboratory environments. Therefore, the contribution of individual bacterial factors to the mutualism, as well as to the predator–prey relation with the participation of one or multiple players, can be easily delineated. These aspects render the system a promising model to address the ecological functions of microbial natural products.

*XP* natural products involved in bacterial cell–cell communication, nematode development, insect pathogenicity, insect immune suppression and the inhibition of other competitive microorganisms are instrumental in maintaining the complex life cycle^7^. Our previous metabolic analysis of 30 *XP* strains preliminarily revealed their biosynthetic capacity for natural products^15^ by linking the metabolic profile of wild-type strains to known natural-product biosynthetic gene clusters (BGCs)^16^. To accumulate knowledge about the functions of natural product in the context of bacteria–nematode–insect interactions, we and others have been characterizing BGCs for natural-product discovery^7,17–22^. However, these studies have mostly revolved around individual BGCs on a single-genome basis or lacked a comprehensive comparison of intra/interspecies BGCs. This did not reveal to what extent BGCs that might be linked to the special ecological niche are either conserved or unique within *XP* genomes. Therefore, a more systematic approach is needed to create a global BGC map for identifying BGCs of ecological importance across *Xenorhabdus* and/or *Photorhabdus*, as well as for exploring the full biosynthetic capacity of *XP* strains to accelerate genome mining.

In this Article, to provide insights into natural products that may account for the niche specificity of *XP*, we apply genome analysis of 45 *XP* strains that cover all sequenced strains in our collection and represent almost all *XP* taxonomy by combining pangenomic and domain sequence similarity network approaches, homologous BGC expression, chemical structure elucidation and biological assays.

## Results

### An overview of *XP* BGCs

**Fig. 1:**
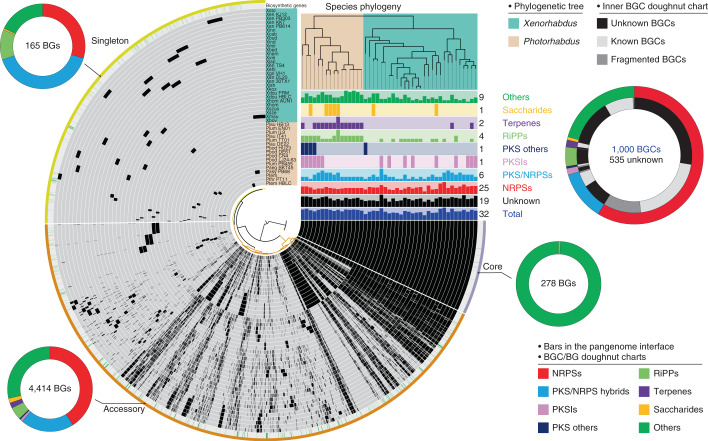
Pangenomic analysis and BGC overview of 45 *XP* genomes by anvi’o. The central plot of the interface represents a hierarchical clustering dendrogram based on gene presence/absence. In the circle interface, each layer (grey) represents all genes (black) in a single genome, the distributions of BGs (green), and, for the bin names, the core region (grey-purple) contains genes present in all 45 *XP* genomes, the accessory region (orange) contains genes common to some *XP* genomes, and the singleton region (yellow) contains species-specific genes present in only one of the genomes. BGC distributions in a strain are represented by the bar charts under the species phylogeny. The maximum number and classification of each BGC are indicated on the right side of the bar charts. The double-layer BGC doughnut chart provides an overview of the proportion of each BGC class (outer layer) and unknown/known/fragmented BGCs (inner layer) in *XP*. Unknown BGCs are clusters without connections to known BGCs in the BiG-SCAPE network (Fig. 3). Known BGCs are previously experimentally identified clusters or those with connections to the MIBiG references in Fig. 3. Fragmented BGCs result from incomplete genome sequencing. BG doughnut charts represent the proportion of the BGC class to which the BGs belong in different pangenomic regions. The numbers of BGCs in total, unknown BGCs and BGs are indicated inside the BGC/BG doughnut charts. Source data

We began by using antiSMASH 5.0 (antibiotics & secondary metabolite analysis shell^23^) to predict and annotate the natural-product BGCs in 29 *Xenorhabdus* and 16 *Photorhabdus* strains (Supplementary Table 1). A total of 1,000 BGCs were detected and categorized into eight classes (Fig. 1, Supplementary Fig. 1 and Supplementary Table 2), corresponding to an average of 22 BGCs per species, which is two- to tenfold higher than the average BGC levels of any other Enterobacteria^24^. Most species show a linear relationship between the number of BGCs and the size of their genome (Supplementary Fig. 2). Compared to *Xenorhabdus*, *Photorhabdus* tends to harbour a larger genome size with more BGCs. Non-ribosomal peptide synthetases (NRPSs) are the most abundant BGC class in *XP*, accounting for 59% of the total BGCs, with ~13 BGCs per species. Owing to the abundance of NRPS BGCs, it seems likely that their products play essential ecological roles. The ‘Others’ group of BGCs composed of various minor classes and hybrid clusters is the second-largest class, the products of which might facilitate bacteria to fulfil specific ecological functions. The polyketide synthase (PKS)/NRPS hybrid class is modestly enriched and broadly distributed. PKS (type I and other PKSs), ribosomally synthesized and post-translationally modified peptide (RiPP), terpene and saccharide BGCs are scant in *XP* compared with the other classes (Supplementary Fig. 3). The biosynthetic gene cluster families (BiG-FAM) database^25^ for gene cluster family (GCF) explorations suggests that 58% of the *XP* GCFs are exclusive (Supplementary Table 3). Therefore, *XP* could be a distinct source for experimental natural product discovery.

### Conserved *XP* BGCs

In the context of prokaryotic genome evolution driven by gene gain and loss over long periods, the gene content of a pangenome that comprises phylogenetically related bacterial species reflects a record of responses to natural selection^26^. Core genes shared by all species in a pangenome are essential for basic biological aspects, whereas accessory and singleton genes presented in some and one species, respectively, are regarded as ‘dispensable’. These ‘dispensable’ genes are still allied to complementary biochemical pathways and functions that might endow bacteria with unique advantages for ecological adaptation^27^. Therefore, we asked a question here, ‘among all predicted BGCs, are there any highly conserved BGCs across *XP* genomes?’ Towards answering this, we performed a pangenome analysis with the anvi’o platform^28,29^ to characterize the gene content of 45 *XP* strains. With the integration of BGC annotations into the pangenome, we could monitor the distributions of genes with natural-product biosynthetic annotations (that is, biosynthetic genes, BGs) in the core, accessory and singleton regions^26^ (Fig. 1). We then set out to filter widespread, consecutive BGs that possibly make up the most prevalent BGCs among different chemical classes. The reasons why we present the most prevalent BGCs by chemical classes are as follows: (1) BGC classes with various biosynthetic logic recruit distinct building blocks (except RiPPs and NRPSs, recruiting identical building blocks) and thereby yield compound classes spanning an enormous range of molecular composition and molecular weight; (2) different compound classes might be biosynthesized and secreted by the *XP* bacteria in different stages of the symbiotic nematode life cycle. Therefore, covering various BGC classes could provide a more comprehensive view of compound classes with distinct biological/physiological functions that would engage in different stages of the life cycle.

**Fig. 2:**
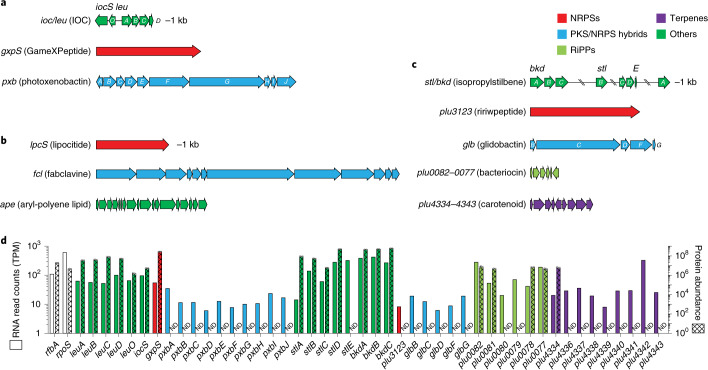
The most widely distributed gene clusters among eight BGC classes in *Xenorhabdus* and/or *Photorhabdus*. **a**, The most conserved BGCs across *XP*, including the previously unidentified *ioc*/*leu* and *pxb*. **b**, The most widely distributed *X*-specific BGCs, including the previously unidentified *lpc*. **c**, The most widely distributed *P*-specific BGCs, including the unknown *plu0082–0077*. **d**, Comparison of the transcriptional and translational levels of genes in the conserved BGCs (*ioc*/*leu*, *gxp*, *pxb*, *stl*/*bkd*, *plu3123*, *glb*, *plu0082–0077* and *plu4334–4343*) in *P. luminescens* subsp. *laumondii* TT01 with the housekeeping genes (*rfbA* and *rpoS*). Proteomic data represent mean ± s.d. from four independent experiments. ND, not detectable; TPM, transcripts per kilobase million. Source data

Surprisingly, although NRPS BGCs are prolific in *XP*, all of their BGs scatter in the accessory and singleton regions (Fig. 1). Almost all BGs that are located in the core region belong to an unknown *ioc*/*leu* BGC, which is a putative β-lactone cluster (Fig. 2a). The *gxpS* (Fig. 2a) responsible for GameXPeptide biosynthesis^30^, located in the accessory region, is the most broadly distributed NRPS GCF across *Xenorhabdus* (72%) and *Photorhabdus* (93%), followed by the antiprotozoal rhabdopeptide/xenortide-like peptides^31^ (Supplementary Fig. 4) that are found in 51% of *Xenorhabdus* and 87% of *Photorhabdus*. A set of five consecutive BGs (*pxbF–J*) in the accessory region composes an unknown cluster (*pxb*; Fig. 2a) representing the most prevalent PKS/NRPS hybrid GCF across *Xenorhabdus* (58%) and *Photorhabdus* (81%).

To scrutinize the prevalence of genus-specific BGCs, we analysed the pangenome of *Xenorhabdus* and *Photorhabdus* separately (Supplementary Fig. 5). NRPS BGs related to the xenoamicin (*xab*) BGC^32^ and eight unknown BGCs are located in the core region of the *Xenorhabdus* pangenome. Among them, an unknown NRPS (*lpcS*; Fig. 2b) stands out because it exists in 96% (28 out of 29) of strains as the most widespread *Xenorhabdus*-specific (*X*-specific) GCF. In the accessory region of the *Xenorhabdus* pangenome, multiple consecutive BGs making up the broad-spectrum antimicrobial fabclavine^33^ BGC (*fcl*; Fig. 2b and Supplementary Fig. 4) are found in 44% of *Xenorhabdus* strains as the most prevalent genus-specific PKS/NRPS hybrid GCF. Sequential consecutive BGs that compose the *ape* BGC (Fig. 2b) are found exclusively in 76% of *Xenorhabdus* strains. The *ape* BGC synthesizing the aryl-polyene lipids^34^ (Supplementary Fig. 4) that protect the bacteria from oxidative stress and promote biofilm formation^34,35^ is the most prominent GCF among Gram-negative bacteria^34,36^. Isopropylstilbene (Fig. 2c and Supplementary Fig. 4) is a multipotent compound and an essential growth factor of dauer-stage nematodes^37^, whose BGs (*stl*/*bkd*) located in the core region are highly conserved across all *Photorhabdus* strains. In the accessory region of the *Photorhabdus* pangenome, BGs of glidobactin (*glb*; a potent eukaryotic proteasome inhibitor^38^), ririwpeptide^39^ (*plu3123*; Supplementary Fig. 4) and carotenoid (*plu4334*–*4343*), as well as an unknown bacteriocin (*plu0082–0077*) make up BGCs that represent the most widespread *Photorhabdus*-specific (*P*-specific) PKS/NRPS hybrid (93%, 15 out of 16), NRPS (87%), terpene (81%) and RiPP (93%) GCFs, respectively (Fig. 2c).

Although these BGCs (Fig. 2a–c) are widespread in *XP*, some of the chemical structures accounting for the biosynthetic pathways remain cryptic. Two major reasons for this might be the BGC being silent in wild-type strains under laboratory conditions, and/or product(s) being undetectable or difficult to isolate. We therefore leveraged our previous transcriptomic and proteomic datasets of *Photorhabdus luminescens* subsp. *laumondii* TT01 wild-type strain^40^ to obtain information about the transcription and translation of the conserved BGCs. The transcriptomic data showed that all conserved BGCs are actively transcribed at different levels (Fig. 2d). However, BGCs encoded by *pxb*, *plu3123*, *glb*, *plu0082–0077* and *plu4334–4343* are partly or completely untranslated, whereas almost all genes belonging to the putative β-lactone (*ioc*/*leu*), GameXPeptide (*gxp*) and isopropylstilbene (*stl*/*bkd*) BGCs are expressed with high protein abundance, comparable to the levels of housekeeping genes (Fig. 2d). The proteomic data, except for the case of the putative β-lactone BGC (*ioc*/*leu*), are in line with the previous metabolic analysis^15^, in which GameXPeptides and isopropylstilbene are the chemotypes in *Photorhabdus* wild-type strains. These findings hint that, among the conserved BGCs yielding previously unidentified natural products, the *pxb*, *plu0082–0077* and *plu4334–4343* are silent clusters due to unknown regulation mechanisms, while the product(s) of β-lactone BGC (*ioc*/*leu*) should be present in the wild-type strain but has yet to be detected and characterized by means of standard spectroscopic methods.

### Unique *XP* BGCs

**Fig. 3:**
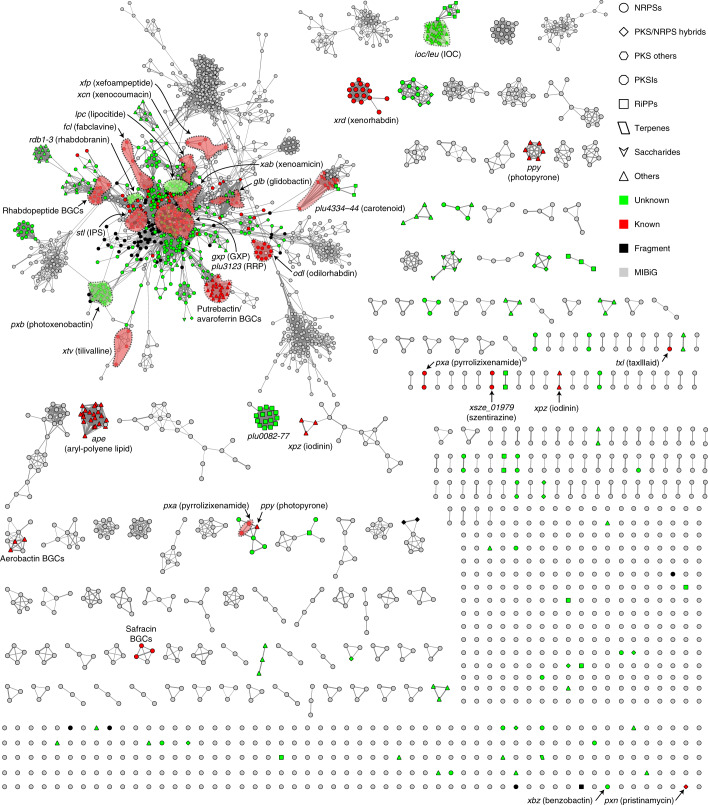
Sequence similarity network of BGCs identified in 45 *XP* genomes by BiG-SCAPE. Previously unidentified BGCs involved in this study and selected known BGCs are annotated and highlighted. BGCs in the main network belonging to a given GCF are not exhaustively highlighted due to nodes being scattered. IPS, isopropylstilbene; GXP, GameXPeptide; RRP, ririwpeptide. Source data

With the unidentified, conserved BGCs in hand, we set out to assess their biosynthetic novelty as well as the thorough biosynthetic capacity of *XP*. We subsequently compared *XP* BGCs against the reference BGCs in the Minimum Information about a Biosynthetic Gene cluster (MIBiG) database^41^ by the biosynthetic gene similarity clustering and prospecting engine (BiG-SCAPE) based on distance metrics^42^. The BiG-SCAPE analysis suggested biosynthetic uniqueness of 535 BGCs (53%) that were found to be unrelated to the MIBiG BGCs and our in-house BGC data. Overall, 46% of NRPS, 61% of PKS/NRPS hybrid, 73% of PKSI, 97% of RiPP, 100% of saccharide and 58% of ‘Other’ BGCs have yet to be identified. The previously unidentified *X*-specific *lpc* BGC, as well as 87% of the known *XP* BGCs (312 entries), including the aforementioned prevalent NRPSs (encoding the biosyntheses of GameXPeptide^30^, rhabdopeptide/xenortide-like peptides^31^ and ririwpeptide^39^) and PKS/NRPS hybrids (encoding the biosyntheses of fabclavine^33^ and glidobactin^38^), are concentrated in the main network (Fig. 3). This indicates they are very similar in terms of domain sequences. Seventy percent of the unknown BGCs (378 entries) distantly related to the known BGCs are mostly on the periphery of the main network (Fig. 3), exemplified by the previously unidentified PKS/NRPS hybrid BGC (*pxb*) that prevails across *XP*. The remaining 30% of the unknown BGCs (157 entries), including the *XP* highly conserved β-lactone (*ioc*/*leu*) and *P*-specific bacteriocin (*plu0082–0077*) BGCs, are classified into 55 GCFs (as 26 isolated clades and 29 singletons) without connections with MIBiG references or the main network, suggesting their underlying biosynthetic novelty.

### A minimal β-lactone in all *XP* is a proteasome inhibitor

**Fig. 4:**
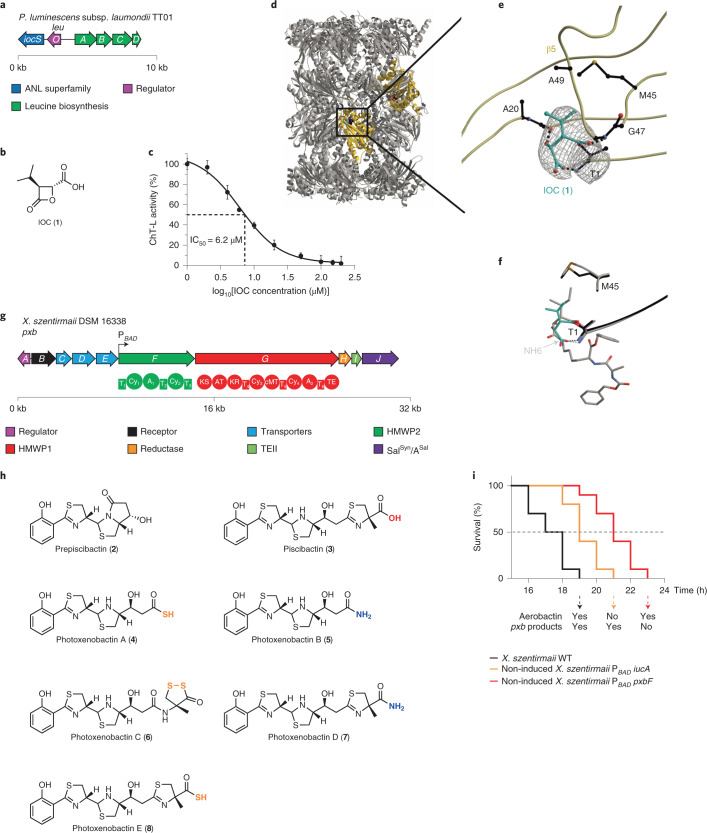
BGCs, chemical structures and bioactivities of IOC and piscibactins/photoxenobactins. **a**, Genetic architecture of the *ioc*/*leu* BGC. **b**, Chemical structure of IOC (**1**). **c**, IC_50_ determination of **1** against the ChT-L activity of the yeast 20S proteasome using the fluorogenic substrate Suc-Leu-Leu-Val-Tyr-AMC (6.2 ± 1.2 µM). Data represent mean ± s.e.m. normalized to a DMSO-treated control from three independent experiments. **d**, Crystal structure of the yeast 20S proteasome in complex with **1** (spherical model, cyan carbon atoms) bound to ChT-L active sites (β5 subunits, gold; PDB 7O2L). **e**, Illustration of the 2*F*_O_ − *F*_C_ electron density map (grey mesh, contoured to 1*σ*) of **1** covalently linked through an ester bond to Thr1O^γ^ of the β5 subunit. Protein residues interacting with **1** are highlighted in black. Dots represent hydrogen bonds between **1** and the protein residues. **f**, Superposition of **1** (cyan) and homobelactosin C (grey; PDB 3E47)^51^ complex structures with the yeast 20S proteasome highlights similar conformations at the ChT-L active site. **g**, Genetic architecture of the *pxb* BGC and domain organization. A black arrow shows the position where an l-arabinose-inducible promoter P_*BAD*_ is inserted. T, thiolation; A, adenylation; Cy, heterocyclization; KS, ketosynthase; AT, acyltransferase; KR, ketoreductase; cMT, carbon methyltransferase; TE, thioesterase domains. **h**, Known chemical structures of prepiscibactin (**2**) and piscibactin (**3**) from *Photobacterium damselae* subsp. *piscida*^57^, as well as previously unidentified photoxenobactins A*–*E (**4***–***8**) from *X. szentirmaii* DSM 16338. The terminal heteroatoms are highlighted. **i**, Survival curve of *G. mellonella* larvae (ten insects per strain) infected with *X. szentirmaii* wild-type (79 cells), non-induced *X. szentirmaii* P_*BAD*_ *iucA* mutant (81 cells) and non-induced *X. szentirmaii* P_*BAD*_ *pxbF* mutant (90 cells). LT_50_ (median lethal time): wild-type, 16.9 h; non-induced P_*BAD*_ *iucA* mutant, 18.6 h; non-induced P_*BAD*_ *pxbF*, 20.3 h mutant. The LT_50_ time point is indicated with a grey dashed line. ΔLT_50_ = LT_50_^mutant^ – LT_50_^wild-type^. The *iuc* BGC encodes the biosynthesis of aerobactin in *X. szentirmaii*^60^. Under a non-induced condition during insect injection assays, the *X. szentirmaii* P_*BAD*_ *iucA* and *X. szentirmaii* P_*BAD*_ *pxbF* mutants are equivalent to corresponding BGC knockout mutants. In **a** and **g**, kb, kilobase.

Recognizing that the putative β-lactone BGC is highly expressed under normal laboratory conditions (Fig. 2d) prompts us to predict a possible chemical structure based on the functions of biosynthetic genes, which might facilitate identification of the authentic product by re-examining the metabolic profile of wild-type strains. The BGC features six genes (Fig. 4a and Supplementary Table 4). *leuABCD* are involved in l-leucine biosynthesis. *leuO* is positioned next to *leuA* and encodes a global transcription factor involved in regulating natural-product biosynthesis^43^ and other physiological traits^44^. *iocS* encodes an enzyme belonging to the ANL (acyl-CoA synthetases, NRPS adenylation domains and luciferase enzymes) superfamily. Such a gene architecture is reminiscent of the biosynthesis of cystargolides^45^, during which 3-isopropylmalate as an intermediate in the leucine pathway is the precursor for one-step lactonization to afford 3-isopropyl-4-oxo-2-oxetanecarboxylic acid (IOC, **1**) with a β-lactone moiety (Supplementary Fig. 6). Although the enzyme responsible for β-lactonization remains uncharacterized in the cystargolide biosynthesis^45^, a recent report demonstrates the acyl-AMP ligase, OleC, to be a β-lactone synthetase during the biosynthesis of long-chain olefinic hydrocarbons^46^. Therefore, we speculated that IocS might be responsible for adenylating the 4-carboxyl group and then triggering lactonization to give IOC (**1**).

To detect the putative β-lactone, we cultured *P. luminescens* subsp. *laumondii* TT01, *Xenorhabdus nematophila* ATCC 19061, and *Xenorhabdus szentirmaii* DSM 16338 wild-type strains in various media. By HPLC–high-resolution mass spectrometry (HPLC-HRMS) analysis of the culture supernatant from Sf-900 (a serum-free insect cell medium) with a negative ion mode, we did detect a peak with *m*/*z* 157.0508 [M–H]^−^, whose deduced sum formula, C_7_H_9_O_4_, coincides with that of **1** (Supplementary Table 5). Finally, (2*R*,3*S*)-**1** was synthesized and demonstrated a retention time and MS/MS fragmentation patterns identical to those of **1** in HPLC-HRMS (Supplementary Fig. 7), confirming the planar structure and tentative stereochemistry of **1** (Fig. 4b).

The ubiquitin-proteasome system responsible for degrading misfolded and malfunctioning proteins in eukaryotes plays an essential role in cell-cycle regulation and apoptosis^47^. The system is also involved in degrading repressors of the insect immune response cascade^48^. The proteasome 20S core particle, the catalytic core of the system, is assembled from four stacked heptameric rings adopting an α_1–7_β_1–7_β_1–7_α_1–7_ stoichiometry^49^. The active-site nucleophile of each proteolytic centre is an N-terminal threonine (Thr1) located at subunits β1 (caspase-like activity), β2 (trypsin-like activity) and β5 (chymotrypsin-like (ChT-L) activity)^50^. Natural products featuring a β-lactone moiety, such as omuralide, belactosins and cystargolides, have been proven to suppress the proteolytic activity of the core particle^46,51^. Their uniform mode of proteasome inhibition relies on opening of the β-lactone and transesterification upon nucleophilic attack by the catalytic N-terminal threonine (Thr1O^γ^)^52^. Nevertheless, β-lactone natural products differ substantially in their chemical structures and thus in their mode of binding. Inspired by cystargolides and belactosins containing an IOC moiety as the reactive head group^51,53^, we assumed that IOC (**1**) might represent the smallest β-lactone that still blocks the activity of the proteasome. Indeed, **1** inhibits the yeast 20S proteasome with a half-maximum inhibitory concentration (IC_50_) value of 6.2 µM for the β5 subunit (Fig. 4c), whereas it has low binding affinities for β1 (625 µM) and β2 (60 µM). We thus solved the crystal structure of **1** in complex with the yeast 20S proteasome at 3.0 Å (PDB 7O2L). The electron density map displayed **1** covalently bound to Thr1O^γ^ of all active sites due to the high ligand concentrations used for crystal soaking (Fig. 4d). However, because **1** lacks strong interactions with protein residues in the caspase- and trypsin-like binding channels, the 2*F*_O_–*F*_C_ map for the ligand is diffuse at β1 and β2. By contrast, **1** is well defined in the β5 subunit (Fig. 4e). Superposition of **1** with known complex structures reveals a similar conformation as observed for the class of belactosins^51,54^ (Fig. 4f and Supplementary Fig. 8). The acyl-oxygen atom of **1** derived from β-lactone ring-opening is stabilized by the oxyanion hole (Gly47NH), whereas the generated hydroxyl group is hydrogen-bonded to the carbonyl oxygen of residue 19. Similar to NH6 in belactosin products^52^, the carboxylate group of **1** interacts with the threonine N terminus and displaces the nucleophilic water molecule (Fig. 4e), thereby preventing hydrolysis of the acyl enzyme complex, and explaining its inhibitory effect. Furthermore, the isopropyl moiety of **1** at the P1 site is stabilized by Ala20, Met45 and Ala49 in the ChT-L channel. Although these interactions are present in other β-lactone-containing compounds, they adopt a diverse and unpredictable mode of binding. Without nitrogen atoms and extension units, **1** might feature the minimal scaffold for proteasome inhibition. Therefore, **1** could be an *XP* universal virulence factor against insects, as well as soil-living food competitors like protozoa, that disturbs the ubiquitin-proteasome system and thereby causes cell-cycle disturbance and immunodeficiency.

### The most prevalent PK/NRP hybrid in *XP* is insecticidal

The prevalent PKS/NRPS hybrid GCF containing 32 *pxb* (photoxenobactin) BGCs is shown to have weak similarity to micacocidin^55^ and yersiniabactin^56^ BGCs in the BiG-SCAPE network (Fig. 3). Notably, compared with HMWP1 encoded by the yersiniabactin BGC in *Yersinia pestis*^56^, its homologue (PxbG) lacks one carbon-methyltransferase domain (cMT_1_) involved in the bismethylation of a C2 polyketide moiety in yersiniabactin. Moreover, PxbG embeds an additional module comprising a heterocyclization domain, an adenylation domain and a thiolation domain (Cy_4_–A_2_–T_6_; Fig. 4g and Supplementary Fig. 9).

To unveil the underlying biosynthetic theme of *pxb* BGC, we overexpressed the cluster in *X. szentirmaii* DSM 16338 by using a promoter exchange strategy^19^ to insert a P_*BAD*_ promoter in front of *pxbF*. Besides prepiscibactin (**2**) and piscibactin (**3**)^57^, the *X. szentirmaii* P_*BAD*_ *pxbF* mutant yielded four additional compounds, termed photoxenobactins A–D (**4**–**7**; Fig. 4h and Supplementary Fig. 10). From a 20-l fermentation broth of the *X. szentirmaii* P_*BAD*_ *pxbF ∆hfq* mutant, which produced the desired compounds with a reduced background of other natural products^19^, we obtained **4**–**6**, as well as photoxenobactin E (**8**; Supplementary Fig. 10). The chemical structures of **4**, **5** and **8** were readily elucidated by HRMS and NMR spectroscopic methods, and that of **7** was confirmed by tandem MS and isotope labelling experiments (Supplementary Figs. 11 and 12 and Supplementary Table 6), revealing that, unexpectedly, **4**, **5**, **7** and **8** have various chain lengths and termini such as thiocarboxylic acid (**4** and **8**) and carboxamide (**5** and **7**). Although the production titre of photoxenobactin C (**6**) in the *X. szentirmaii* P_*BAD*_ *pxbF* mutant appeared to be sufficient for isolation, we only obtained a trace amount of the pure compound. Photophobia and thermo-instability in any kind of organic solvents are the culprits, leading to conversion into an array of rearranged products, such as methyl ester piscibactin (**9**) in methanol (Supplementary Fig. 13). Finally, combining extensive labelling experiments (Supplementary Figs. 14 and 15), and 2D NMR data (Supplementary Fig. 12), we proved that **6** bears a unique dithioperoxoate moiety.

Inspired by piscibactin being able to chelate gallium and ferric ions^57^, we set out to explore whether photoxenobactins are metallophores, because metallophores are essential for bacteria to acquire trace elements from environments and can have additional functions (for example, toxicity, signalling, protection and antibiotics)^58^. A fraction mainly containing *pxb* BGC products was incubated with different inorganic metal salts (for example, Ga^III^, Fe^III^, Cu^II^, Zn^II^, Mo^VI^ and V^V^), and only piscibactin-Ga^III^/Fe^III^/Cu^II^ (**10**–**12**) and photoxenobactin D-Ga^III^/Fe^III^/Cu^II^ (**13**–**15**) were detected (Supplementary Fig. 16). An earlier report describes killing of *Galleria mellonella* upon injection of *Escherichia coli* carrying a *pxb* BGC from *Photorhabdus asymbiotica*. Ulbactin E and a compound with the sum formula C_20_H_25_O_4_N_3_S_3_, which was a putative desmethyl yersiniabactin, were found in the methanol extract of insect carcasses, suggesting both compounds as virulence factors against insects^59^. Indeed, C_20_H_25_O_4_N_3_S_3_ coincides with methyl ester piscibactin (**9**), a rearranged product of **6** that occurs in methanol, as observed herein (Supplementary Fig. 13). We thus reasoned that **6** should be one of the authentic insecticidal compounds. Next, we attempted to re-examine the toxicity of *pxb* BGC products during the insect infection process by comparing it with that of aerobactin, an identified virulence-related siderophore in *X. szentirmaii*^60^. Because the *X. szentirmaii* P_*BAD*_ *pxbF* mutant synthesizes **2**–**7** only upon the induction of l-arabinose, none of the compounds can be produced by the mutant inside insects due to the absence of the l-arabinose inducer. Hence, a non-induced promoter exchange mutant is equal to a BGC knockout strain. We then injected *X. szentirmaii* wild-type strain, which produces **2**–**7** and toxic aerobactin encoded by the *iuc* BGC^60^, as well as the non-induced *X. szentirmaii* P_*BAD*_ *iucA* and non-induced *X. szentirmaii* P_*BAD*_ *pxbF* mutants into *G. mellonella* larvae (Fig. 4i). The wild-type strain killed insects 3.4 h faster than the non-induced P_*BAD*_ *pxbF* mutant. Furthermore, the *pxb* BGC products exerted a greater impact on insect virulence than aerobactin in that the non-induced P_*BAD*_ *iucA* mutant killed insects 1.7 h faster than the non-induced P_*BAD*_ *pxbF* mutant.

### The most widespread NRP in *XP* suppresses insect immunity

**Fig. 5:**
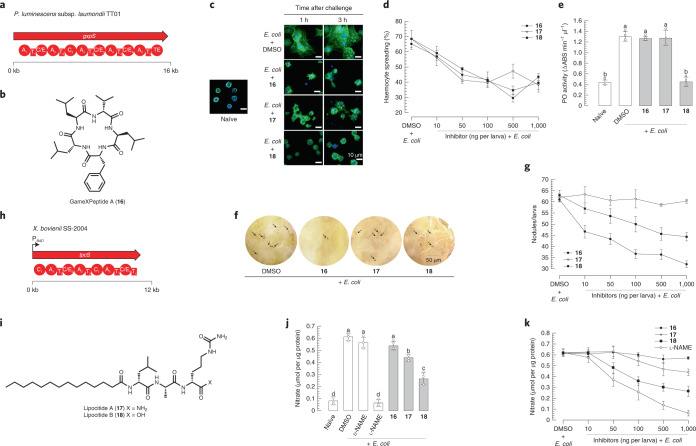
BGCs, chemical structures and bioactivities of GameXPeptide A and lipocitides. **a**, Domain organization of GxpS. **b**, Chemical structure of GameXPeptide A (**16**). **c**, In vivo observation of haemocytes-spreading behaviour in different time intervals upon injection of **16**–**18** (1,000 ng per larva) in *S. exigua* larvae. Blue, nucleus; green, actin cytoskeleton. *n* = 1 biologically independent larva per experiment over three independent experiments. **d**, In vitro analysis of haemocyte-spreading behaviour. **16**–**18** suppressed haemocyte spreading in a dose-dependent manner, with IC_50_ values of 17.2, 10.0 and 26.2 ng per larva, respectively. *n* = 100 cells per larva were randomly chosen for counting spread cells over three independent experiments. **e**, Suppression of phenoloxidase (PO) activity in *S. exigua* larvae by **18** (1,000 ng per larva). *n* = 1 biologically independent larva per experiment over three independent experiments. **f**, Suppression of haemocyte nodule formation in *S. exigua* larvae by **16** and **18** (1,000 ng per larva). Nodules were counted at 8 h post infection (black spots, indicated by black arrows). *n* = 5 biologically independent larvae per experiment over three independent experiments. **g**, Dose-dependent suppression of nodule formation by **16** and **18**, with IC_50_ values of 25.8 and 86.1 ng per larva, respectively. *n* = 5 biologically independent larvae per experiment over three independent experiments. **h**, Domain organization of the LpcS. A black arrow shows the position where an l-arabinose-inducible promoter P_*BAD*_ is inserted. **i**, Previously unidentified chemical structures of lipocitides A (**17**) and B (**18**) from *X. bovienii* SS-2004. **j**, Suppression of NO production in the haemolymph of *S. exigua* larvae injected with **17** and **18** (1,000 ng per larva). *n* = 3 biologically independent larvae per experiment over three independent experiments. **k**, Dose-dependent suppression of NO production in the haemolymph of *S. exigua* larvae by **17** and **18**. l-NAME (N_ω_-nitro-l-arginine methyl ester hydrochloride) and d-NAME (N_ω_-nitro-d-arginine methyl ester hydrochloride) are used as controls. *n* = 3 biologically independent larvae per experiment over three independent experiments. In **a** and **h**, kb, kilobase; A, adenylation; T, thiolation; C, condensation; C/E, condensation/epimerization; E, epimerization; TE, thioesterase domains. In **d**, **e**, **g**, **j** and **k**, data represent mean ± s.d. Letters above standard error bars indicate significant differences among means at type I error = 0.05 (LSD test).

GxpS, an NRPS with five modules (Fig. 5a), is responsible for the biosynthesis of GameXPeptides, a class of cyclic pentapeptides composed of valine, leucine and phenylalanine (Fig. 5b). Although GameXPeptides are one of the diagnostic chemotypes with high production titres in almost all *XP*^15^, their function has remained cryptic over the past decade. Our recent bioactivity screening for crude extracts produced by specifically overexpressed mutants^19^ indicated that GameXPeptides might be one of the bioactive contributors of wild-type strains inhibiting in vitro production of prostaglandin E_2_ without cytotoxicity and antimicrobial activity. With the synthetic GameXPeptide A (**16**) in hand, we therefore pursued its possible suppression of insect immune responses. Insects rely on innate immunity consisting of cellular and humoral immune responses to overcome infections^61^. Cellular immune responses mediated by eicosanoids involve encapsulation that is performed by immune haemocytes along with morphological changes, melanization activated by phenoloxidase, nodulation and phagocytosis^62^. The cytoplasmic extension observed in the haemocytes of the lepidopteran insect *Spodoptera exigua*, as an immune response to the *E. coli* challenge, was remarkably inhibited by **16** (Fig. 5c) in a dose-dependent manner (Fig. 5d), with an IC_50_ value of 17.2 ng per larva (Supplementary Table 7). Although **16** exerted no suppression against the phenoloxidase activation (Fig. 5e), it remarkably decreased the number of nodules formed (Fig. 5f) in a dose-dependent manner (Fig. 5g) with an IC_50_ value of 25.8 ng per larva (Supplementary Table 8). These results suggest that **16** specifically suppresses insect haemocyte spreading and nodule formation upon insects being challenged by *E. coli*, and thereby defeats the insect cellular immune response. It is worth mentioning that the inhibitions of phenoloxidase activity and the proteolytic cascade leading to active phenoloxidase are accomplished by two known widespread compound classes, rhabduscin^63^ and rhabdopeptide/xenortide-like peptides^7^, respectively. Consequently, the functional characterization of ubiquitous GameXPeptides is a substantial advance toward deconstructing *XP* to suppress insect immune systems during symbiotic nematode invasion.

### The universal product in *Xenorhabdus* inhibits the insect NO pathway

The most broadly distributed *X*-specific GCF existing in all but *Xenorhabdus cabanillasii* JM26 is centralized in the main BiG-SCAPE network and displays a degree of relatedness with the *xcn* (xenocoumacin)^64^ and *fcl* (fabclavine)^33^ GCFs (Fig. 3). We designated this *X*-specific cluster as *lpc*, which encodes a tetramodular NRPS with an unusual terminal thiolation–condensation/epimerization–thiolation (T_3_–C/E_4_–T_4_) domain architecture (Fig. 5h). This BGC is silent under laboratory conditions, consistent with the transcriptional level of *lpcS* in *X. szentirmaii* US wild-type strain^40^ being about 16-fold lower than those of the housekeeping genes (Supplementary Fig. 17). We were able to activate the BGC in *Xenorhabdus bovienii* SS-2004 by the promoter exchange strategy. The *X. bovienii* P_*BAD*_ *lpcS* mutant produced an array of N-terminal acylated linear tripeptides (Supplementary Fig. 18). Two major products, lipocitides A and B (**17** and **18**; Fig. 5i), were purified, and their structures were identified by NMR spectroscopy (Supplementary Fig. 12 and Supplementary Table 9), Marfey’s method (Supplementary Fig. 19) and chemical synthesis, revealing that **17** and **18** bear a myristoyl and a consecutive amino-acid sequence of d-leucine/l-alanine/d-citrulline, as well as a carboxamide and carboxylic acid in their respective C termini. Comparison of the tandem MS of the other lipocitides in *X. bovienii* SS-2004 with **17** and **18** revealed that lipocitides feature either d-leucine/l-alanine/d-citrulline-OH or d-leucine/l-alanine/d-citrulline-NH_2_ as a backbone and differ in the N-acyl substitutions (Supplementary Fig. 20). Identical compounds, termed bovienimides, as well as a recognition of the BGC conservativeness, were reported by the Crawford laboratory^65^ during revision of this manuscript.

Nitric oxide (NO) converted from l-arginine by NO synthases is an upstream component of the eicosanoid signalling pathway to trigger insect innate immune responses against exogenous challenges^62^. Inspired by l-citrulline and arginine-derived compounds being inhibitors of NO synthesis^66^, we examined whether the major lipocitides, **17** and **18**, could inhibit NO production to defeat insect immune responses. The elevated NO level measured by the nitrate concentration in the haemolymph of *S. exigua* larvae caused by *E. coli* infection was suppressed by both compounds (Fig. 5j) in a dose-dependent manner (Fig. 5k), with IC_50_ values of 2.37 and 0.42 μg per larva, respectively (Supplementary Table 10). Earlier reports showed that NO activates phospholipases A_2_ for producing downstream eicosanoid signalling molecules^67^, thereby mediating cellular immune responses. Both **17** and **18** suppressed cytoplasmic extension in the haemocytes of *S. exigua* following *E. coli* challenge (Fig. 5c,d and Supplementary Table 7). In addition, **18** significantly suppressed phenoloxidase activation (Fig. 5e) and decreased the number of nodules formed (Fig. 5f,g and Supplementary Table 8). These results indicate that lipocitides suppress insect NO production, which leads to sequential inhibitions of cellular immune responses and thus might cause fatal immunosuppressive conditions for the insects under infection by the *Xenorhabdus* symbiotic nematode. In contrast, GameXPeptide A (**16**) displayed no suppression of NO production (Fig. 5j,k), which indicates that GameXPeptides have a different upstream target from lipocitides or mediate other signalling transduction pathways.

### T-shape PK/NRP hybrid with prodrug activation mechanism

**Fig. 6:**
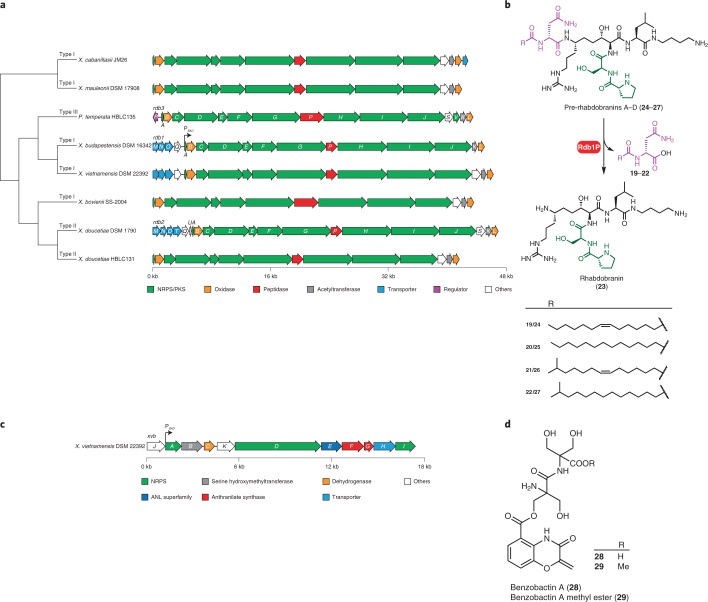
Representative BGCs of uniqueness and chemical structures thereof in *XP*. **a**, Phylogeny and gene organization of the *rdb* BGCs. The phylogenetic tree is based on the protein sequences of BGCs. BGC subclassification is indicated next to the branch. **b**, Chemical structures of previously unidentified pre-rhabdobranins A–D (**24**–**27**) and rhabdobranin (**23**) from *X. budapestensis* DSM 16342, as well as the proposed late-stage biosynthesis involved in a prodrug activation mechanism, similar to xenocoumacin and colibactin. The N-terminus capped acylated d-asparaginyl moiety (**19**–**22**) and the dipeptidyl branch are highlighted in pink and green, respectively. The stereocentres were predicted by analysing the conserved motif in condensation and ketoreductase domains that are responsible for stereocontrol. **c**, Genetic architecture of the *xvb* BGC. **d**, Previously unidentified benzobactins A (**28**) and a methyl ester thereof (**29**) from *X. vietnamensis* DSM 22392. In **a** and **c**, a black arrow shows the position where an l-arabinose-inducible promoter P_*BAD*_ is inserted.

The above survey of previously unidentified conserved BGCs has showcased the abilities of *XP* to produce pervasive and structurally unique natural products. We then set out to examine the uncharacterized BGCs that only exist in specific species to assess the biosynthetic potential of *XP*. In the BiG-SCAPE main network (Fig. 3), eight unknown PKS/NRPS hybrid BGCs from seven *Xenorhabdus* and one *Photorhabdus* strains (Fig. 6a) compose a GCF, termed *rdb* (rhabdobranin). The *rdb* BGCs feature a peptidase encoded gene, suggesting a prodrug activation mechanism similar to the biosyntheses of xenocoumacin and amicoumacin, which are potent antibiotics inhibiting messenger RNA translation^7^ and colibactin, which is a genotoxin alkylating DNA^10^. Although the nodes of the *rdb* GCF are adjacent to those of the rhabdopeptide/xenortide-like BGCs, the *rdb* BGCs connect neither to amicoumacin and xenocoumacin^64^ BGCs nor to any MIBiG entries. We classified these eight highly similar BGCs into three types, *rdb1*–*3*, based on the presence or absence of the first adenylation domain in RdbH and the thioesterase domain in RdbI (Supplementary Fig. 21), which might lead to products with distinct numbers of amino-acid residues and nonlinear biosynthetic assembly line logic, respectively.

To identify products derived from this GCF, we focused on *rdb1*, which contains five out of eight BGCs in this GCF, and attempted to activate the *rdb1* in *Xenorhabdus budapestensis* DSM 16342 by inserting a P_*BAD*_ promoter in front of *rdb1A*. The *X. budapestensis* P_*BAD*_ *rdb1A* mutant yielded four *N*-myristoyl-d-asparagine congeners (**19**–**22**), as well as a non-XAD-resin-bound hydrophilic compound with a low production level (**23**; Supplementary Fig. 22). Because an acylated d-asparaginyl capping the N terminus of xenocoumacin, zwittermicin and colibactin has been found to be a self-resistance mechanism^68^, the detection of *N*-myristoyl-d-asparagine analogues was consistent with our hypothesis that a prodrug strategy was involved in the *rdb* biosynthesis. To accumulate the inactive prodrugs for structural identification, we deleted the peptidase encoded gene *rdb1P*, and the *X. budapestensis* P_*BAD*_ *rdb1A ∆rdb1P* mutant led to loss of **19**–**22** and high production of four new peaks with larger masses, designated as pre-rhabdobranins A–D (**24**–**27**; Fig. 6b) with differences in the N-acylated moiety. Pre-rhabdobranin D (**27**) was purified from the *X. budapestensis* P_*BAD*_ *rdb1A ∆rdb1P ∆hfq*, and its structure was determined by HRMS and NMR spectroscopy (Supplementary Tables 5, 11 and 12 and Supplementary Fig. 12). Intriguingly, pre-rhabdobranins are characterized by a proline-serine dipeptidyl side chain that branches off at the N atom of an aminomalonyl building block. To the best of our knowledge, this represents an uncommon T-shape peptide, in contrast to the canonical linear-chain-elongation on thiotemplated assembly lines.

### Orphan assembly line recruits non-canonical building blocks

BGCs as singletons in the BiG-SCAPE network could be ideal test cases for genome mining for novel natural product discovery. We selected an NRPS BGC termed *xvb* (*X.vietnamensis* DSM 22392 benzobactins) for characterization (Fig. 6c). The BGC encodes adenylation domains with unpredictable substrate specificity and specialized tailoring enzymes for substrate modification (for example, a putative serine hydroxymethyltransferase encoded by *xvbB*), as well as synthases for non-amino-acid substrates (two putative anthranilate synthases encoded by *xvbF* and *xvbG*). These indicate the *xbv* product(s) might contain non-canonical building blocks. To determine the product(s) derived from this orphan BGC, we inserted a P_*BAD*_ promoter to express the *xvb* BGC that yielded benzobactin A (**28**) and its methyl ester (**29**; Supplementary Fig. 23). Their structures were confirmed by HRMS and NMR spectroscopy methods (Supplementary Tables 5 and 13 and Supplementary Fig. 12), revealing that **28** and **29** feature a rare benzoxazolinate moiety that has only been found in C-1027^69^ and ashimides^70^ from *Streptomyces*, as well as a non-proteinogenic amino-acid residue, 2-hydroxymethylserine, which is a rare building block in natural products (Fig. 6d). **28** showed cytotoxic activity against the HepG2 cell line with an IC_50_ value of 19.0 µg ml^−1^.

## Discussion

On the journey to decode the roles of *XP* natural products in mediating bacteria–nematode–insect interactions in the ecological niche, we previously carried out a metabolic exploration of 30 *XP* strains by rapid MS-based network analysis^15^. This revealed that the wild-type strains produce a plethora of natural products, most of which belong to the compound class of non-ribosomal peptides. However, in general, the MS-based network approach is constrained by (1) BGCs that are transcriptionally or translationally silent under standard laboratory conditions (for example, BGC expressions need to be in an insect-mimicking medium^40,71^ or under iron-limited conditions^60^) and (2) compounds that are membrane-bound (for example, aryl-polyene lipids^34^) and that are difficult to detect by standard LC/MS methods (for example, compounds that are extremely hydrophilic/hydrophobic, too small/large or poorly ionized/fragmented). Here, to overcome the limitations of the metabolic analysis, we take the ‘BGCs first’ strategy, because BGCs account for the genomic capacity of a strain for producing natural products (see also Extended Discussion in the Supplementary Information).

All the *XP* species live in nearly the same ecological niche, but they harbour BGCs that are distinctive in terms of numbers and classes. For example, the number of BGCs in *Xenorhabdus indica* DSM 17382 is three times that in *Xenorhabdus japonica* DSM 16552. *Photorhabdus temperata* subsp. *thracensis* DSM 15199 features seven BGC classes, whereas *X. japonica* DSM 16552 only has three classes (Supplementary Fig. 1). Therefore, we assume that such deviations among *XP* species are possibly indicative of a minimum number of required BGCs—the highly conserved BGCs—for *XP* to maintain their lifestyle adaptation. The *ioc*/*leu* BGC responsible for IOC (**1**) biosynthesis was present across all *XP* genomes, but none of the NRPS GCFs universally exist in every *XP* species, though the NRPSs are the most abundant class. Indeed, the *ioc*/*leu* BGC is also widely distributed in other γ-Proteobacteria, such as the free-living pathogens *Vibrio cholerae* and *Y. pestis* (Supplementary Table 3). Although this BGC has yet to be studied in other microorganisms and the degree of structural conservation of IOC (**1**) among γ-Proteobacteria is unknown, it is conceivable that the conservation of structural genes *leuA*–*D* for l-leucine biosynthesis and *iocS* for putative lactonization can serve as an indicator that IOC (**1**) is highly conserved among γ-Proteobacteria inhibiting eukaryotic proteasomes. The *pxb* BGC, as the most widespread PKS/NRPS hybrid GCF across *XP*, produces piscibactins (**2** and **3**) and photoxenobactins (**4**–**8**), both of which are structurally related to yersiniabactin but with different chain lengths and C termini. In contrast to the precise target-oriented biosynthesis of the yersiniabactin BGC, it appears that the *pxb* BGC is more diversity-oriented, although the biosynthetic machinery remains cryptic. Yersiniabactin, with high affinities for ferric iron, contributes to the virulence of human pathogens like *Y. pestis and E. coli*^72^. Our study showed that the *pxb* products are associated with the insecticidal activity of *X. szentirmaii*, but only piscibactin (**3**) and photoxenobactin D (**7**) retain metal-chelating abilities. This suggests that the other *pxb* products might be non-metal-chelation virulence factors against insects. In particular, photoxenobactin C (**6**) with a dithioperoxoate moiety is highly reactive and thus might account for the overall insecticidal activity. As GameXPeptides and lipocitides are insect immunosuppressants targeting different transduction pathways, both compound classes could synergistically contribute to a potent overall effect from which producer strains can benefit. The chemical structure identification and functional characterization of the most ubiquitous *Xenorhabdus* and/or *Photorhabdus* natural products have made substantial progress towards deconstructing the niche specificity of *XP*.

*XP* adaptation to the harsh environment and competition against other soil microorganisms might be a driving force for selecting valuable BGCs that produce highly efficacious natural products. Combining the pangenomic and sequence similarity network approaches provides deeper insights into the BGCs responsible for natural product formation, and thereby allows more systematic inference of associations as to the underlying roles of widespread or unique natural products in the ecological niche. Such a combined approach can also be applied to microbiomes from other niches to narrow down the list of candidate BGCs that probably encode ecologically important natural products. With the functional characterization of the most conserved *XP* natural products, future detailed analysis of their targets, as well as potential synergistic/antagonistic interactions among different compound classes (for example, the synergistic immune suppression of GameXPeptides and lipocitides), might lead to a more comprehensive understanding of how *XP* orchestrate the interplay of natural products to maintain the symbiotic lifestyle.

## Methods

### General experimental procedures

All chemicals were purchased from Sigma-Aldrich, Acros Organics or Iris BIOTECH. Isotope-labelled chemicals were purchased from Cambridge Isotope Laboratories. Genomic DNA of selected *Xenorhabdus* and *Photorhabdus* strains was isolated using the Qiagen Gentra Puregene Yeast/Bact Kit. DNA polymerases (Taq, Phusion and Q5) and restriction enzymes were purchased from New England Biolabs or Thermo Fisher Scientific. DNA primers were purchased from Eurofins MWG Operon. PCR amplifications were carried out on thermocyclers (SensoQuest). Polymerases were used according to the manufacturers’ instructions. DNA purification was performed from 1% Tris-acetate-EDTA (TAE) agarose gel using an Invisorb Spin DNA Extraction Kit (STRATEC Biomedical AG). Plasmids in *E. coli* were isolated by alkaline lysis. HPLC-UV-MS analysis was conducted on an UltiMate 3000 system (Thermo Fisher) coupled to an AmaZonX mass spectrometer (Bruker) with an ACQUITY UPLC BEH C18 column (130 Å, 2.1 mm × 100 mm, 1.7-μm particle size, Waters) at a flow rate of 0.6 ml min^−1^ (5–95% acetonitrile/water with 0.1% formic acid, vol/vol, 16 min, UV detection wavelength 190–800 nm). HPLC-UV-HRMS analysis was conducted on an UltiMate 3000 system (Thermo Fisher) coupled to an Impact II qTof mass spectrometer (Bruker) with an ACQUITY UPLC BEH C18 column (130 Å, 2.1 mm × 100 mm, 1.7-μm particle size, Waters) at a flow rate of 0.4 ml min^−1^ (5–95% acetonitrile/water with 0.1% formic acid, vol/vol, 16 min, UV detection wavelength 190–800 nm). Flash purification was performed on a Biotage SP1 flash purification system (Biotage) by a C_18_ main column (Interchim, PF50C18HP-F0080, 120 g) with a self-packed pre-column (Interchim, PF-DLE-F0012, Puriflash dry-load empty F0012 Flash column) coupled with a UV detector. HPLC purification was performed on preparative and semipreparative Agilent 1260 systems coupled to a diode array detector (DAD) and a single quadrupole detector with a C18 ZORBAX Eclipse XDB column (9.4 mm × 250 mm, 5 μm, 3 ml min^−1^; 21.2 mm × 250 mm, 5 μm, 20 ml min^−1^; 50 mm × 250 mm, 10 μm, 40 ml min^−1^). Freeze drying was performed using a BUCHI Lyovapor L-300 Continuous system. NMR experiments were carried out on a Bruker AVANCE 500-, 600- or 700-MHz spectrometer equipped with a 5-mm cryoprobe. 2*R*,3*S*-IOC (**1**) and GameXPeptide A (**16**) were synthesized by WuXi App Tec following the literature (ref. ^73^ for 2*R*,3*S*-**1** and ref. ^74^ for **16**).

### Genome sequencing, assembly and annotation

Isolated DNA was sequenced on the Illumina NextSeq 500 platform. DNA libraries were constructed using the Nextera XT DNA preparation kit (Illumina) and whole-genome sequencing was performed using 2× 150-bp paired-end chemistry. A sequencing depth of >50× was targeted for each sample. Adapters and low-quality ends were trimmed with Trimmomatic 0.39 (ref. ^75^) and the parameters [2:30:10 LEADING:3 TRAILING:3 SLIDINGWINDOW:4:10 MINLEN:12] using a database of adapter sequences as provided by Illumina. All genomes were assembled using SPAdes v. 3.10.1 (ref. ^76^) executed with the following parameters: --cov-cutoff auto --careful in paired-end mode plus mate pairs (in cases where accompanying mate-pair libraries were available). Genome annotation was performed using Prokka v. 1.12 (ref. ^77^) with the following parameters: --usegenus--genus GENUS--addgenes--evalue 0.0001--rfam--kingdom Bacteria--gcode 11--gram --mincontiglen 200. Geneious Prime 2021 was used in genome visualization and analysis.

### antiSMASH annotations and BiG-FAM preliminary classification

The antiSMASH 5.0 (ref. ^23^) web server was employed to mine all the genome sequences for the presence of putative natural-product BGCs. The annotations were conducted using default settings with the extended parameters of ClusterBlast, Cluster Pfam analysis and Pfam-based GO term annotation. The annotated BGCs were summarized for each strain (Supplementary Fig. 1) and visualized in the anvi’o 6.1 (refs. ^28,78^) layers (Fig. 1 and Supplementary Figs. 3 and 5). We then submitted the antiSMASH job IDs to the biosynthetic gene cluster families database (BiG-FAM 1.0.0)^25^ for preliminary GCF explorations and classifications of annotated BGCs (Supplementary Table 3), followed by BiG-SCAPE 1.0.0 (ref. ^42^) refinement with a cutoff of 0.65 (Source Data Fig. 3). The GCFs were double-checked manually via the interactive network (Fig. 3), and corrections were made if necessary. A putative thiopeptide BGC (Xszus_1.region006, Xsze_2.region003, Xsto_4.region001, Xpb_30.3_21.region001, Xmir_10.region001, Xmau_6.region001, Xkoz_3.region001, Xjap_NZ_FOVO01000011.region001, Xish_1.region003, Xhom_ANU1.region005, Xhom_2.region003, Xets_11.region001, XenKK7.region002, XenDL20_c00108_NODE_12.region001, Xekj_19.region001, Xehl_28.region001, Xe30TX1_c0031_NODE_38.region001, Xdo_HBLC131_1.region001, Xdo_FRM16.1.region005, Xbov_NC_013892.1.region004, Ptem_HBLC135_17.region001, Ppb6_4.region001, Plum_TT01_1.region008, Pthr_PT1.1_23.region001, Plau_IT4.1_12.region001, Plum_IL9_35_scf0001.region001, Pbod_HU2.3_20.region001, Plau_HB1.3_105.region001, Plum_EN01_24_scf0009.region001, Pbod_DE6.1_24.region001, Plau_DE2.2_108.region001, Phpb_1.region001, Pbod_LJ_007.region001, Pbod_CN4_25_scf0020.region001, Paeg_BKT4.5_19.region001, P_tem_1.region017 and so on) that exists throughout 45 *XP* genomes was excluded in the analysis, because it turned out that its annotation by antiSMASH 5.0 is a false positive and early reports suggest that this cluster is responsible for ribosomal methylthiolation^79,80^. Two BGCs, Xdo_HBLC131_4.region001 encoding the biosynthesis of glidobactins in *X. doucetiae* HBLC131 and Ptem_HBLC135_2.region002 encoding the biosynthesis of ririwpeptides in *P. temperata* HBLC135, were artificially integrated into their respective genome by CRAGE^39^ previously, and thus the two BGCs were also excluded in our analysis.

### Pangenome analysis

#### Biosynthetic gene cluster boundary definition

The cluster boundary was defined by antiSMASH with the start nucleotide of the first biosynthetic gene (5′ end) and the stop nucleotide of the last biosynthetic gene (3′ end), and was manually corrected if necessary. Non-structural genes (such as transporters, regulators, transposases and so on) on the outer periphery of an operon were excluded. We compiled a table with contigs of all BGCs encoded by a given genome, BGC start and stop nucleotide positions, BGC classifications by antiSMASH and BiG-SCAPE (see the BiG-SCAPE analysis section), and possible biosynthetic pathways that the BGCs encode (Source Data Fig. 1). These tables would be integrated into the contigs databases of the pangenome for filtering the biosynthetic genes and monitoring distributions of biosynthetic gene homology groups.

#### Interface generation

All genomes were obtained from the National Center for Biotechnology Information (NCBI). Supplementary Table 1 reports their accession numbers. The pangenome analysis herein mainly followed the anvi’o 6.1 pangenomic workflow^28,78^. After simplifying the header lines of 45 FASTA files for genomes using ‘anvi-script-reformat-fasta’, we converted FASTA files into anvi’o contigs databases by the ‘anvi-gen-contigs-database’ and then decorated the contigs database with hits from HMM models by ‘anvi-run-hmms’. The program ‘anvi-run-ncbi-cogs’ was run to annotate genes in the contigs databases with functions from the NCBI’s Clusters of Orthologous Groups (COGs). Tables of gene caller IDs with start and stop nucleotide positions were exported by ‘anvi-export-table’. By linking the gene caller IDs with BGCs via the start and stop nucleotide positions, genes that fell within a given BGC boundary were considered to be natural product biosynthetic genes (Source Data Fig. 1). Thereafter, the biosynthetic genes were furnished with a classification and a possible compound name, both of which were derived from the BGC that the biosynthetic genes made up. The obtained tables were imported back to contigs databases by ‘anvi-import-functions’. External genome storage was created by ‘anvi-gen-genomes-storage’ to store DNA and amino-acid sequences, as well as functional annotations of each gene. With the genome storage in hand, we used the program ‘anvi-pan-genome’ with the genomes storage database, the flag ‘--use-ncbi-blast’ and the parameter ‘--mcl-inflation 8’. The results were displayed in an interface by ‘anvi-display-pan’. The organization of the pangenome interface as shown in the dendrogram in the centre was represented by ‘presence/absence’ patterns. The core gene bin was characterized by searching the gene homology group (gene homology group represents amino-acid sequences from one or more genomes aligned by muscle^81^) using filters with ‘Min number of genomes gene homology group occurs, value = 45’. The singleton bin was identified by ‘Max number of genomes gene homology group occurs, value = 1’. The rest of the gene clusters that were neither sorted into the core gene bin nor the singleton bin were appended to the accessory bin. The single-copy-core-gene (scg) bin was found by ‘Min number of genomes gene homology group occurs, value = 45’ and ‘Max number of genes from each genome, value = 1’. The scg bin was refined by ‘Max functional homogeneity index 0.9’ and ‘Min geometric homogeneity index 1’. The resulting protein sequences were exported by ‘anvi-get-sequences-for-gene-clusters’ and aligned using ClustalW 1.2.2, which is incorporated in Geneious Prime 2021. Phylogenetic trees were generated using the Geneious tree builder utilizing the Jukes–Cantor distance model and the unweighted pair group method with arithmetic mean (UPGMA), and subsequently imported back to anvi’o by ‘anvi-import-misc-data’ and visualized by the interface. The statistical data of BGCs obtained from antiSMASH 5.0 (ref. ^23^) and BiG-SCAPE^42^ were imported to the layers of the interface by ‘anvi-import-misc-data’ for visualization.

#### Biosynthetic gene and biosynthetic gene cluster filtering

The bin summary (scg, core, accessory and singleton) with BGC classifications was exported by ‘anvi-summarize’ to monitor the distributions of the biosynthetic gene homology group in the pangenomes (Source Data Fig. 1 and Supplementary Data). In the Excel sheets, ‘core’ and ‘scg’ filters were selected from the ‘bin_name’ column, and the ‘(Blank)’ filter from the ‘BGC_classification’ column was unselected. The table was then sorted by ‘genome_name’ and ‘gene_callers_id’ columns in ascending order. This then displayed consecutive core biosynthetic genes that could possibly make up a BGC. The same procedure was used to filter BGCs in the accessory or singleton region.

### BiG-SCAPE analysis

BGCs in all genome sequences obtained from antiSMASH 5.0 (ref. ^23^) analyses were compared to reference BGCs from MIBiG repository 2.0 (refs. ^41,82^) using BiG-SCAPE 1.0.0 (ref. ^42^) with the PFAM database 32.0 (ref. ^83^). The analysis was conducted using default settings with the mode ‘auto’, mixing all classes and retaining singletons. Networks were computed for raw distance cutoffs of 0.30–0.95 in increments of 0.05. Results were visualized as a network using Cytoscape 3.7.2 (ref. ^84^) for a cutoff of 0.65 (Fig. 3 and Source Data Fig. 3). Statistical data for the BGCs were analysed and evaluated using Origin 2020b and Excel from Microsoft Office 365.

### Strain and culture conditions

Wild-type strains and the mutants thereof and *E. coli* (Supplementary Table 14) were cultivated on lysogeny broth (LB) agar plates at 30 °C overnight, and subsequently inoculated into liquid LB culture at 30 °C with shaking at 200 r.p.m. For compound production, the overnight LB culture was transferred into 5 ml of LB, XPP^19^ or Sf-900 II SFM medium (1:100, vol/vol) with 2% (vol/vol) Amberlite XAD-16 resins, 0.1% l-arabinose as the inducer for mutants with a P_*BAD*_ promoter, and selective antibiotics such as ampicillin (Am, 100 µg ml^−1^), kanamycin (Km, 50 µg ml^−1^) or chloramphenicol (Cm, 34 µg ml^−1^) at 30 °C, with shaking at 200 r.p.m.

### Culture extraction and HPLC-UV-MS analysis

The XAD-16 resins were collected after 72 h and extracted with 5 ml of methanol or ethyl acetate. The solvent was dried under rotary evaporators, and the dried extract was resuspended in 500 μl of methanol or acetonitrile/water (1:1 vol/vol for photoxenobactins), of which 5 μl was injected and analysed by HPLC-UV-MS or HPLC-UV-HRMS. Unless otherwise specified, HPLC-UV-MS and HPLC-UV-HRMS chromatograms in the figures are shown on the same scale. Bruker Compass DataAnalysis 4.3 was used for data collection and analysis of chromatography and MS. MetabolicDetec 2.1 was utilized to differentiate MS profiles between induced and non-induced promoter insertion mutants for identifying possible metabolites produced by targeted BGCs.

### Construction of P_*BAD*_ promoter insertion mutants

A 500–800-bp section upstream of the target gene (*lpcS*, *pxbF*, *rdb1A* and *xvbA*) was amplified with a corresponding primer pair as listed in Supplementary Table 15. The resulting fragments were cloned using Hot Fusion^85^ into a pCEP_kan or pCEP_cm backbone that was amplified by pCEP_Fw and pCEP_Rv. After transformation of a constructed plasmid into *E. coli* S17-1 *λ* pir, clones were verified by PCR with primers pCEP-Ve-Fw and pDS132-Ve-Rv. A wild-type strain (*X. bovienii* SS-2004, *X. szentirmaii* DSM 16338, *X. budapestensis* DSM 16342 or *X. vietnamensis* DSM 22392) or a deletion mutant (*X. szentirmaii ∆hfq*, *X. budapestensis ∆rdb1P* or *X. budapestensis ∆rdb1P ∆hfq*) was used as a recipient strain. The recipient strain was mated with *E*. *coli* S17-1 *λ* pir (donor) carrying a constructed plasmid (Supplementary Table 16). Both strains were grown in the LB medium to an optical density at 600 nm (OD_600_) of 0.6 to 0.7, and the cells were washed once with fresh LB medium. Subsequently, the donor and recipient strains were mixed on an LB agar plate in ratios of 1:3 and 3:1, and incubated at 37 °C for 3 h followed by incubation at 30 °C for 21 h. After that, the bacterial cell layer was collected with an inoculating loop and resuspended in 2 ml of fresh LB medium. A 200-μl sample of the resuspended culture was spread out on an LB agar plate with Am/Km or Am/Cm and incubated at 30 °C for two days. Individual insertion clones were cultivated and analysed by HPLC-UV-HRMS, and the genotype of all mutants was verified by plasmid- and genome-specific primers.

### Construction of deletion mutants

A ~1,000-bp upstream and a ~1,000-bp downstream fragment of *hfq* in *X. budapestensis* DSM 16342 were amplified using the primer pairs listed in Supplementary Table 15. The amplified fragments were fused using the complementary overhangs introduced by primers and cloned into the pEB17 vector that was linearized with PstI and BglII by Hot Fusion^85^. Transformation of *E. coli* S17-1 *λ* pir with the resulting plasmid (Supplementary Table 16) and conjugation with *X. budapestensis* DSM 16342, as well as the generation of double crossover mutants via counterselection on LB plates containing 6% sucrose, were carried out as previously described^86^. The deletion mutant was verified via PCR using the primer pairs listed in Supplementary Table 15, which yielded a ~2,000-bp fragment for mutants genetically equal to the WT strain and a ~1,000-bp fragment for the desired deletion mutant. The same procedure was used to generate *Δrdb1P* mutants, during which *E. coli* S17-1 *λ* pir carrying pEB17 *rdb1P* was mated with the *X. budapestensis* DSM 16342 wild-type and *X. budapestensis ∆hfq* mutant.

### Labelling experiments for structural elucidation of photoxenobactins C and D by MS

The cultivation of strains for labelling experiments was carried out as described above. For photoxenobactin C (**6**) labelling experiments, the overnight culture was transferred into LB medium additionally fed with 4-fluorosalicylate-SNAC, l-methionine-(methyl-*d*_3_), l-[U-^13^C,^15^N]cysteine and l-[U-^34^S]cysteine at a final concentration of 1 mM. In terms of inverse feeding experiments, cell pellets of the 100-μl overnight culture were washed once with ISOGRO ^13^C or ^13^C,^15^N medium (100 μl) and resuspended in the corresponding isotope labelling medium (100 μl). The feeding culture in the isotope labelling medium (5 ml) was inoculated with a washed overnight culture (50 μl) and additional l-cysteine was added at a final concentration of 1 mM.

For photoxenobactin D (**7**) labelling experiments, the cell pellets of the 100-μl overnight culture were washed once with ISOGRO ^13^C or ^15^N medium (100 μl) and then resuspended in the corresponding isotope labelling medium (100 μl). A 5-ml isotope labelling medium was inoculated with a washed overnight culture (50 μl).

### Isolation and purification

For photoxenobactin isolation, 10 ml of LB medium was inoculated with a colony of the *X. szentirmaii* P_*BAD*_ *pxbF ∆hfq* mutant from an LB agar plate and cultivated overnight. A 10-ml culture was taken to inoculate 2 × 100 ml of LB medium (OD_600_ ≈ 0.1). The 2 × 100-ml cultures were incubated overnight and the whole culture volume (200 ml) was used to inoculate a 20-l LB fermenter (Braun) supplemented with 2% XAD-16 and 0.2% arabinose (antifoam was added when required). Fermenter settings were as follows: 30 °C without pH control, three six-blade impellers 150 r.p.m. After 24 h, 10 l of the culture was collected from the fermenter, and the XAD resins were separated from the cells by filtration. (1) The XAD resins were extracted with 2 × 1 l of ethyl acetate with 1% formic acid, and the combined organic phase was dried under reduced pressure. (2) The culture without XAD was centrifuged and the supernatant was extracted with 3 × 5 l of ethyl acetate with 1% formic acid, and the combined organic layers were dried under reduced pressure. (3) The cell pellet was extracted with 2 × 1 l of ethyl acetate with 1% formic acid, and the organic supernatant was dried under reduced pressure. After 48 h, the remaining 10 l of bacterial culture were extracted as described in steps (1) to (3). The combined extracts from 20 l of culture were fractionated by a flash purification system with a C18 column with a gradient elution of acetonitrile/water 20–100% at 20 ml min^−1^ (every 10% gradient step was performed with five column volumes, except the 60–70% step, which was performed with ten column volumes). Fractions containing photoxenobactins were combined and dried under reduced pressure. Final purification was achieved via preparative and semipreparative HPLCs with a gradient of 30% acetonitrile/water (0–30 min) and 30–100% acetonitrile/water (30–40 min). The fractions were combined in brown flasks and were immediately freeze-dried to afford photoxenobactin A (**4**, 0.8 mg), photoxenobactin B (**5**, 0.6 mg), photoxenobactin C (**6**, 1.2 mg) and photoxenobactin E (**8**, 2.2 mg).

For the isolation and purification of lipocitides A and B, 2% of XAD-16 resins from a 6-l LB culture of the *X. bovienii* P_BAD_ *lpcS* mutant induced by l-arabinose were collected after 72 h of incubation at 30 °C with shaking at 120 r.p.m., and were washed with water and extracted with methanol (3 × 1 l) to yield a crude extract (5.3 g after evaporation). The extract was dissolved in methanol and was subjected to preparative HPLC with a C18 column using an acetonitrile/water gradient (0.1% formic acid) for 0–32 min, 55–80%, 40 ml min^−1^ to afford lipocitides A (**17**, 4.8 mg) and B (**18**, 9.0 mg).

Two percent of XAD-16 resins from a 12-l LB culture of the *X. budapestensis* P_*BAD*_ *rdb1A ∆rdb1P ∆hfq* mutant induced by l-arabinose were collected after 72 h of incubation at 30 °C with shaking at 120 r.p.m. and washed with water and extracted with methanol (3 × 2 l) to yield a crude extract (15.3 g after evaporation). The extract was subject to a Sephadex LH-20 column eluted with methanol. The fraction (2.8 g) containing pre-rhabdobranins was subjected to preparative HPLC with a C18 column using an acetonitrile/water gradient (0.1% formic acid) for 0–20 min, 15–35%, 40 ml min^−1^ to afford a fraction (206 mg) mainly containing pre-rhabdobranin D, which was further purified by semipreparative HPLC with a C18 column using an acetonitrile/water gradient (0.1% formic acid) for 0–24 min, 5–53%, 3 ml min^−1^ to afford pre-rhabdobranin D (**27**, 59.1 mg).

Benzobactin A (**28**) and its methyl ester (**29**), which were detected in *X. vietnamensis* P_*BAD*_ *xvbA*, were also produced by *Pseudomonas chlororaphis* subsp. *piscium* DSM 21509 (unpublished). Owing to the high production level in *Pseudomonas chlororaphis* subsp. *piscium* DSM 21509, **28** and **29** were isolated from the *Pseudomonas* strain. Four percent of XAD-16 resins from a 12-l XPP culture of *Pseudomonas chlororaphis* subsp. *piscium* DSM 21509 P_*BAD*_ *pbzA* mutant induced by l-arabinose were collected after 72 h of incubation at 30 °C with shaking at 120 r.p.m., and washed with water and extracted with methanol (3 × 2 l) to yield a crude extract (95.4 g after evaporation). The extract was dissolved in methanol and subjected to preparative HPLC with a C18 column using an acetonitrile/water gradient (0.1% formic acid) for 0–18 min, 5–59%, 20 ml min^−1^ to afford ten fractions. Fractions 2 (95.6 mg) and 3 (50.7 mg) were further purified by semipreparative HPLC with a C18 column using an acetonitrile/water gradient (0.1% formic acid) for 0–35 min, 5–95%, 3 ml min^−1^ to afford benzobactin A (**28**, 3.2 mg) and its methyl ester (**29**, 0.9 mg), respectively.

### NMR spectroscopy

Measurements were carried out using ^1^H and ^13^C NMR, ^1^H-^13^C heteronuclear single quantum coherence (HSQC), ^1^H-^13^C heteronuclear multiple bond correlation (HMBC), ^1^H-^1^H correlation spectroscopy (COSY), ^1^H-^13^C heteronuclear multiple quantum correlation/^1^H-^1^H correlation spectroscopy (HMQC-COSY) and ^1^H-^13^C heteronuclear single quantum coherence/^1^H-^1^H total correlation spectroscopy (HSQC-TOCSY). Chemical shifts (*δ*) were reported in parts per million (ppm) and referenced to the solvent signals. Data are reported as follows: chemical shift, multiplicity (br = broad, s = singlet, d = doublet, t = triplet, dd = doublet of doublet, m = multiplet and ov = overlapped) and coupling constants (in hertz). Bruker TopSpin 4.0 was used for NMR data collection and spectral interpretation.

### General synthetic procedures

The Fmoc protecting group was removed with 2 ml of 40% piperidine/dimethylformamide (DMF; 5 min) followed by 2 ml of 20% piperidine/DMF (10 min). Washings between coupling and deprotection steps were performed with DMF (five syringe volumes) and dichloromethane (DCM) (five syringe volumes). Resin loadings were determined by Fmoc cleavage from a weighted resin sample^87^. The combined filtrates containing Fmoc cleavage products were quantified spectrophotometrically at 301 nm using a UV–vis spectrophotometer with Hellma absorption cuvettes with a path length of 1 cm. Loadings were calculated (in mmol resin) using Lambert–Beer’s law with *ɛ* = 7,800 M^−1^ cm^−1^: loading (mmol) = ${\frac{{{\rm{Abs}}\,{({\rm{sample}})}}}{{\varepsilon l}}} \times V$, where *ɛ* is the molar extinction coefficient, *V* is the sample volume in liter and *l* is the optical path length in cm. Final cleavage was achieved by shaking the resin in 2 ml of a mixture of TFA/TIPS/H_2_O (95:2.5:2.5) for 1 h. The filtrate was then collected and the resin washed three times (2 ml each) with DCM, and the combined filtrates were dried under reduced pressure.

#### Syntheses of lipocitide A

Fmoc-protected Rink Amide resin (192 mg, 0.52 mmol g^−1^, 0.1 mmol) was placed in a polypropylene 6-ml syringe vessel fitted with polyethylene porous filter disks and swollen in 3 ml of DMF for 10 min. Subsequently, the Fmoc-protected resin was deprotected and then washed as described in the general synthetic procedures. Fmoc-d-Cit-OH (198.0 mg, 0.5 mmol, 5 equiv.), 1-hydroxy-7-azabenzotriazole (HOAT, 0.83 ml, 0.5 mmol, 5 equiv.), hexafluorophosphate azabenzotriazole tetramethyl uronium (HATU, 190.5 mg, 0.5 mmol, 5 equiv.) and N,N-diisopropylethylamine (DIPEA, 170 μl, 1.0 mmol, 10 equiv.) were dissolved in 1.5 ml of dry DMF. After 5 min, the clear solution was added to the resin and shaken at room temperature overnight. The resin was washed and loading was calculated (79.2%) as described in the general synthetic procedures. Acylation of Fmoc-l-Ala-OH (74.1 mg, 0.24 mmol, 3 equiv.), Fmoc-d-Leu-OH (84.8 mg, 0.24 mmol, 3 equiv.) and myristic acid (54.8 mg, 0.24 mmol, 3 equiv.) were carried out using the abovementioned procedure. Final cleavage was performed as described in the general synthetic procedures, and the crude product (70.8 mg) was purified by HPLC to obtain lipocitide A (**17**, Supplementary Fig. 100; 24.3 mg, 54.0%) as a white solid.

#### Syntheses of lipocitide B

2-CTC resin (63 mg, 1.6 mmol g^−1^, 0.1 mmol) was placed in a polypropylene 6-ml syringe vessel fitted with polyethylene porous filter disks. The resin was incubated with Fmoc-d-Cit-OH (119.0 mg, 0.3 mmol, 3 equiv.) and DIPEA (153 μl, 0.9 mmol, 9 equiv.) in 1.5 ml of dry DCM at room temperature overnight. The resin was washed and loading was calculated (56.7%) as described in the general synthetic procedures. Acylations of Fmoc-l-Ala-OH (52.9 mg, 0.17 mmol, 3 equiv.), Fmoc-d-Leu-OH (60.1 mg, 0.17 mmol, 3 equiv.) and myristic acid (38.9 mg, 0.24 mmol, 3 equiv.) were performed with additional HOAT (0.47 ml, 0.28 mmol, 5 equiv.), HATU (108 mg, 0.28 mmol, 5 equiv.) and DIPEA (96 μl, 0.56 mmol, 10 equiv.). Final cleavage was carried out as described in the general synthetic procedures, and the crude (54.2 mg) was purified by HPLC to obtain lipocitide B (**18**, Supplementary Fig. 101; 18.6 mg, 57.6%) as a white solid.

#### Synthesis of *S*-(2-acetamidoethyl)4-fluoro-2-hydroxybenzothioate (4-fluorosalicylate SNAC)

To a solution of 4-fluorosalicylic acid (156 mg, 1.0 mmol, 1.0 equiv.) and hydroxybenzotriazole (HOBt, 162 mg, 1.2 mmol, 1.2 equiv.) in 45 ml of THF, N,N′-dicyclohexylcarbodiimide (DCC, 248 mg, 1.2 mmol, 1.2 equiv.) was added, followed by *N*-acetylcysteamine (112 µl, 1.0 mmol, 1.0 equiv.). After 1 h at room temperature, K_2_CO_3_ (138 mg, 1.0 mmol, 1.2 equiv.) was added and the reaction was stirred for an additional 2 h. The reaction mixture was then filtered and concentrated by rotary evaporation. The solid residue was dissolved in ethyl acetate and washed with sat. NaHCO_3_ (50 ml) and water (50 ml). The organic layer was dried over MgSO_4_, concentrated, and purified by flash chromatography (1–10% MeOH in CHCl_3_) to give 26 mg (10%) *S*-(2-acetamidoethyl)4-fluoro-2-hydroxybenzothioate (Supplementary Fig. 102).

### IC_50_ value determination with the purified yeast 20S proteasome core particle

Yeast 20S proteasome core particle (yCP) from *Saccharomyces cerevisiae* was purified according to previously described methods^88,89^. The concentration of purified yCP was determined spectrophotometrically at 280 nm. yCP (final concentration: 0.05 mg ml^−1^ in 100 mM Tris-HCl, pH 7.5) was mixed with dimethyl sulfoxide (DMSO) as a control or serial dilutions of IOC (**1**) in DMSO, thereby not surpassing a final concentration of 10% (vol/vol) DMSO. After an incubation time of 45 min at room temperature, fluorogenic substrates Boc-Leu-Arg-Arg-AMC (AMC, 7-amino-4-methylcoumarin), Z-Leu-Leu-Glu-AMC and Suc-Leu-Leu-Val-Tyr-AMC (final concentration of 200 µM) were added to measure the residual activity of caspase-like (C-L, β1 subunit), trypsin-like (T-L, β2 subunit) and chymotrypsin-like (ChT-L, β5 subunit), respectively. The assay mixture was incubated for another 60 min at room temperature, then diluted 1:10 in 20 mM Tris-HCl, pH 7.5. The AMC molecules released by hydrolysis were measured in triplicate with a Varian Cary Eclipse fluorescence spectrophotometer (Agilent Technologies) at *λ*_exc_ = 360 nm and *λ*_em_ = 460 nm. Relative fluorescence units were normalized to the DMSO-treated control. The calculated residual activities were plotted against the logarithm of the applied inhibitor concentration and fitted with GraphPad Prism 9.0.2. IC_50_ values were deduced from the fitted data. These depend on enzyme concentration and are comparable within the same experimental settings.

### Crystallization and structure determination of the yCP in complex with IOC (1)

Crystals of the yCP were grown in hanging drops at 20 °C, as previously described^88,89^. The protein concentration used for crystallization was 40 mg ml^−1^ in Tris/HCl (20 mM, pH 7.5) and EDTA (1 mM). The drops contained 1 μl of protein and 1 μl of the reservoir solution (30 mM magnesium acetate, 100 mM 2-(*N*-morpholino)ethanesulfonic acid (pH 6.7) and 10% (wt/vol) 2-methyl-2,4-pentanediol). Crystals appeared after two days and were incubated with **1** at a final concentration of 10 mM for at least 24 h. Droplets were then complemented with a cryoprotecting buffer (30% (wt/vol) 2-methyl-2,4-pentanediol, 15 mM magnesium acetate, 100 mM 2-(*N*-morpholino)ethanesulfonic acid, pH 6.9) and vitrified in liquid nitrogen. The dataset from the yCP:IOC complex was collected using synchrotron radiation (*λ* = 1.0 Å) at the X06SA-beamline (Swiss Light Source). X-ray intensities and data reduction were evaluated using the XDS program package version 5 February 2021 (Supplementary Table 17)^90^. Conventional crystallographic rigid body, positional and temperature factor refinements were carried out with REFMAC5 5.0.32 (ref. ^91^) and the CCP4 Program Suite 7.1.016 (ref. ^92^) using coordinates of the yCP structure as the starting model (PDB 5CZ4)^50^. Model building was performed by the programs SYBYL-X and COOT 0.8.7 (ref. ^93^). The final coordinates yielded excellent residual factors, as well as geometric bond and angle values. Coordinates were confirmed to fulfil the Ramachandran plot and have been deposited in the RCSB (PDB 7O2L).

### Haemocyte-spreading assays

*Spodoptera exigua* larvae were collected from Welsh onion (*Allium fistulsum* L.) fields in Andong, Korea. Insects were reared in the laboratory under the following conditions: 25 ± 2 °C constant temperature, 16:8 h (light/dark) photoperiod and 60 ± 5% relative humidity. Larvae were reared on an artificial diet^94^ and 10% sucrose solutions were fed to adult insects. Fifth instar larvae were used in all experiments. For analysing haemocyte behaviours in vivo, fifth instar larvae of *S. exigua* were co-injected with 1 µl of heat-killed (95 °C for 10 min) *E. coli* TOP10 (2.4 × 10^4^ cells per larva) with the test compound (0–1,000 ng per larva) by using a Hamilton microsyringe (Reno). At 1 h post-injection, 10 µl of haemolymph from each larva was collected on the glass slide and incubated for 5 min inside a dark wet chamber at room temperature. The medium was replaced with 3.7% of formaldehyde dissolved in phosphate buffered saline (PBS) and incubated for 10 min. After washing three times with PBS, cells were permeabilized with 0.2% Triton X-100 in PBS for 2 min at room temperature. After incubation, the slides were washed with PBS three times. Blocking was performed using 5% skimmed milk (Invitrogen) dissolved in PBS, followed by incubation for 10 min. After washing once with PBS, the cells were incubated with fluorescein isothiocyanate (FITC)-tagged phalloidin in PBS for 1 h at room temperature. After washing three times, the cells were incubated with 4′,6-diamidino-2-phenylindole (DAPI, 1 mg ml^−1^, Thermo Scientific) in PBS for nucleus staining. Finally, after washing twice in PBS, cells were observed under a fluorescence microscope (DM2500, Leica) at ×400 magnification. Haemocyte spreading was determined by the extension of F-actin out of the original cell boundary. For the in vitro assay, ~100 μl of haemolymph was collected into 400 μl of anticoagulation buffer (ACB; 186 mM NaCl, 17 mM Na_2_EDTA, 41 mM citric acid, pH 4.5). After adding ACB, the medium was incubated for 30 min on ice. After centrifugation at 300*g* for 5 min, 400 μl of supernatant was discarded. The rest of the suspension was gently mixed with 200 μl of TC100 insect tissue culture medium (Welgene). From this suspension, 10 µl of haemolymph was collected on the glass slide. The slides were co-injected with 1 µl of *E. coli* TOP10 (2.4 × 10^4^ cells per larva) with the test compound (0–1,000 ng per larva), followed by the procedure described above. Means were compared by a least squared difference (LSD) test of one-way analysis of variance (ANOVA) using POC GLM of the SAS program (SAS Institute, 1989) and discriminated at type I error = 0.05.

### Nodulation assays

*E. coli* TOP10 was heat-killed by incubating at 95 °C for 10 min. Fifth-instar larvae of *S. exigua* were injected with 1 µl of bacteria (2.4 × 10^4^ cells per larva) using a Hamilton microsyringe along with 1 µl of different concentrations (10, 50, 100, 500 and 1,000 ppm) of inhibitors. Control larvae were injected with bacteria and DMSO. At 8 h after bacterial injection, nodules were counted by dissecting larvae under a stereomicroscope (Stemi SV 11, Zeiss) at ×50 magnification.

### Phenoloxidase activity assays

The PO activity from plasma was estimated as previously described^95^. Briefly, DOPA (l-3,4-dihydroxyphenylalanine) was used as a substrate for determining PO activity from treated larvae plasma. For PO activation, each fifth-instar larva of *S. exigua* was challenged with 2.4 × 10^4^ cells of heat-killed *E. coli* TOP10. Different inhibitors were co-injected (1 µg per larva) along with *E. coli* TOP10. After 8 h of bacterial challenge, haemolymph was collected from treated larvae in a 1.5-ml tube containing a few granules of phenylthiocarbamide (Sigma-Aldrich) to prevent melanization. Haemocytes were separated from plasma by centrifuging at 4 °C for 5 min at 300*g*. A reaction volume of 200 µl consisted of 180 µl of 10 mM DOPA in PBS (pH 7.4) and 20 µl of plasma. Absorbance was measured using a VICTOR multi-label plate reader (PerkinElmer) at 490 nm. PO activity was expressed as ΔABS per min per µl of plasma. Each treatment was replicated three times with independent samples.

### Measurement of nitric oxide

The NO was indirectly quantified by measuring its oxidized form, nitrate (NO_3_^−^), using the Griess reagent of a Nitrate/Nitrite Colorimetric Assay Kit (Cayman Chemical). Fifth-instar larvae were injected with 1 µl of heat-killed *E. coli* TOP10 (2.4 × 10^4^ cells per larva) using a Hamilton microsyringe along with 1 µl of the test compound. Haemolymph was collected from each sample 1 h post infection. A 150-μl volume of haemolymph from three L5 larvae was collected and homogenized in 350 μl of 100 mM PBS pH 7.4 with a homogenizer (Ultra-Turrax T8, Ika Laboratory). After centrifugation at 14,000*g* for 20 min at 4 °C, the supernatant was used to measure the nitrate amounts, and the total protein was measured in each sample by a Bradford assay. The samples were analysed in a 200-μl final reaction volume. Briefly, 80 μl of samples were added to the wells, then 10 μl of enzyme cofactor mixture and 10 μl of nitrate reductase mixture were added. After incubation at room temperature for 1 h, 50 μl of Griess reagent R1 and immediately 50 μl of Griess reagent R2 were added to each well. The plate was left at room temperature for 10 min for colour development. For a standard curve to quantify the nitrate concentrations of the samples, nitrates with final concentrations of 0, 5, 10, 15, 20, 25, 30 and 35 μM in a 200-μl reaction volume were used. The absorbance was recorded at 540 nm on a VICTOR multi-label plate reader. Our measurements used three larvae per sample, and we repeated the treatment with three biological samples.

### Galleria injection assays

Precultures of *X. szentirmaii* DSM wild-type strain and the mutants thereof were grown in LB medium and inoculated into fresh cultures at an OD_600_ of 0.1. Cells were grown to exponential phase (OD_600_ ≈ 1) and then diluted to an OD_600_ of 0.00025. A 5-µl volume of the diluted bacterial culture was injected into the last left pro-leg of the larvae (LB medium as a negative control). *G. mellonella* larvae were kept at 4 °C for 10 min before injection. After infection, the larvae were incubated at 25 °C. Dead *Galleria* larvae were frozen at −20 °C, then at −80 °C, and freeze-dried for one day. Freeze-dried larvae were ground. Every injection experiment was aliquoted into two portions, one of which was extracted with 25 ml of acetone/ethyl acetate (vol/vol, 1:1) while the other one was extracted with acetone/methanol. Extracts were dried and resuspended in 3 ml of acetonitrile/water (1:1, vol/vol) with a tenfold dilution for HPLC-MS-UV analysis. To compare the survival percentage of *G. mellonella* larvae infected with the WT strain and mutants and to determine median lethal time (LT_50_) values, Kaplan–Meier curves were generated by GraphPad PRISM 8.4.3.

### Cytotoxicity assays

HepG2 cells (hepatoblastoma cell line; ACC 180, DSMZ) were cultured under conditions recommended by the depositor, and cells were propagated in Dulbecco’s modified Eagle medium supplemented with 10% fetal bovine serum. To determine the cytotoxicity of test compounds, cells were seeded at 6 × 10^3^ cells per well of 96-well plates in 120 μl of complete medium. After 2 h of equilibration, compounds were added in serial dilution in 60 µl of complete medium. Compounds as well as the solvent control and doxorubicin as an in-assay positive control (IC_50_ of 0.06 ± 0.01 µg ml^−1^) were tested as duplicates in two independent experiments. After 5 days of incubation, 20 μl of 5 mg ml^−1^ MTT (thiazolyl blue tetrazolium bromide) in PBS was added per well, and the cells were further incubated for 2 h at 37 °C. The medium was then discarded and cells were washed with 100 μl of PBS before adding 100 μl of 2-propanol/10 N HCl (250:1) to dissolve the formazan granules. The absorbance at 570 nm was measured using a microplate reader (Tecan Infinite M200Pro with Tecan iControl 2.0), and cell viability was expressed as a percentage relative to the respective solvent control. IC_50_ values were determined by sigmoidal curve fitting using GraphPad PRISM 8.4.3.

### Statistical analysis

In Fig. 5d,e,g,j,k, means were compared using an LSD test of one-way ANOVA using POC GLM of the SAS program (SAS Institute, 1989) for continuous variables and discriminated at type I error = 0.05. The results were plotted using Sigma Plot 12.0.

### Reporting Summary

Further information on research design is available in the Nature Research Reporting Summary linked to this Article.

## Online content

Any methods, additional references, Nature Research reporting summaries, source data, extended data, supplementary information, acknowledgements, peer review information; details of author contributions and competing interests; and statements of data and code availability are available at 10.1038/s41557-022-00923-2.

## Supplementary information

Supplementary Information Supplementary Discussion, Tables 6, 9, 11–13 and 17 and Figs. 1–102.

Reporting Summary

Supplementary Tables Supplementary Table 1 Wild-type strains used in bioinformatics analysis. Supplementary Table 2 BGCs annotations of 45 *XP* genomes by antiSMASH and in-house database. Supplementary Table 3 GCF analysis and exploration of *XP* BGCs by BiG-FAM. Supplementary Table 4 Putative functional assignments of biosynthetic genes in this study. Supplementary Table 5 HR-ESI-MS data of all compounds described in this work. Supplementary Table 6 ^1^H and ^13^C NMR data assigments for photoxenobactins A–C (4–6) and E (8) in DMSO-*d*_6_ (for NMR spectra and HRMS see Supplementary Figs. 31–52). Supplementary Table 7 IC_50_ values of GameXPeptide A (16) and lipocitides A (17) and B (18) against haemocyte-spreading behaviour. Supplementary Table 8 IC_50_ values of GameXPeptide A (16) and lipocitide B (18) against nodule formation. Supplementary Table 9 ^1^H (500 MHz) and ^13^C (125 MHz) NMR data assigments for lipocitides A (17) and B (18) in DMSO-*d*_6_ (for NMR spectra and HRMS see Supplementary Figs. 55–66). Supplementary Table 10 IC_50_ values of lipocitides A (17) and B (18) against nitric oxide production. Supplementary Table 11 ^1^H (500 MHz) and ^13^C (125 MHz) NMR data assignments for N-(ω-7-myristol)-d-asparagine (1) in DMSO-*d*_6_ (for NMR spectra and HRMS see Supplementary Figs. 67–72). Supplementary Table 12 ^1^H (700 MHz) and ^13^C (175 MHz) NMR data assignments for pre-rhabdobranin D (27) in DMSO-*d*_6_ (for NMR spectra and HRMS see Supplementary Figs. 80–87). Supplementary Table 13 ^1^H (500 MHz) and ^13^C (125 MHz) NMR data assignments for benzobactin A (28) and its methyl ester (29) in DMSO-*d*_6_ (for NMR spectra and HRMS see Supplementary Figs. 88–99). Supplementary Table 14 Strains used in this study. Supplementary Table 15 Primers used in this study. Supplementary Table 16 Plasmids used in this study.

Supplementary Data 1 Supplementary Data Fig. 5a Pangenome analysis of 29 *Xenorhabdus* genomes by anvi’o.

Supplementary Data 2 Supplementary Data Fig. 5b Pangenome analysis of 16 *Photorhabdus* genomes by anvi’o.

Supplementary Data 3 Supplementary Data Fig. 17 Transcriptional analysis of biosynthetic genes in the conserved BGCs (*ioc*/*leu*, *gxp*, *pxb*, *lpcS*, *fcl* and *ape*) in *X. szentirmaii* US.

## Data Availability

The genome sequence data that support the findings of this study are available in NCBI GenBank database under accession nos. AYSJ00000000, CP011104.1, CP016176.1, FO704550, FOVO01000000, JADEUF000000000, JAGJDU000000000, JAGJJP000000000, JAGJJQ000000000, JAGJJR000000000, JAGJJS000000000, JAGJJT000000000, JAGJJU000000000, JAGJJV000000000, JAGJJW000000000, LOIC00000000, LOMY00000000, MKGQ00000000, MKGR00000000, MUBJ00000000, MUBK00000000, NC_005126.1, NC_013892.1, NC_014228.1, NIBS00000000, NIBT00000000, NIBU00000000, NIBV00000000, NITY00000000, NITZ00000000, NIUA00000000, NJAH00000000, NJAI00000000, NJAJ00000000, NJAK00000000, NJCW00000000, NJCX00000000, NJGH00000000, NKHP00000000, NKHQ00000000, NSCM00000000, VNHN00000000, WSEY00000000, WSFA00000000 and WSFB00000000. For the corresponding genomes, see Supplementary Table 1. Crystallographic data have been deposited in the Protein Data Bank (https://www.rcsb.org) under PDB 7O2L. All other data generated or analysed in this study are available within the Article and its Supplementary Information and Source Data. Source data are provided with this paper.

## Source data

Source Data Fig. 1 Pangenome analysis of 45 *Xenorhabdus* and *Photorhabdus* genomes by anvi’o.
Source Data Fig. 2 Transcriptional analysis of biosynthetic genes in the conserved BGCs (*ioc/leu, gxp, pxb, stl/bkd, plu3123, glb, plu0082–0077* and *plu4334–4343*) in *P. luminescens* subsp. *laumondii* TT01. Translational analysis of biosynthetic genes in the conserved BGCs (*ioc/leu, gxp, pxb, stl/bkd, plu3123, glb, plu0082–0077* and *plu4334–4343*) in *P. luminescens* subsp. *laumondii* TT01.
Source Data Fig. 3 Domain sequence network comparison between *Xenorhabdus/Photorhabdus* BGCs and MIBiG 2.0 by BiG-SCAPE with a raw distance cutoff of 0.65.
