# Supplementary material for: Global analysis of biosynthetic gene clusters reveals conserved and unique natural products in entomopathogenic nematode-symbiotic bacteria
Source: Nat Chem. 2022 Apr 25;14(6):701–12. doi: 10.1038/s41557-022-00923-2 (PMC9177418; doi:10.1038/s41557-022-00923-2)
Supplement: Supplementary file 1 — Supplementary Discussion, Tables 6, 9, 11–13 and 17 and Figs. 1–102. [file 41557_2022_923_MOESM1_ESM.pdf]

nature portfolio

https://doi.org/10.1038/s41557-022-00923-2

Supplementary information

Global analysis of biosynthetic gene clusters reveals conserved and unique natural products in entomopathogenic nematode-symbiotic bacteria

In the format provided by the authors and unedited

Supplementary Information

Global analysis of biosynthetic gene clusters reveals conserved and unique natural products in entomopathogenic nematode-symbiotic bacteria

Yi-Ming Shi[1,2*], Merle Hirschmann[2], Yan-Ni Shi[1,2], Shabbir Ahmed[3], Desalegne Abebew[2], Nicholas J. Tobias[4,5], Peter Grün[2], Jan J. Crames[1,2], Laura Pöschel[2], Wolfgang Kuttenlochner[6], Christian Richter[7], Jennifer Herrmann[8], Rolf Müller[8], Aunchalee Thanwisai[9], Sacha J. Pidot[10], Timothy P. Stinear[10], Michael Groll[6], Yonggyun Kim[3], Helge B. Bode[1,2,4,5*]

[1]Department of Natural Products in Organismic Interactions, Max-Planck Institute for Terrestrial Microbiology, 35043 Marburg, Germany

[2]Molecular Biotechnology, Department of Biosciences, Goethe University Frankfurt, 60438 Frankfurt am Main, Germany

[3]Department of Plant Medicals, College of Life Sciences, Andong National University, 36729 Andong, Korea

[4]LOEWE Center for Translational Biodiversity Genomics (TBG), 60325 Frankfurt, Germany

[5]Senckenberg Gesellschaft für Naturforschung, 60325 Frankfurt, Germany

[6]Center for Protein Assemblies, Department of Chemistry, Technical University of Munich, 85748 Garching, Germany

[7]Institute for Organic Chemistry and Chemical Biology, Center for Biomolecular Magnetic Resonance, Goethe University Frankfurt, 60438 Frankfurt am Main, Germany

[8]Helmholtz Institute for Pharmaceutical Research Saarland (HIPS), Helmholtz Center for Infection Research & German Center for Infection Research (DZIF), partner site Hannover-Braunschweig, 66123 Saarbrücken, Germany

[9]Department of Microbiology and Parasitology, Faculty of Medical Science, Naresuan University, 65000 Phitsanulok, Thailand

[10]Department of Microbiology and Immunology, Peter Doherty Institute for Infection and Immunity, University of Melbourne, 3010 Melbourne, Australia

*Corresponding author email: yi-ming.shi@mpi-marburg.mpg.de; helge.bode@mpi-marburg.mpg.de

Extended Discussion

To date, the biosynthetic gene clusters (BGCs) of entomopathogenic bacteria *Xenorhabdus* and *Photorhabdus* (*XP)* have not been systematically analyzed. We examined the genomes of 45 *XP* strains covering almost all currently found *XP* taxonomy that are accessible in our strain collection, via pangenome[1] and domain sequence similarity networks[2]. The pangenomic analysis allows us to visualize the distribution of biosynthetic genes (BGs) in the core, accessory, and singleton regions of the pangenomes. By tracking the frequencies of occurrence for consecutive BGs in the core and accessory regions, we were able to rapidly refine the most highly conserved BGCs among 1,000 BGC entries. We discovered that 11 BGCs, belonging to the classes of NRPSs, PKS/NRPS hybrids, RiPPs, terpenes, and others, represent the most ubiquitous gene cluster families (GCFs) across *XP* or in one of the two genera (**Fig. 2**). Among them, four GCFs, namely *ioc/leu*, *pxb*, *lpc*, and *plu0082–0077* were previously unidentified. A domain sequence similarity network was used to assess the full biosynthetic capacity of *XP*, which enables us to extensively map relationships between the *XP* BGCs and MIBiG references and thereby pinpoints BGCs that have great potential for producing novel natural products. This leads to the discovery of 535 unknown BGCs, 30% of which are thought to be unique. With the global BGC map in hand, a promoter exchange strategy in the wild-type strain or Δ*hfq* mutant for homologously overexpressing BGCs of interest is then applied to efficiently translate BGCs into truly natural products for isolation, structural elucidation, and functional characterization.

Besides iron scavenging, yersiniabactin forms a complex with Cu^{II}, which serves as a superoxide dismutase mimic involved in the protection against the oxidative burst in phagocytes[3]. Due to the high structural similarity between piscibactin (**3**)/photoxenobactin D (**7**) and yersiniabactin, as well as the cupric chelating property of **3** and **7**, it is tempting to speculate that **3** and **7** scavenge environmental copper, forming cupric complexes to protect the bacteria from reactive oxygen species generated by the insect immune system.

Structurally related natural products might be functionally similar and thus possibly fulfill functional complementation in strains that lack one of the metabolites[4], a hypothesis that could be supported by the discovery of the widespread genus-specific GCFs (e.g. arylpolyene and carotenoid). The pigmented arylpolyene lipids with two large conjugated systems protect its producing strain against reactive oxygen species[5,6]. While the *ape* BGCs encoding their biosynthesis are supposed to be the most ubiquitous GCF in Gram-negative bacteria[6,7], these clusters are exclusively present in *Xenorhabdus* but absent from *Photorhabdus*. The *plu4334–4343* BGC as the most widely distributed *Photorhabdus*-specific (*P*-specific) terpene GCF is supposed to produce carotenoids that could conceivably fulfill the role of antioxidative protection[8] for *Photorhabdus*.

A recent survey of a correlation between taxonomic distance in myxobacteria and natural product novelty points out that the probability of novel natural product discovery could be increased by

exploring genera located in taxonomically distant clades[9]. This strategy could be extensively applied in free-living microorganisms. However, it is less likely to succeed in *XP* strains, because of their narrow ecological niche, in which bacteria are obligate mutualistic associations with specific soil nematodes, as well as limited taxonomic strains—only 26 *Xenorhabdus* and 19 *Photorhabdus* taxa have been identified[10]. Nonetheless, inspired by the discovery of structurally unique photoxenobactins (**4**–**8**), pre-rhabdobranins (**24**–**27**), and benzobactins (**28** and **29**), genome mining for novel natural products from *XP* might still be possible. A rarefaction analysis showed that the number of GCFs would increase upon the addition of every new genome, suggesting that sequencing of more *XP* isolates will probably reveal further previously unseen GCFs (**Supplementary Fig. 24**). This implies that the acquisition event of BGCs by horizontal gene transfer constantly occurs in *XP*, despite their relatively narrow niche. Distributions of GCFs at the genus level, as well as matrices comparing GCFs and BGCs to species, were then created to infer the differences within *XP* (**Supplementary Figs. 25** and **26**). While *Xenorhabdus* and *Photorhabdus* genera have 33 out of 176 common GCFs, a striking GCF diversity exists in *Xenorhabdus* even though *Xenorhabdus* has a tendency for fewer BGCs per genome than *Photorhabdus* (**Supplementary Fig. 2**). *Xenorhabdus*-specific (*X*-specific) GCFs per species are 1.5 times as many as that of *P*-specific. Together with the higher number of unique *X*-specific GCFs on average, which is in line with the more discrete GCF and BGC distribution pattern, these could point towards *Xenorhabdus* being more likely to encode novel BGCs compared to *Photorhabdus*. Additionally, despite limited taxonomic species, BGC explorations within-species variation still holds great promise. For example, *Xenorhabdus stockiae* taxonomically related strains, *Xenorhabdus* sp. KK7.4, *Xenorhabdus* sp. KJ12.1, *Xenorhabdus* sp. PB30.3, and *Xenorhabdus* sp. PB61.4, over-representing across Thailand[11] harbor seven unique genus-specific GCFs, including four isolated clade GCFs and three singletons (**Supplementary Fig. 27**).

Although these findings significantly expand our knowledge of the *XP* natural products, the strategy of genome-based natural product discovery for functional assignment has three limitations. First, the strategy relies on high-quality sequenced genomes, ideally with complete assembly. Some genomes we analyze here are not fully assembled with several pieces of fragmented BGCs across contigs, most of which appear to be part of an unknown mega NRPS BGC. Although the existence of fragmented NRPS BGCs would not change the tendency of NRPSs in the statistics, a clearer overview could be obtained by closing the assembly gaps. Second, our BGC annotations only rely on antiSMASH algorithms[12], and this may have a strong bias towards those that have been annotated. We realized that, for example, three widespread known BGCs encoding the biosyntheses of rhabduscin[13], leupeptins[14], and anthraquinones[15] were not picked up by antiSMASH 5.0. Also, with respect to RiPP BGCs, it is still impossible to achieve an extensive annotation by a general bioinformatics tool, while it requires implementation of multiple algorithms[16], as well as mining for specific tailoring enzymes[17]. Third, compound formation in a spontaneous

manner with low or even without the involvement of BGCs. There is increasing evidence that complex molecules with an isolable amount from culture media, such as rhabdoplanin[18], carbocyclinone-534 (ref[19]), and duotap-520 (ref[19]), are spontaneously formed through highly reactive building blocks that are ubiquitous in *Xenorhabdus* and/or *Photorhabdus* (e.g. rhabduscin[13] and isopropylstilbene[19]). Therefore, an attempt to pair genomics and metabolomics[20,21] will be very promising to address the shortcomings of an individual approach.

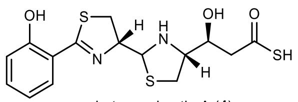

photoxenobactin A (**4**)

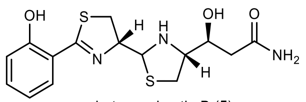

photoxenobactin B (**5**)

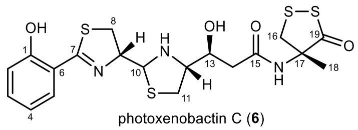

photoxenobactin C (**6**)

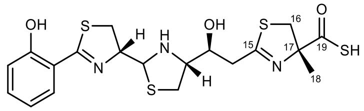

photoxenobactin E (**8**)

Supplementary Table 6 | ^{1}H and ^{13}C NMR data assignments for photoxenobactins A–C (**4**–**6**) and E (**8**) in DMSO-d_6 (for NMR spectra and HRMS see Supplementary Figs. 31–52).

	No.	Photoxenobactin A (**4**)		Photoxenobactin B (**5**)		Photoxenobactin C (**6**)		Photoxenobactin E (**8**)	
		δ_H (mult., *J*)[a]	δ_C, mult.[f]	δ_H (mult., *J*)[b]	δ_C, mult.[f]	δ_H (mult., *J*)[c]	δ_C, mult.[f]	δ_H (mult., *J*)[d]	δ_C, mult.[e]
SA									
	1	-	158.9, C	-	158.9, C	-	nd	-	172.4, C
	2	6.99 (br d, 7.7)	117.1, CH	6.98 (dd, 7.3, 1.2)	117.2, CH	6.97 (ov)	119.7, CH	6.50 (d, 8.5)	124.4, CH
	3	7.44 (ov)	134.1, CH	7.44 (ov)	133.8, CH	7.44 (ov)	130.7, CH	7.09 (td, 8.5, 1.8)	133.9, CH
	4	6.97 (td, 7.7, 1.1)	119.5, CH	6.95 (dd, 7.3, 1.2)	119.6, CH	6.97 (ov)	117.4, CH	6.32 (br t, 7.3)	112.1, CH
	5	7.46 (br d 7.7)	130.7, CH	7.42 (ov)	130.8, CH	7.44 (ov)	134.0, CH	7.16 (dd, 7.9, 1.8)	132.3, CH
	6	-	115.9, C	-	116.5, C	-	116.1, C	-	116.7, C
	7	-	173.8, C	-	171.8, C	-	173.3, C	-	172.1, C
L-Cys-1									
	8	3.61 (dd, 11.5, 9.1)	33.7, CH_2	3.39 (ov)	31.1, CH_2	3.52 (ov)	34.6, CH_2	3.45 (ov)	33.7, CH_2
		3.34 (ov)		3.29 (ov)		3.52 (ov)		3.09 (ov)	
	9	5.41 (ddd, 9.1, 7.5, 5.6)	78.3, CH	6.36 (td, 8.8, 3.6)	76.0, CH	4.94 (m)	81.5, CH	4.40 (ddd, 13.1, 10.3, 7.5)	78.7, CH
	10	5.65 (br d, 5.6)	64.3, CH	5.36 (br d, 3.6)	61.7, CH	5.35 (d, 8.3)	64.5, CH	4.71 (dd, 10.3, 6.8)	70.6, CH
	10NH	nd	-	nd	-	nd	-	5.31 (dd, 10.3, 6.9)	-
L-Cys-2									
	11	3.27 (ov)	27.2, CH_2	3.01 (t, 10.4)	26.3, CH_2	2.97 (dd, 10.4, 8.7)	30.8, CH_2	3.45 (dd, 12.3, 7.6)	37.5, CH_2
		3.02 (dd, 10.3, 7.8)		2.65 (ov)		2.90 (dd, 10.4, 6.6)		3.03 (br t, 11.4)	
	12	4.70 (td, 7.8, 4.5)	74.9, CH	4.35 (ddd, 10.4, 5.5, 3.5)	75.1, CH	4.23 (m)	68.9, CH	3.72 (dd, 18.1, 10.3)	66.3, CH
	13	4.43 (t, 4.9)	67.2, CH	4.09 (t, 3.9)	65.5, CH	4.46 (m)	66.8, CH	3.99 (br s)	67.8, CH
	13OH	nd	-	nd	-	nd	-	7.52 (s)	-
Polyketide									
	14	3.47 (ov)	57.8, CH_2	3.08 (dd, 16.5, 4.5)	48.0, CH_2	2.79 (16.4, 6.6)	39.3, CH_2	3.08 (ov)	40.0, CH_2
		3.94 (d, 17.5)		2.60 (ov)		2.47 (ov)		2.88 (d, 17.0)	
	15	-	198.4, C	-	162.6, C	-	162.8, C	-	170.6, C
L-Cys-3									
	16	-	-	-	-	3.46 (ov)	45.7, CH_2	3.59 (d, 11.5)	39.4, CH_2
						3.37 (ov)		3.15 (d, 11.5)	
	17	-	-	-	-	-	68.4, C	-	93.1, C
	18	-	-	-	-	1.42 (s)	22.8, CH_3	1.56 (s)	25.6, CH_3
	19	-	-	-	-	-	208.3, C	-	212.1, C

Data were recorded at [a]700, [b]500, [c]600, [d]800, and [e]200 MHz, respectively. [f]Data were extracted from HSQC and HMBC spectra. nd = not detectable. The stereochemistry was predicted by analyzing the *pxb* BGC and comparison of chemical shifts with piscibactins[22] and yersiniabactin[23,24].

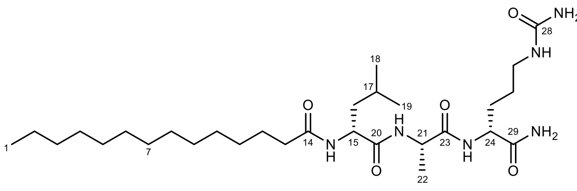

lipocitide A (**17**)

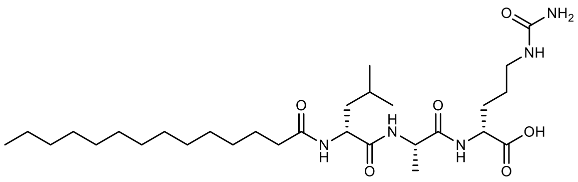

lipocitide B (**18**)

Supplementary Table 9 | ^{1}H (500 MHz) and ^{13}C (125 MHz) NMR data assignments for lipocitides A (**17**) and B (**18**) in DMSO-d_6 (for NMR spectra and HRMS see Supplementary Figs. 55–66).

	No.	Lipocitide A (**17**)		Lipocitide B (**18**)	
		δ_H (mult., J)	δ_C, mult.	δ_H (mult., J)	δ_C, mult.
FA					
	1	0.85(ov)	14.4, CH_3	0.85 (ov)	14.4, CH_3
	2	1.24 (ov)	22.6, CH_2	1.24 (ov)	22.6, CH_2
	3	1.24 (ov)	31.8, CH_2	1.24 (ov)	31.8, CH_2
	4	1.24 (ov)	29.2, CH_2	1.24 (ov)	29.2, CH_2
	5	1.24 (ov)	29.5, CH_2	1.24 (ov)	29.5, CH_2
	6	1.24 (ov)	29.5, CH_2	1.24 (ov)	29.5, CH_2
	7	1.24 (ov)	29.5, CH_2	1.24 (ov)	29.5, CH_2
	8	1.24 (ov)	29.5, CH_2	1.24 (ov)	29.5, CH_2
	9	1.24 (ov)	29.5, CH_2	1.24 (ov)	29.5, CH_2
	10	1.24 (ov)	29.2, CH_2	1.24 (ov)	29.2, CH_2
	11	1.28 (ov)	29.0, CH_2	1.28(m)	29.0, CH_2
	12	1.48 (ov)	25.7, CH_2	1.56 (ov)	25.7, CH_2
	13	2.11 (m)	35.5, CH_2	2.10 (m)	35.6, CH_2
	14	-	173.1, C	-	173.0, C
D-Leu					
	15NH	8.00 (t, 7.9)	-	8.06 (br s)	-
	15	4.24 (ov)	51.9, CH	4.26 (ov)	51.8, CH
	16	1.48 (ov)	40.9, CH_2	1.41 (m)	40.9, CH_2
	17	1.48 (ov)	24.7, CH	1.56 (ov)	24.7, CH
	18	0.84 (d, 6.4)	23.4, CH_3	0.83 (d, 6.6)	23.4, CH_3
	19	0.89 (d, 6.4)	22.0 CH_3	0.88 (d, 6.6)	22.0, CH_3
	20	-	172.6, C	-	172.5, C
L-Ala					
	21NH	8.13 (t, 6.9)	-	8.18 (br s)	-
	21	4.24 (ov)	48.7, CH	4.26 (ov)	48.6, CH
	22	1.20 (d, 7.1)	18.6, CH_3	1.20 (d, 7.1)	19.0, CH_3
	23	-	172.4, C	-	172.3, C
D-Cit					
	24NH	7.90 (dd, 8.0, 5.7)	-	7.90 (br d, 8.2)	-
	24	4.14(m)	52.6, CH	4.12 (m)	52.5, CH
	25a	1.68 (m)	29.7, CH_2	1.71 (m)	29.5, CH_2
	25b	1.58 (m)		1.56 (ov)	
	26	1.30 (ov)	27.1, CH_2	1.33 (m)	26.9, CH_2
	27NH	5.92 (t, 5.2)	-	5.91 (t, 5.0)	-
	27	2.91 (m)	39.1, CH_2	2.91 (dd, 12.4, 6.2)	39.3, CH_2
	28	-	159.2, C	-	159.2, C
	28NH_2	5.37 (s)<br>5.36 (s)	-	5.36 (s)	-
	29	-	174.1, C	-	174.1, C
	29NH_2	7.28 (d, 5.0)<br>7.02 (br s)	-	-	-

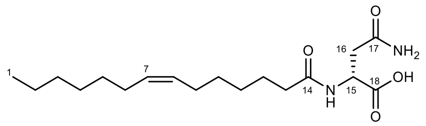

N-(ω-7-myristoyl)-D-asparagine (**19**)

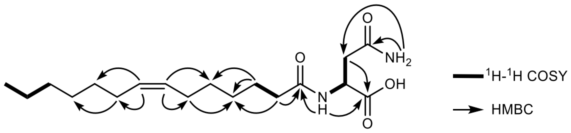

Supplementary Table 11 | ^{1}H (500 MHz) and ^{13}C (125 MHz) NMR data assignments for N-(ω-7-myristoyl)-D-asparagine (**19**) in DMSO-d_6 (for NMR spectra and HRMS see Supplementary Figs. 67–72)[a].

	No.	δ_H (mult., *J*)	δ_C, mult.
FA[a]			
	1	0.86 (t, 6.8)	14.4, CH_3
	2	1.26 (ov)	22.5, CH_2
	3	1.26 (ov)	31.6, CH_2
	4	1.26 (ov)	28.7, CH_2
	5	1.26 (ov)	29.5, CH_2
	6	1.97 (ov)	27.0, CH_2
	7	5.33 (ov)	130.1, CH
	8	5.33 (ov)	130.1, CH
	9	1.97 (ov)	27.1, CH_2
	10	1.26 (ov)	29.6, CH_2
	11	1.26 (ov)	28.7, CH_2
	12	1.47 (m)	25.6, CH_2
	13	2.08 (t, 7.4)	35.7, CH_2
	14	-	172.3, C
D-Asn			
	15	4.35 (br s)	40.9, CH
	15NH	7.79 (br s)	-
	16	2.38 (m)	38.3, CH_2
	17	-	172.3, C
	17NH_2	7.54 (br s)	-
		6.81 (br s)	
	18	-	172.2, C

[a]The geometry of C-7/C-8 double bond was determined to be *cis* by comparing its chemical shifts with a known compound[25] that is a moiety of colibactin.

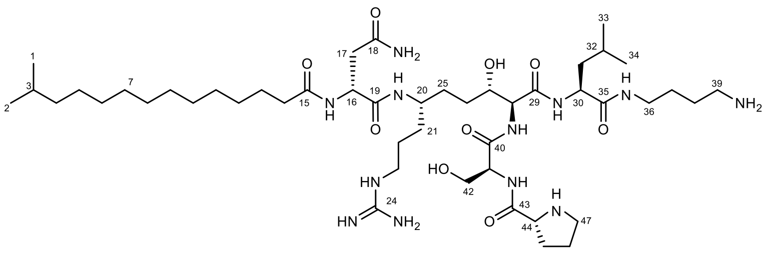
pre-rhabdobranin D (**27**)

Supplementary Table 12 | ^{1}H (700 MHz) and ^{13}C (175 MHz) NMR data assignments for pre-rhabdobranin D (**27**) in DMSO-d_6 (for NMR spectra and HRMS see Supplementary Figs. 80–87)[a].

	No.	δ_H (mult., J)	δ_C, mult.
FA			
	1,2	0.86 (d, 6.6)	23.0, CH_3
	3	1.49 (ov)	27.9, CH
	4	1.13 (br dd, 14.2, 6,7)	39.0, CH_2
	5	1.23 (ov)	27.3, CH_2
	6	1.23 (ov)	29.8, CH_2
	7	1.23 (ov)	29.6, CH_2
	8	1.23 (ov)	29.6, CH_2
	9	1.23 (ov)	29.6, CH_2
	10	1.23 (ov)	29.5, CH_2
	11	1.23 (ov)	29.4, CH_2
	12	1.23 (ov)	29.2, CH_2
	13	1.45 (ov)	25.7, CH_2
	14	2.09 (t, 7.5)	35.7, CH_2
	15	-	172.8, C
D-Asn			
	16NH	8.06 (d, 7.8)	-
	16	4.49 (dd, 14.1, 7.8)	50.5, CH
	17	2.48 (ov)	38.0, CH_2
		2.38 (dd, 15.0, 7.8)	
	18	-	172.2, C
	18NH_2	7.40 (s)	-
		6.91 (s)	
	19	-	171.5, C
L-Arg/polyketide			
	20NH	7.63 (d, 8.7)	-
	20	3.66 (m)	47.9, CH
	21	1.43 (ov)	31.9, CH_2
		1.30 (m)	
	22	1.45 (ov)	20.4, CH_2
	23	3.02 (ov)	40.9, CH_2
	24	-	157.8, C
	25	1.44 (ov)	30.9, CH_2
	26	1.42 (ov)	29.8, CH_2
		1.36 (m)	
	27	3.62 (m)	70.7, CH
	28	4.15 (m)	58.7, CH
	28NH	8.19 (d, 7.0)	-
	29	-	170.8, C
L-Leu			
	30NH	8.15 (d, 7.4)	-
	30	4.12 (m)	52.0, CH
	31	1.48 (ov)	40.5, CH_2
	32	1.61 (ov)	24.6, CH
	33	0.86 (d, 6.6)	23.6, CH_3
	34	0.79 (d, 6.6)	21.5, CH_3
	35	-	172.4, C
putrescine			
	36NH	7.69 (t, 5.5)	-
	36	3.02 (ov)	38.4, CH_2
	37	1.57 (ov)	26.1, CH_2
		1.43 (ov)	
	38	1.48 (ov)	25.0, CH_2

	39	2.74 (t, 7.2)	38.9, CH_2
L-Ser			
	40	-	171.1, C
	41NH	8.29 (d, 7.4)	-
	41	4.36 (dd, 12.9, 5.6)	55.0, CH
	42	3.68 (dd, 10.8, 5.6)<br>3.51 (dd, 10.8, 5.6)	64.4, CH_2
	43	-	174.8, C
D-Pro			
	44	3.60 (m)	60.4, CH
	45	1.94 (ddd, 15.4, 12.4, 8.3)<br>1.69 (dt, 12.4, 6.8)	30.6, CH_2
	46	1.57 (ov)<br>1.43 (ov)	26.3, CH_2
	47	2.87 (dt, 10.1, 6.6)<br>2.78 (dt, 10.1, 6.6)	47.0, CH_2

[a]The stereochemistry was predicted by analyzing the *rdb1* BGC.

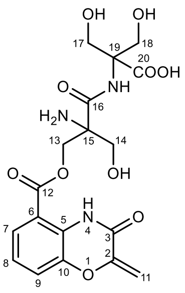

benzobactin A (**28**)

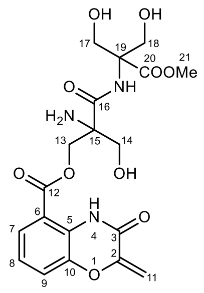

benzobactin A methyl ester (**29**)

Supplementary Table 13 | ^{1}H (500 MHz) and ^{13}C (125 MHz) NMR data assignments for benzobactin A (**28**) and its methyl ester (**29**) in DMSO-d_6 (for NMR spectra and HRMS see Supplementary Figs. 88–99).

	No.	benzobactin A		benzobactin A methyl ester	
		δ_H (mult., *J*)	δ_C, mult.	δ_H (mult., *J*)	δ_C, mult.
benzoxazolinate					
	2	-	147.5, C	-	148.0, C
	3	-	154.9, C	-	155.4, C
	4	11.36 (s)	-	11.37 (s)	-
	5	-	126.0, C	-	126.5, C
	6	-	111.2, C	-	111.7, C
	7	7.52 (dd, 8.0, 1.3)	123.0, CH	7.52 (dd, 8.0, 1.3)	123.5, CH
	8	7.15 (t, 8.0)	122.8, CH	7.15 (t, 8.0)	123.4, CH
	9	7.34 (br d, 7.4)	118.9, CH	7.34 (br d, 8.0)	119.4, C
	10	-	141.0, C	-	141.5, C
	11	5.52 (d, 1.5)<br>5.19 (d, 1.5)	98.7, CH_2	5.53 (d, 1.6)<br>5.20 (d, 1.6)	99.2, CH_2
	12	-	163.1, C	-	163.6, C
2-hydroxymethyl Ser-1					
	13	4.52 (d, 8.4)<br>4.47 (d, 8.4)	70.6, CH_2	4.51 (d, 8.5)<br>4.48 (d, 8.5)	71.0, CH_2
	14	3.83 (d, 11.4)<br>3.67 (d, 10.7)	65.2, CH_2	3.84 (ov)<br>3.64 (ov)	65.5, CH_2
	14-OH	nd	-	5.30 (t, 5.5)	-
	15	-	80.4, C	-	80.8, C
	16	-	170.7, C	-	171.3, C
2-hydroxymethyl Ser-2					
	17	3.88 (d, 10.7)<br>3.64 (dd, 11.1, 3.6)	60.4, CH_2	3.83 (ov)<br>3.83 (ov)	60.7, CH_2[a]
	17-OH	nd	-	4.99 (ov)	-
	18	3.67 (d, 10.7)<br>3.64 (dd, 11.1, 3.6)	60.4, CH_2	3.69 (m)	60.5, CH_2[a]
	18-OH	nd		4.99 (ov)	-
	19	-	66.7, C	-	67.2, C
	19-NH	7.61 (s)	-	7.53 (s)	
	20	-	172.4, C	-	171.9, C
	21	-	-	3.63 (s)	52.7, CH_3

[a]exchangeable signals. nd = not detectable.

Supplementary Table 17 | Crystallographic data collection and refinement statistics of yCP:IOC.

	yCP:IOC
**Crystal parameters**	
Space group	$P2_1$
Cell constants	a = 135.1 Å
	b = 301.5 Å
	c = 144.3 Å
	β = 112.9 °
CPs / AU[a]	1
**Data collection**	
Beam line	X06SA, SLS
Wavelength (Å)	1.0
Resolution range (Å)[b]	30–3.0 (3-1–3.0)
No. observations	635463
No. unique reflections[c]	203696
Completeness (%)[b]	96.2 (98.6)
R_{merge} (%)[b, d]	8.5 (57.7)
I/ σ (I)[b]	9.1 (2.6)
**Refinement (REFMAC5)**	
Resolution range (Å)	30-3.0
No. refl. working set	193349
No. refl. test set	10176
No. non hydrogen	49509
No. of ligand atoms	66
Solvent (H_2O, ions, MES)	138
R_{work}/R_{free} (%)[e]	17.8 / 21.5
r.m.s.d. bond (Å) / angle (°)[f]	0.002 / 1.2
Average B-factor ($Å^2$)	90.5
Ramachandran Plot (%)[g]	97.4 / 2.3 / 0.3
PDB accession code	7O2L

[a] Asymmetric unit
[b] The values in parentheses for resolution range, completeness, R_{merge} and I/σ (I) correspond to the highest resolution shell
[c] Data reduction was carried out from a single crystal. Friedel pairs were treated as identical reflections
[d] $R_{merge}(I) = \Sigma_{hkl}\Sigma_j \mid I(hkl)_j - \langle I(hkl)\rangle \mid / \Sigma_{hkl} \Sigma_j I(hkl)_j$, where $I(hkl)_j$ is the jth measurement of the intensity of reflection hkl and $\langle I(hkl)\rangle$ is the average intensity
[e] $R = \Sigma_{hkl} \mid |F_{obs}| - |F_{calc}| \mid / \Sigma_{hkl} |F_{obs}|$, where R_{free} is calculated without a sigma cut off for a randomly chosen 5% of reflections, which were not used for structure refinement, and R_{work} is calculated for the remaining reflections
[f] Deviations from ideal bond lengths/angles
[g] Percentage of residues in favored / allowed / outlier region

a

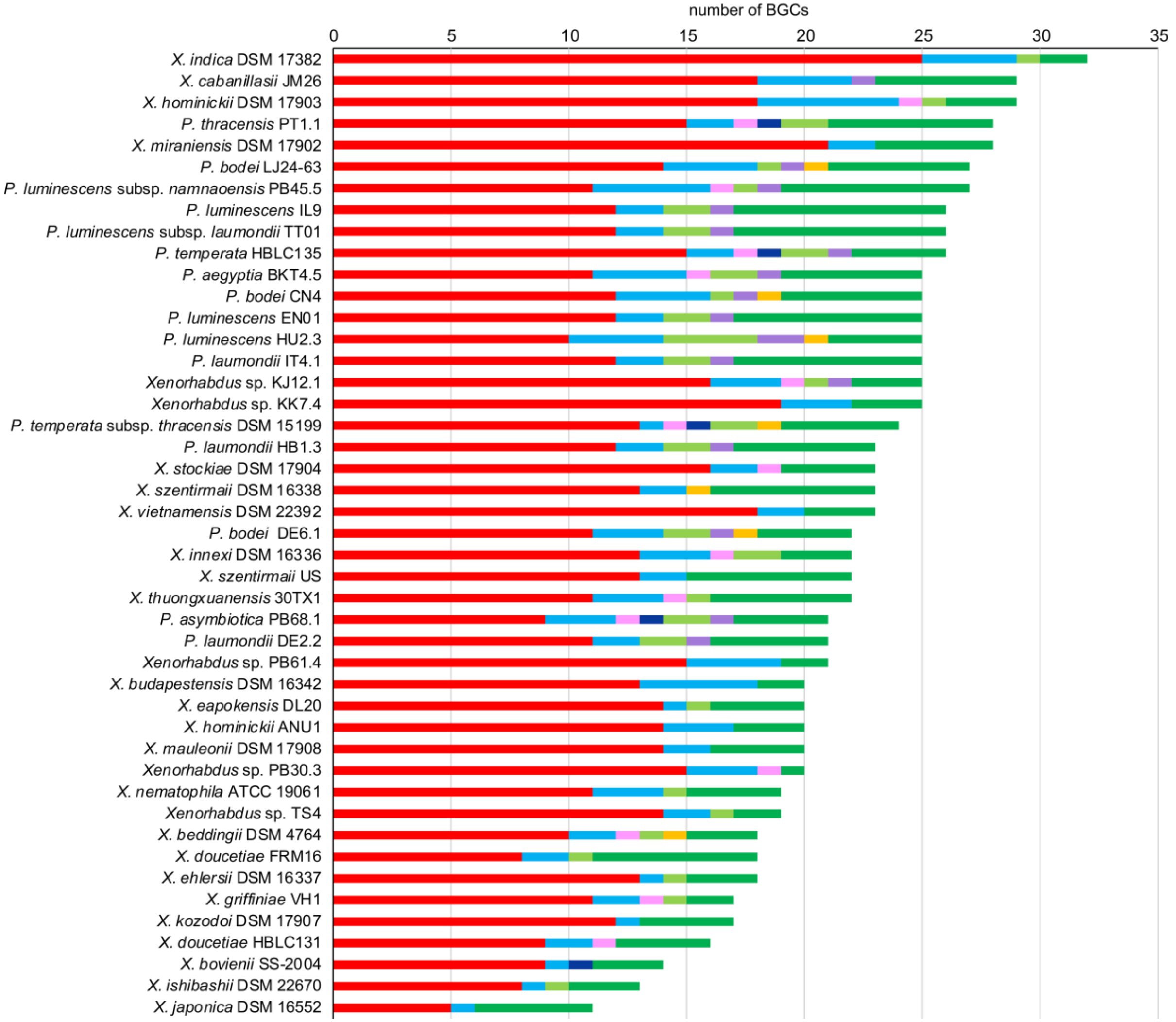

number of BGCs
0
5
10
15
20
25
30
35
X. indica DSM 17382
X. cabanillasii JM26
X. hominickii DSM 17903
P. thracensis PT1.1
X. miraniensis DSM 17902
P. bodei LJ24-63
P. luminescens subsp. namnaoensis PB45.5
P. luminescens IL9
P. luminescens subsp. laumondii TT01
P. temperata HBLC135
P. aegyptia BKT4.5
P. bodei CN4
P. luminescens EN01
P. luminescens HU2.3
P. laumondii IT4.1
Xenorhabdus sp. KJ12.1
Xenorhabdus sp. KK7.4
P. temperata subsp. thracensis DSM 15199
P. laumondii HB1.3
X. stockiae DSM 17904
X. szentirmaii DSM 16338
X. vietnamensis DSM 22392
P. bodei DE6.1
X. innexi DSM 16336
X. szentirmaii US
X. thuongxuanensis 30TX1
P. asymbiotica PB68.1
P. laumondii DE2.2
Xenorhabdus sp. PB61.4
X. budapestensis DSM 16342
X. eapokensis DL20
X. hominickii ANU1
X. mauleonii DSM 17908
Xenorhabdus sp. PB30.3
X. nematophila ATCC 19061
Xenorhabdus sp. TS4
X. beddingii DSM 4764
X. doucetiae FRM16
X. ehlersii DSM 16337
X. griffiniae VH1
X. kozodoi DSM 17907
X. doucetiae HBLC131
X. bovienii SS-2004
X. ishibashii DSM 22670
X. japonica DSM 16552

b

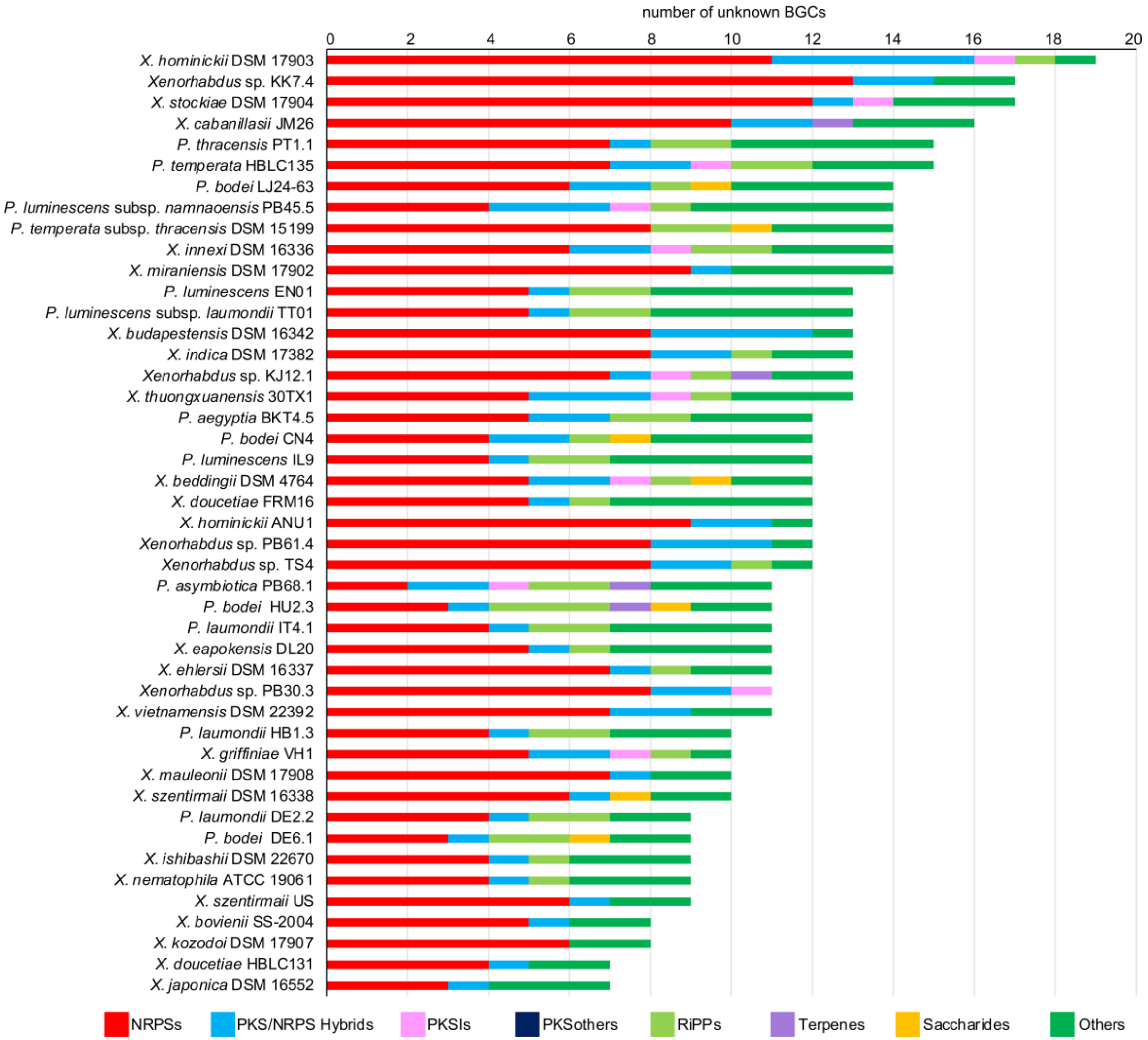

Supplementary Fig. 1 | The number and classes of (a) total BGCs (including fragments) annotated by antiSMASH 5.0 and (b) unknown BGCs in each *XP* genome.

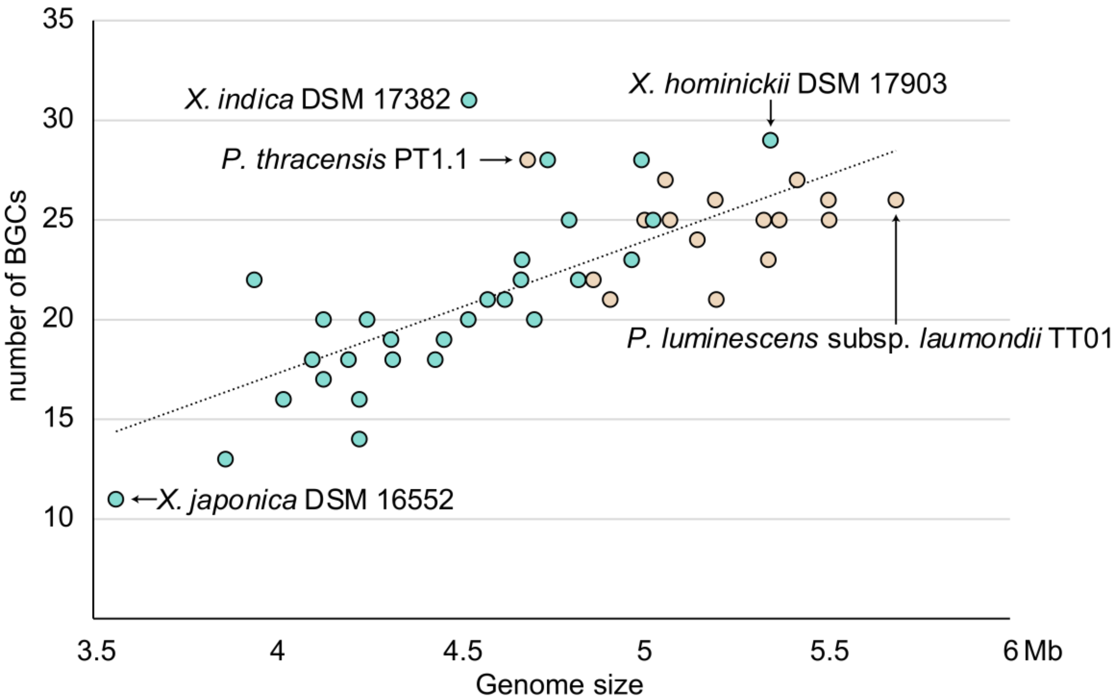

Supplementary Fig. 2 | Relationship between predicted BGCs and genome size for each strain. Strains that harbor the most and fewest BGCs, as well as the largest and smallest genome sizes are denoted. *Xenorhabdus* genomes, blue-green nodes. *Photorhabdus* genomes, grey-yellow nodes.

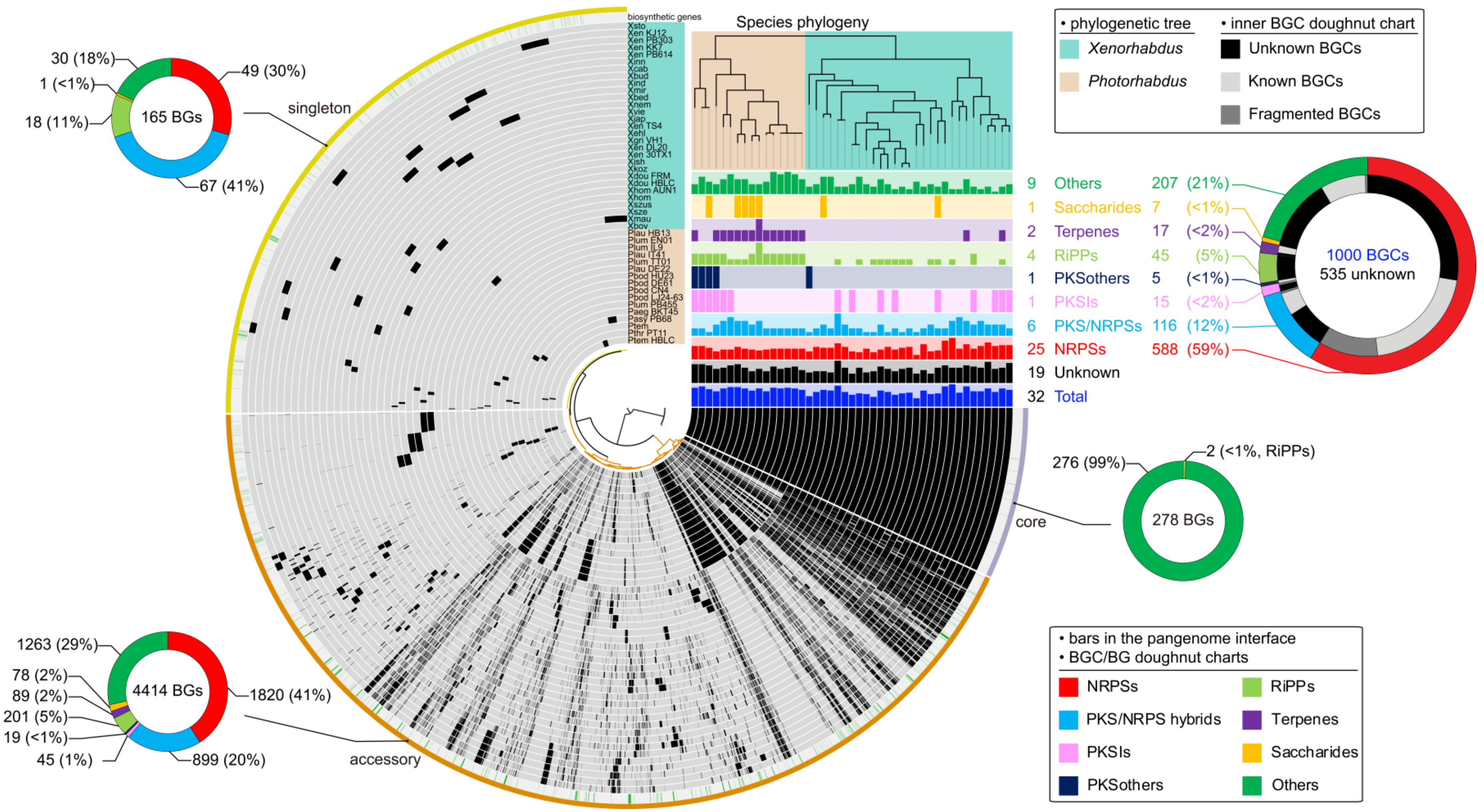

Supplementary Fig. 3 | Pangenomic analysis and BGC overview of 45 *XP* genomes by anvi'o with additional statistical data. The maximum number and classification of each BGC class are indicated on the right side of the bar charts. The total number of BGC in each class and its percentage are indicated on the left side of the double-layer BGC doughnut chart. The numbers of BGs and their percentages are labeled on the BG doughnut charts.

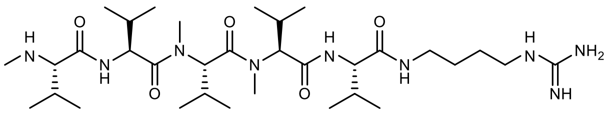

rhabdopeptide/xenortide-like peptide

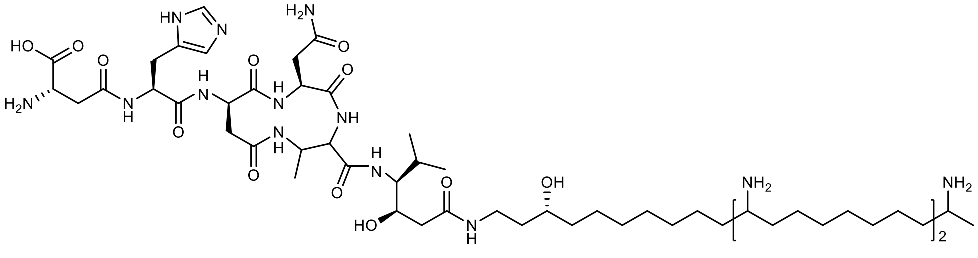

fabclavine IVa

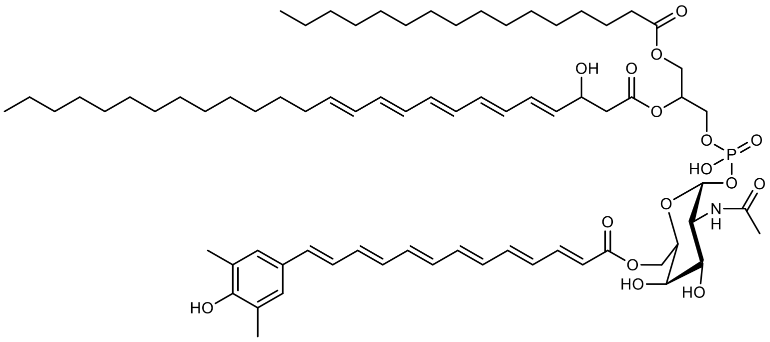

APEL-1284

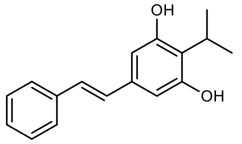

isopropylstilbene

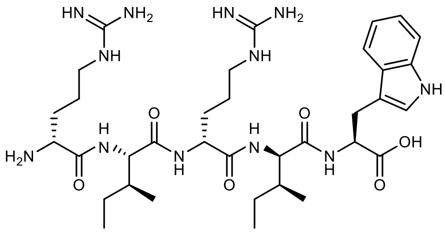

ririwpeptide A

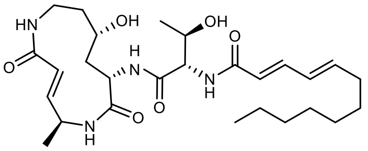

glidobactin A

Supplementary Fig. 4 | Known natural products widely distributed in *Xenorhabdus* and/or *Photorhabdus*. Rhabdopeptide/xenortide-like peptides[26] are the second most broadly distributed non-ribosomal peptide in *XP*. Fabclavines[27] are the most prevalent *Xenorhabdus*-specific polyketide/non-ribosomal peptide hybrid. Arylpolyene lipids (APELs)[28] are the most prominent compound class in Gram-negative bacteria[7] and are exclusively present in *Xenorhabdus* but absent in *Photorhabdus*. Isopropylstilbene[29] is highly conserved across all *Photorhabdus*. Ririwpeptides[30] and glidobactins[31] are the most widespread *Photorhabdus*-specific non-ribosomal peptide and polyketide/non-ribosomal peptide hybrid, respectively.

a

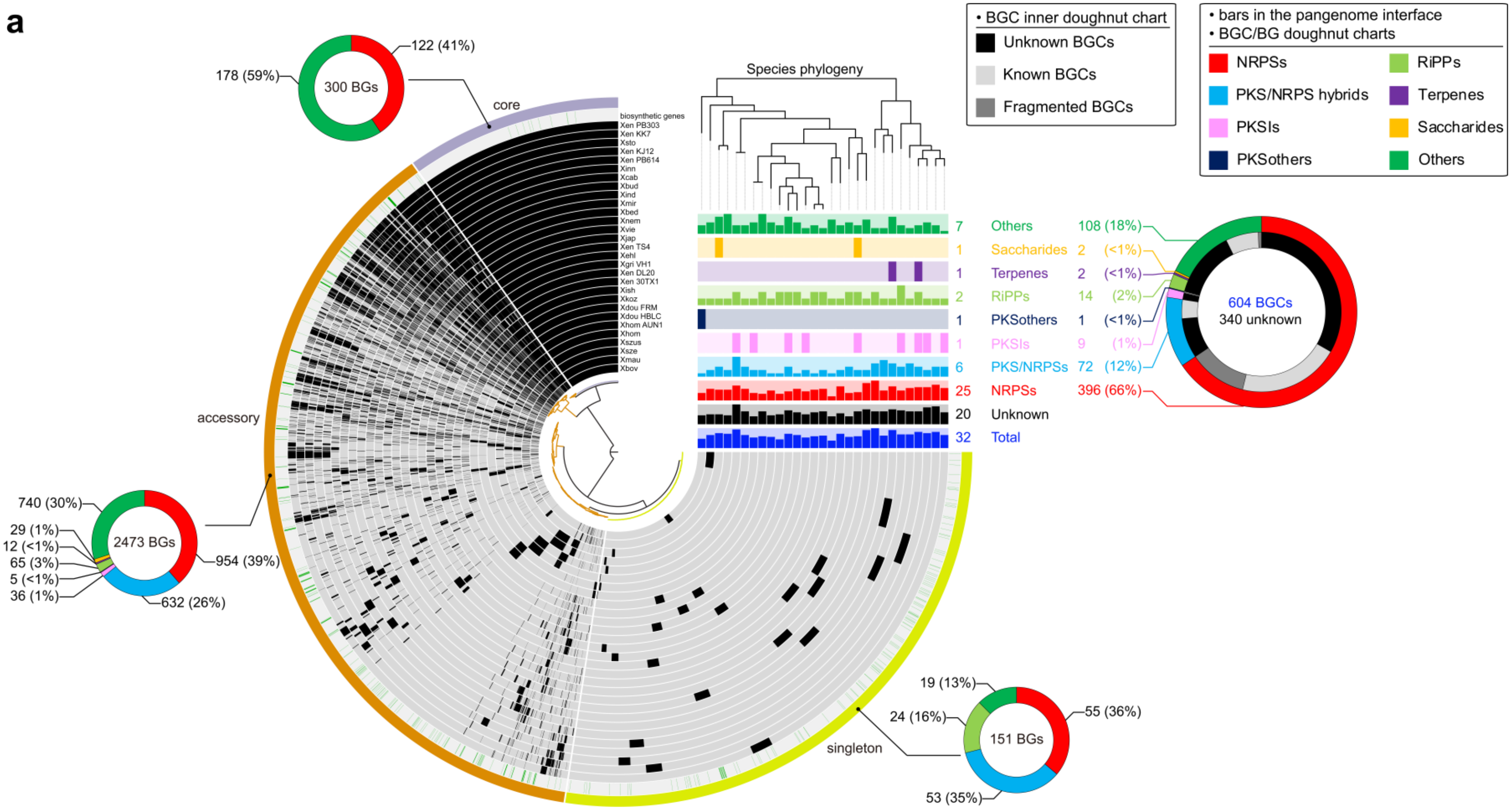

• BGC inner doughnut chart
Unknown BGCs
Known BGCs
Fragmented BGCs
• bars in the pangenome interface
• BGC/BG doughnut charts
NRPSs
PKS/NRPS hybrids
PKSIs
PKSothers
RiPPs
Terpenes
Saccharides
Others
Species phylogeny
300 BGs
122 (41%)
178 (59%)
core
biosynthetic genes
Xen PB303
Xen KK7
Xsto
Xen KJ12
Xen PB614
Xinn
Xcab
Xbud
Xind
Xmir
Xbed
Xnem
Xvie
Xjap
Xen TS4
Xehl
Xgri VH1
Xen DL20
Xen 30TX1
Xish
Xkoz
Xdou FRM
Xdou HBLC
Xhom AUN1
Xhom
Xszus
Xsze
Xmau
Xbov
7 Others 108 (18%)
1 Saccharides 2 (<1%)
1 Terpenes 2 (<1%)
2 RiPPs 14 (2%)
1 PKSothers 1 (<1%)
1 PKSIs 9 (1%)
6 PKS/NRPSs 72 (12%)
25 NRPSs 396 (66%)
20 Unknown
32 Total
604 BGCs
340 unknown
accessory
2473 BGs
740 (30%)
29 (1%)
12 (<1%)
65 (3%)
5 (<1%)
36 (1%)
632 (26%)
954 (39%)
singleton
151 BGs
19 (13%)
24 (16%)
53 (35%)
55 (36%)

b

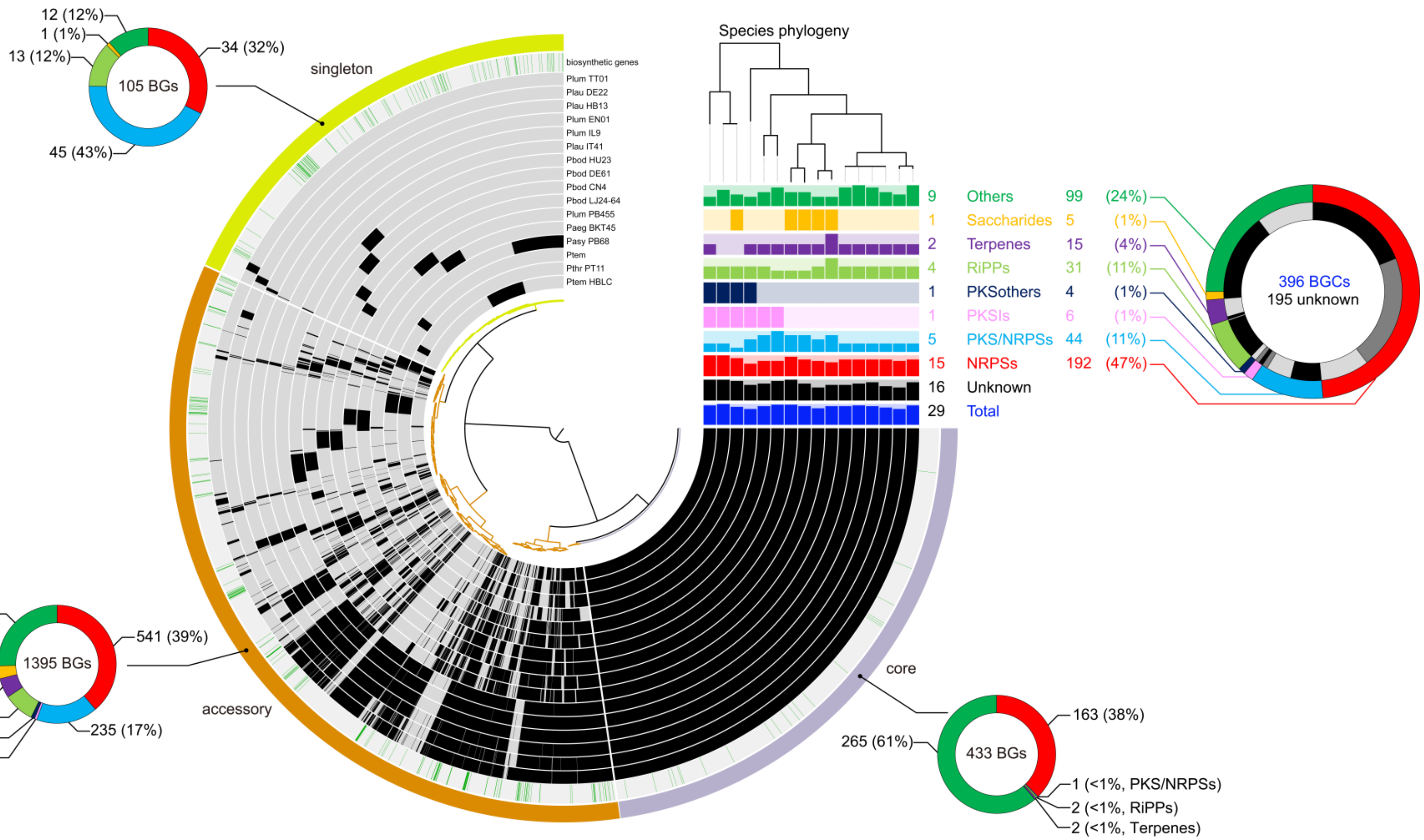

Supplementary Fig. 5 | Pangenomic and BGs/BGCs analysis of 29 *Xenorhabdus* and 16 *Photorhabdus* genomes. Overview of classifications and distribution of BGs/BGCs in (**a**) 29 *Xenorhabdus* and (**b**) 16 *Photorhabdus*. The central plot of the interface represents a hierarchical clustering dendrogram based on gene presence/absence. In the circle interface, each layer (grey) represents all genes (black) in a single genome; distributions of biosynthetic genes (green); bin names, core region (grey-purple), accessory region (orange), singleton region (yellow); core region contains genes present in (**a**) all 29 *Xenorhabdus* genomes or (**b**) all 16 *Photorhabdus* genomes; accessory region contains genes common to some (**a**) *Xenorhabdus* or (**b**) *Photorhabdus* genomes; singleton region contains species-specific genes present in only one of the genomes. BGC distributions

in a strain are represented by the bar charts under the species phylogeny. The maximum number and classification of each BGC are indicated on the right side of the bar charts. Double-layer BGC doughnut charts provide an overview of the proportion of each BGC class (outer layer) and unknown/known/fragmented BGCs (inner layer) in (**a**) 29 *Xenorhabdus* and (**b**) 16 *Photorhabdus*. The total number of BGC in each class and its percentage are indicated on the left side of the BGC doughnut charts. Unknown BGCs, BGCs without connections to known BGCs in the BiG-SCAPE network (Fig. 3). Known BGCs, previously experimentally identified BGCs or those with connections to the MIBiG references in Fig. 3. Fragmented BGCs, resulting from incomplete genome sequencing. BG doughnut charts (labeled with numbers and percentages) represent the proportion of the BGC class that the BGs belong to in different pangenomic regions. The numbers of BGCs in total, unknown BGCs, and BGs are indicated inside the BGC/BG doughnut charts.

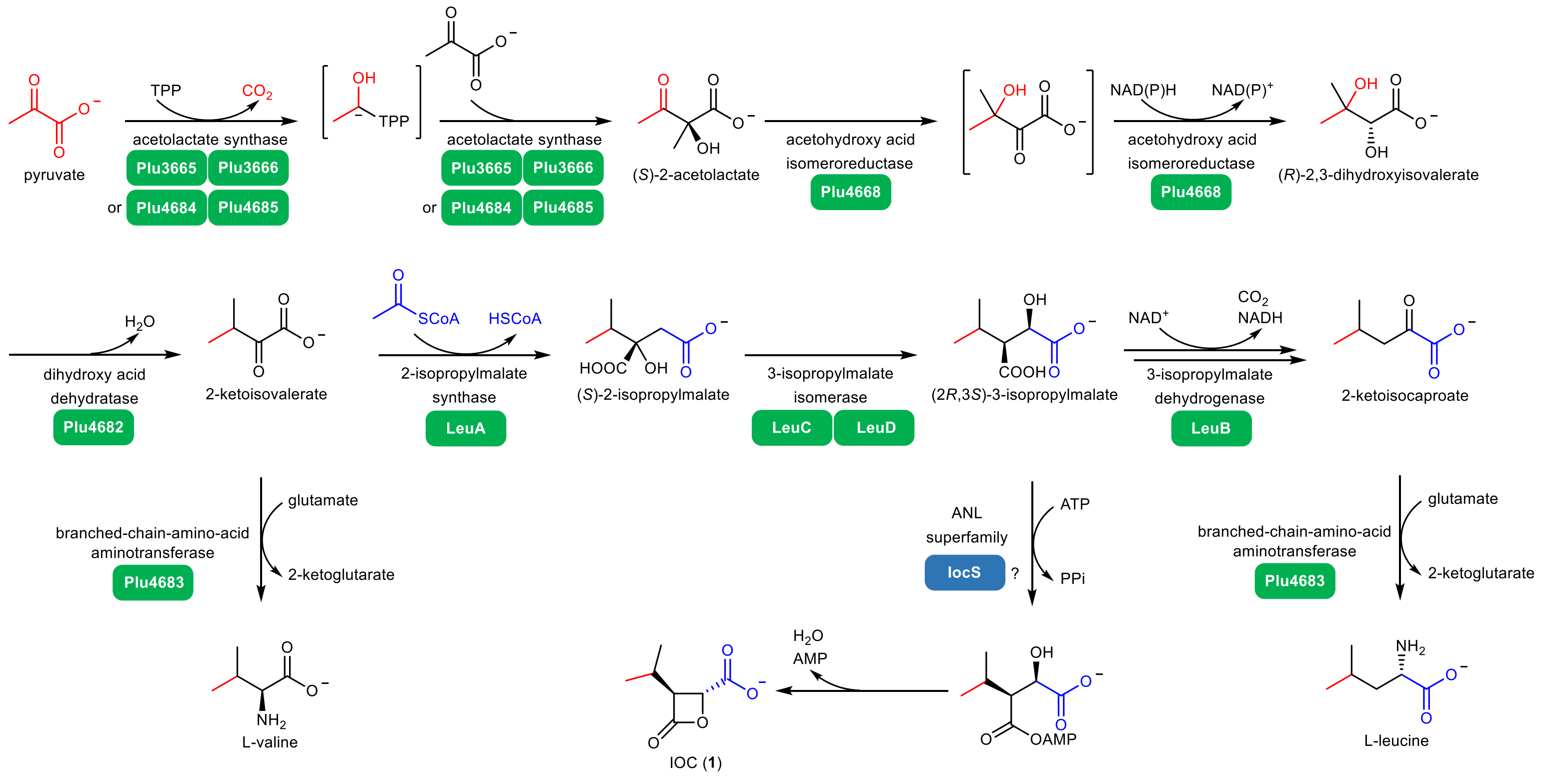

Supplementary Fig. 6 | Proposed biosynthetic pathway of IOC (1) in *P. luminescens* subsp. *laumondii* TT01.

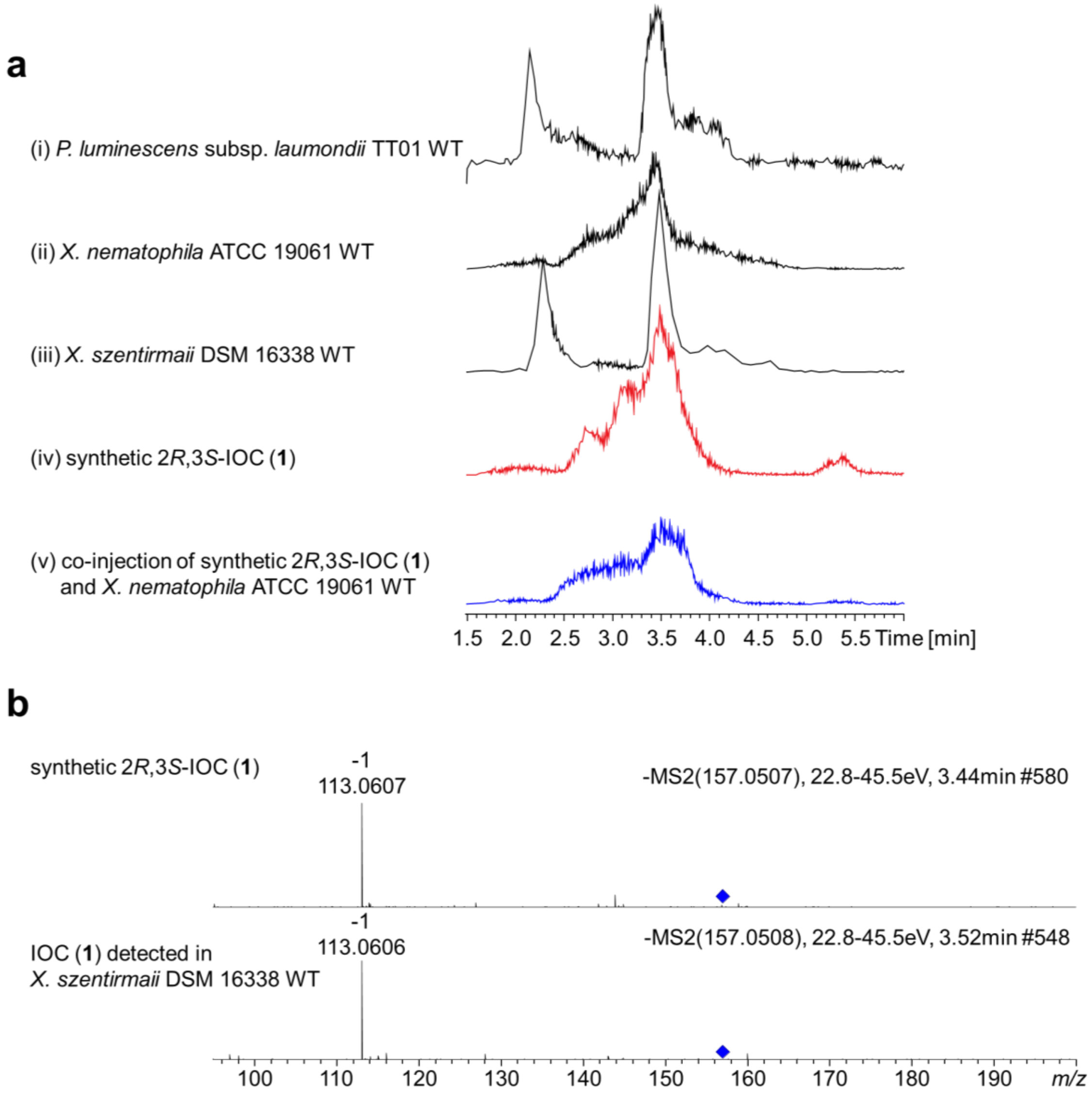

Supplementary Fig. 7 | HPLC-MS analysis of IOC in different *Xenorhabdus* and *Photorhabdus* strains in Sf-900 medium. a, 157.05075 $[M - H]^-$ EICs of the culture supernatants of (i) *P. luminescens* subsp. *laumondii* TT01 WT, (ii) *X. nematophila* ATCC 19061 WT, and (iii) *X. szentirmaii* DSM 16338 WT, as well as (iv) synthetic 2*R*,3*S*-IOC (**1**) and (v) co-injection of synthetic 2*R*,3*S*-IOC (**1**) and the culture supernatant of *X. nematophila* ATCC 19061 WT. **b,** Comparison of the MS/MS fragmentation patterns of the synthetic 2*R*,3*S*-IOC (**1**) and IOC (**1**) detected in the culture supernatant of *X. szentirmaii* DSM 16338 WT. The blue diamond indicates the parent ions. Representative data from three independent experiments are shown.

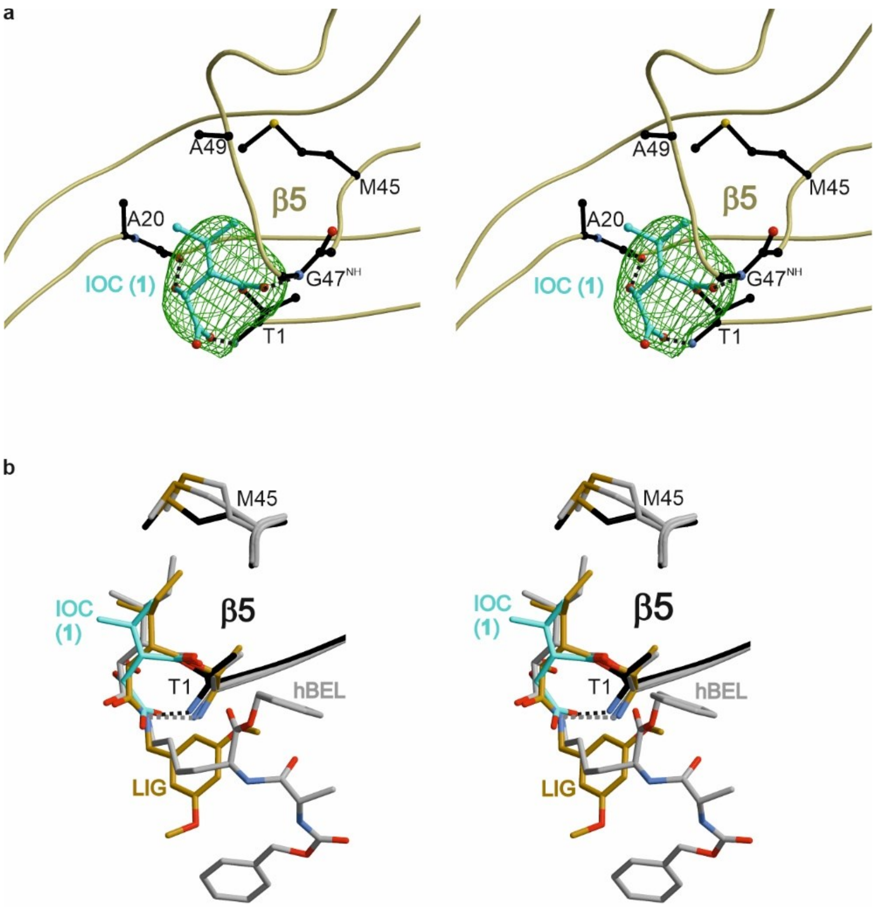

Supplementary Fig. 8 | Stereo representations of IOC (1). a, F_O−F_C electron density map (green mesh, contoured to 3σ, IOC has been omitted prior to phase calculations) of **1** covalently linked through an ester bond to Thr1O$^{\gamma}$ of subunit β5. Protein residues interacting with **1** are highlighted in black. Dots illustrate hydrogen bonds between **1** and protein residues. **b,** Superposition of **1** (cyan, PDB ID 7O2L), homobelactosin C (hBEL, grey, PDB ID 3E47)[32] and (3*S*)-((1*S*)-methylpropyl)-4-oxooxetane-(2*R*)-carboxamide (LIG, gold, PDB ID 4Z1L)[33] complex structures with the yeast 20S proteasome at the chymotrypsin-like active site.

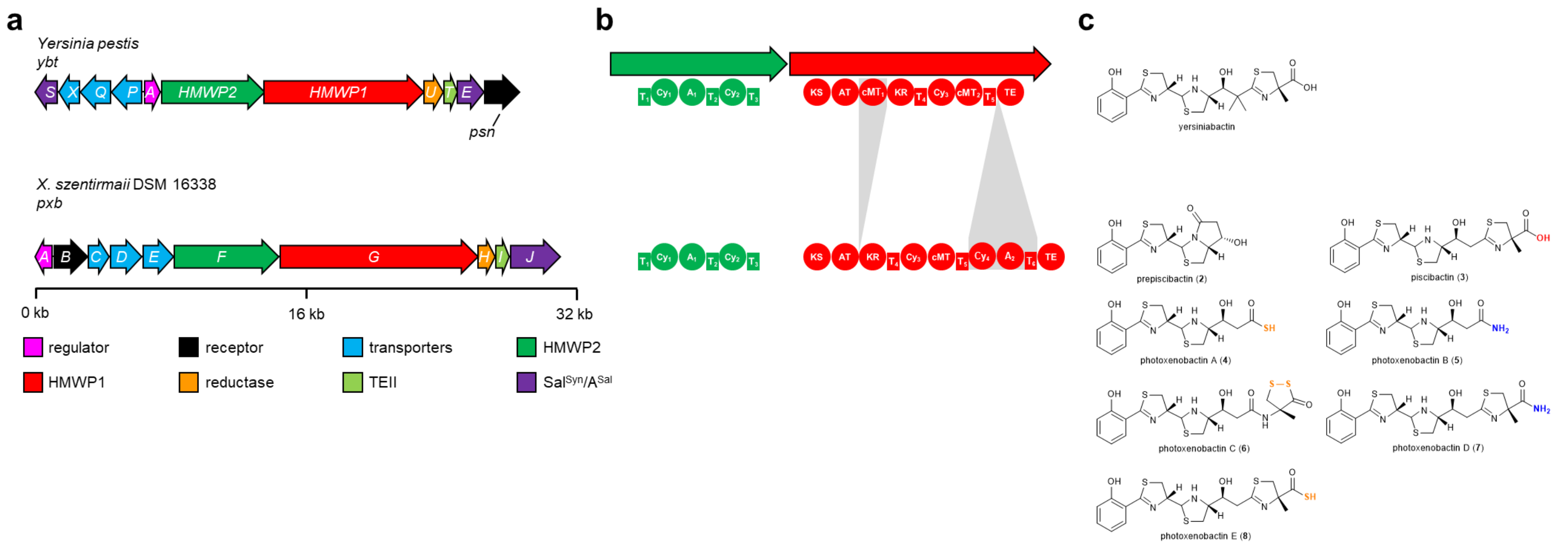

Supplementary Fig. 9 | BGCs and chemical structures of yersiniabactin and photoxenobactins. a, Comparison of yersiniabactin-related BGCs in *Yersinia pestis* (*ybt*) and *X. szentirmaii* (*pxb*). kb, kilobase. **b,** Domain organization of HMWP1 and HMWP2 homologs encoded by two BGCs. Domain differences are indicated with shades of gray. T, thiolation; A, adenylation; Cy, heterocyclization; KS, ketosynthase; AT, acyltransferase; KR, ketoreductase; cMT, carbon methyltransferase; and TE, thioesterase domains. **c,** Known chemical structures, yersiniabactin from *Y. pestis* and prepiscibactin (**2**) and piscibactin (**3**) from *Photobacterium damselae* subsp. *piscida*[22], as well as previously unidentified photoxenobactins A–E (**4**–**8**) from *X. szentirmaii* DSM 16338. The terminal heteroatoms are highlighted.

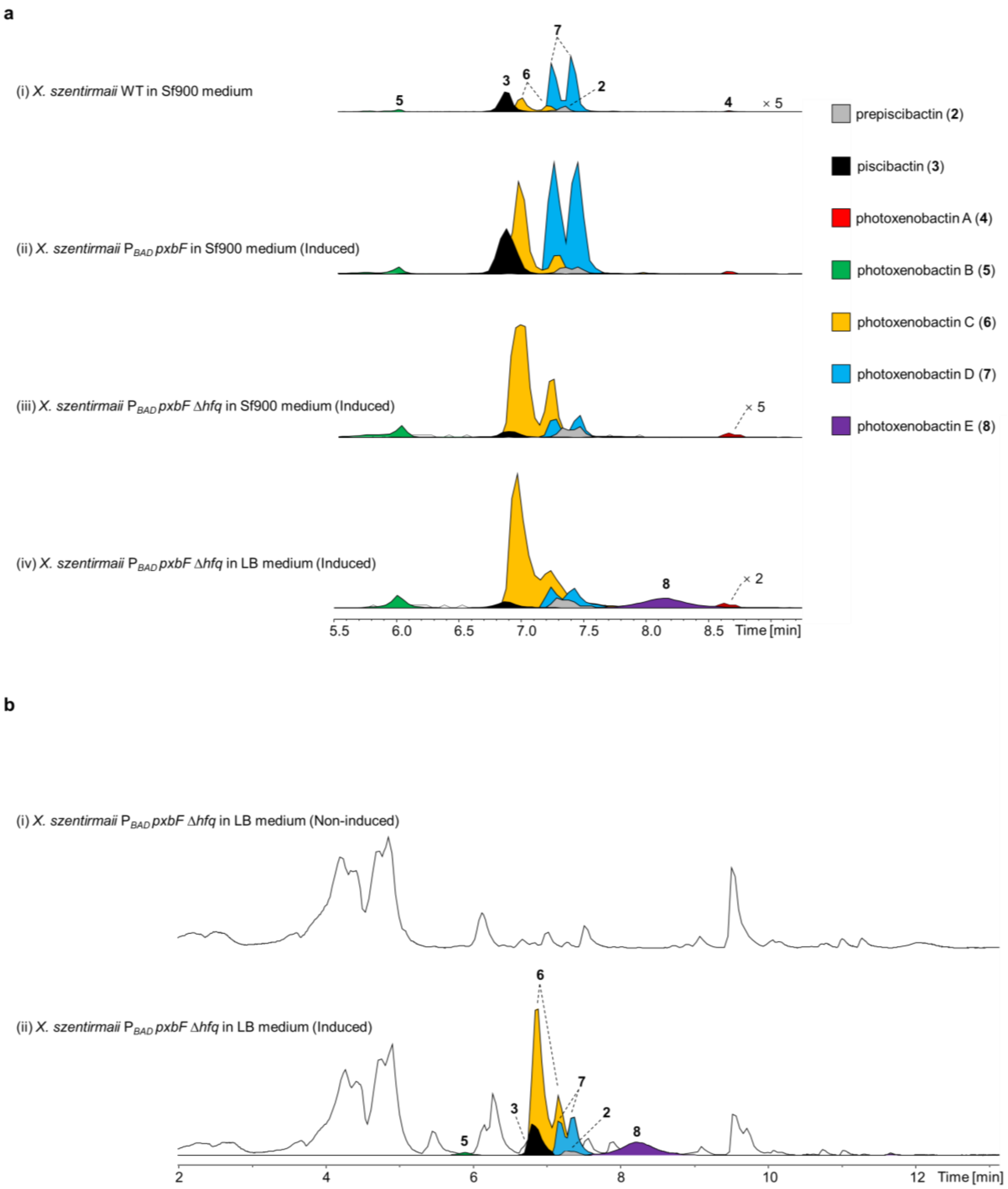

Supplementary Fig. 10 | HPLC-MS analysis of photoxenobactins and piscibactins in *X. szentirmaii* DSM 16338 wild-type strain and the promoter exchange mutants thereof in different media. a, EICs of the (i) wild-type strain and (ii-iv) mutants with L-arabinose induction. Shown are (i-iv) prepiscibactin (**2**), piscibactin (**3**), and photoxenobactins A (**4**), B (**5**), C (**6**), D (**7**), and E (**8**). **2**–**7** are present in the wild-type strain with low production titers. Each compound contains a pair of C-10 epimers, which were not differentiated. Intensities in traces (i), (iii), and (iv) are magnified for visualizing tiny peaks. Magnifications are indicated on the right side of traces or on the top of the peak. **b,** BPCs of promoter exchange of the Δ*hfq* mutant (i) without and (ii) with L-arabinose induction. Desired peaks are highlighted in (**b**) the BPC of trace ii. Photoxenobactin E (**8**) was produced in a detectable amount in the *X. szentirmaii* P_{BAD} *pxbF* Δ*hfq* mutant. Representative data from three independent experiments are shown.

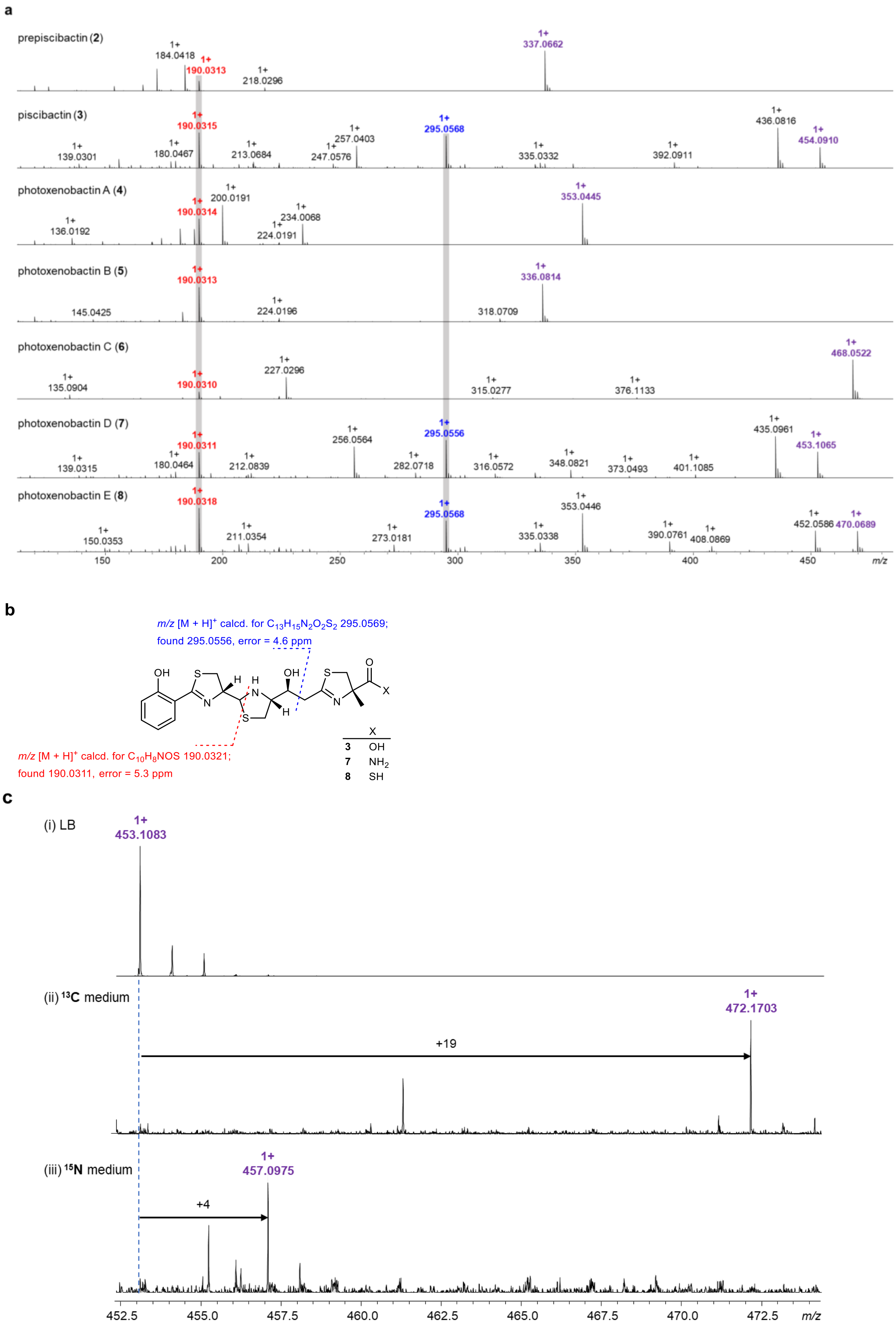
a
prepiscibactin (2)
piscibactin (3)
photoxenobactin A (4)
photoxenobactin B (5)
photoxenobactin C (6)
photoxenobactin D (7)
photoxenobactin E (8)
m/z
b
m/z [M + H]+ calcd. for C13H15N2O2S2 295.0569;
found 295.0556, error = 4.6 ppm
m/z [M + H]+ calcd. for C10H8NOS 190.0321;
found 190.0311, error = 5.3 ppm
X
3 OH
7 NH2
8 SH
c
(i) LB
1+ 453.1083
(ii) 13C medium
1+ 472.1703
+19
(iii) 15N medium
1+ 457.0975
+4
m/z

Supplementary Fig. 11 | MS/MS of piscibactins and photoxenobactins (2–8) and isotope labeling experiments for photoxenobactin D (7). a, Comparison of MS/MS fragmentation patterns of prepiscibactin (**2**), piscibactin (**3**), and photoxenobactins A–E (**4**–**8**). Purple masses indicate parent ions. **b,** *m/z* 190.031 $[M + H]^+$ fragment ion indicating a hydroxyphenylthiazoline moiety[23] is highlighted in red in (**a**). *m/z* = 295.056 $[M + H]^+$ highlighted blue in (**a**) is a common fragment ion shared by piscibactin (**3**) and photoxenobactins D (**7**) and E (**8**). **c,** Structural elucidation of photoxenobactin D (**7**) by MS analyses of the isotope labeling experiments. Black arrows indicate mass shifts. The mass shift in (ii) the ^{13}C labeling medium indicated that the number of carbon atoms is 19 in **7**, the same as that of **3**. A mass shift of 4 Da in (iii) the ^{15}N labeling medium indicated that **7** has four nitrogen atoms. Thus, **7** is one nitrogen more than **3**, consistent with the sum formula predicted by HRMS (Supplementary Table 5). The MS/MS fragments of **3** and **7** are highly similar in the low mass region up to *m/z* = 295.056 $[M + H]^+$. This suggested that the 1-Da difference between **3** and **7** lies in the C terminus, in which the carboxylic acid of **3** is replaced by a carboxamide in **7**.

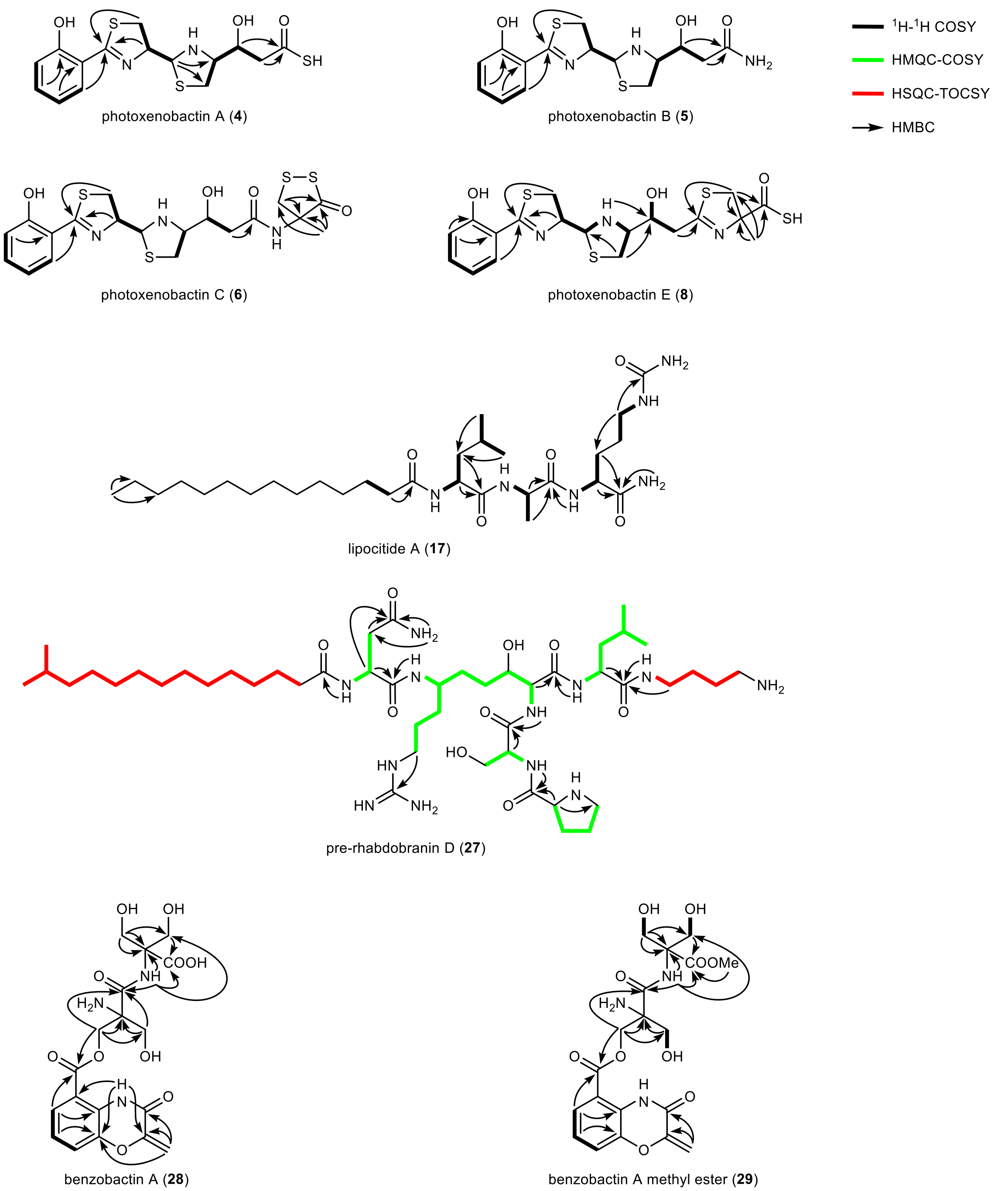

Supplementary Fig. 12 | 2D NMR correlations of new natural products produced by *XP* strains.

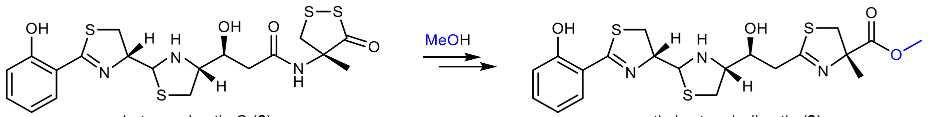

photoxenobactin C (**6**) methyl ester piscibactin (**9**)

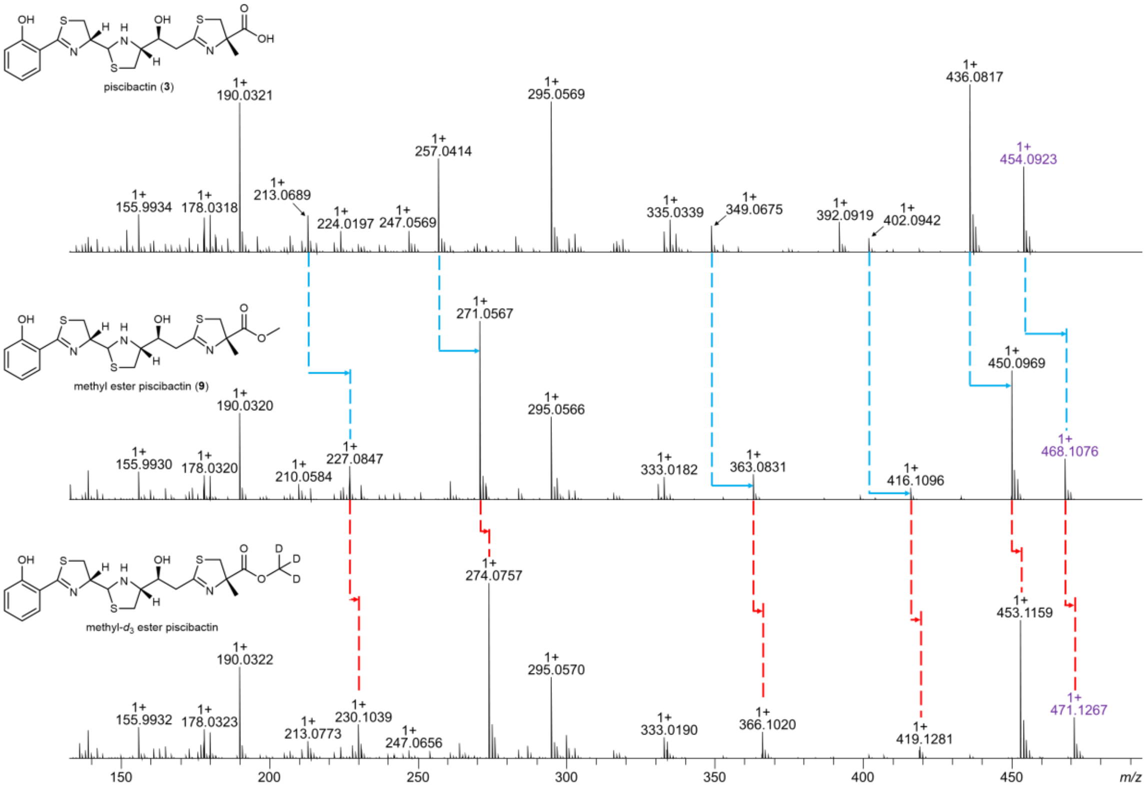

Supplementary Fig. 13 | Proposed conversion of photoxenobactin C (6) in methanol. The conversion of photoxenobactin C (**6**) into methyl ester piscibactin (**9**) in methanol is dramatically accelerated under heating and UV light. Fragmentation patterns with a 14-Da shift between piscibactin (**3**) and methyl ester piscibactin (**9**) shown with blue arrows were observed in the incubation of photoxenobactin C (**6**) in methanol. This indicates a difference in the methyl group. Fragmentation patterns with a 3-Da shift between methyl ester piscibactin (**9**) and methyl-d_3 ester piscibactin that was obtained by the incubation of photoxenobactin C (**6**) in methanol-d_4 are shown with red arrows, indicating a difference in the d_3-methyl group.

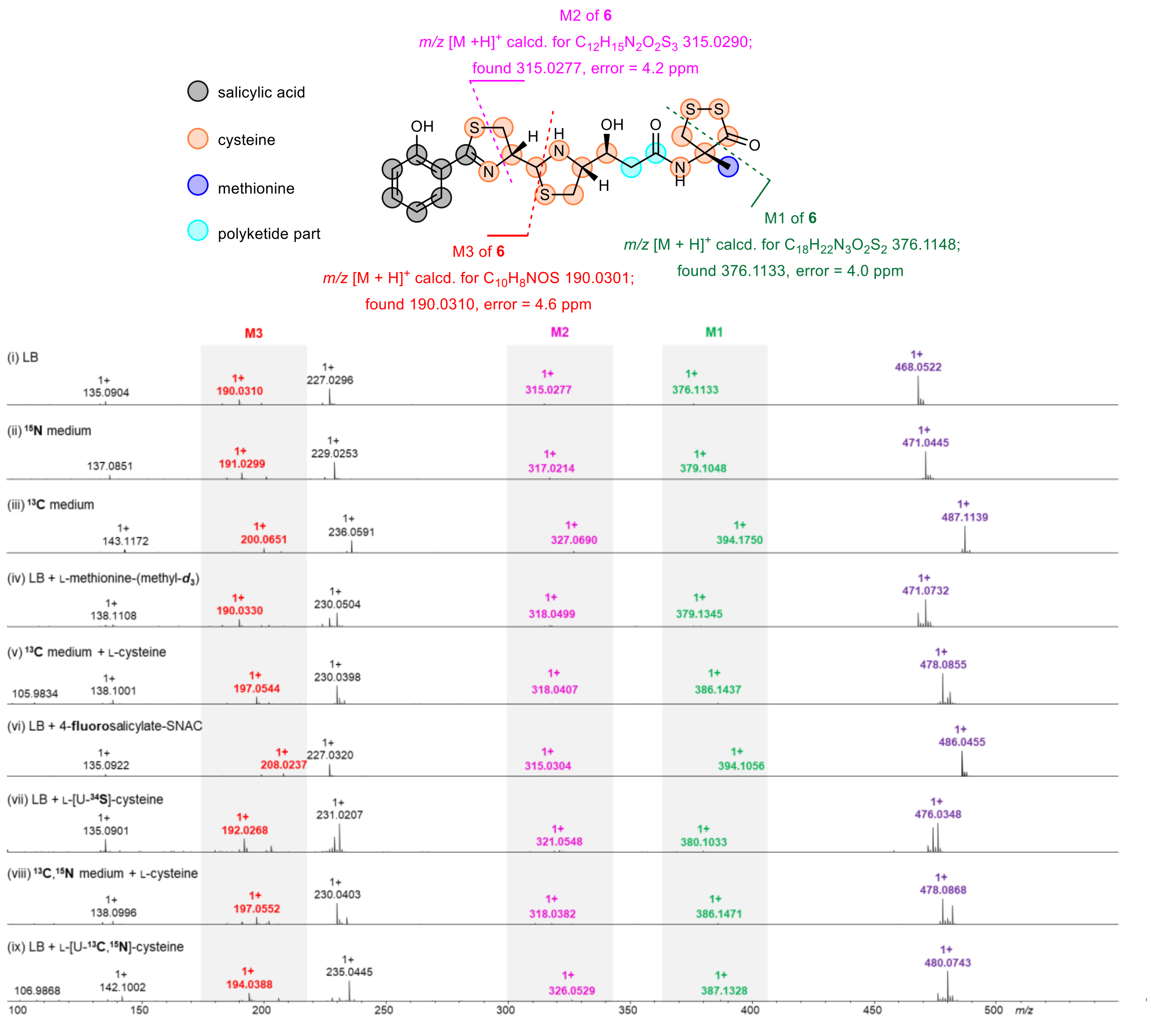

Supplementary Fig. 14 | Mass spectrometry fragmentation patterns of photoxenobactin C (6) resulting from (ii-ix) labeling experiments. Positions incorporated with different building blocks that were supported by labels and/or MS^2 are shown as colored spheres. Purple masses indicate parent ions ($M - H_2O + H^+$) and fragment ions M1–3 are green, pink, and red.

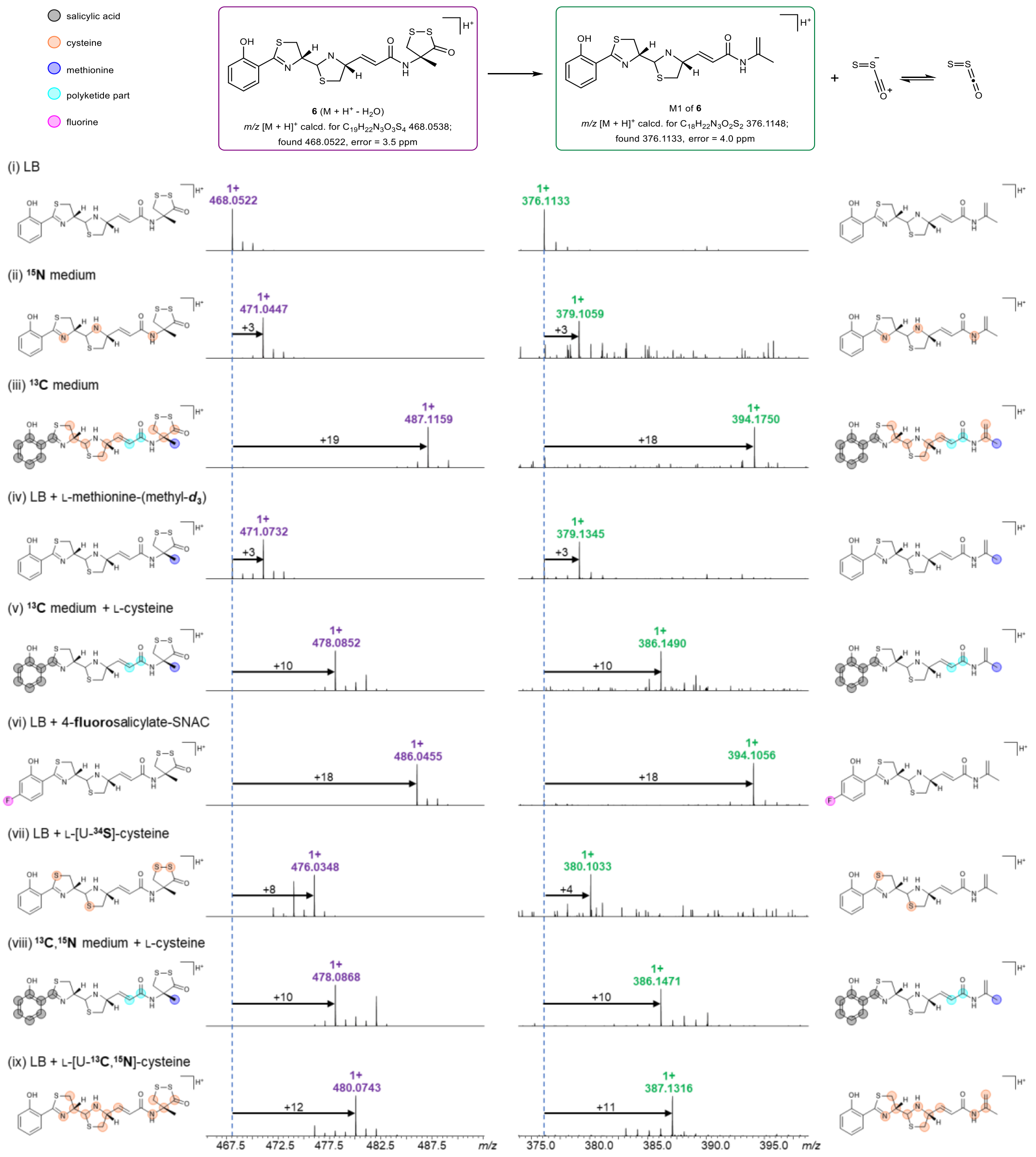

Supplementary Fig. 15 | Mass spectrometry identification of photoxenobactin C (6) and a dithioperoxoate moiety thereof by (ii-ix) labeling experiments. Positions shown as colored spheres are incorporated with labels in corresponding experiments. Purple and green masses/frames indicate parent ions (M – H_2O + H^+) and fragment ions M1, respectively. Black arrows indicate mass shifts. The number of nitrogen and carbon atoms was confirmed by (ii) ^{15}N and (iii) ^{13}C labeling media. A mass shift of 3 Da in (iv) L-methionine-(methyl-d_3) feeding showed the incorporation of one *S*-adenosylmethionine-derived methyl group. (vi) 4-Fluorosalicylate-SNAC supplement with a mass shift of 18 Da confirmed the incorporation of salicylate. (v and viii) Inverse feeding experiments with L-cysteine in ^{13}C & ^{13}C,^{15}N media background together with (ix) L-[U-^{13}C,^{15}N]-cysteine feeding confirmed three cysteine building blocks being part of photoxenobactin C (**6**). However, four sulfur atoms were found to be incorporated by (vii) L-[U-^{34}S]-cysteine feeding. Comparison of the parent masses and fragment ions M1 suggested the C terminus lost a COS_2 moiety which contains a carbon atom as observed in (iii) and (ix) as well as two sulfur atoms as in (vii).

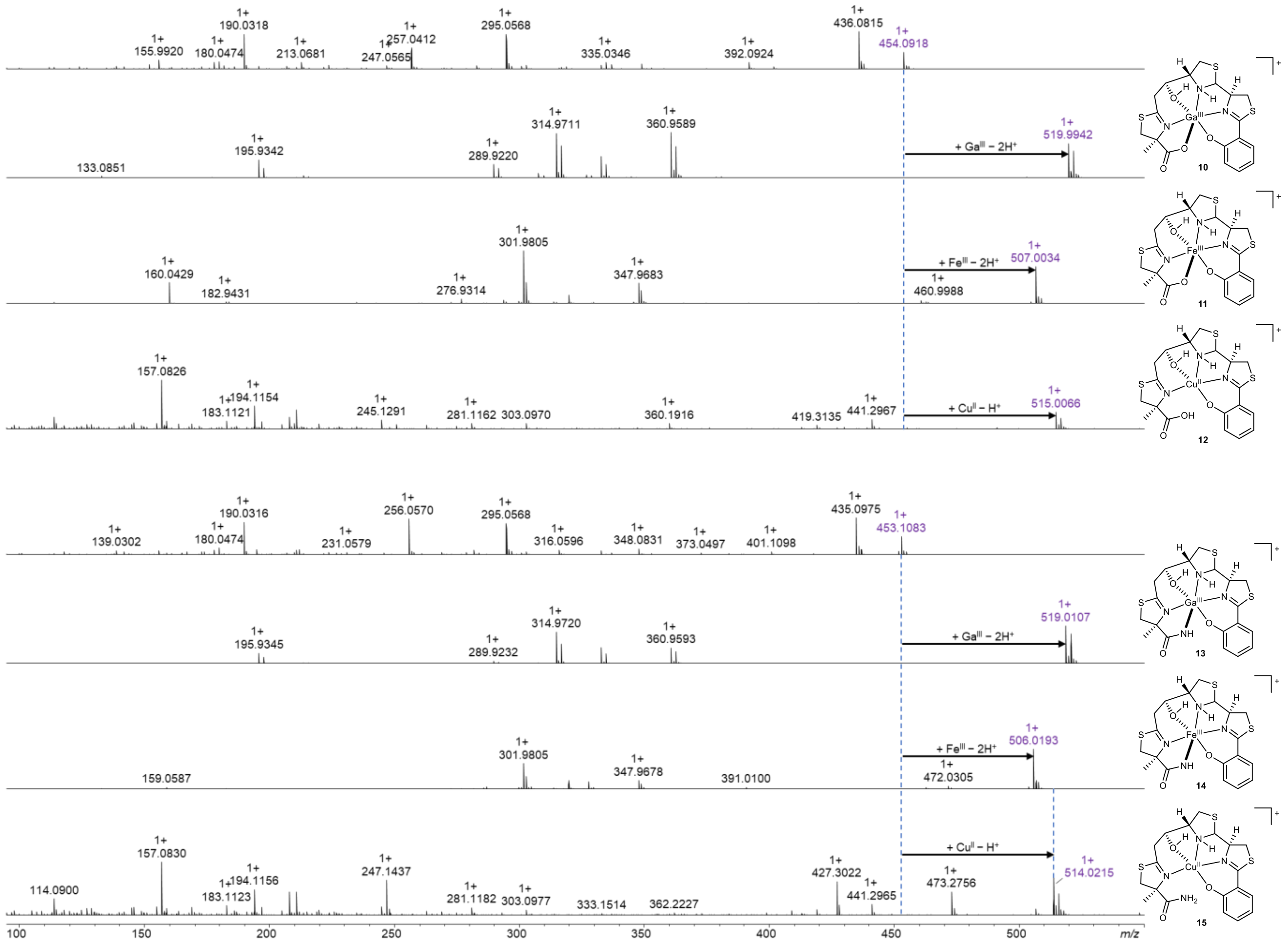

Supplementary Fig. 16 | Metal chelating properties of piscibactin (10–12) and photoxenobactin D (13–15). Ethyl acetate extracts of the *X. szentirmaii* P_{BAD} *pxbF* mutant (induced) were incubated with $Fe(NO_3)_3$, $Ga(NO_3)_3$, and $CuCl_2$. Both piscibactin (**3**) and photoxenobactin D (**7**) can chelate Fe^{III}, Ga^{III}, and Cu^{II}. Purple indicates parent ions ($M + H^+$ or M^+). Black arrows indicate mass shifts.

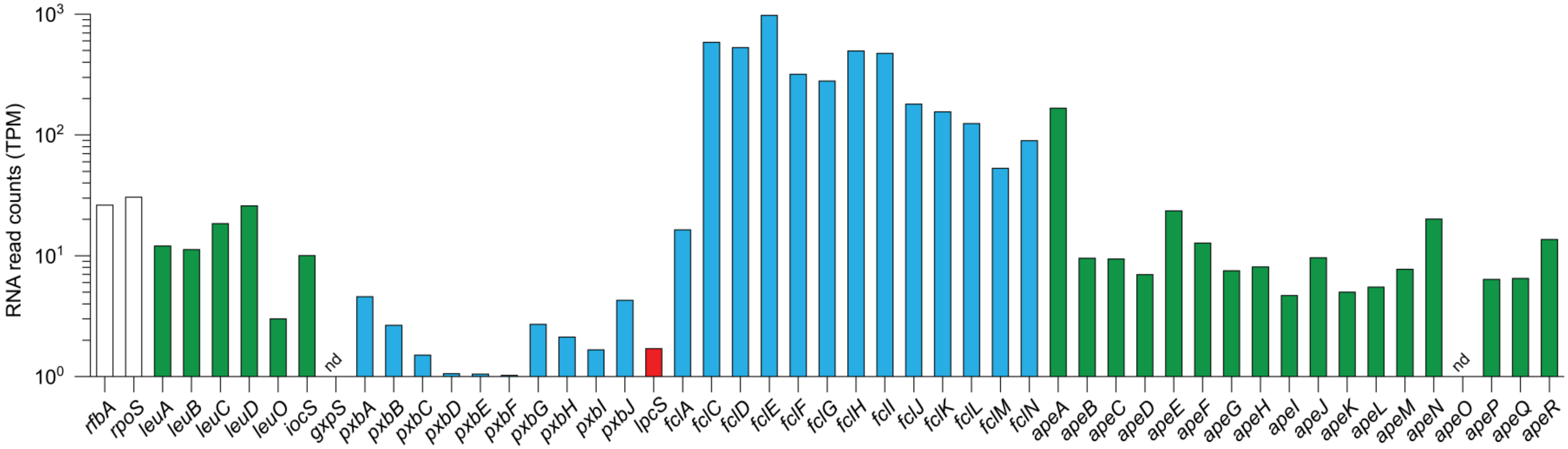

Supplementary Fig. 17 | Comparison of transcriptional levels of biosynthetic genes in the conserved BGCs (*ioc/leu, gxp, pxb*, *lpc, fcl*, and *ape*) in *X. szentirmaii* US wild-type strain with the housekeeping genes (*rfbA* and *rpoS*).

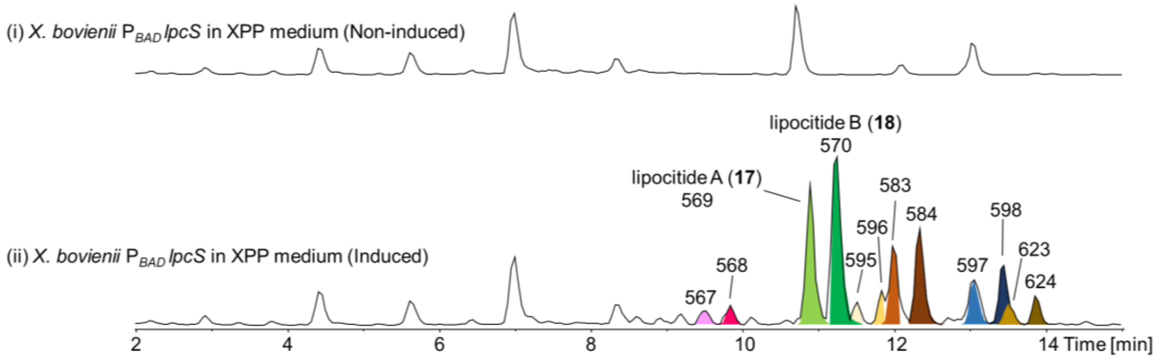

Supplementary Fig. 18 | HPLC-MS analysis of lipocitides in the *X. bovienii* SS-2004 P*BAD* *lpcS* mutant in XPP medium. BPCs of the promoter exchange mutant (i) without and (ii) with L-arabinose induction. Lipocitides are highlighted with colors with corresponding $[M + H]^+$ ions. Representative data from three independent experiments are shown.

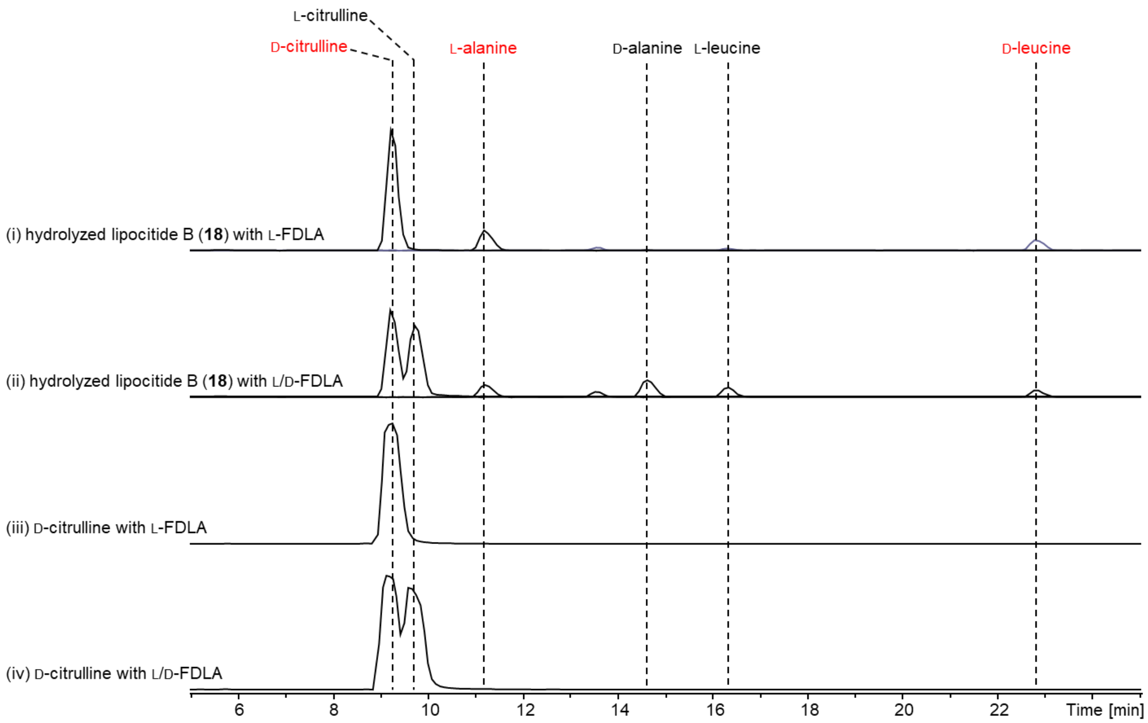

Supplementary Fig. 19 | Absolute configuration determination of lipocitide B (18) by the advanced Marfey's method. HPLC-MS analysis of lipocitide B (**18**) that was hydrolyzed by HCl and subsequently derivatized with L-FDLA and L/D-FDLA. Shown are EICs of FDLA derivatized citrulline, alanine, and leucine. The configuration of amino acids is determined by the elution order. L-FDLA derivatized L-amino acids are eluted prior to D-amino acids[34], except citrulline as illustrated in traces (iii) and (iv). L-FDLA derivatized (iii) D-citrulline has a shorter retention time than (iv) L-citrulline. The determined configuration of an amino acid residue is highlighted in red.

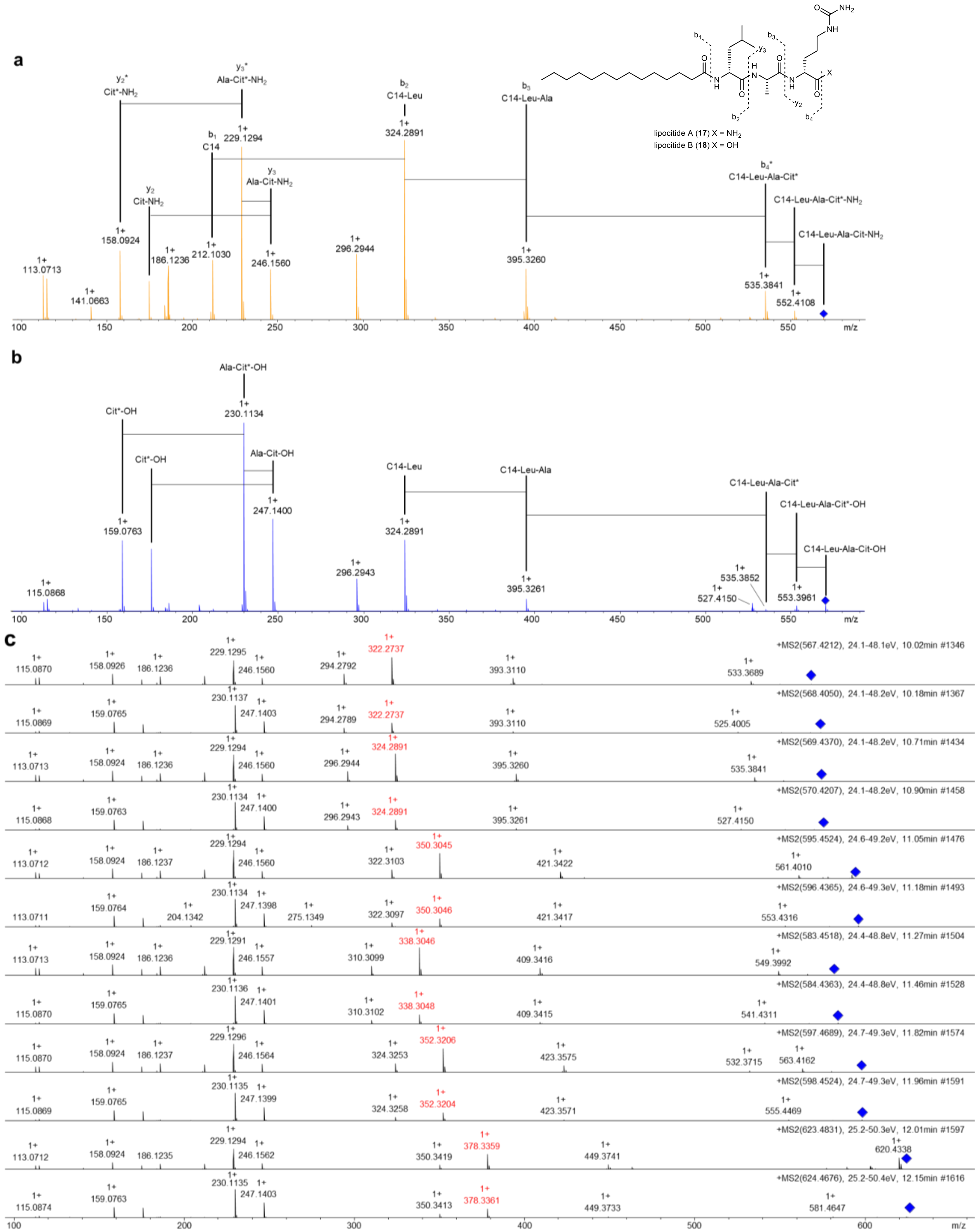

Supplementary Fig. 20 | MS/MS fragmentation patterns of lipocitides. a, Fragmentation patterns of lipocitide A (**17**). **b,** Fragmentation patterns of lipocitide B (**18**). The asterisk in a citrulline and fragment ion represents a loss of NH_2 in the ureido group. **c,** Comparison of MS/MS fragmentation patterns between lipocitides. Fragment ions highlighted in red represent an acyl/Leu pattern, indicating that lipocitides differ in N-acyl groups. The blue diamond indicates the parent ions.

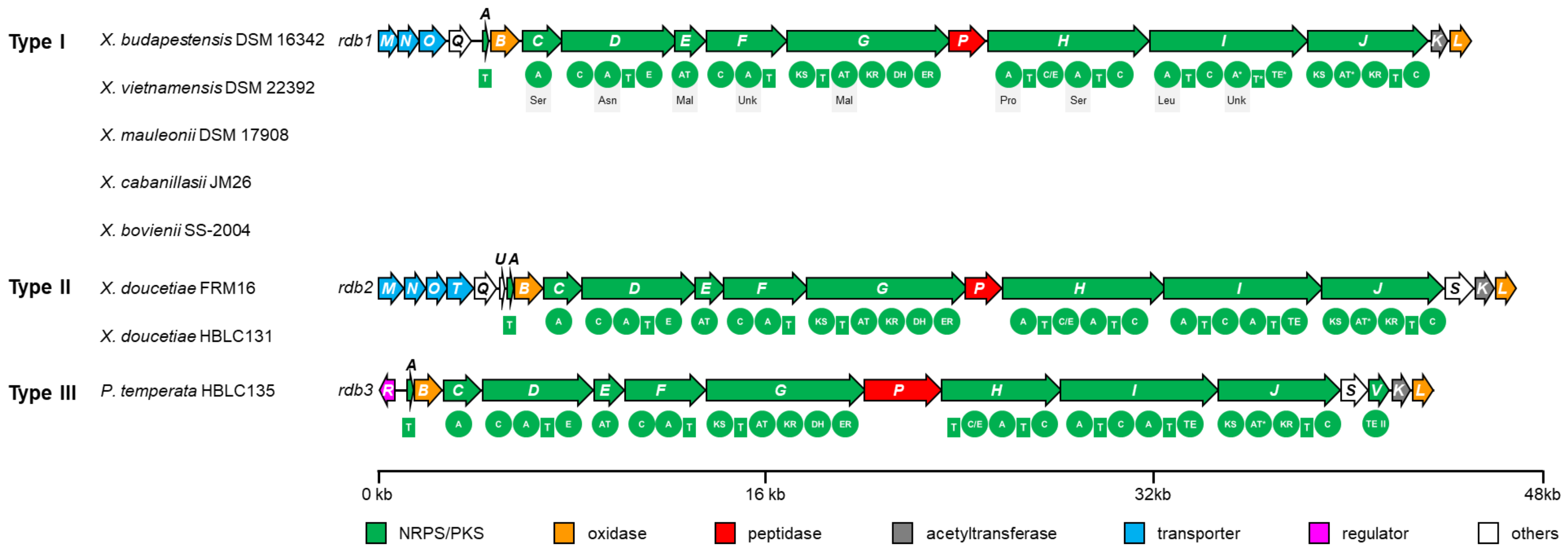

Supplementary Fig. 21 | Domain organization of three types of *rdb* BGCs with predicted substrates of adenylation and acyltransferase domains. *rdb1* encodes a weakly predicted TE domain in Rdb1I, while that encoded in *rdb2* and *rdb3* is clearly annotated. Additionally, an extra type II TE, Rdb3V, is encoded in *rdb3*. Compared with Rdb1H and Rdb2H, the Rdb3H lacks an A domain. T, thiolation; A, adenylation; C, condensation; E, epimerization; AT, acyltransferase; KS, ketosynthase; KR, ketoreductase; DH, dehydratase; ER, enoyl reductase; cMT, carbon methyltransferase; and TE, thioesterase domains. Ser, serine; Asn, asparagine; Mal, malonyl; Pro, proline; Leu, leucine; Unk, unknown. Presumably inactive domain is labeled with an asterisk.

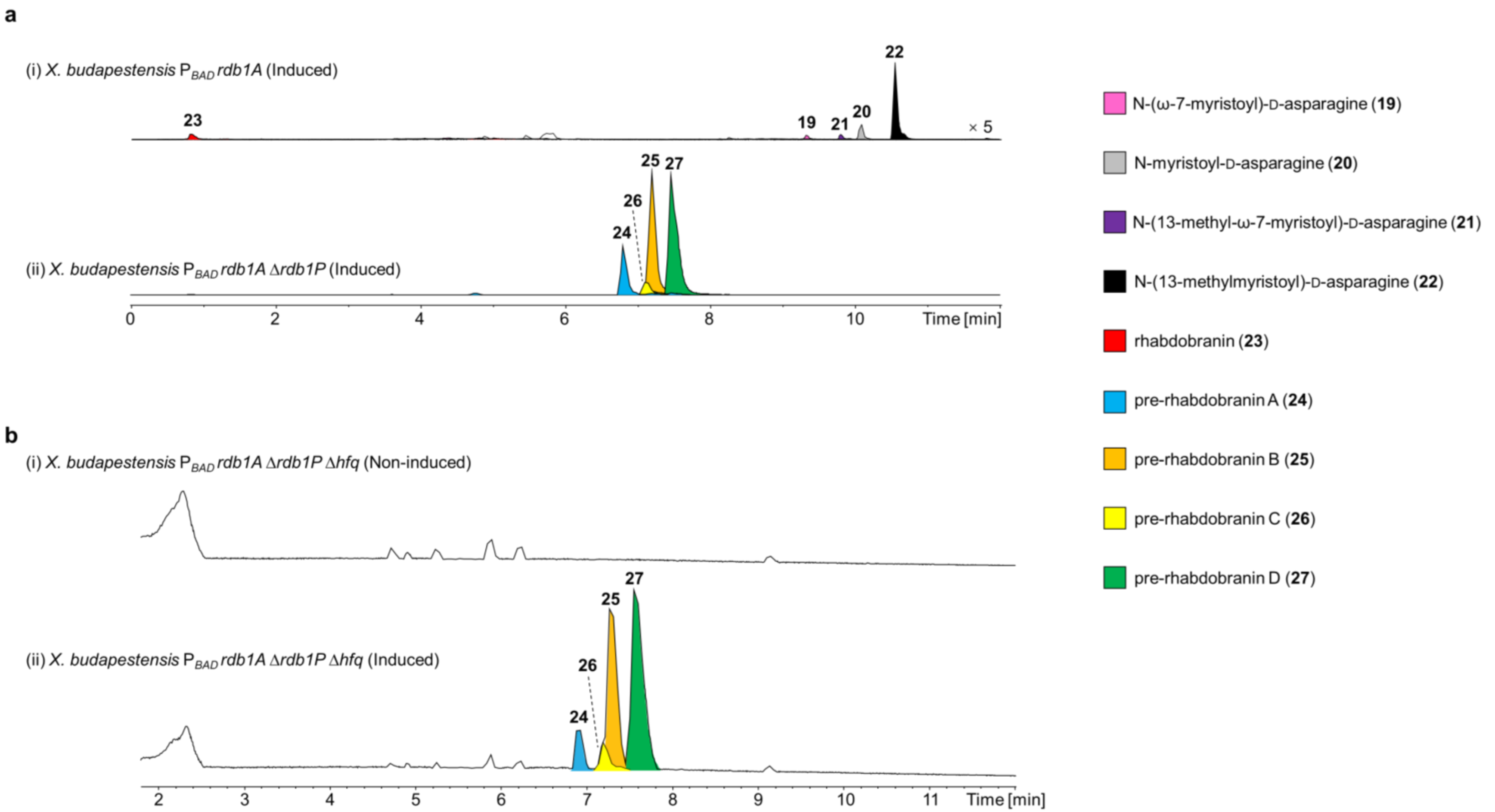

Supplementary Fig. 22 | HPLC-MS analysis of (pre-)rhabdobranins in the promoter exchange mutants of *X. budapestensis* DSM 16342 in LB medium. a, EICs of the *X. budapestensis* P_{BAD} *rdb1A* and *X. budapestensis* P_{BAD} *rdb1A Δrdb1P* mutants. Shown are (i) N-(ω-7-myristoyl)-D-asparagine (**19**), N-myristoyl-D-asparagine (**20**), N-(13-methyl-ω-7-myristoyl)-D-asparagine (**21**), N-(13-methylmyristoyl)-D-asparagine (**22**), and rhabdobranin (**23**); (ii) pre-rhabdobranin A (**24**), pre-rhabdobranin B (**25**), pre-rhabdobranin C (**26**), and pre-rhabdobranin D (**27**). Intensities in trace (i) are magnified for visualizing tiny peaks. Magnifications are indicated on the right side of traces. **b,** BPCs of the (i) non-induced and (ii) induced promoter exchange mutants of the *X. budapestensis* P_{BAD} *rdb1A Δrdb1P Δhfq*. Desired peaks are highlighted in trace (ii). Mutants were induced with L-arabinose. Representative data from three independent experiments are shown.

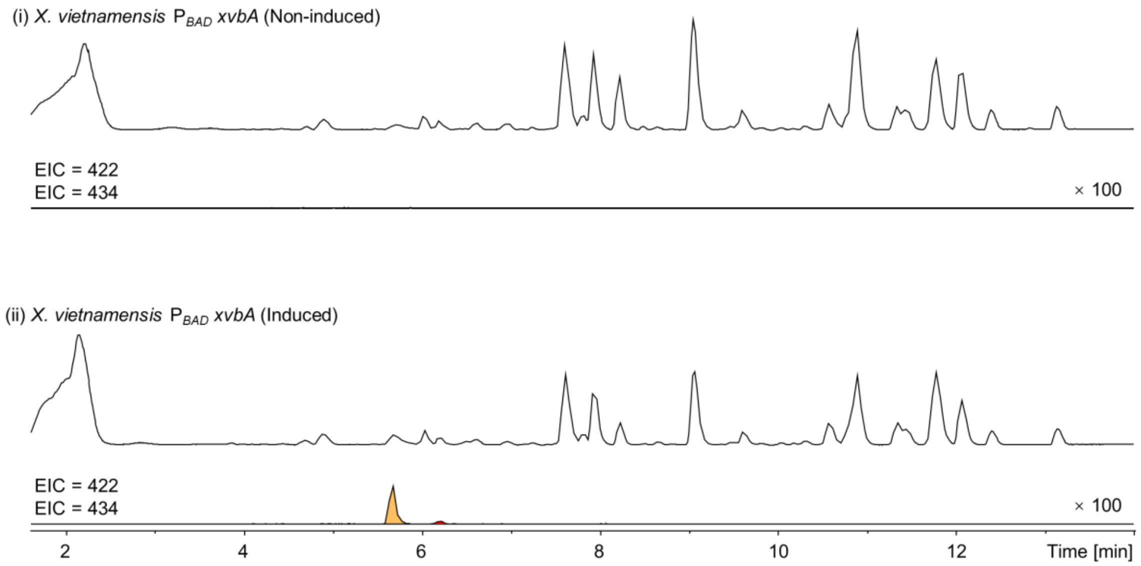

Supplementary Fig. 23 | HPLC-MS analysis of benzobactins in the promoter exchange mutants of *X. vietnamensis* DSM 22392 in XPP medium. (i) BPCs and EICs of the non-induced mutant. (ii) BPCs and EICs of the induced mutant. Desired peaks are highlighted in trace (ii). Benzobactin A (**28**, orange) and the methyl ester thereof (**29**, red) are highlighted in the EICs. Intensities in EIC traces (i) and (ii) are magnified for visualizing tiny peaks. Magnifications are indicated on the right side of traces. Mutants were induced with L-arabinose. Representative data from three independent experiments are shown.

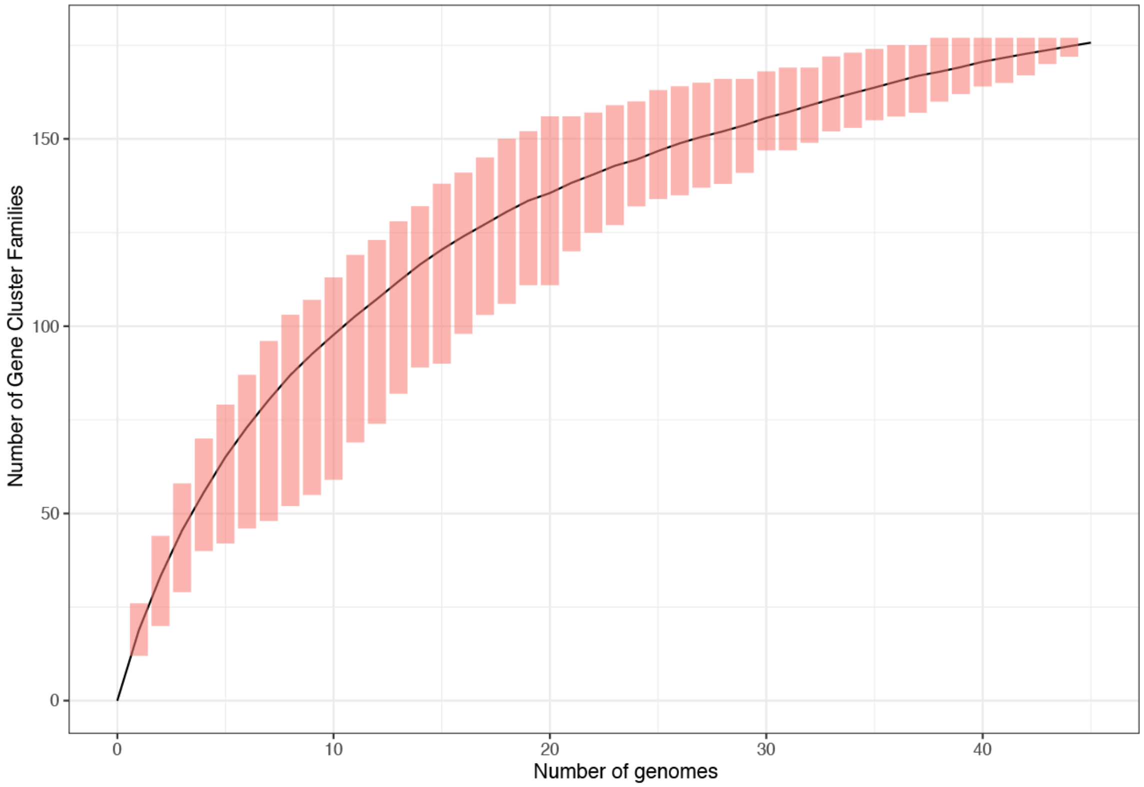

Supplementary Fig. 24 | Rarefaction analysis for the 176 GCFs in 45 *XP* strains. The line shows the mean accumulation of 100 permutations with bars indicating the maximum and minimum values over these random shuffles. A rarefaction analysis was computed in R using the rarefaction function as a part of the mircopan package with n.perm set to 100. GCFs are defined by BiG-SCAPE distance metrics with a raw distance cut-off of 0.65 and refined based on our in-house BGC database.

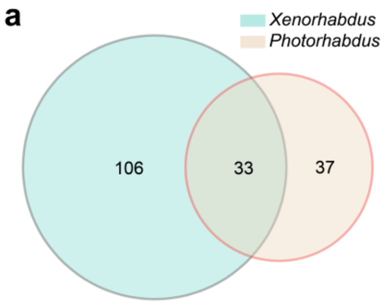

b

BGCs/GCFs	*Xenorhabdus* sum	*Xenorhabdus* mean	*Photorhabdus* sum	*Photorhabdus* mean
BGCs	604	20.8	396	24.8
unknown BGCs	340	11.7	195	12.2
GCFs	139	4.8	70	4.4
genus-specific GCFs	106	3.7	37	2.3
unique genus-specific GCFs*	40	1.4	16	1.0
unique genus-specific isolated clade GCFs*	17	0.6	8	0.5
singletons	23	0.8	8	0.5

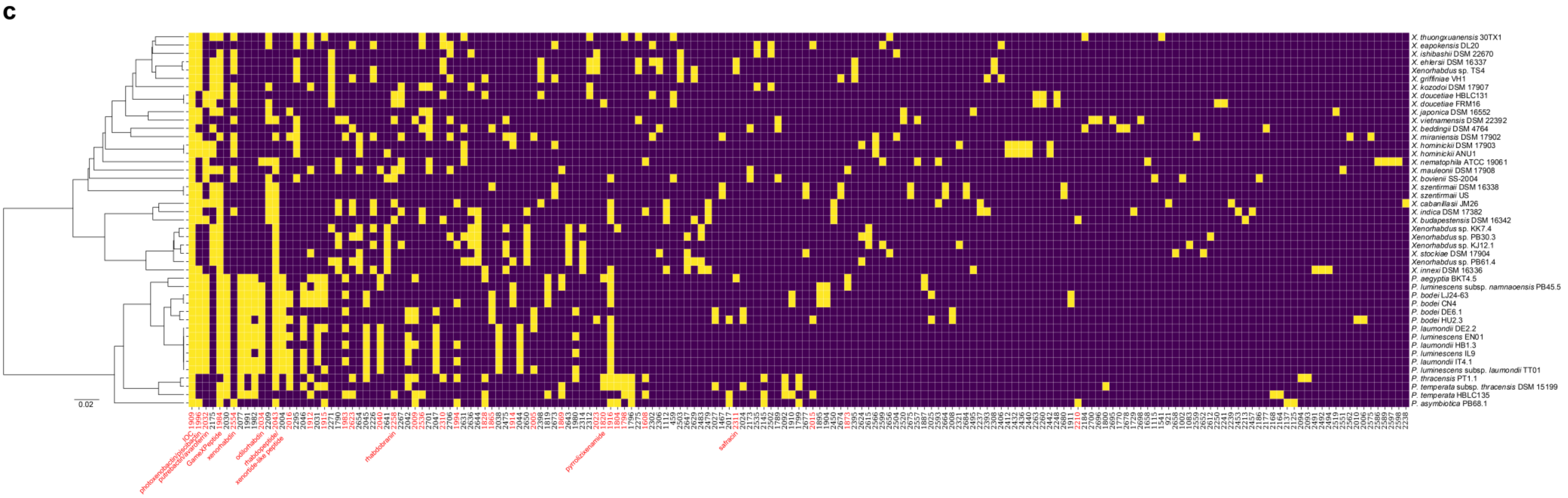

Supplementary Fig. 25 | Distributions of GCFs on genus and species levels. a, Venn diagram on the distribution of GCFs between *XP* genera. **b,** Distribution of BGCs and GCFs in *Xenorhabdus* and/or *Photorhabdus*. Asterisk indicates BGCs within a given GCF having connections with neither MIBiG reference BGCs nor the main BiG-SCAPE network. **c,** Phyletic distribution of GCFs in *XP*. A yellow square indicates the presence of a GCF in the respective species. GCFs shared by *XP* are highlighted in red and compound annotations are indicated for those having been identified. GCFs are defined by BiG-SCAPE distance metrics with a raw distance cut-off of 0.65 and refined based on our in-house BGC database.

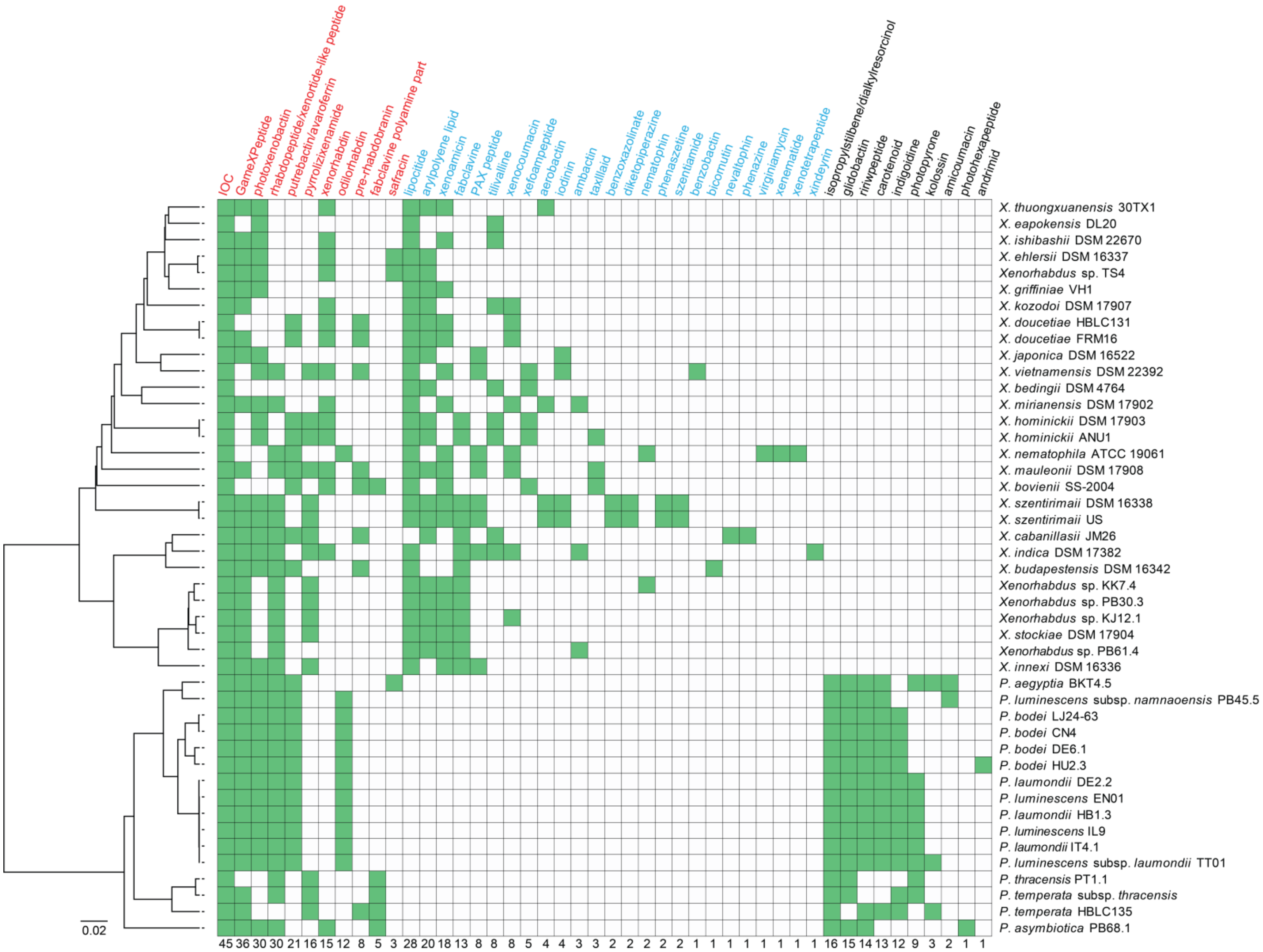
IOC
GameXPeptide
photoxenobactin
rhabdopeptide/xenortide-like peptide
putrebactin/avaroferrin
pyrrolizixenamide
xenorhabdin
odilorhabdin
pre-rhabdobranin
fabclavine polyamine part
safracin
lipocitide
arylpolyene lipid
xenoamicin
fabclavine
PAX peptide
tilivalline
xenocoumacin
xefoampeptide
aerobactin
iodinin
ambactin
taxllaid
benzoxazolinate
diketopiperazine
nematophin
phenazetine
szentiamide
benzobactin
bicornutin
nevaltophin
phenazine
virginiamycin
xenematide
xenotetrapeptide
xindeyrin
isopropylstilbene/dialkylresorcinol
glidobactin
rinwpeptide
carotenoid
indigoidine
photopyrone
kolossin
amicoumacin
photohexapeptide
andrimid
X. thuongxuanensis 30TX1
X. eapokensis DL20
X. ishibashii DSM 22670
X. ehlersii DSM 16337
Xenorhabdus sp. TS4
X. griffiniae VH1
X. kozodoi DSM 17907
X. doucetiae HBLC131
X. doucetiae FRM16
X. japonica DSM 16522
X. vietnamensis DSM 22392
X. bedingii DSM 4764
X. mirianensis DSM 17902
X. hominickii DSM 17903
X. hominickii ANU1
X. nematophila ATCC 19061
X. mauleonii DSM 17908
X. bovienii SS-2004
X. szentirimaii DSM 16338
X. szentirimaii US
X. cabanillasii JM26
X. indica DSM 17382
X. budapestensis DSM 16342
Xenorhabdus sp. KK7.4
Xenorhabdus sp. PB30.3
Xenorhabdus sp. KJ12.1
X. stockiae DSM 17904
Xenorhabdus sp. PB61.4
X. innexi DSM 16336
P. aegyptia BKT4.5
P. luminescens subsp. namnaoensis PB45.5
P. bodei LJ24-63
P. bodei CN4
P. bodei DE6.1
P. bodei HU2.3
P. laumondii DE2.2
P. luminescens EN01
P. laumondii HB1.3
P. luminescens IL9
P. laumondii IT4.1
P. luminescens subsp. laumondii TT01
P. thracensis PT1.1
P. temperata subsp. thracensis
P. temperata HBLC135
P. asymbiotica PB68.1
0.02
45 36 30 30 21 16 15 12 8 5 3 28 20 18 13 8 8 8 5 4 4 3 3 2 2 2 2 2 1 1 1 1 1 1 1 1 16 15 14 13 12 9 3 2 1 1

Supplementary Fig. 26 | Phyletic distribution of *XP* BGCs. BGCs are limited to those that are previously known and identified in this study. A green square indicates the presence of a BGC in the respective species. BGCs shared by *XP* are in red. *Xenorhabdus*-specific BGCs are in blue. *Photorhabdus*-specific BGCs are in black. The number at the bottom of the matrix indicates the sum of each BGC from 45 *XP* genomes. Coincidently, the most prevalent BGCs sorted by chemical classes are still in line with the most prevalent BGCs sorted by numbers. If we sorted the BGCs by numbers, IOC, GameXPeptide, and photoxenobactin BGCs are still the top three among the *XP* BGCs. Lipocitide, arylpolyene lipid, and fabclavine BGCs are among the top four in *Xenorhabdus*-specific BGCs. Isopropylstilbene, glidobactin, ririwpeptide, and carotenoid BGCs are still the top four in *Photorhabdus*-specific BGCs. *Xenorhabdus* BGCs are more discrete than those in *Photorhabdus*. This also indicates that *Photorhabdus* is more conserved among different species.

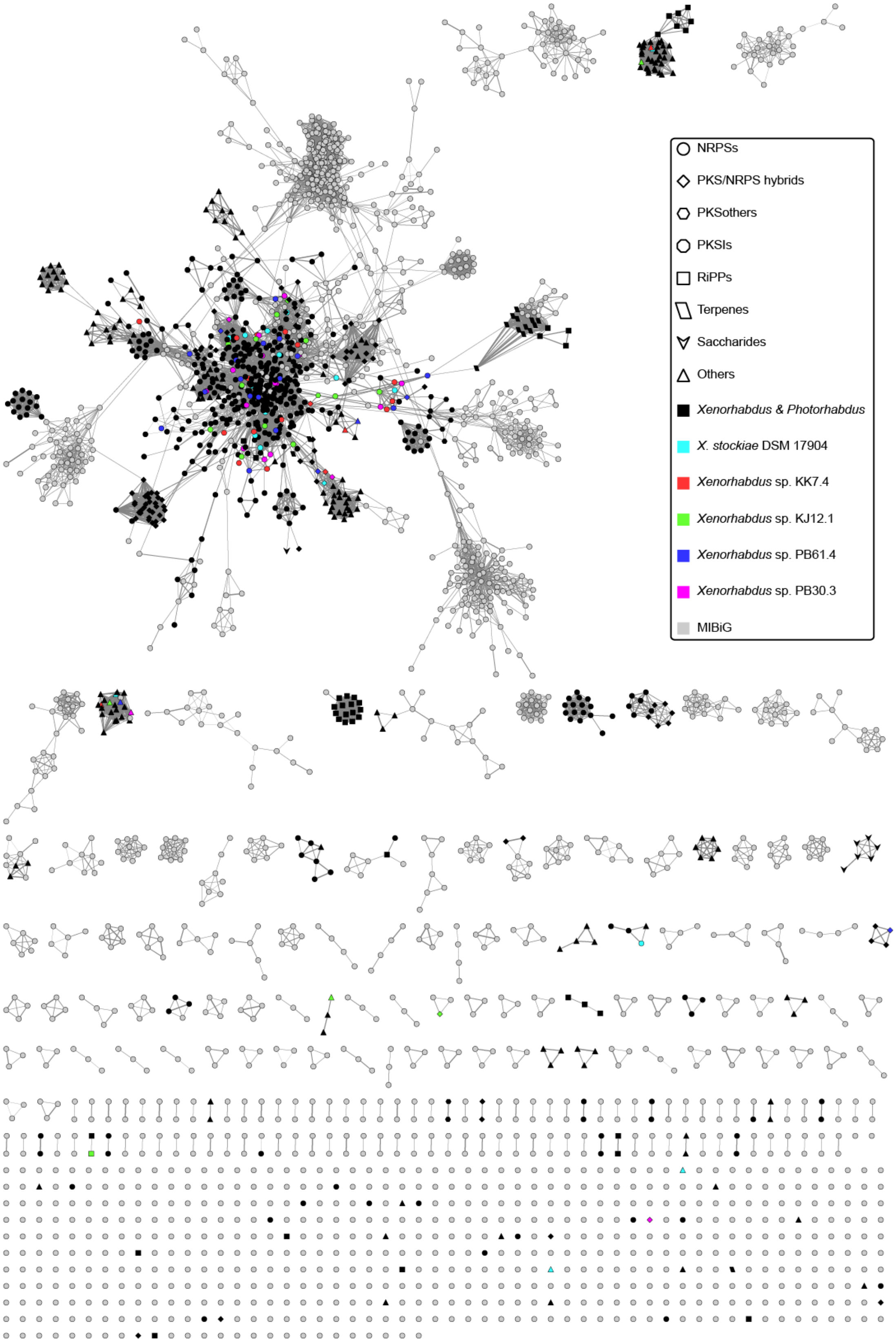

Supplementary Fig. 27 | BGC distribution of *Xenorhabdus stockiae* DMS 17904, *Xenorhabdus* sp. KK7.4, *Xenorhabdus* sp. KJ12.1, *Xenorhabdus* sp. PB30.3, and *Xenorhabdus* sp. PB61.4 in the sequence similarity network.

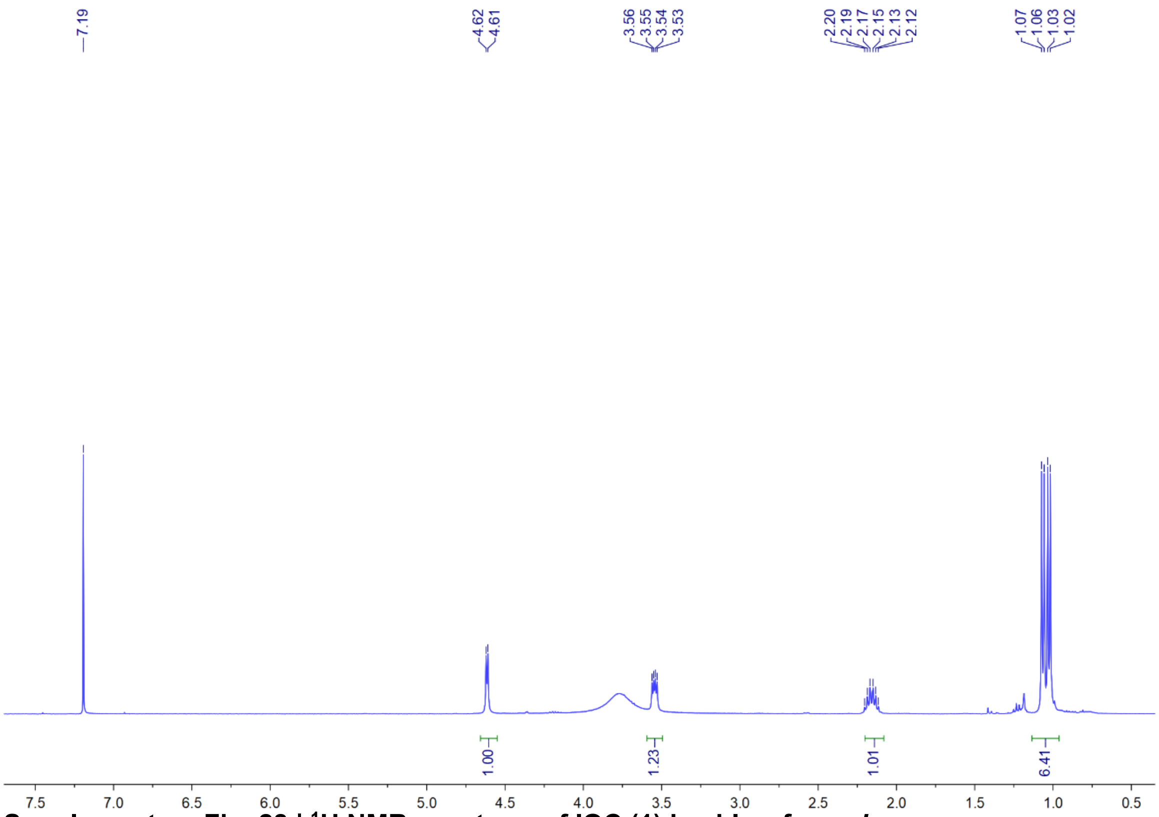

Supplementary Fig. 28 | ^{1}H NMR spectrum of IOC (1) in chloroform-*d*.

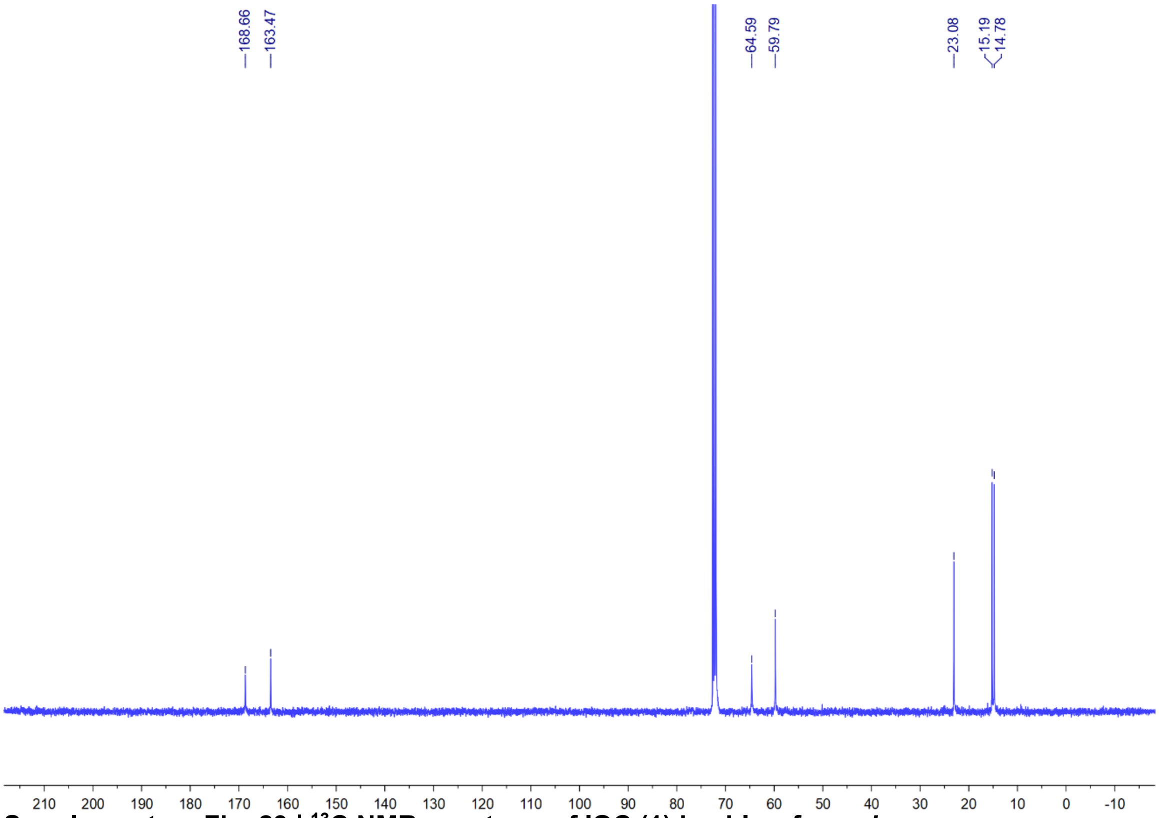

Supplementary Fig. 29 | ^{13}C NMR spectrum of IOC (1) in chloroform-*d*.

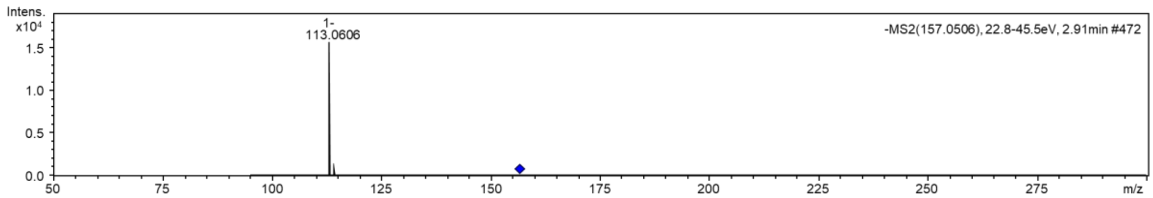

Supplementary Fig. 30 | HR-ESI-MS of IOC (1).

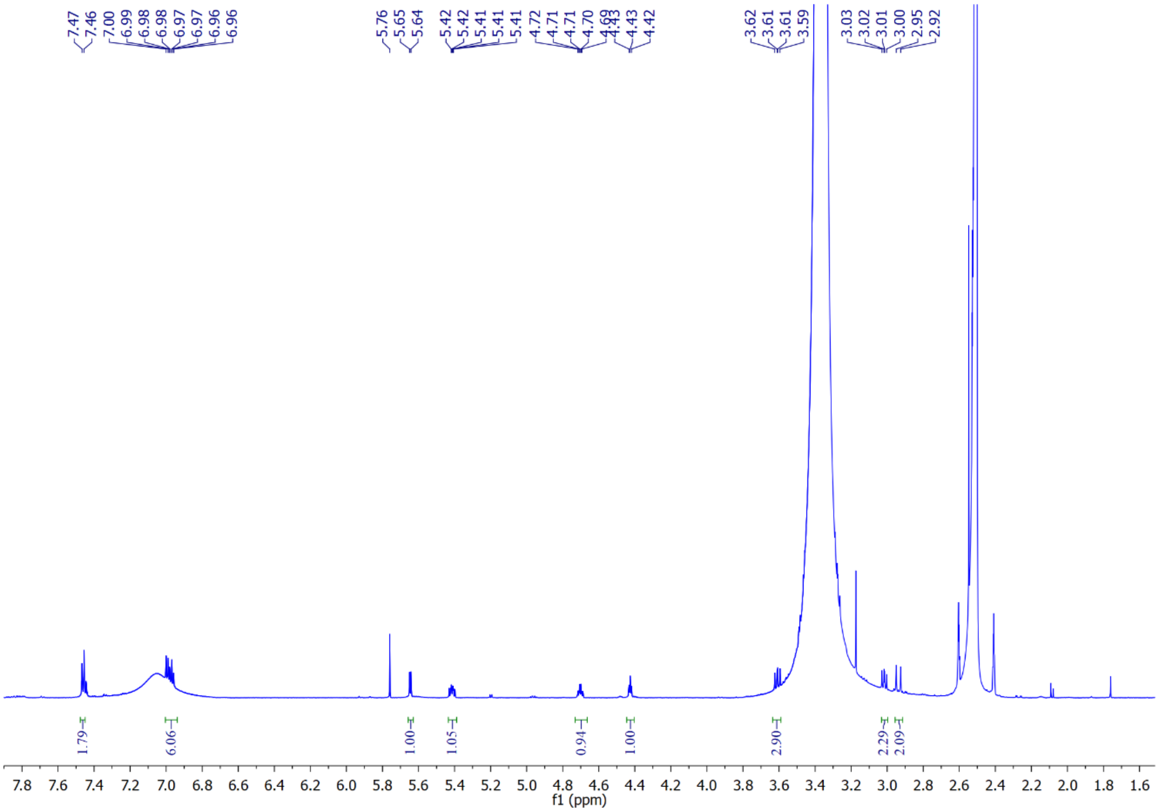

Supplementary Fig. 31 | ^{1}H NMR spectrum of photoxenobactin A (4) in DMSO-d_6.

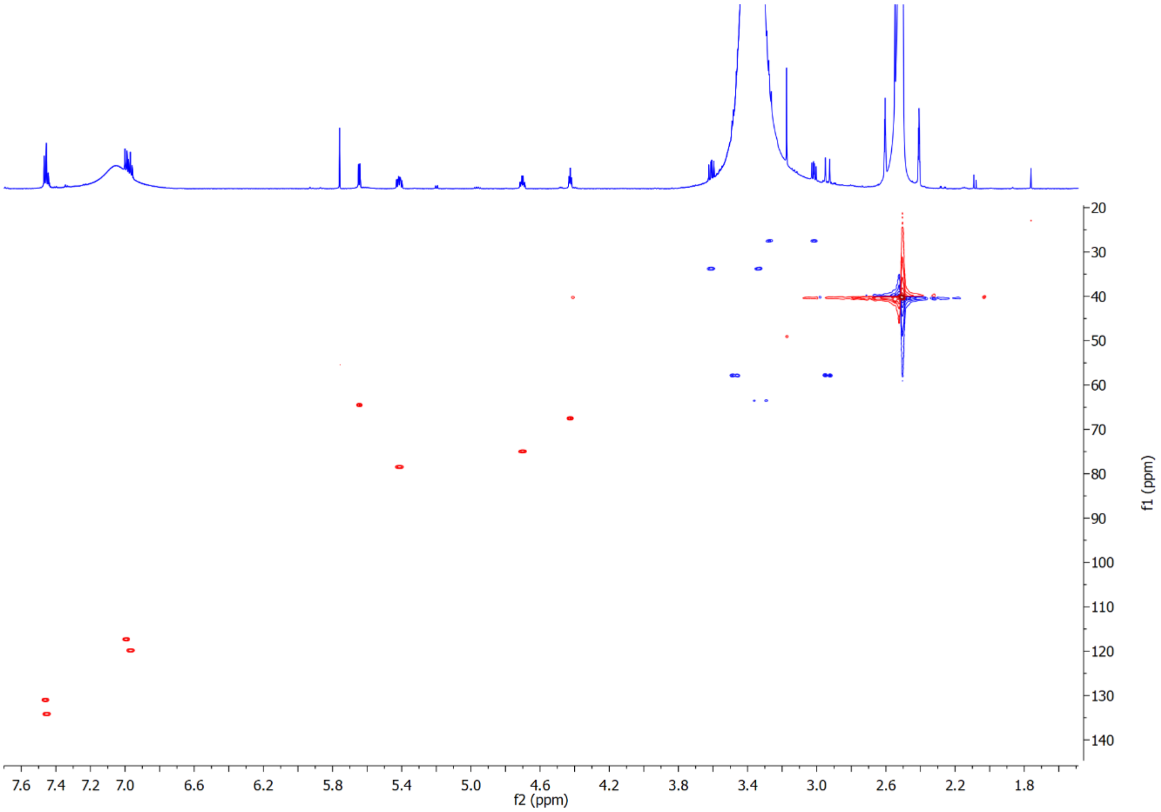

Supplementary Fig. 32 | HSQC spectrum of photoxenobactin A (4) in DMSO-d_6.

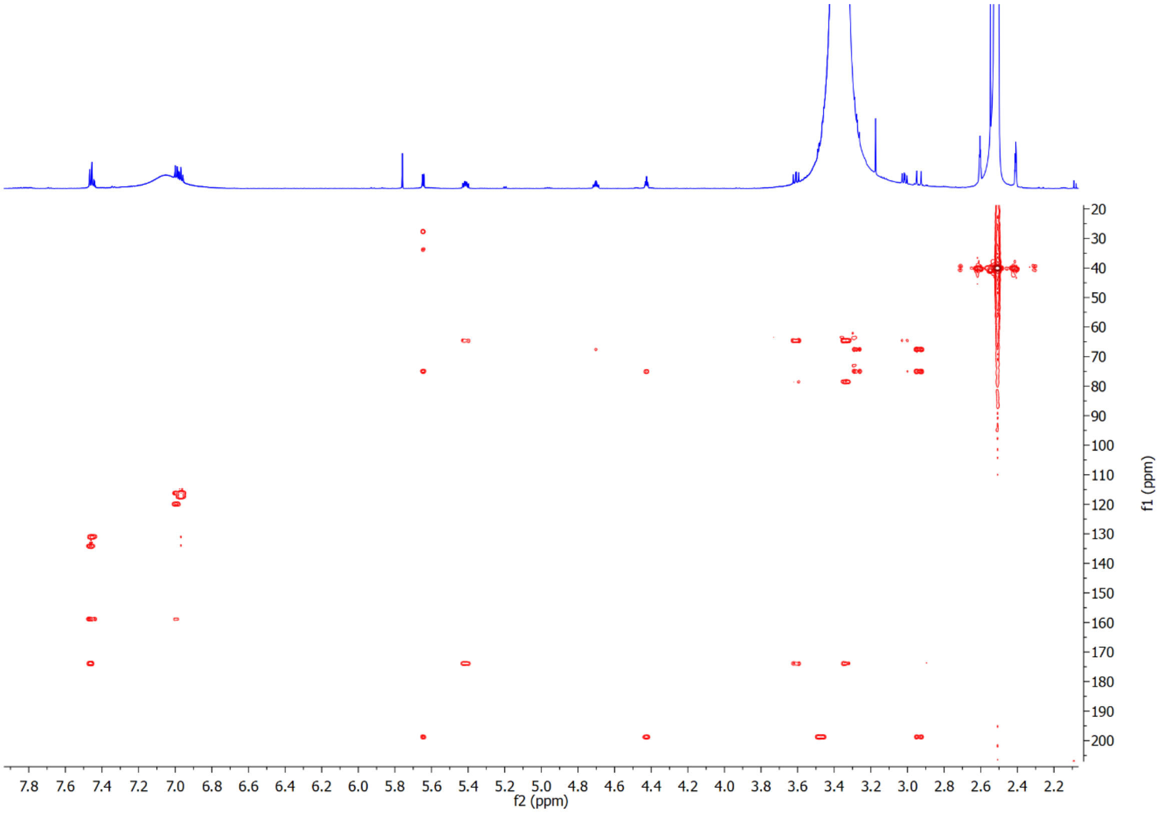

Supplementary Fig. 33 | HMBC spectrum of photoxenobactin A (4) in DMSO-d_6.

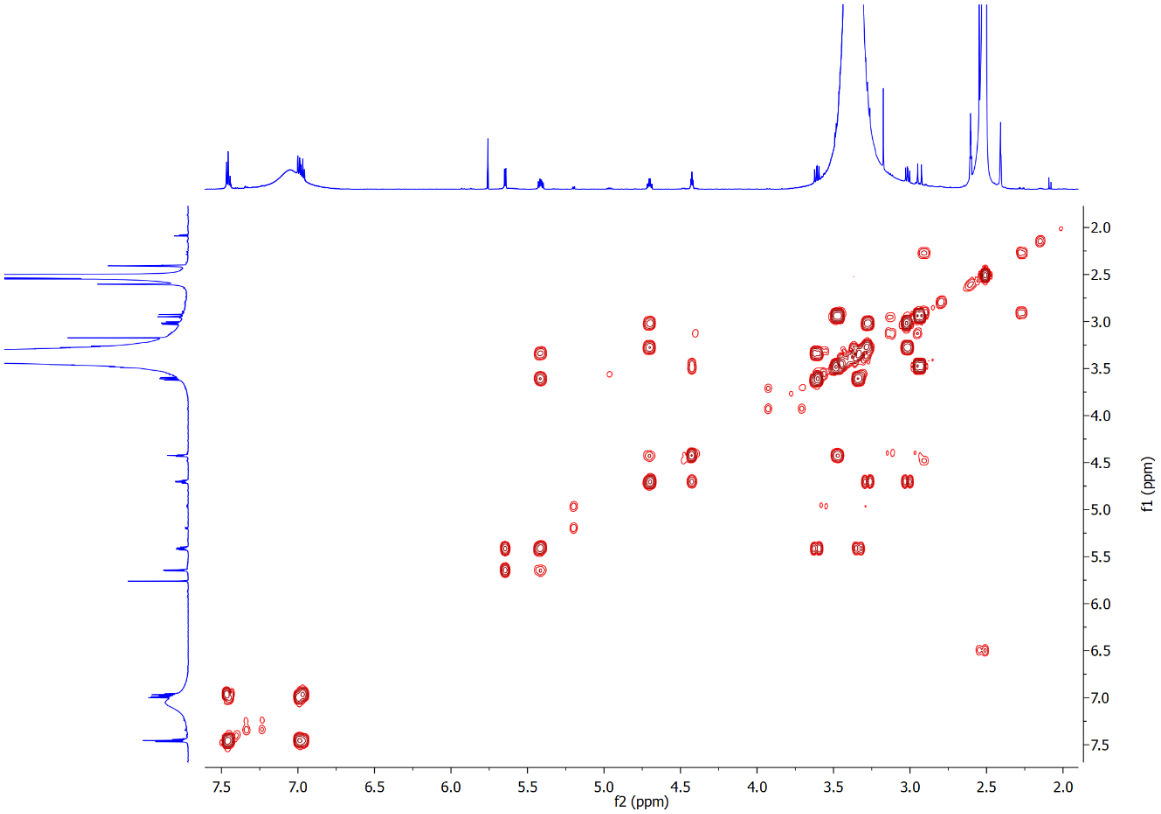

Supplementary Fig. 34 | ^{1}H-^{1}H COSY spectrum of photoxenobactin A (4) in DMSO-d_6.

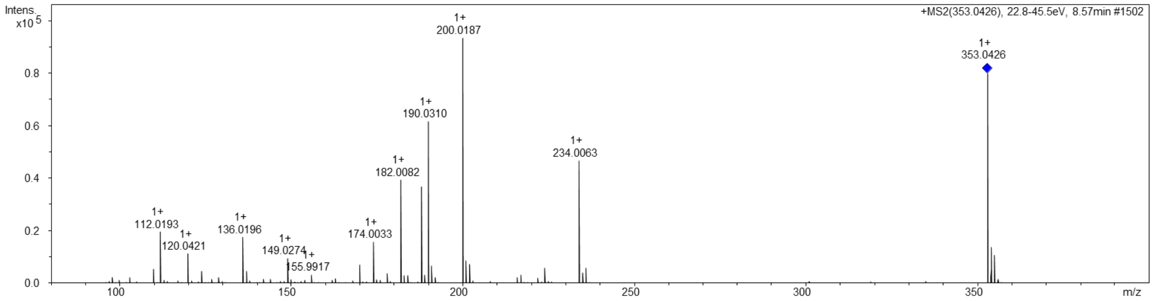

Supplementary Fig. 35 | HR-ESI-MS of photoxenobactin A (4).

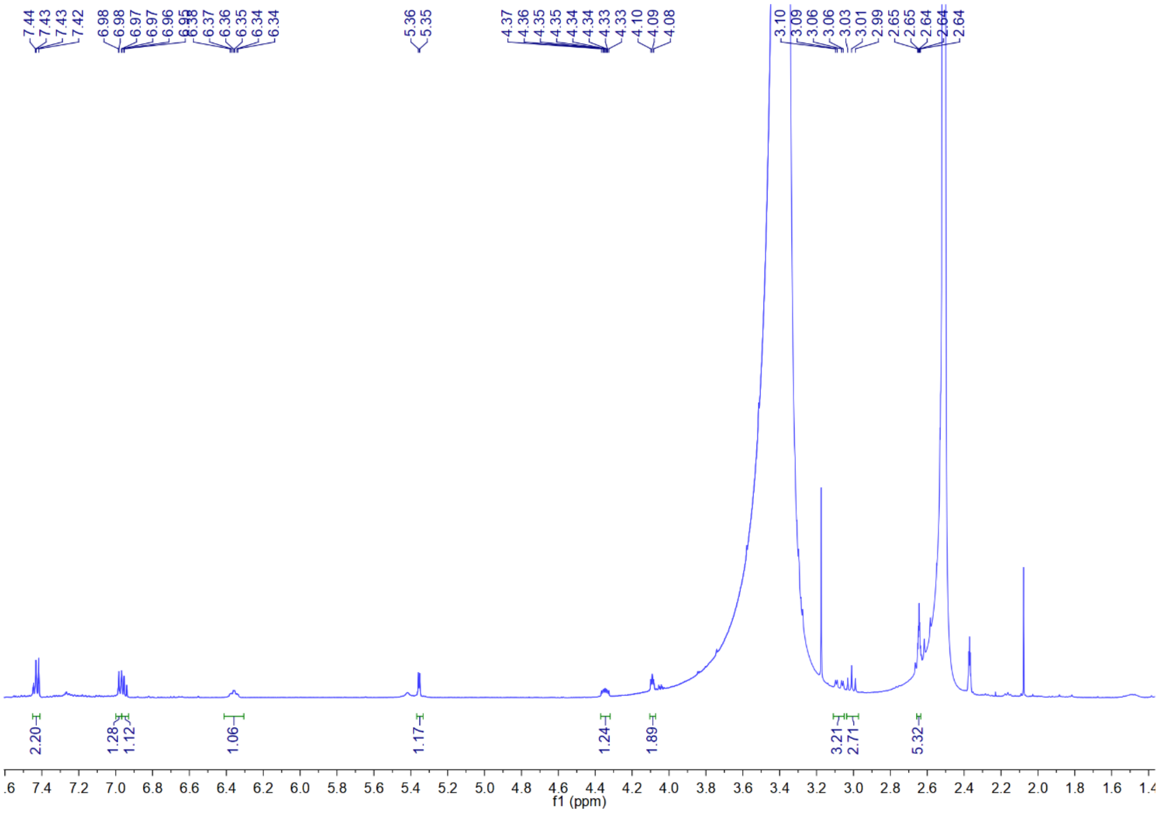

Supplementary Fig. 36 | ^{1}H NMR spectrum of photoxenobactin B (5) in DMSO-*d*$_6$.

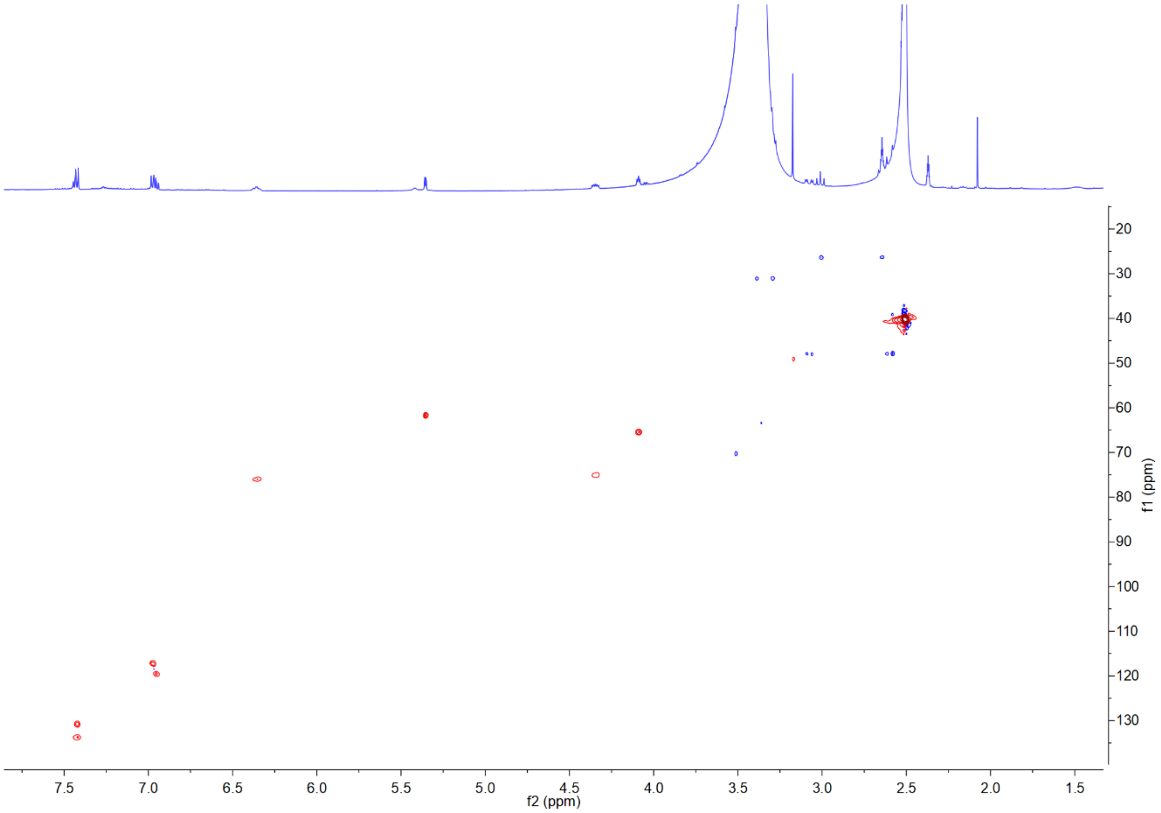

Supplementary Fig. 37 | HSQC spectrum of photoxenobactin B (5) in DMSO-*d*$_6$.

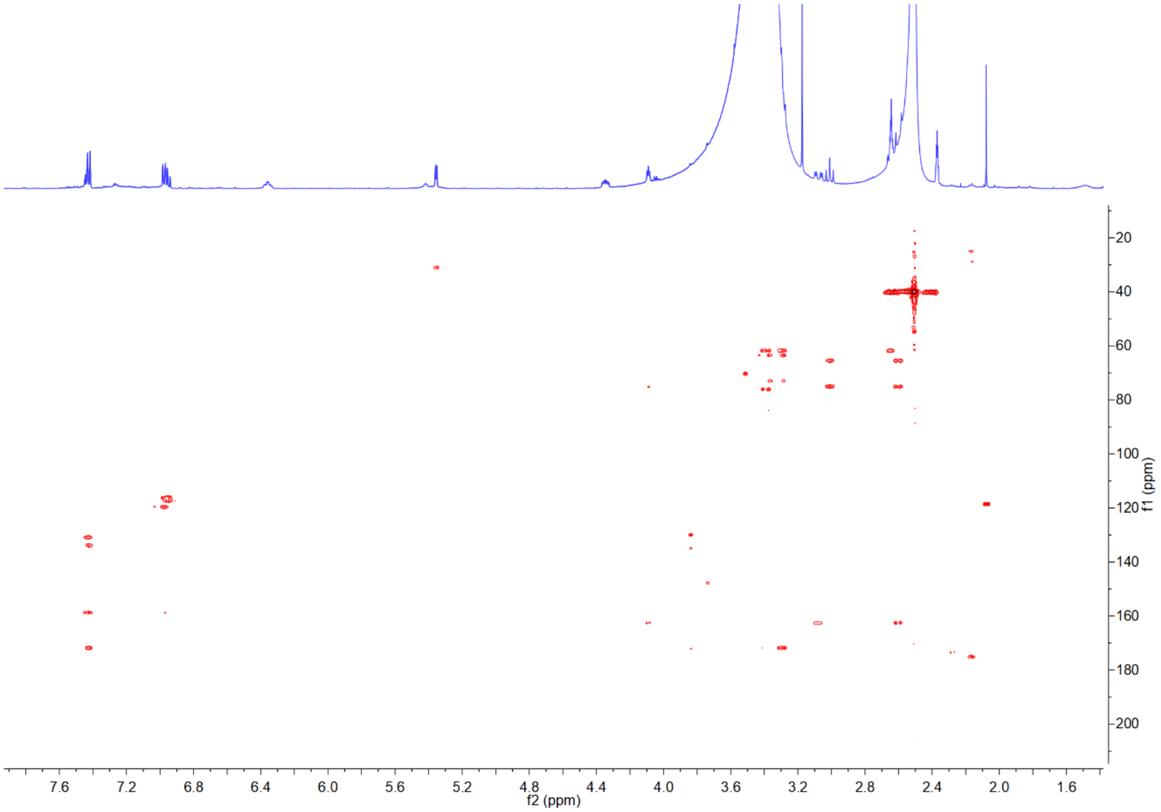

Supplementary Fig. 38 | HMBC spectrum of photoxenobactin B (5) in DMSO-*d*6.

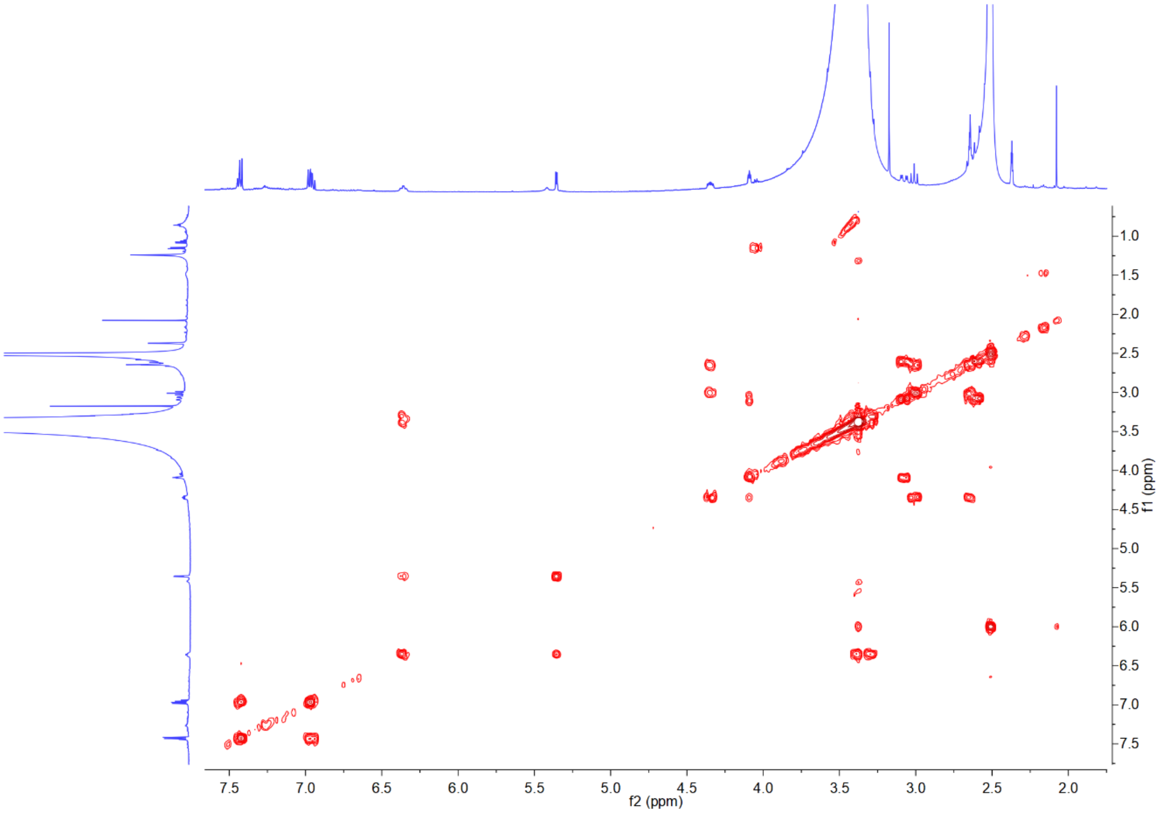

Supplementary Fig. 39 | ^{1}H-^{1}H COSY spectrum of photoxenobactin B (5) in DMSO-*d*6.

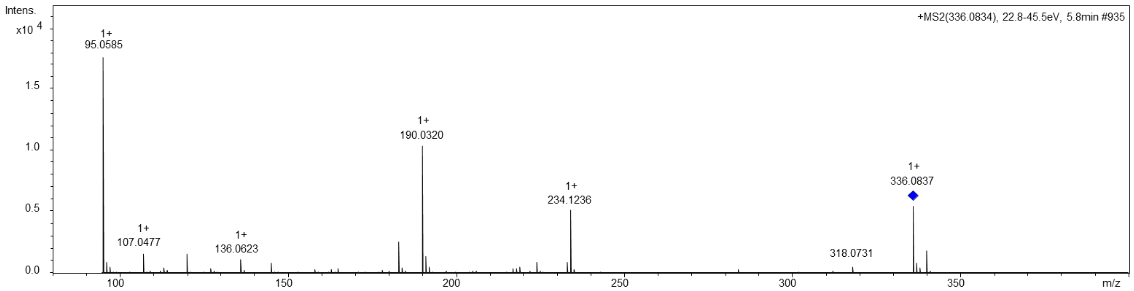

Supplementary Fig. 40 | HR-ESI-MS of photoxenobactin B (5).

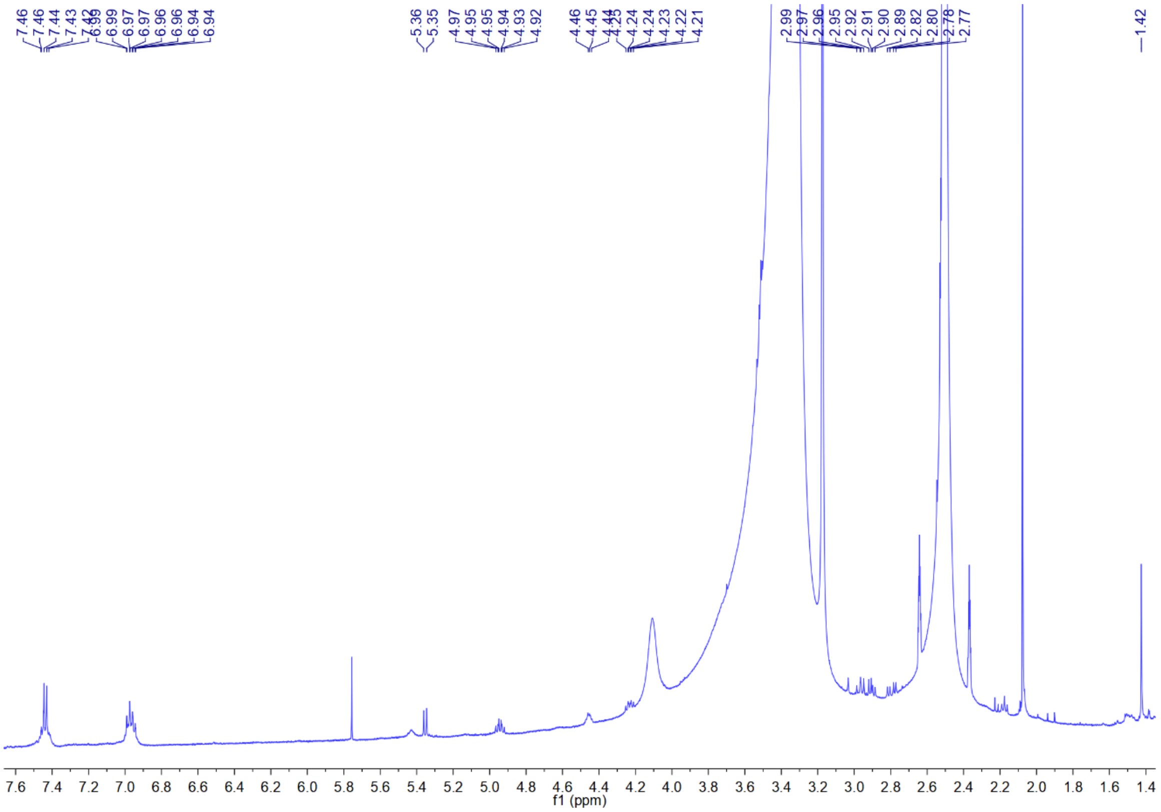

Supplementary Fig. 41 | ^{1}H NMR spectrum of photoxenobactin C (6) in DMSO-d_6.

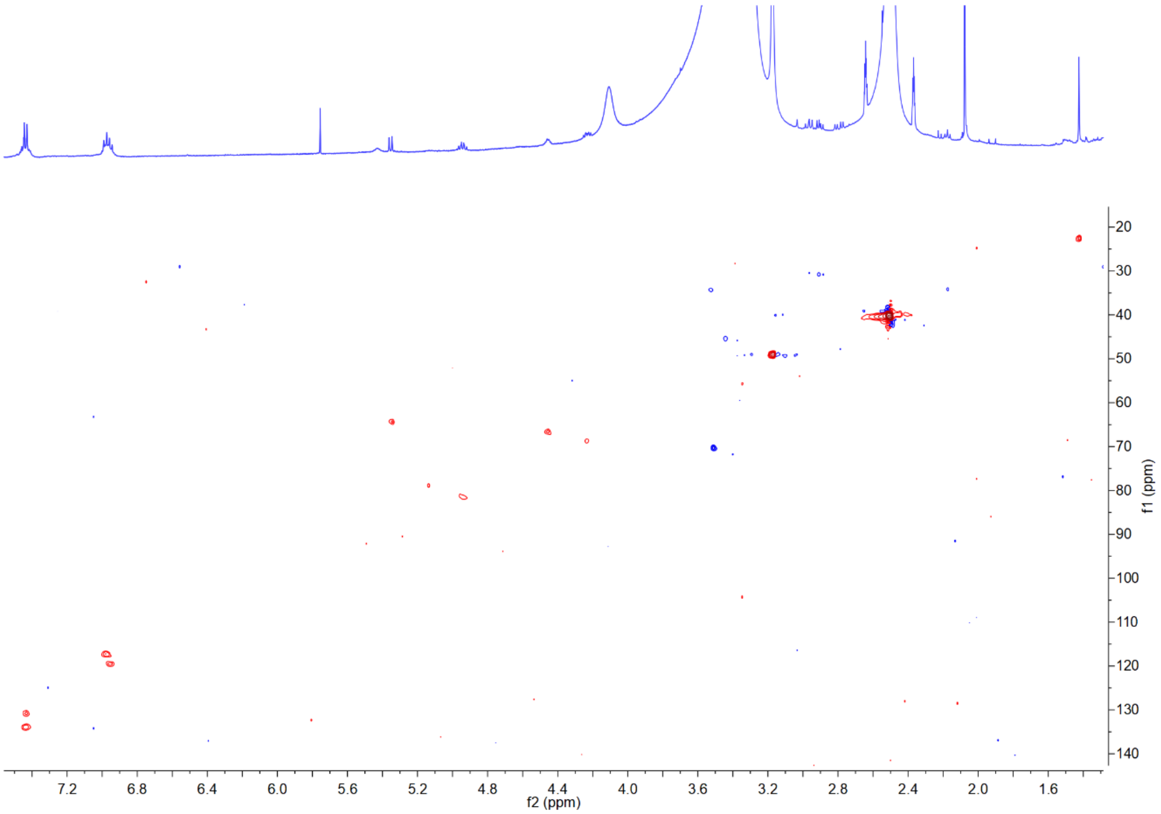

Supplementary Fig. 42 | HSQC spectrum of photoxenobactin C (6) in DMSO-d_6.

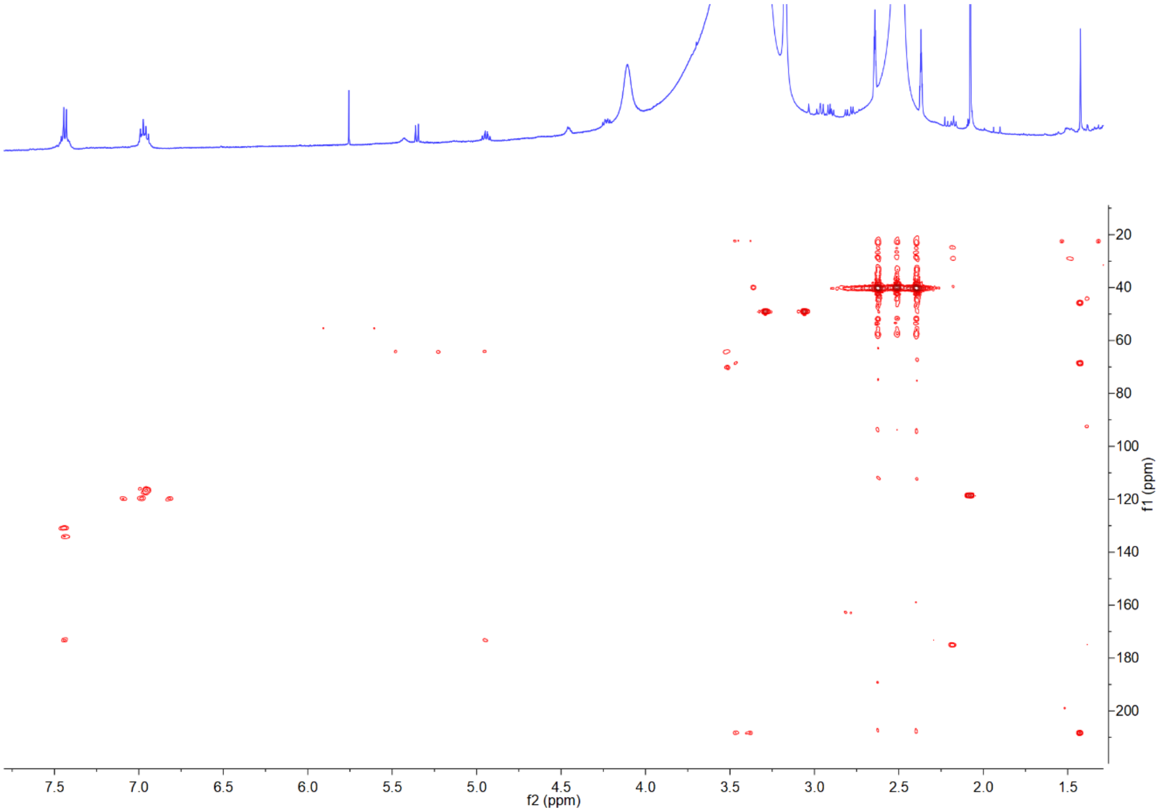

Supplementary Fig. 43 | HMBC spectrum of photoxenobactin C (6) in DMSO-*d*6.

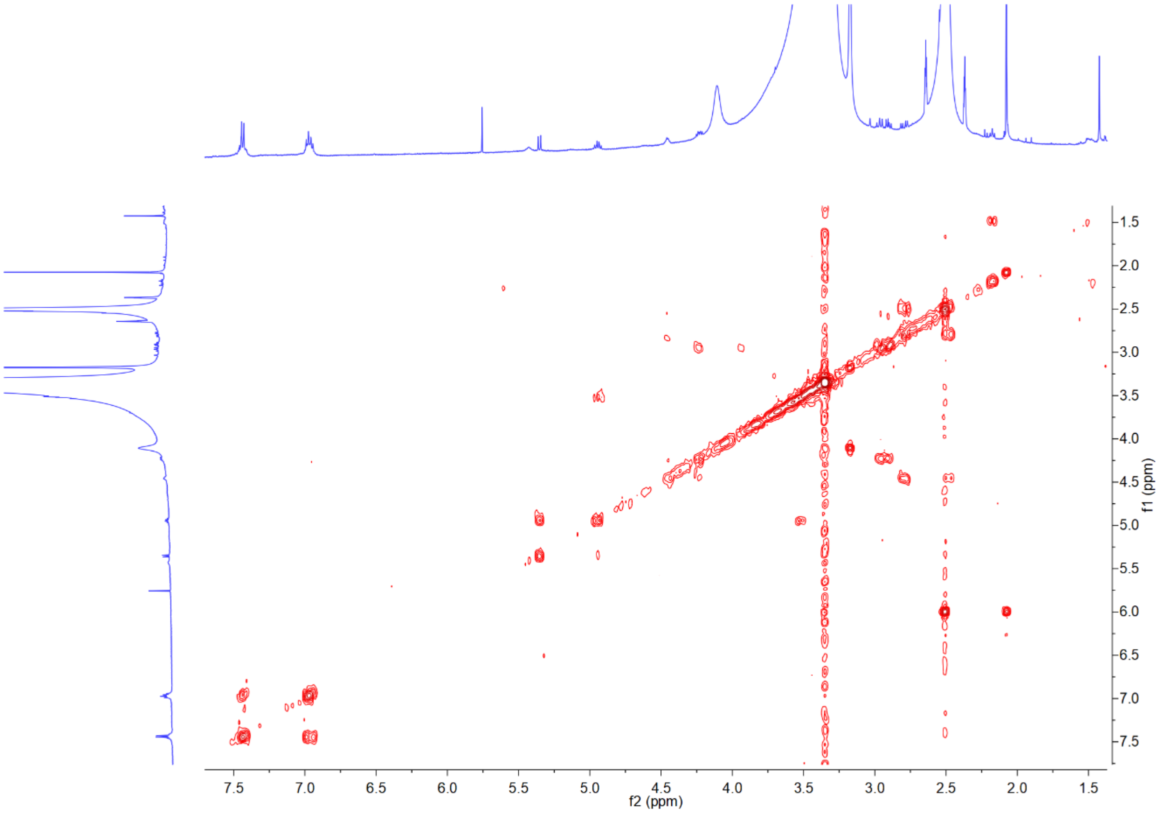

Supplementary Fig. 44 | ^{1}H-^{1}H COSY spectrum of photoxenobactin C (6) in DMSO-*d*6.

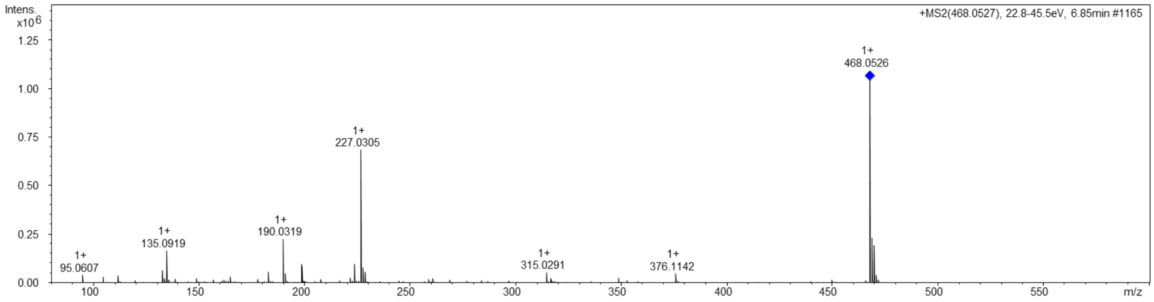

Supplementary Fig. 45 | HR-ESI-MS of photoxenobactin C (6).

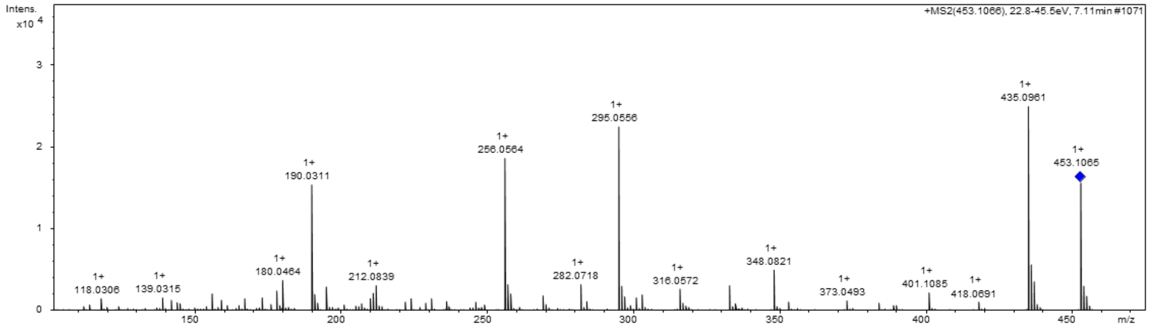

Supplementary Fig. 46 | HR-ESI-MS of photoxenobactin D (7).

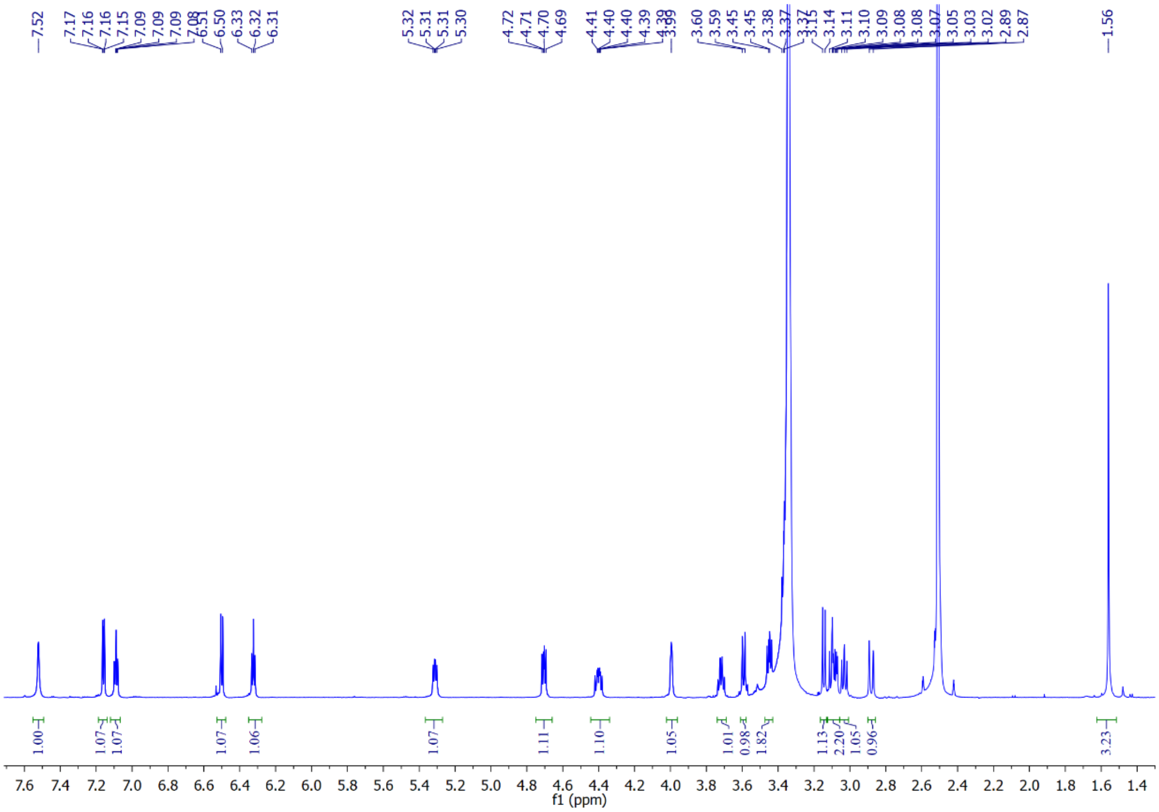

Supplementary Fig. 47 | ^{1}H NMR spectrum of photoxenobactin E (8) in DMSO-d_6.

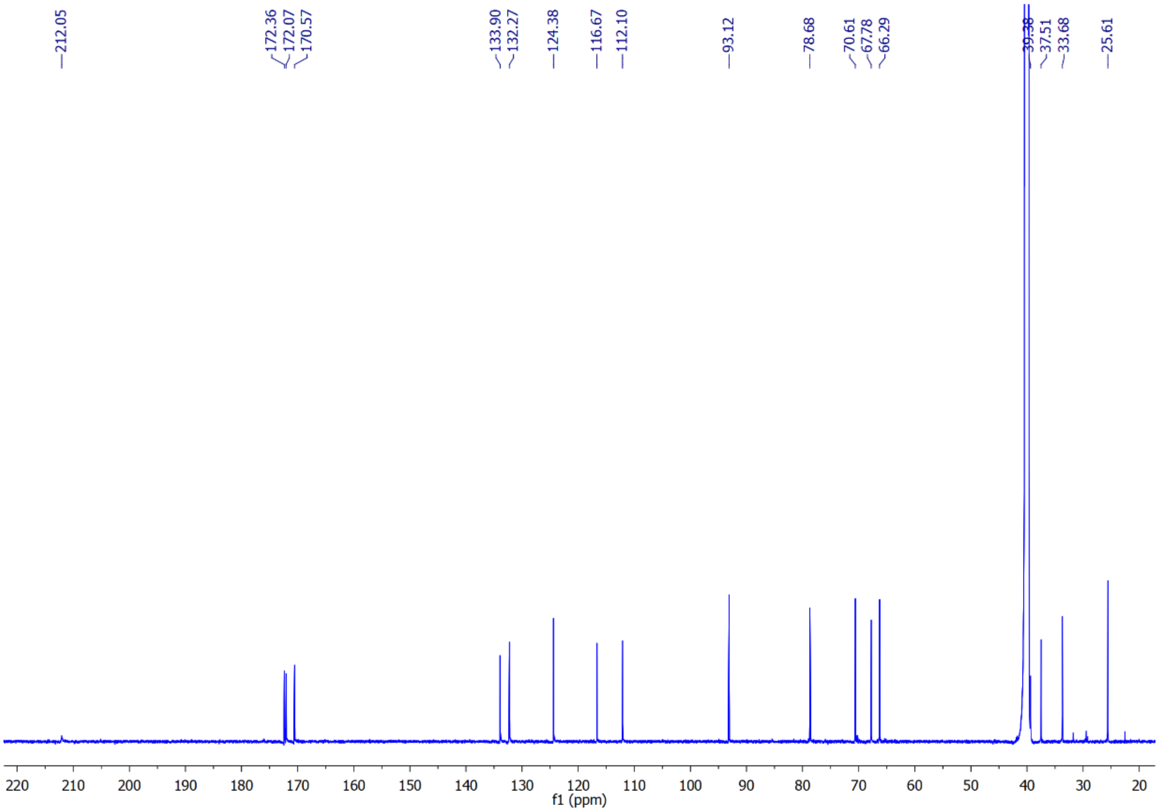

Supplementary Fig. 48 | ^{13}C NMR spectrum of photoxenobactin E (8) in DMSO-d_6.

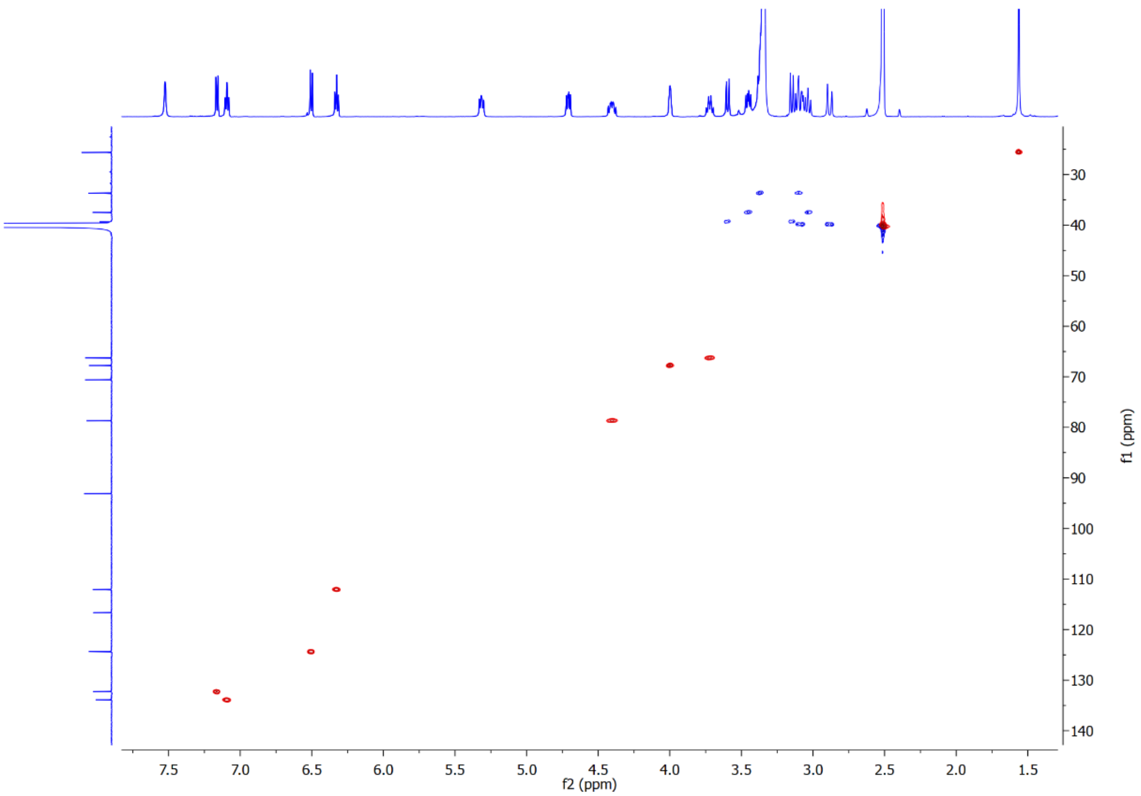

Supplementary Fig. 49 | HSQC spectrum of photoxenobactin E (8) in DMSO-*d*6.

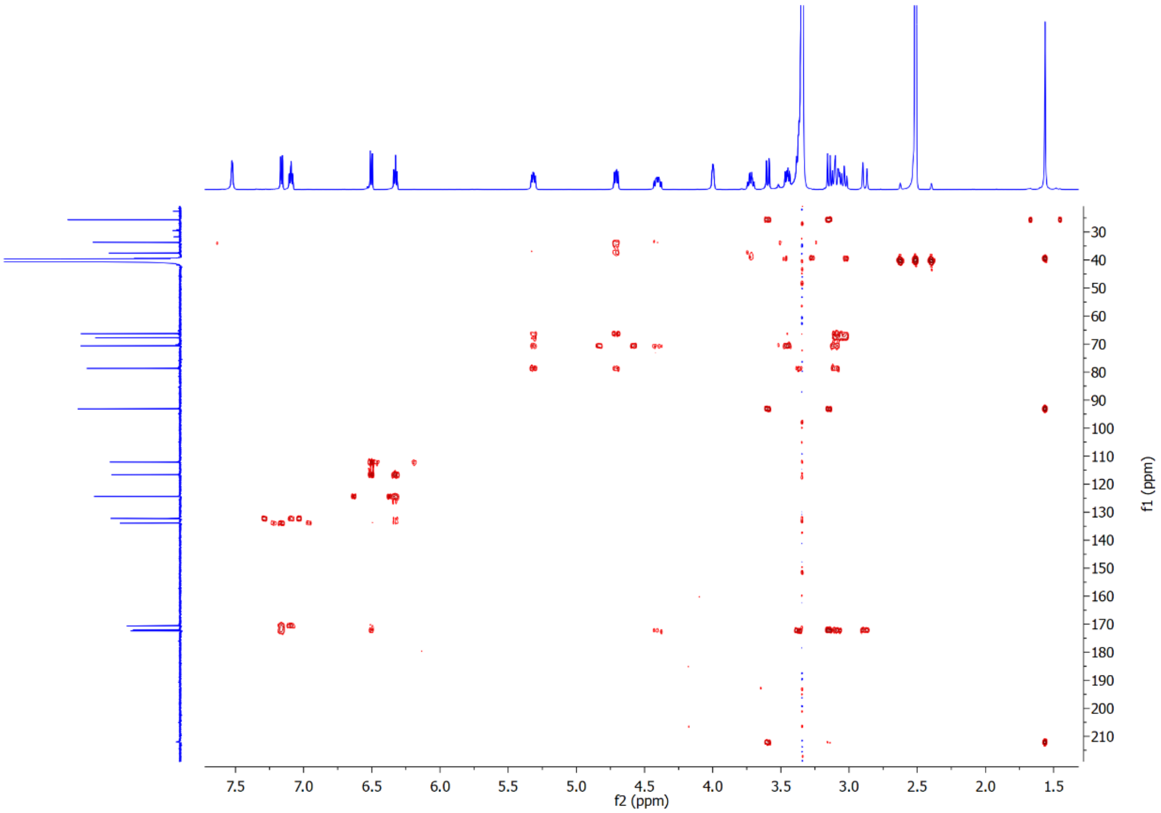

Supplementary Fig. 50 | HMBC spectrum of photoxenobactin E (8) in DMSO-*d*6.

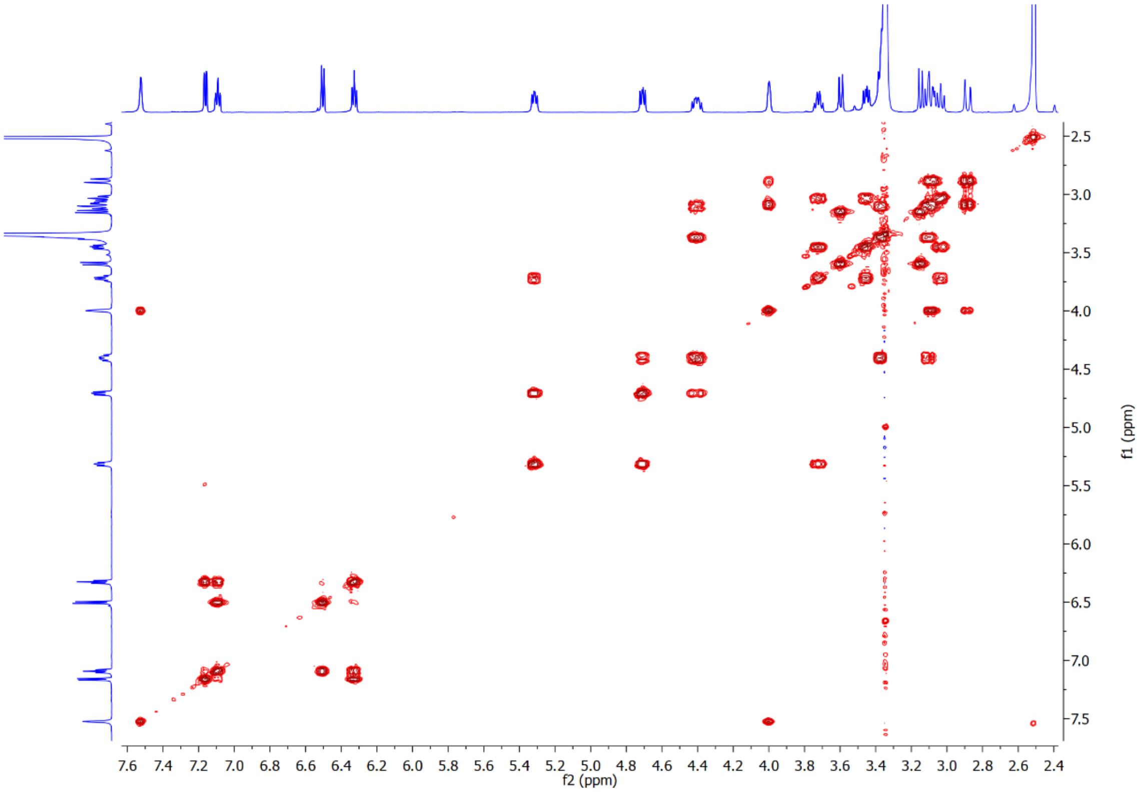

Supplementary Fig. 51 | ¹H-¹H COSY spectrum of photoxenobactin E (8) in DMSO-*d*6.

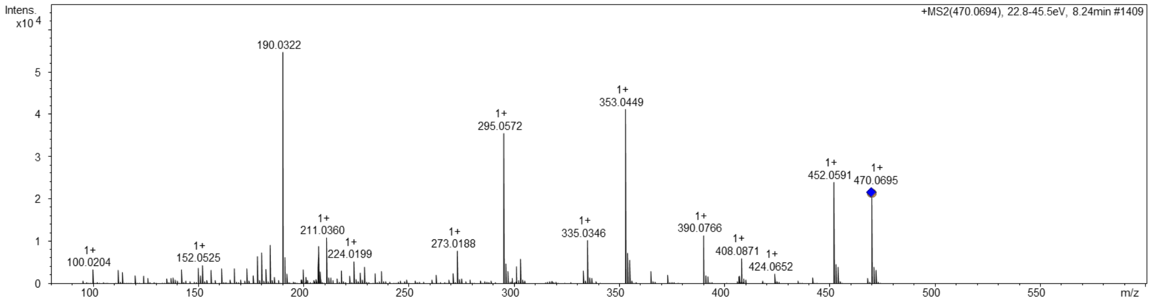

Supplementary Fig. 52 | HR-ESI-MS of photoxenobactin E (8).

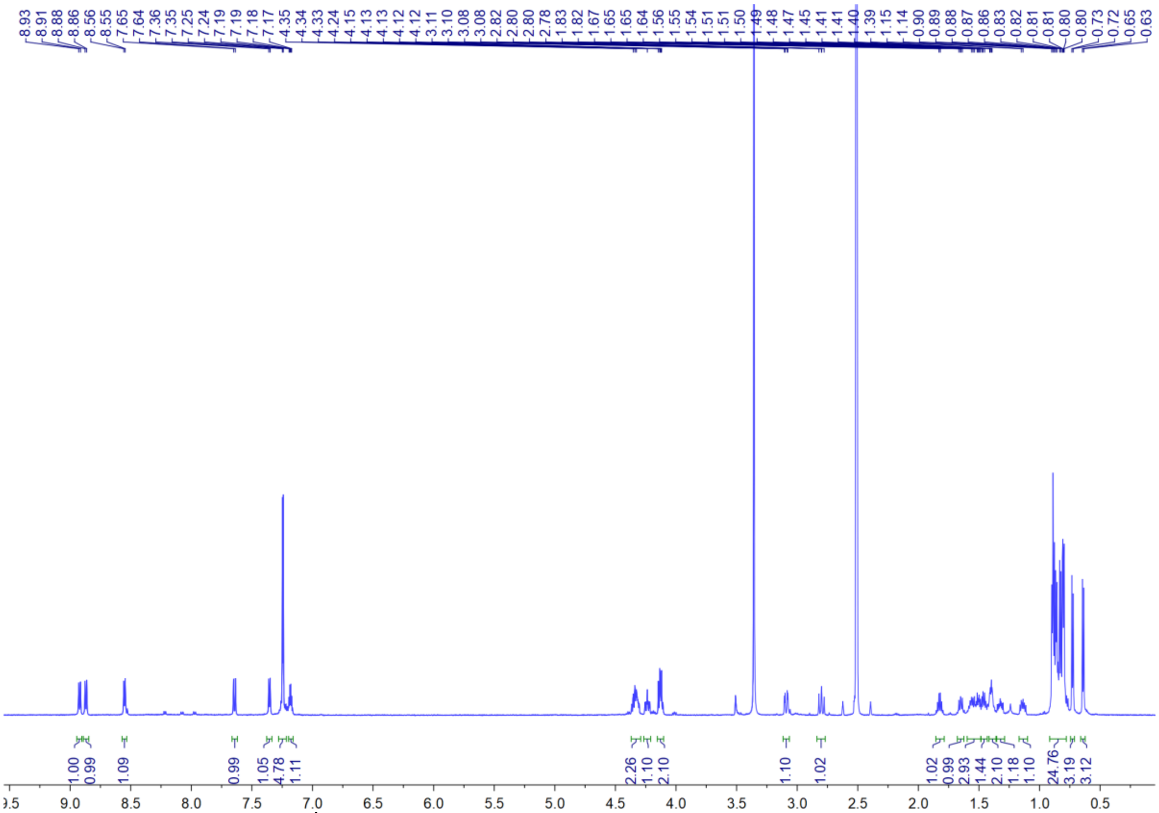

Supplementary Fig. 53 | ^{1}H NMR spectrum of GameXPeptide A (16) in DMSO-d_6.

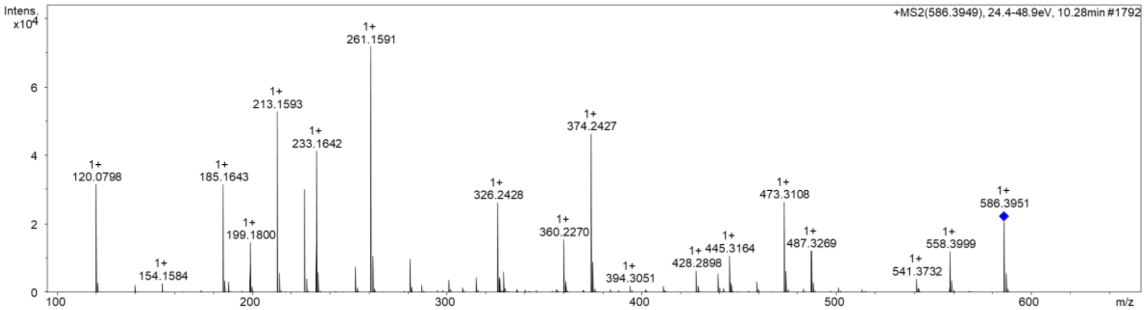

Supplementary Fig. 54 | HR-ESI-MS of GameXPeptide A (16).

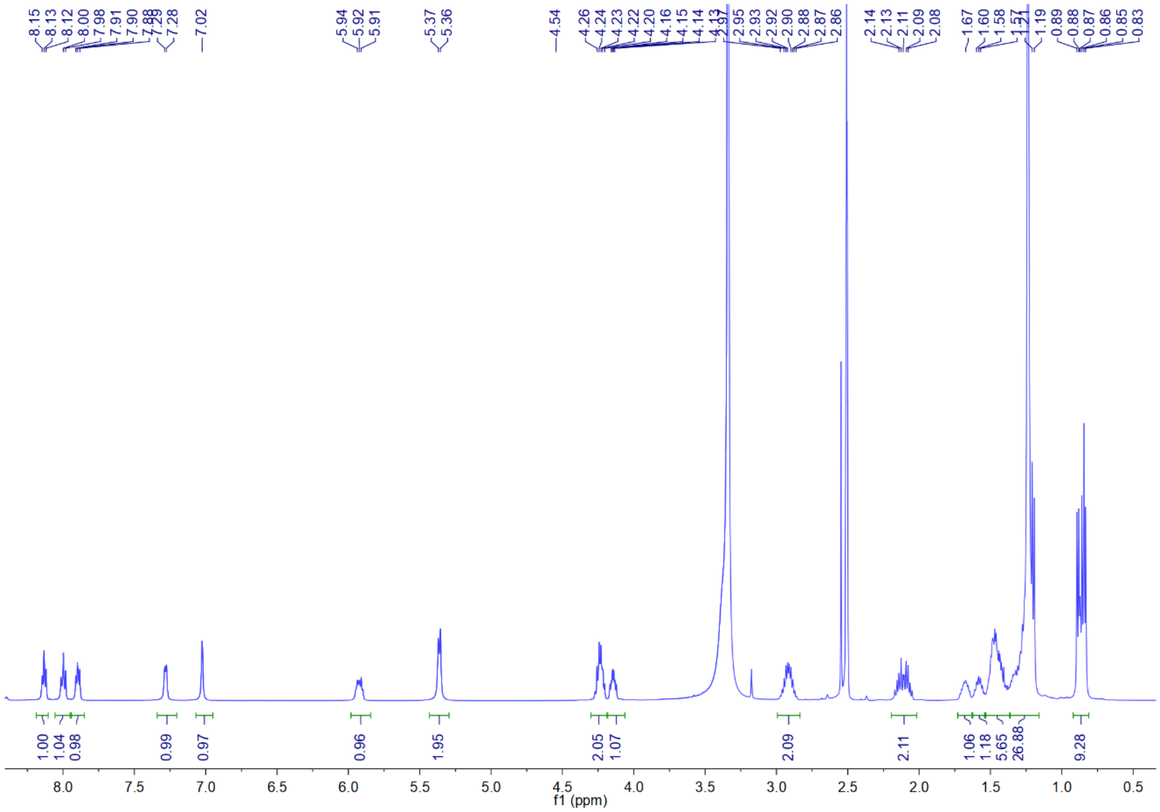

Supplementary Fig. 55 | ^{1}H NMR spectrum of lipocitide A (17) in DMSO-d_6.

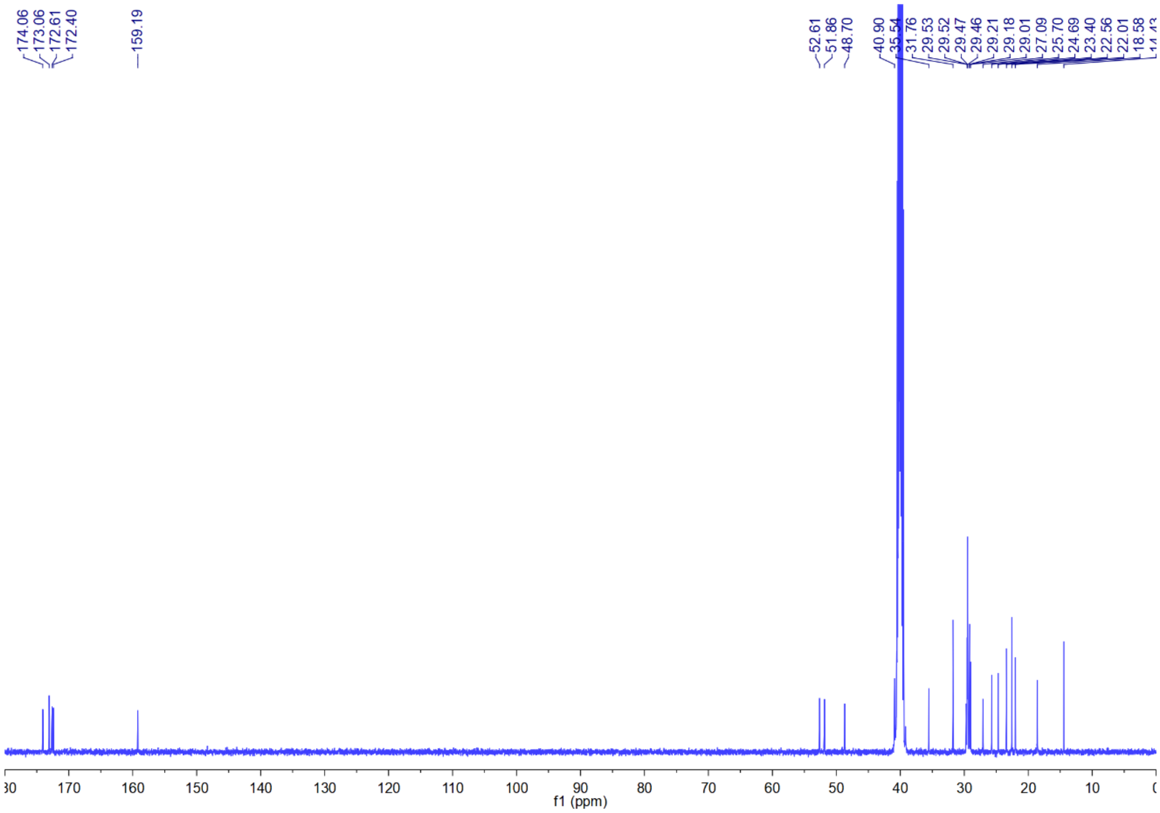

Supplementary Fig. 56 | ^{13}C NMR spectrum of lipocitide A (17) in DMSO-d_6.

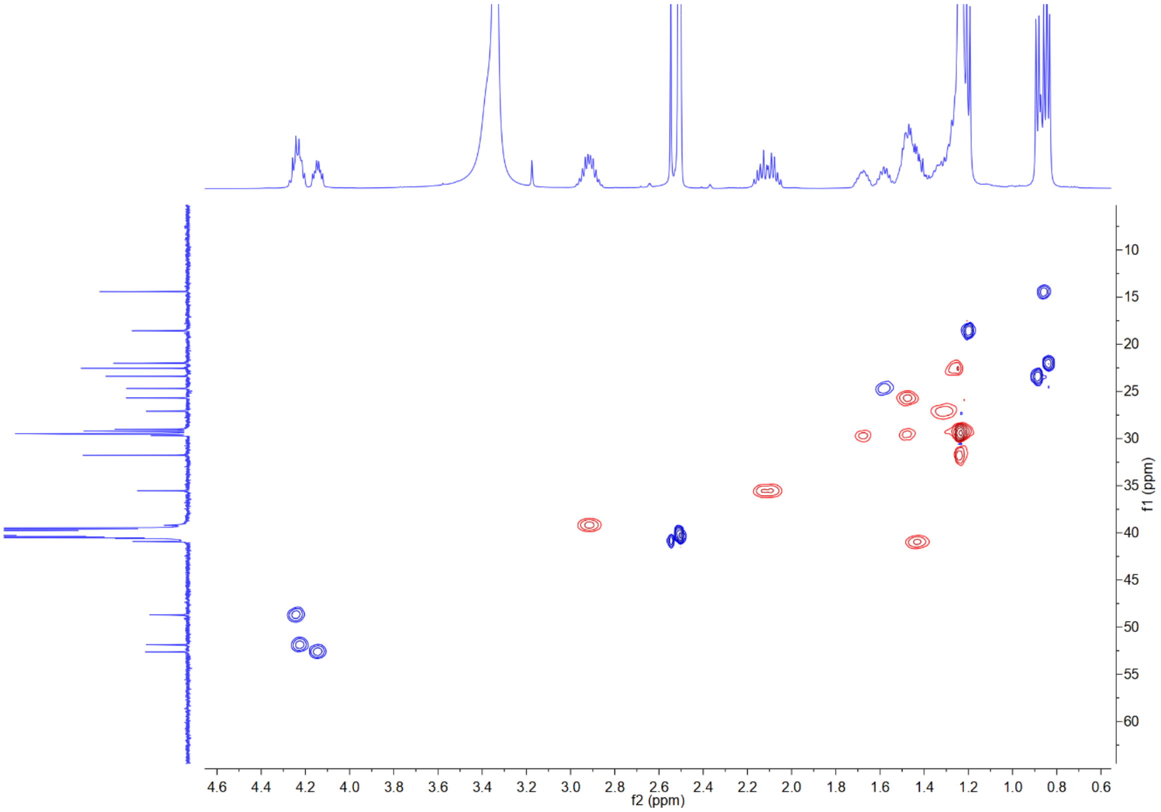

Supplementary Fig. 57 | HSQC spectrum of lipocitide A (17) in DMSO-*d*6.

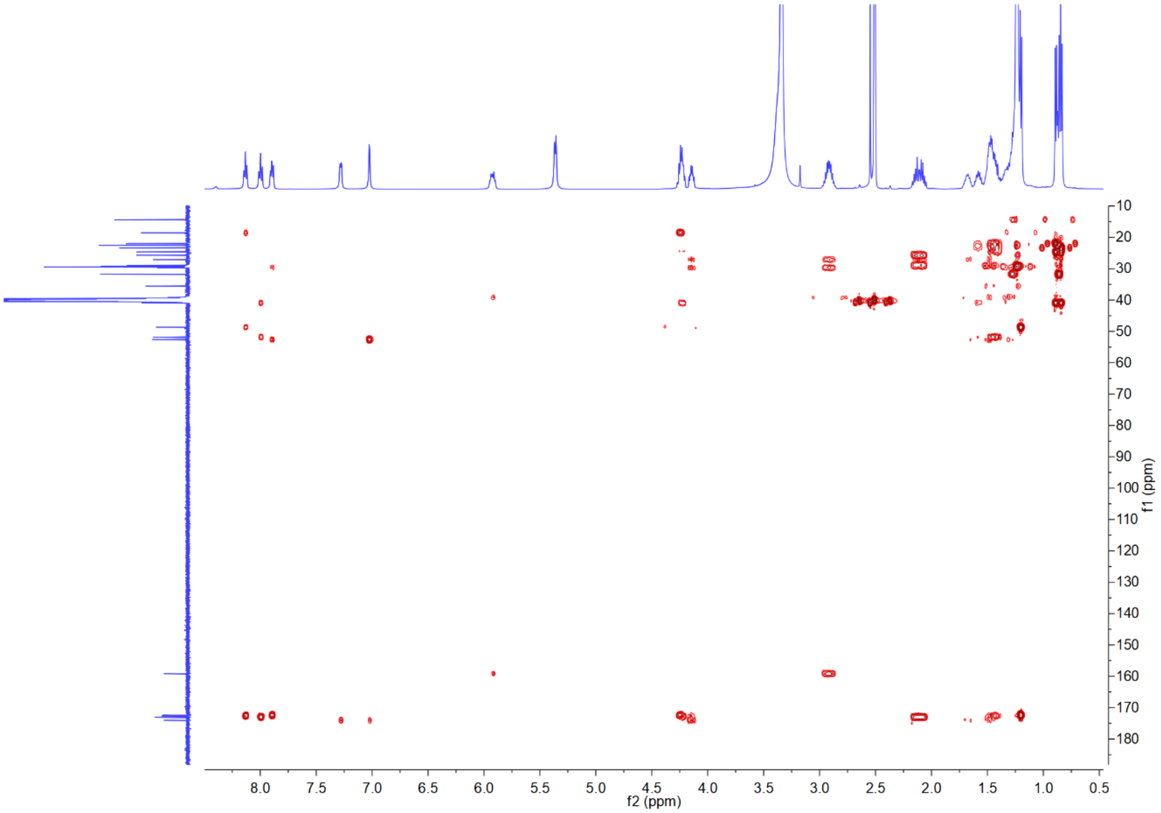

Supplementary Fig. 58 | HMBC spectrum of lipocitide A (17) in DMSO-*d*6.

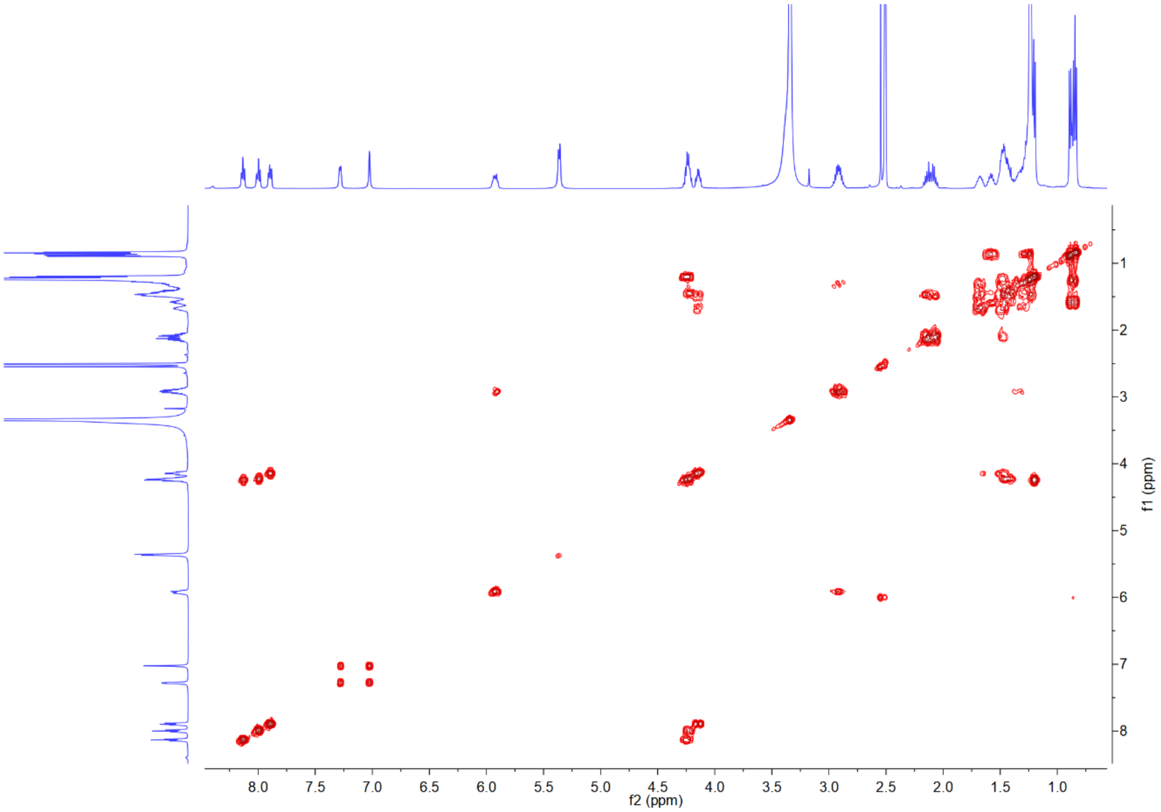

Supplementary Fig. 59 | ^1H-^1H COSY spectrum of lipocitide A (17) in DMSO-d_6.

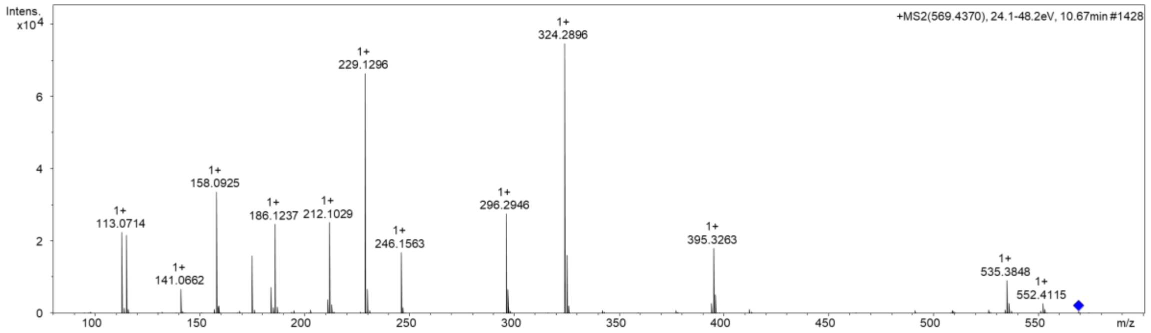

Supplementary Fig. 60 | HR-ESI-MS of lipocitide A (17).

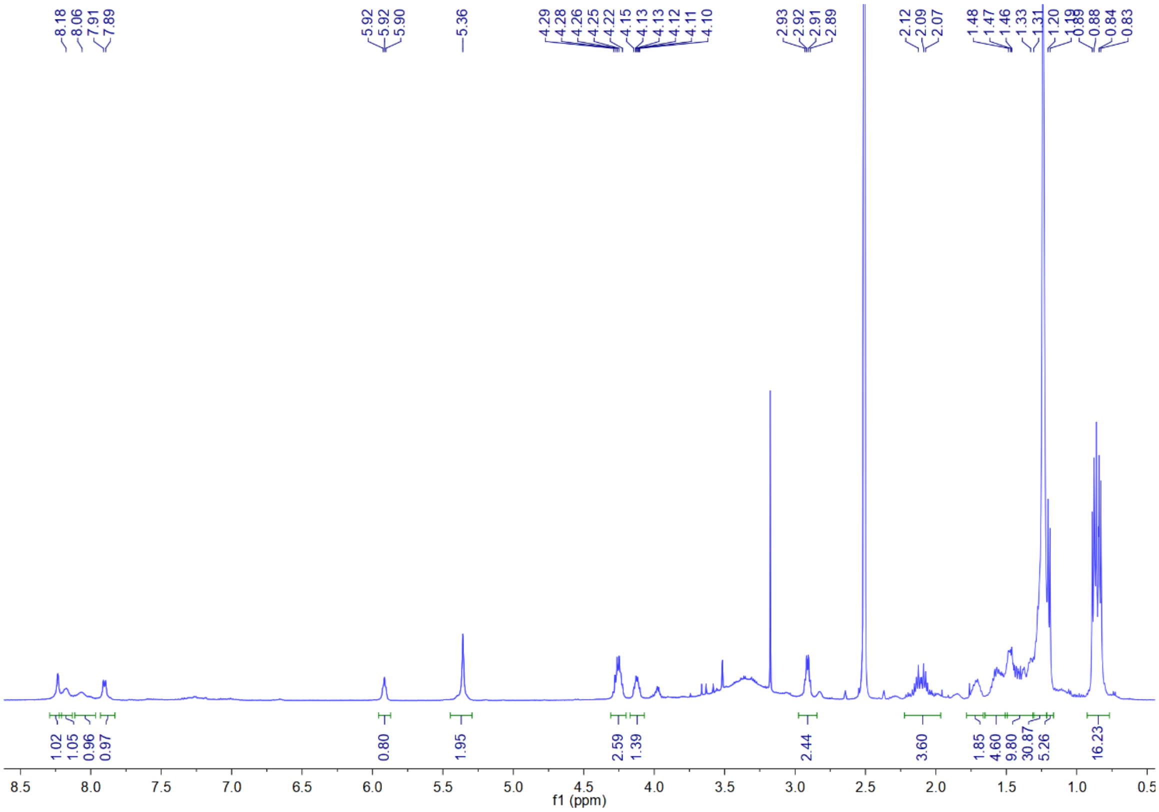

Supplementary Fig. 61 | ^{1}H NMR spectrum of lipocitide B (18) in DMSO-d_6.

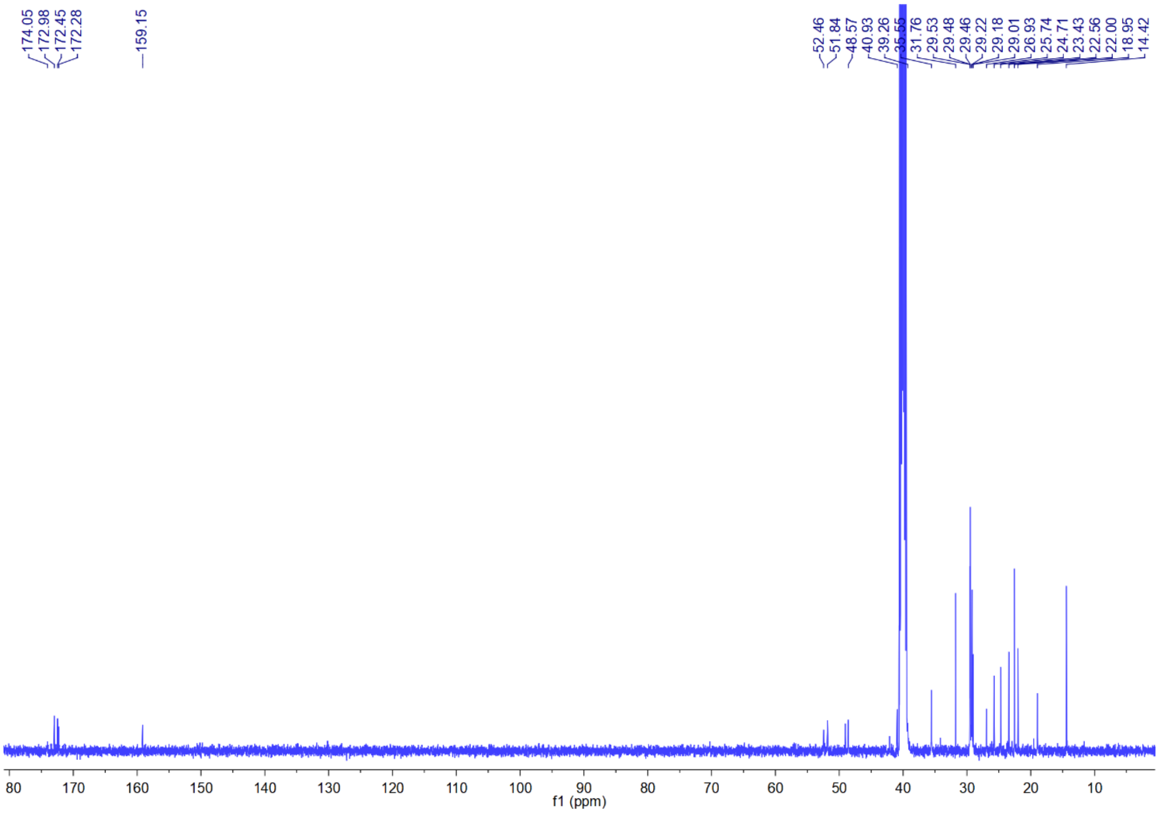

Supplementary Fig. 62 | ^{13}C NMR spectrum of lipocitide B (18) in DMSO-d_6.

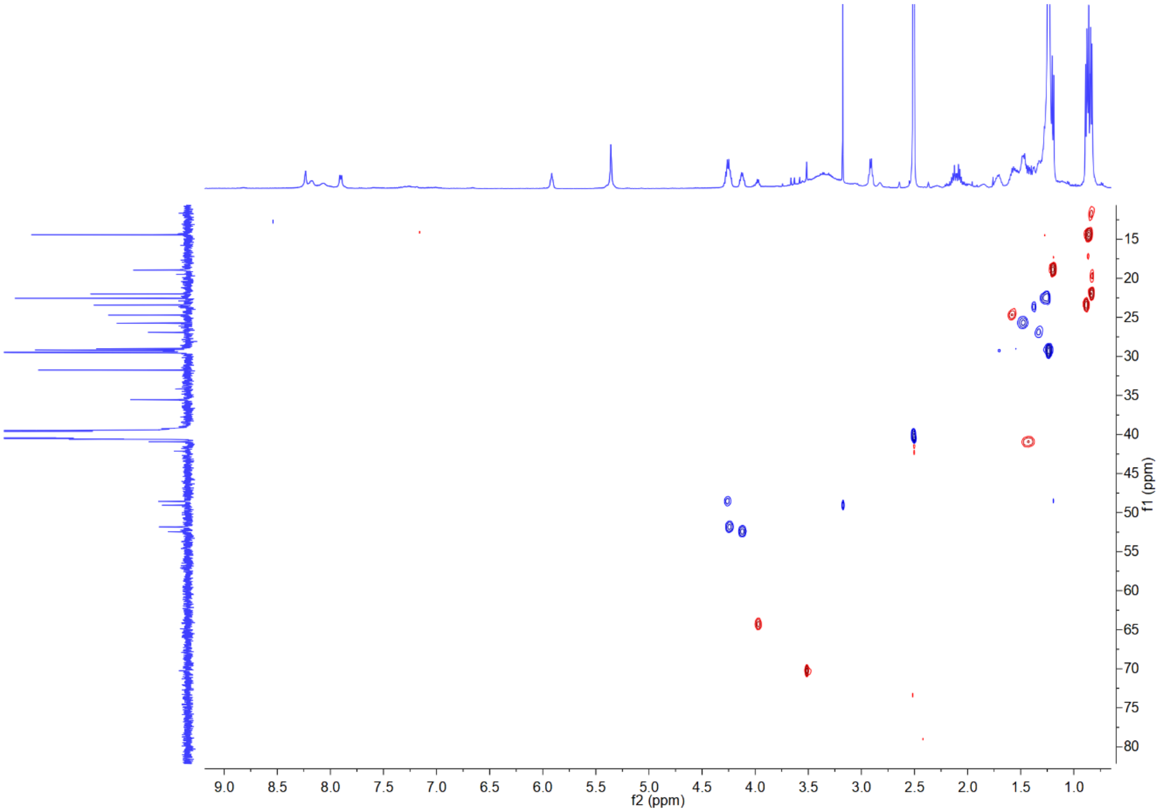

Supplementary Fig. 63 | HSQC spectrum of lipocitide B (18) in DMSO-*d*6.

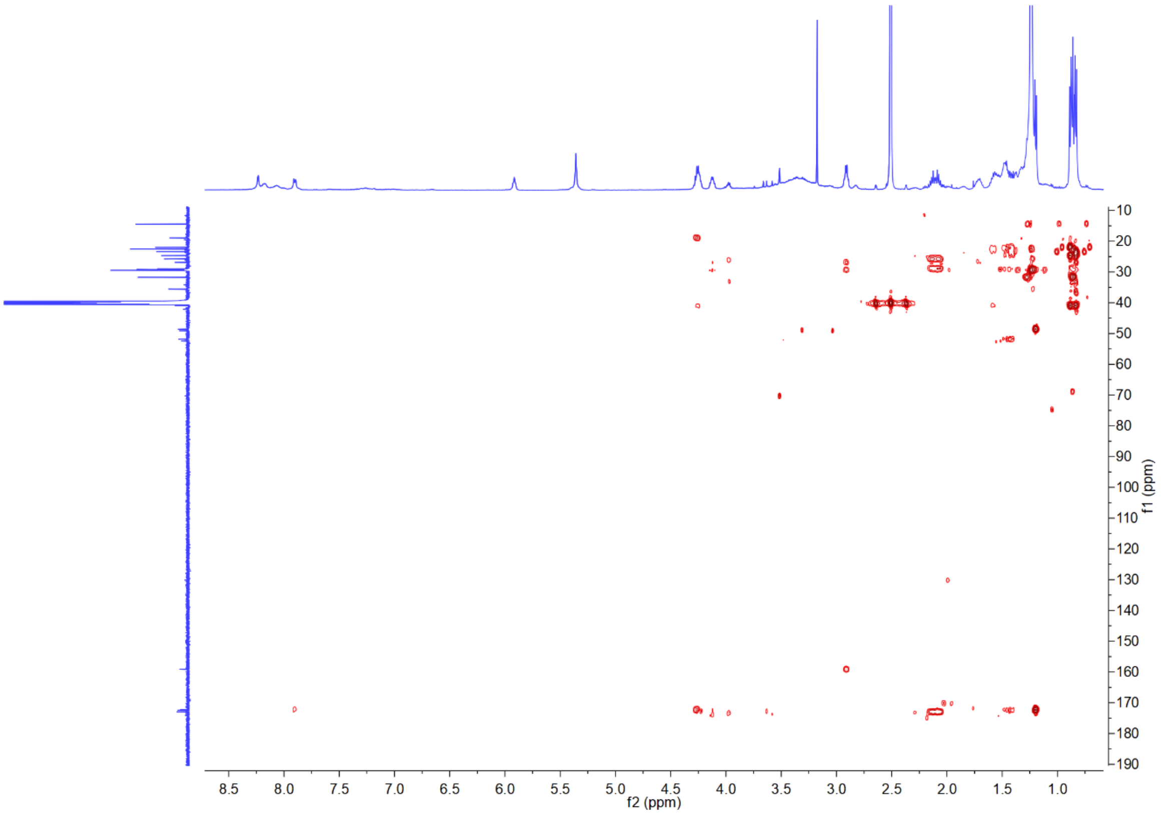

Supplementary Fig. 64 | HMBC spectrum of lipocitide B (18) in DMSO-*d*6.

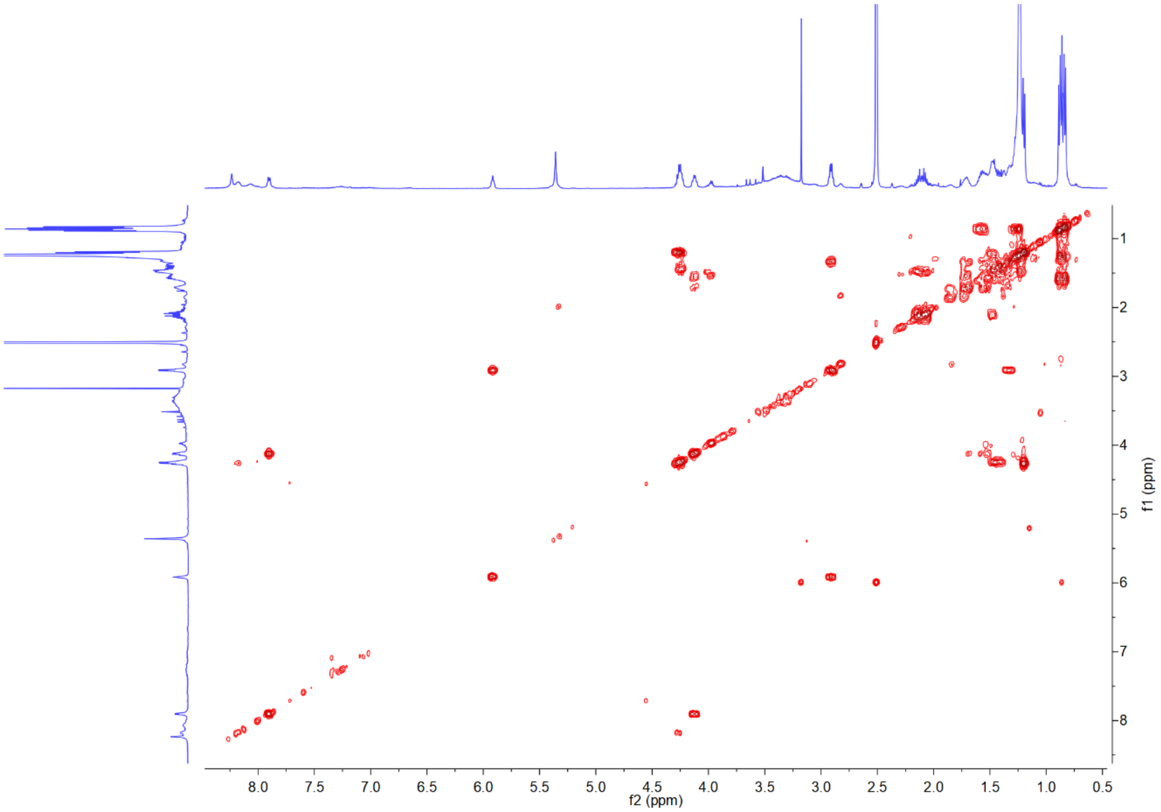

Supplementary Fig. 65 | ^{1}H-^{1}H COSY spectrum of lipocitide B (18) in DMSO-d_6.

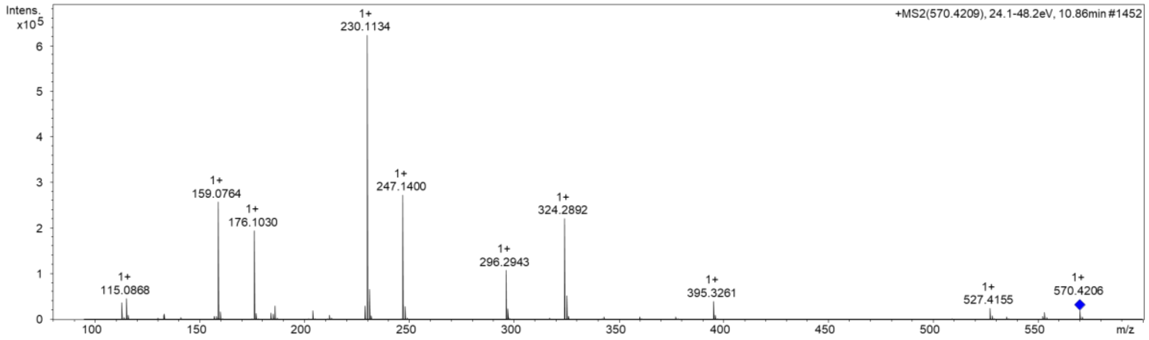

Supplementary Fig. 66 | HR-ESI-MS of lipocitide B (18).

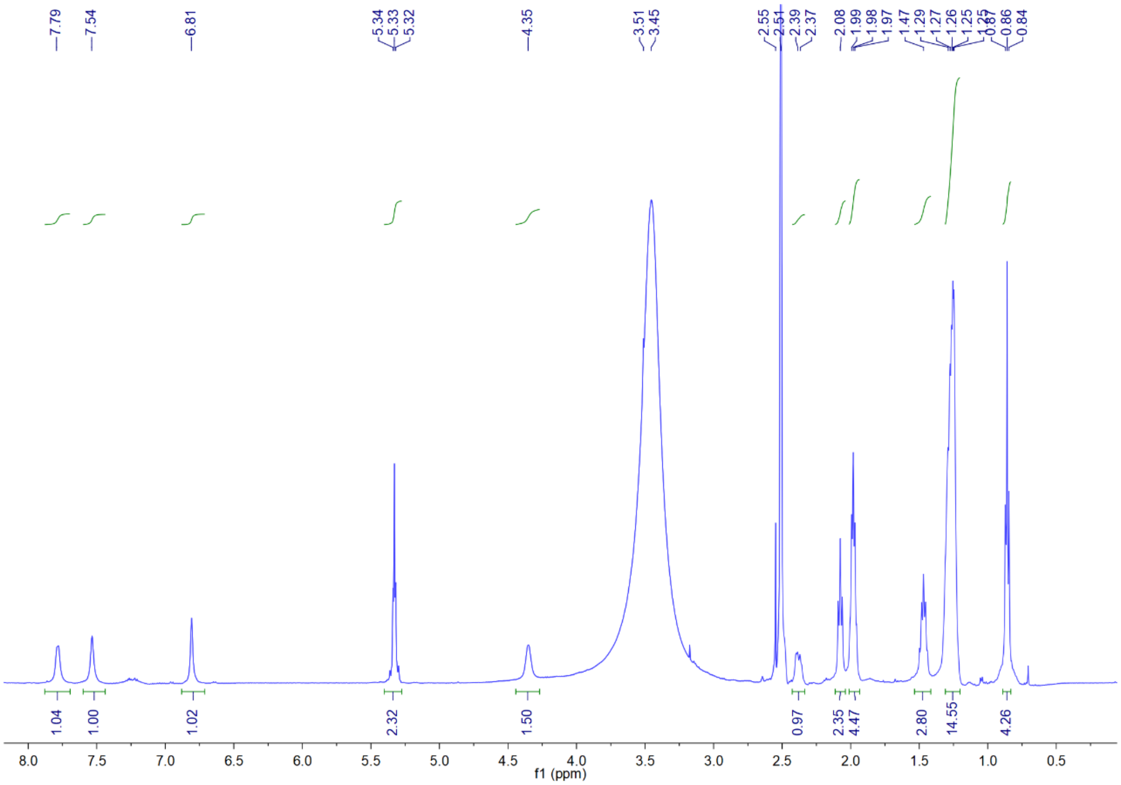

Supplementary Fig. 67 | ^{1}H NMR spectrum of N-(ω-7-myristoyl)-D-asparagine (19) in DMSO-d_6.

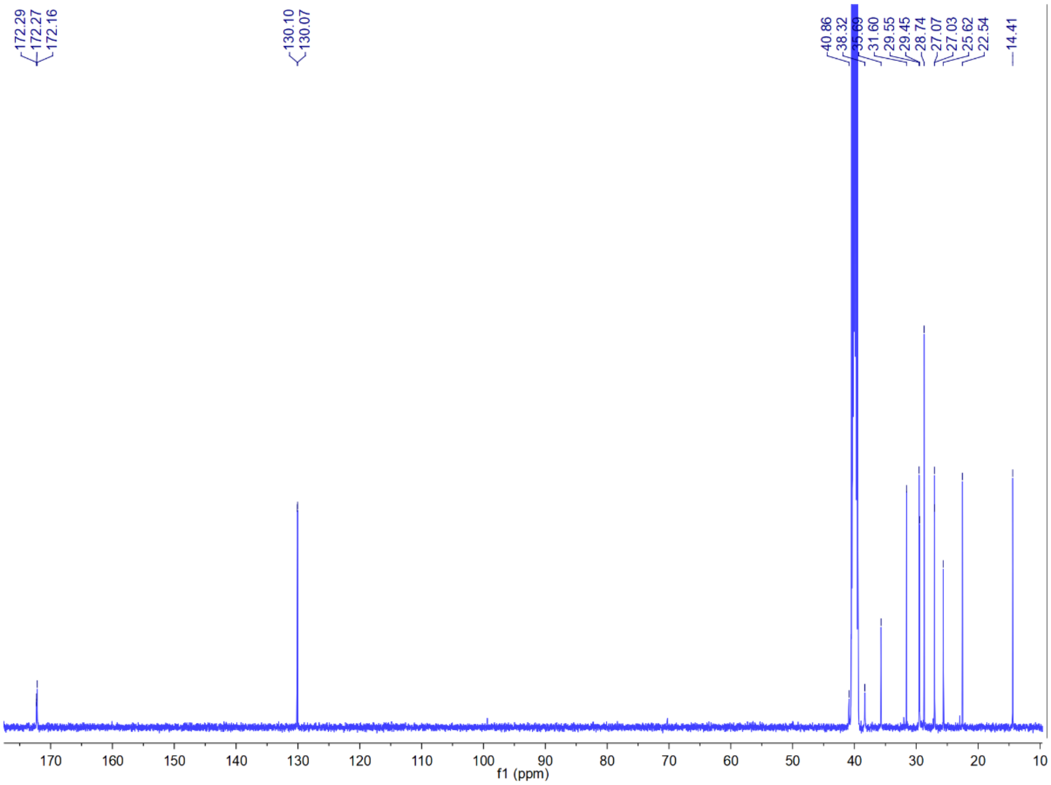

Supplementary Fig. 68 | ^{13}C NMR spectrum of N-(ω-7-myristoyl)-D-asparagine (19) in DMSO-d_6.

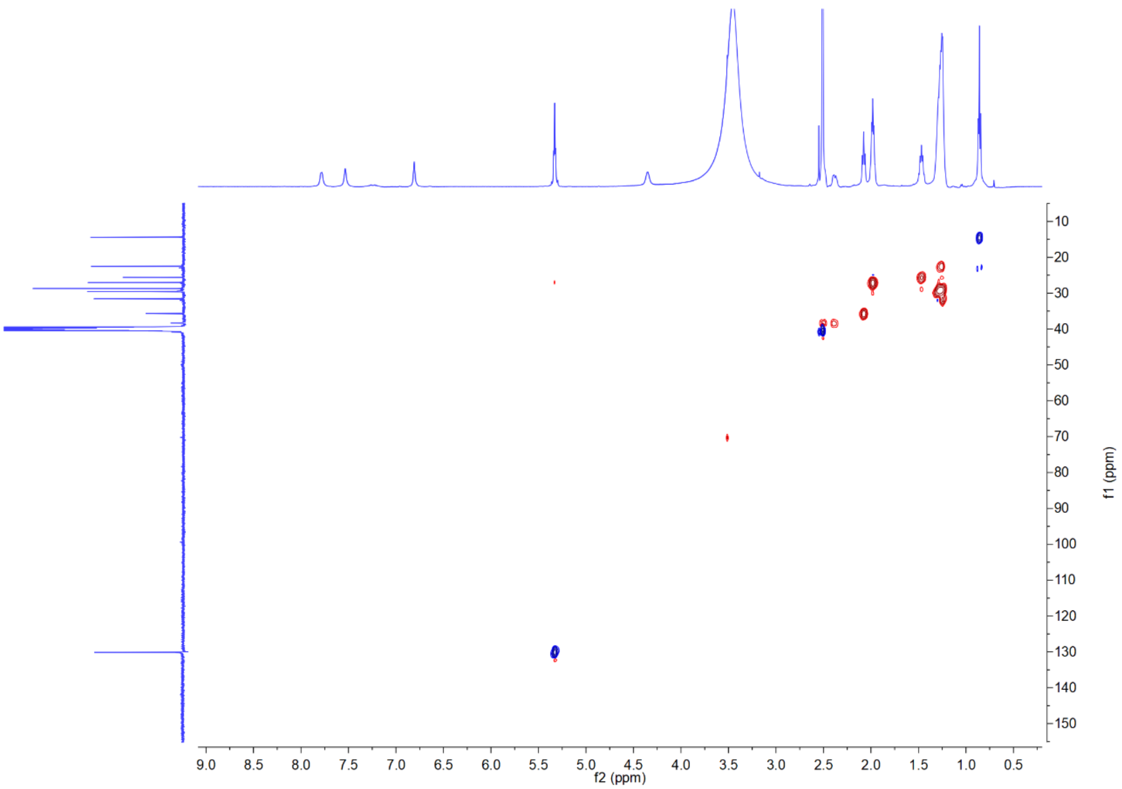

Supplementary Fig. 69 | HSQC spectrum of N-(ω-7-myristoyl)-D-asparagine (19) in DMSO-*d*6.

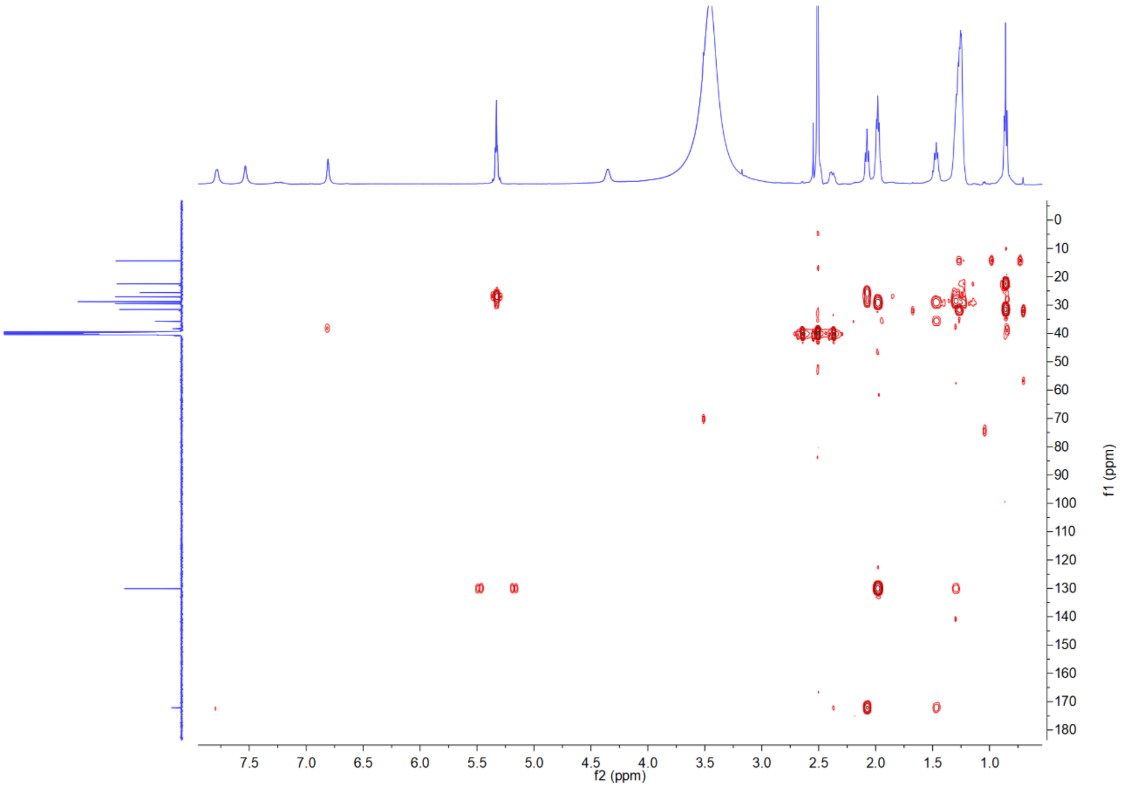

Supplementary Fig. 70 | HMBC spectrum of N-(ω-7-myristoyl)-D-asparagine (19) in DMSO-*d*6.

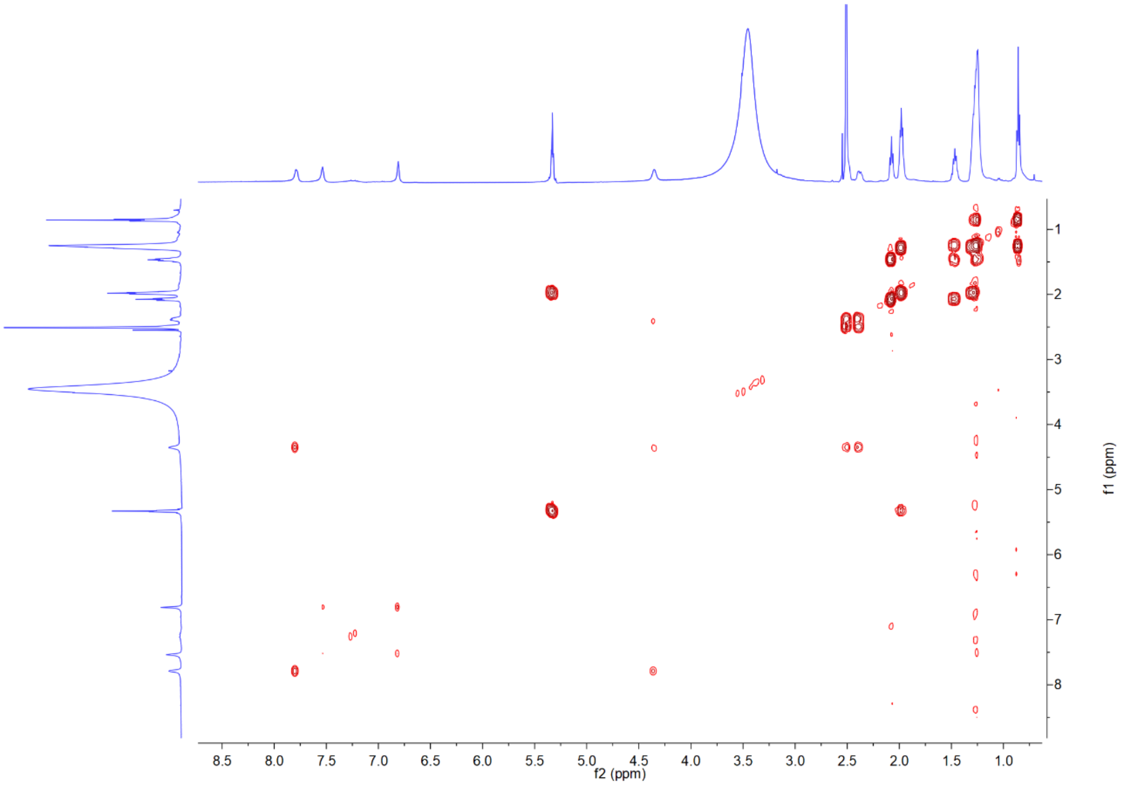

Supplementary Fig. 71 | ^{1}H-^{1}H COSY spectrum of N-(ω-7-myristoyl)-D-asparagine (19) in DMSO-d_6.

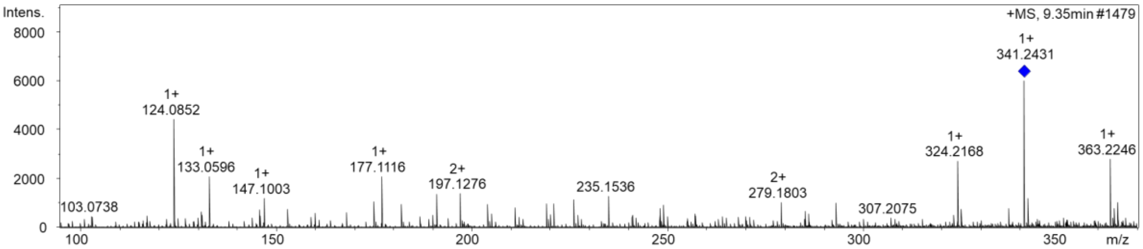

Supplementary Fig. 72 | HR-ESI-MS of N-(ω-7-myristoyl)-D-asparagine (19).

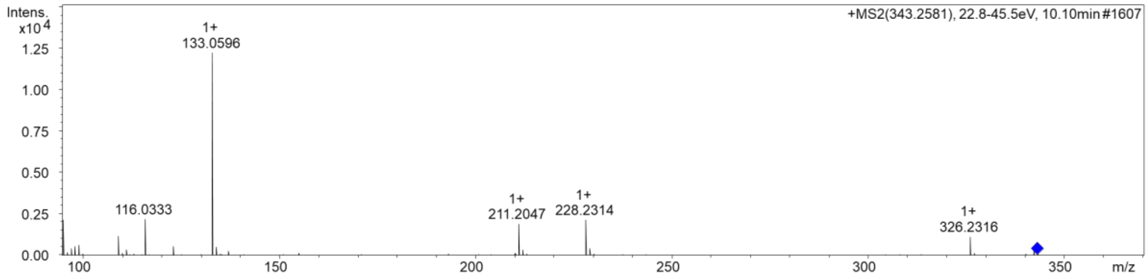

Supplementary Fig. 73 | HR-ESI-MS of N-myristoyl-D-asparagine (20).

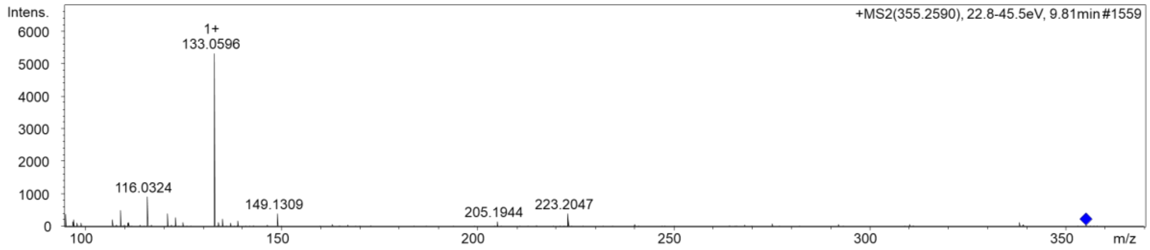

Supplementary Fig. 74 | HR-ESI-MS of N-(13-methyl-ω-7-myristoyl)-D-asparagine (21).

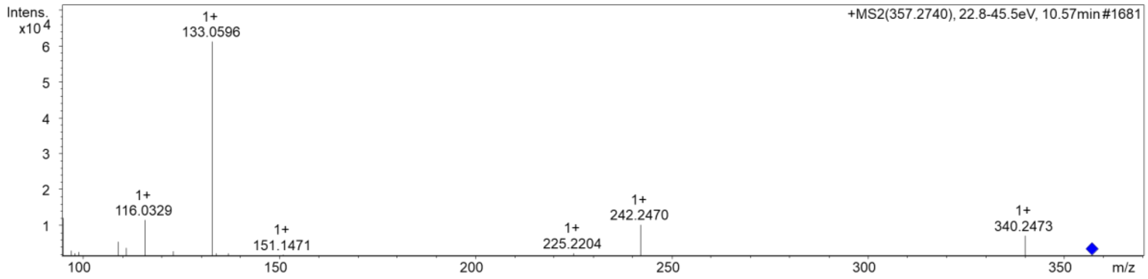

Supplementary Fig. 75 | HR-ESI-MS of N-(13-methylmyristoyl)-D-asparagine (22).

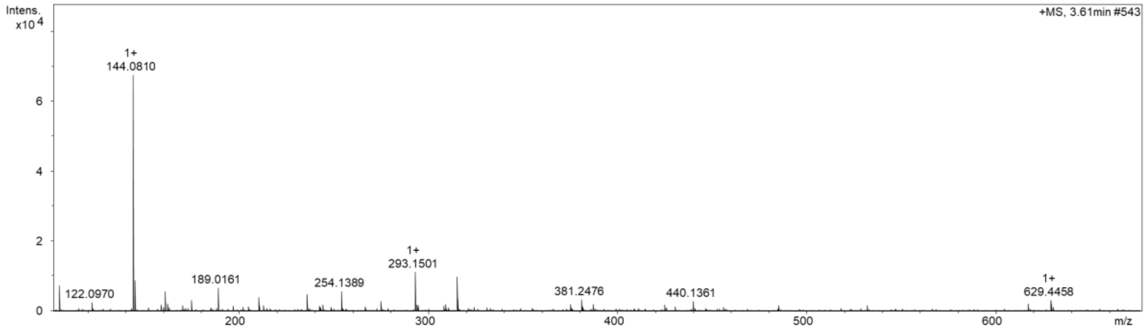

Supplementary Fig. 76 | HR-ESI-MS of rhabdobranin (23).

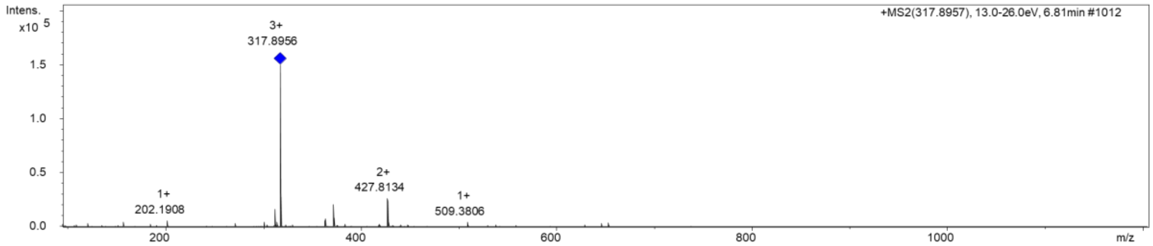

Supplementary Fig. 77 | HR-ESI-MS of pre-rhabdobranin A (24).

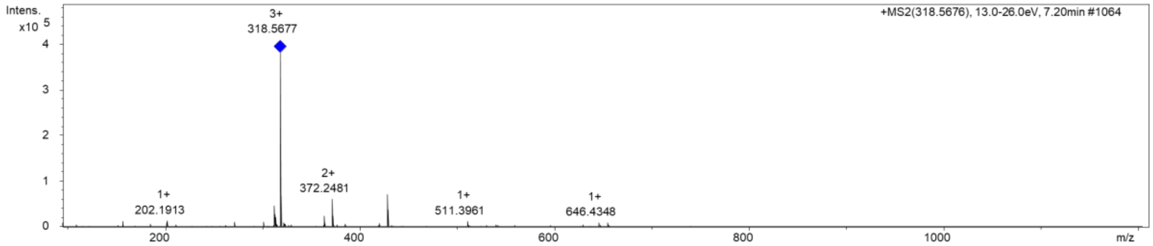

Supplementary Fig. 78 | HR-ESI-MS of pre-rhabdobranin B (25).

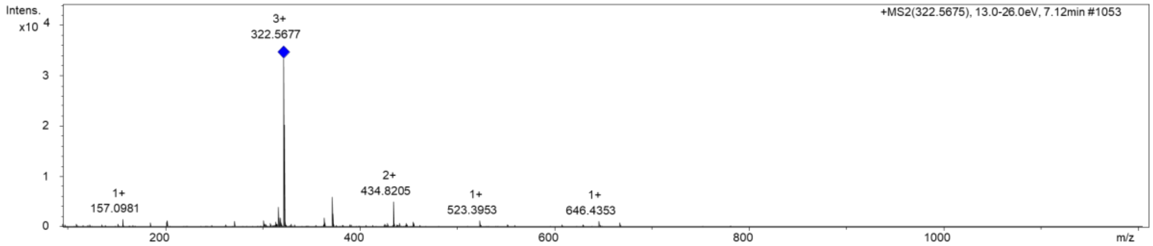

Supplementary Fig. 79 | HR-ESI-MS of pre-rhabdobranin C (26).

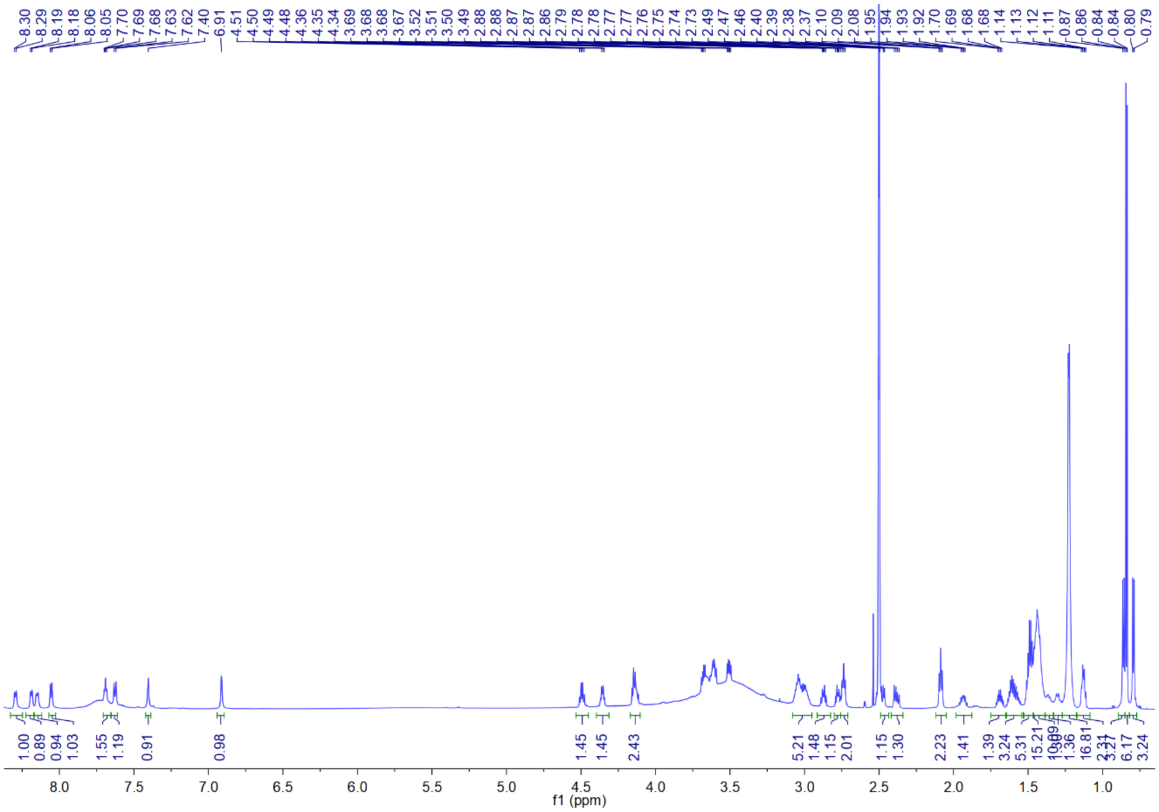

Supplementary Fig. 80 | ^{1}H NMR spectrum of pre-rhabdobranin D (27) in DMSO-d_6.

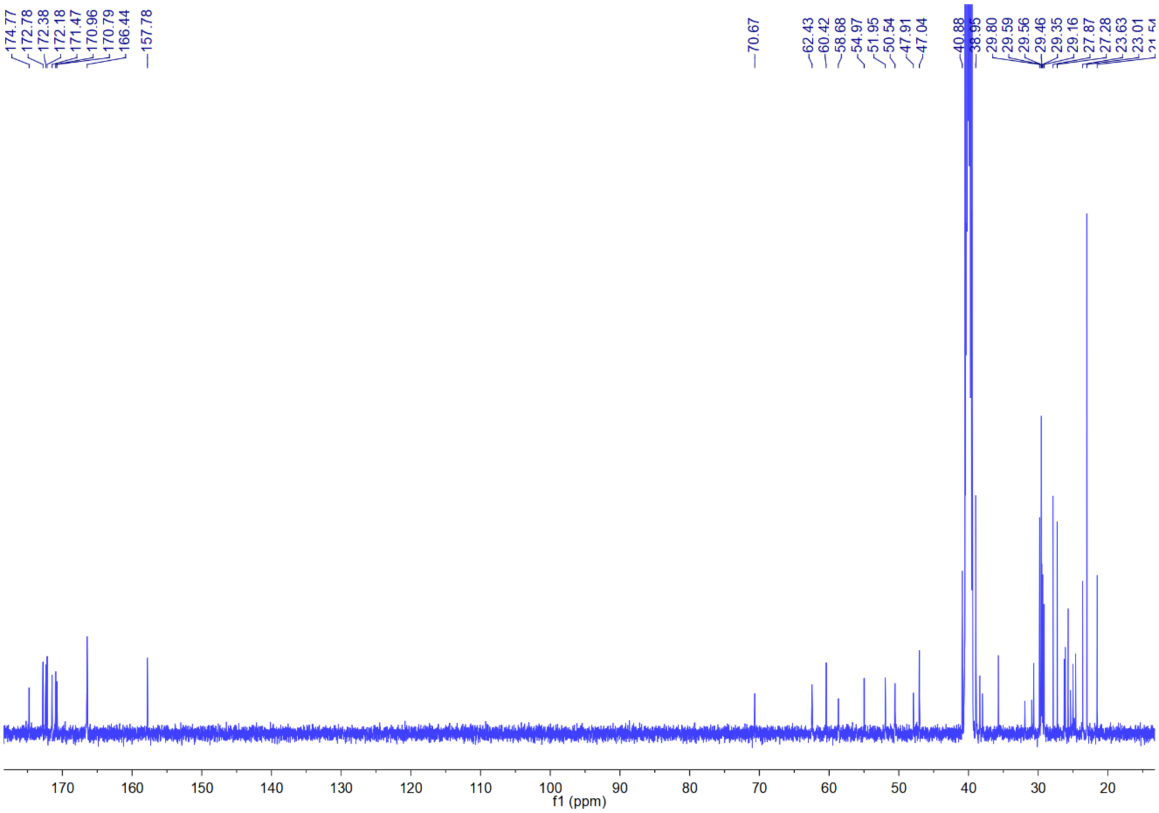

Supplementary Fig. 81 | ^{13}C NMR spectrum of pre-rhabdobranin D (27) in DMSO-d_6.

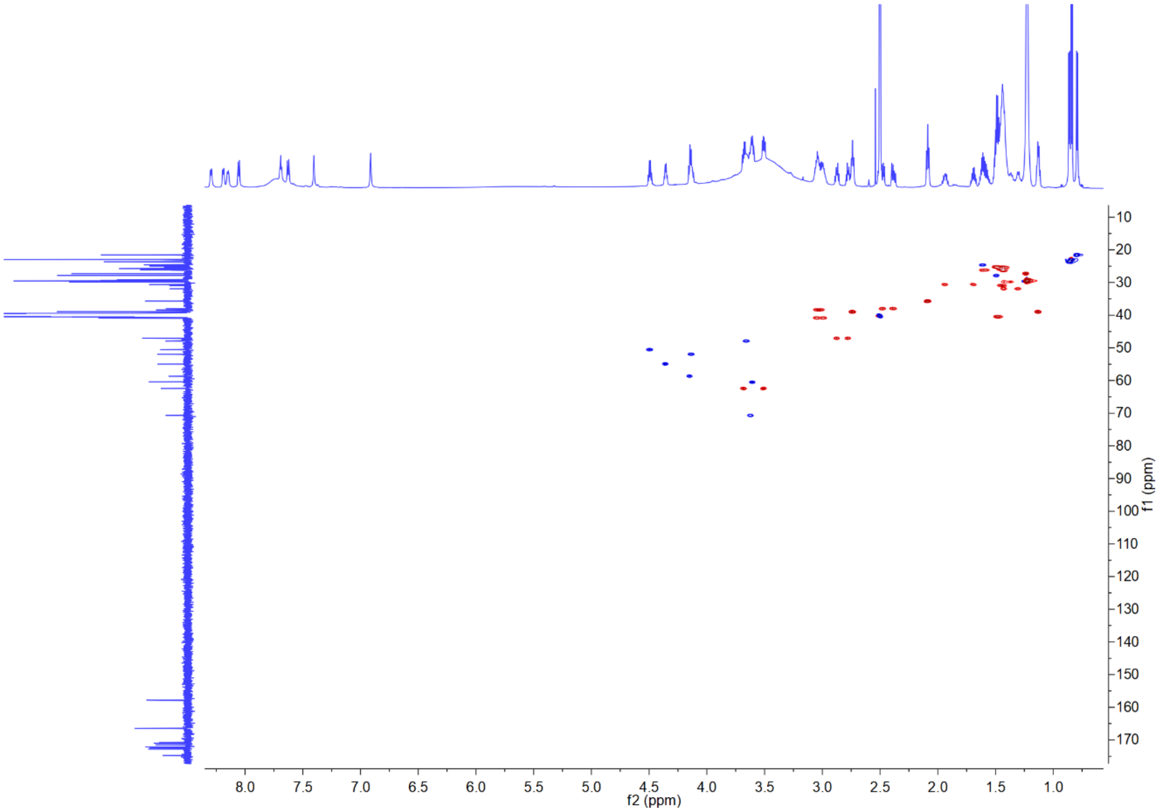

Supplementary Fig. 82 | HSQC spectrum of pre-rhabdobranin D (27) in DMSO-d_6.

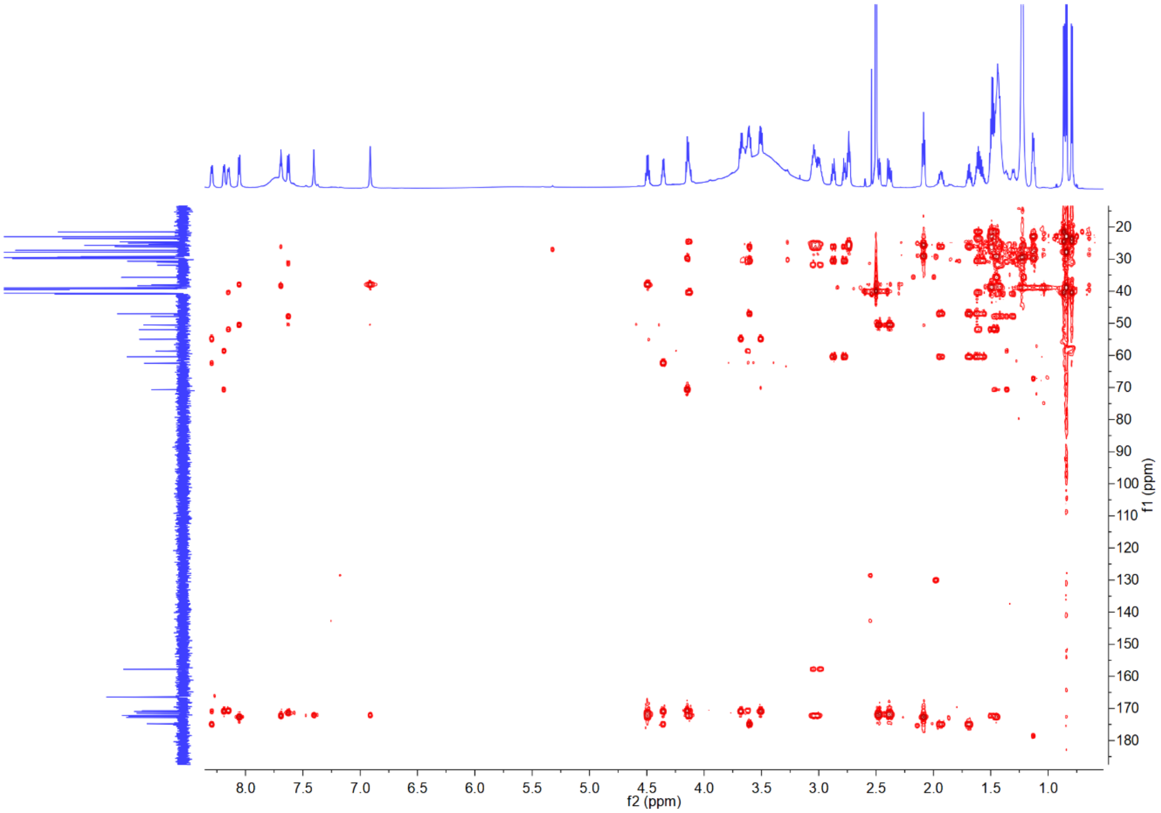

Supplementary Fig. 83 | HMBC spectrum of pre-rhabdobranin D (27) in DMSO-d_6.

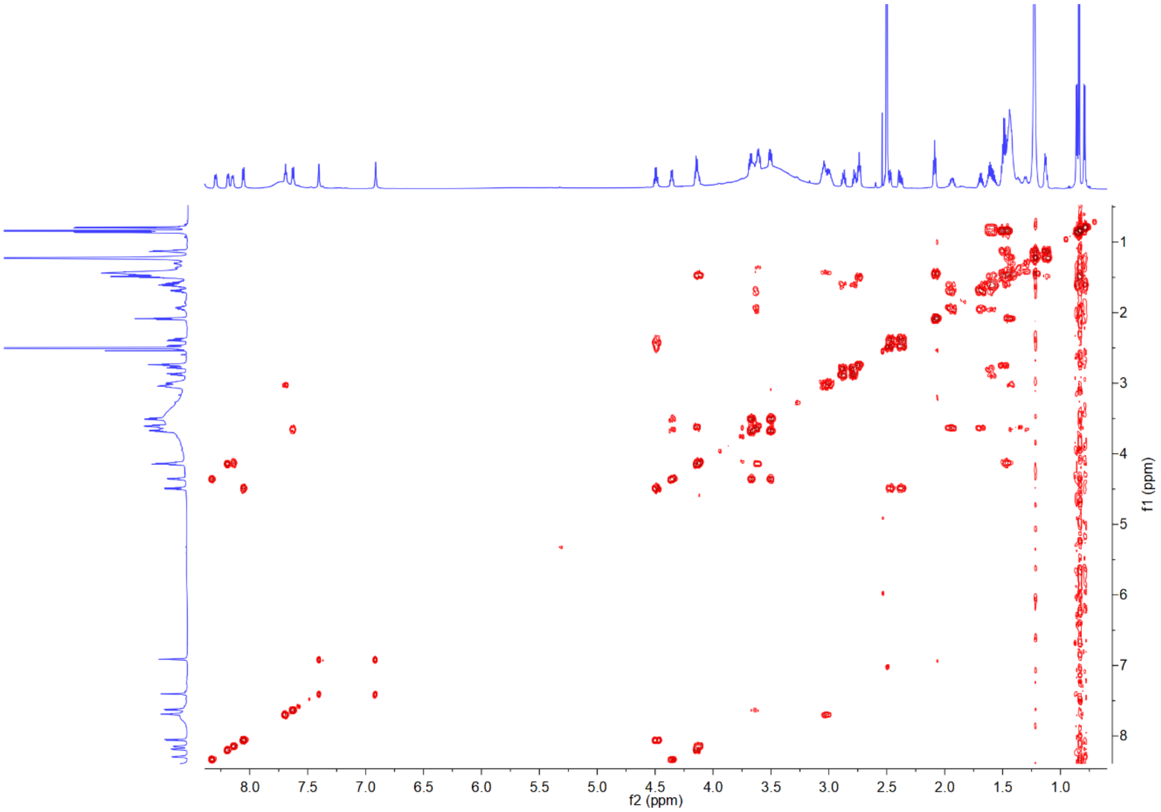

Supplementary Fig. 84 | ^{1}H-^{1}H COSY spectrum of pre-rhabdobranin D (27) in DMSO-d_6.

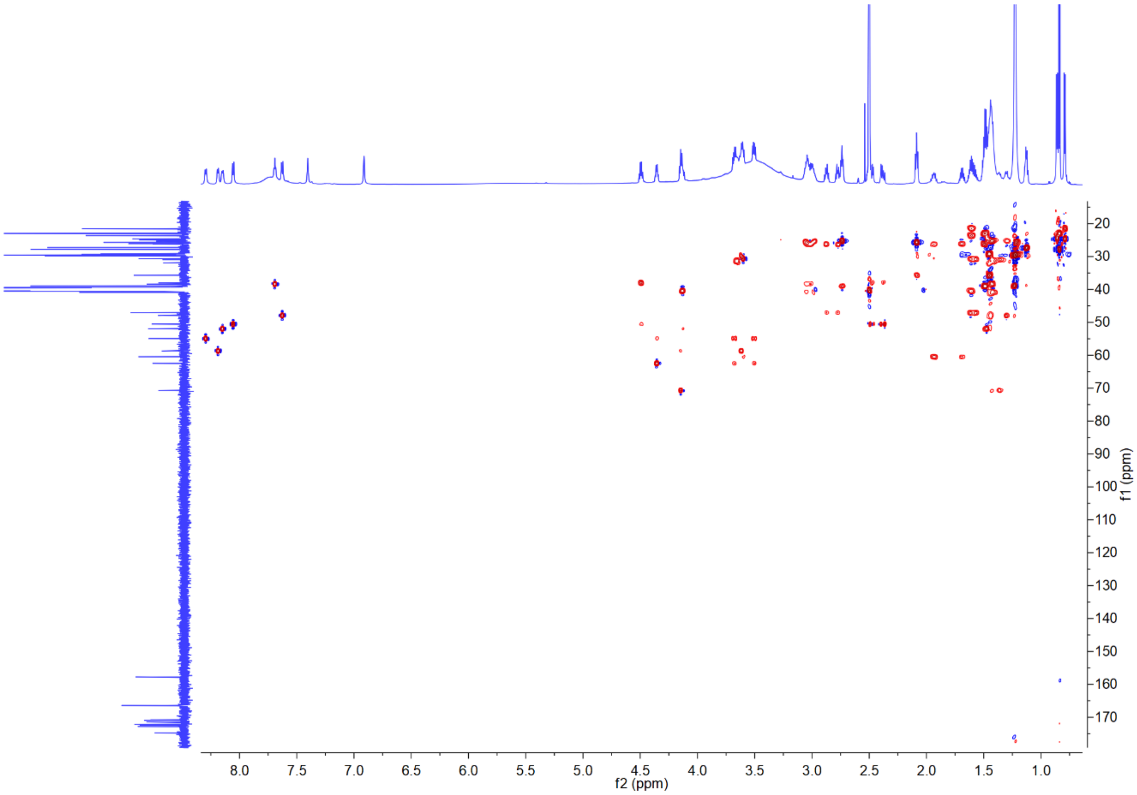

Supplementary Fig. 85 | HMQC-COSY spectrum of pre-rhabdobranin D (27) in DMSO-d_6.

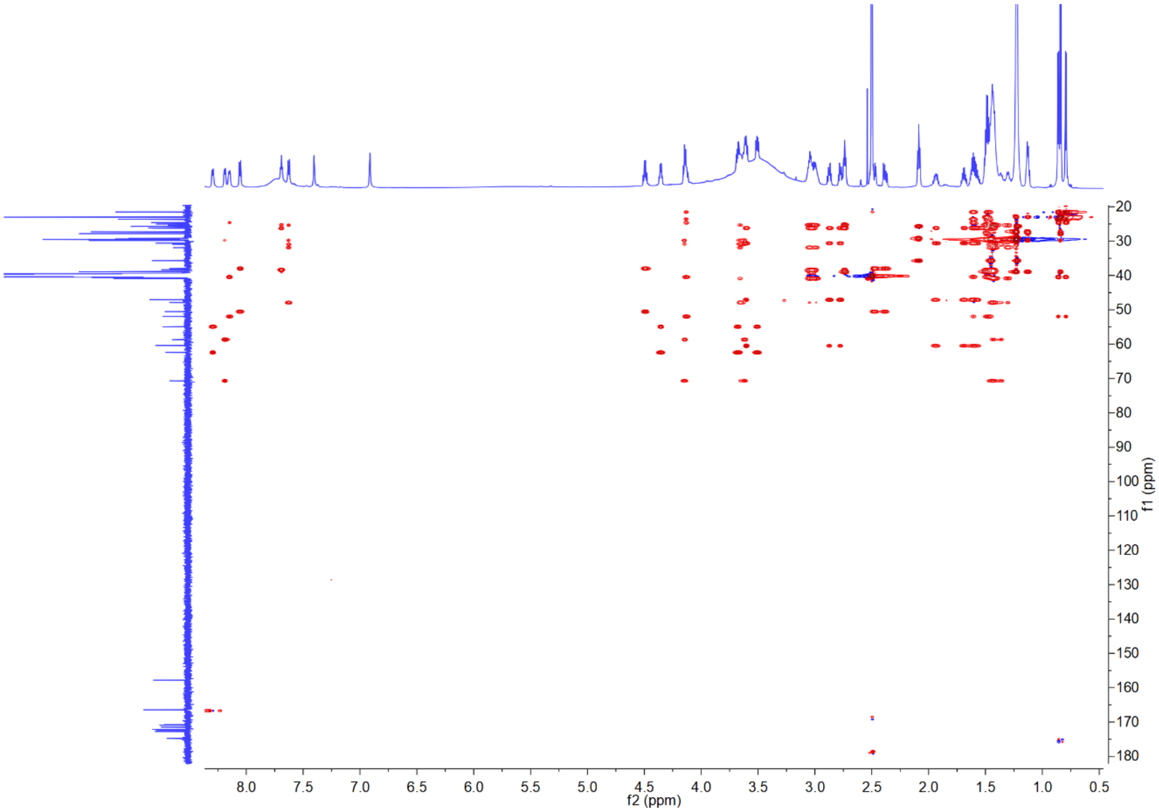

Supplementary Fig. 86 | HSQC-TOCSY spectrum of pre-rhabdobranin D (27) in DMSO-d_6.

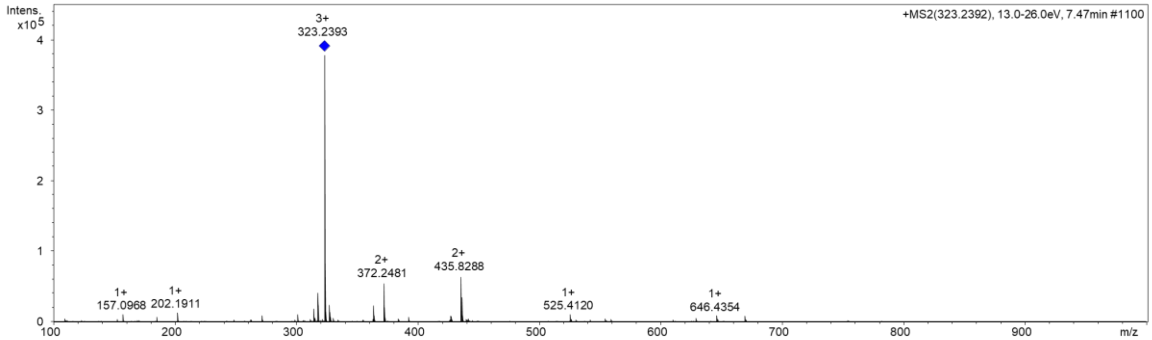

Supplementary Fig. 87 | HR-ESI-MS of pre-rhabdobranin D (27).

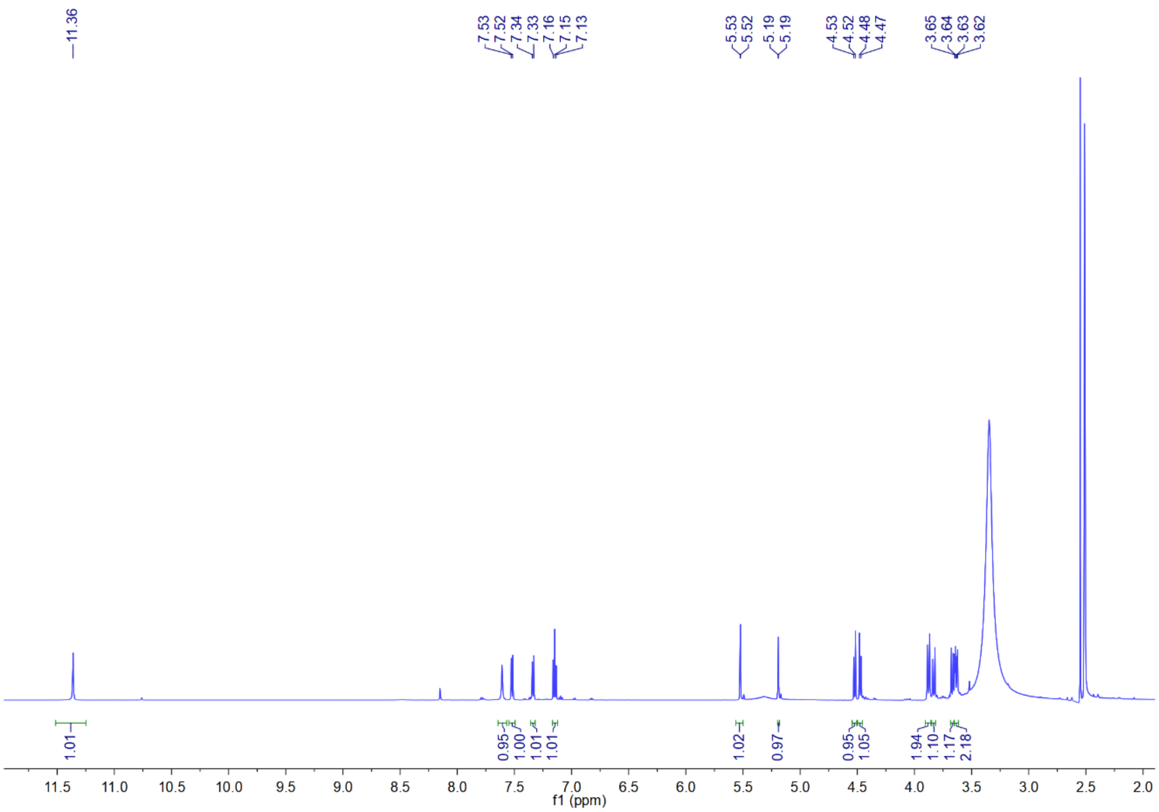

Supplementary Fig. 88 | ^{1}H NMR spectrum of benzobactin A (28) in DMSO-d_6.

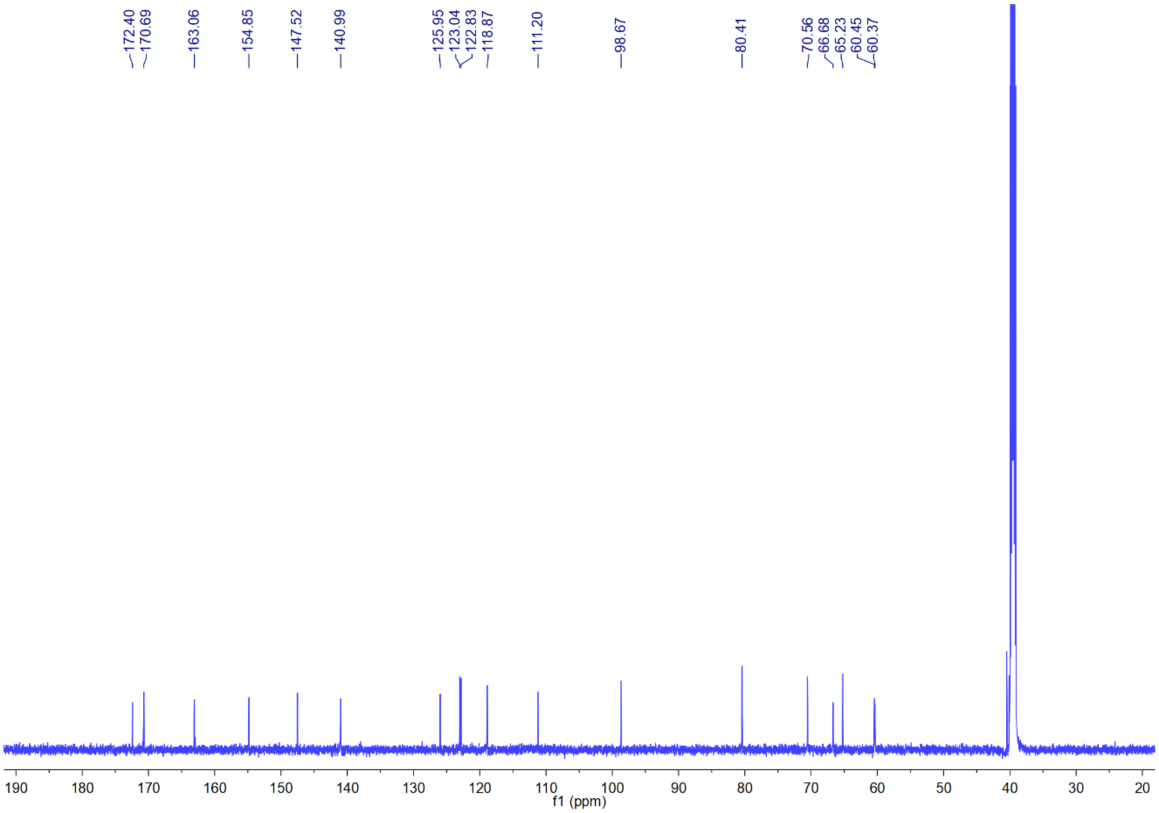

Supplementary Fig. 89 | ^{13}C NMR spectrum of benzobactin A (28) in DMSO-d_6.

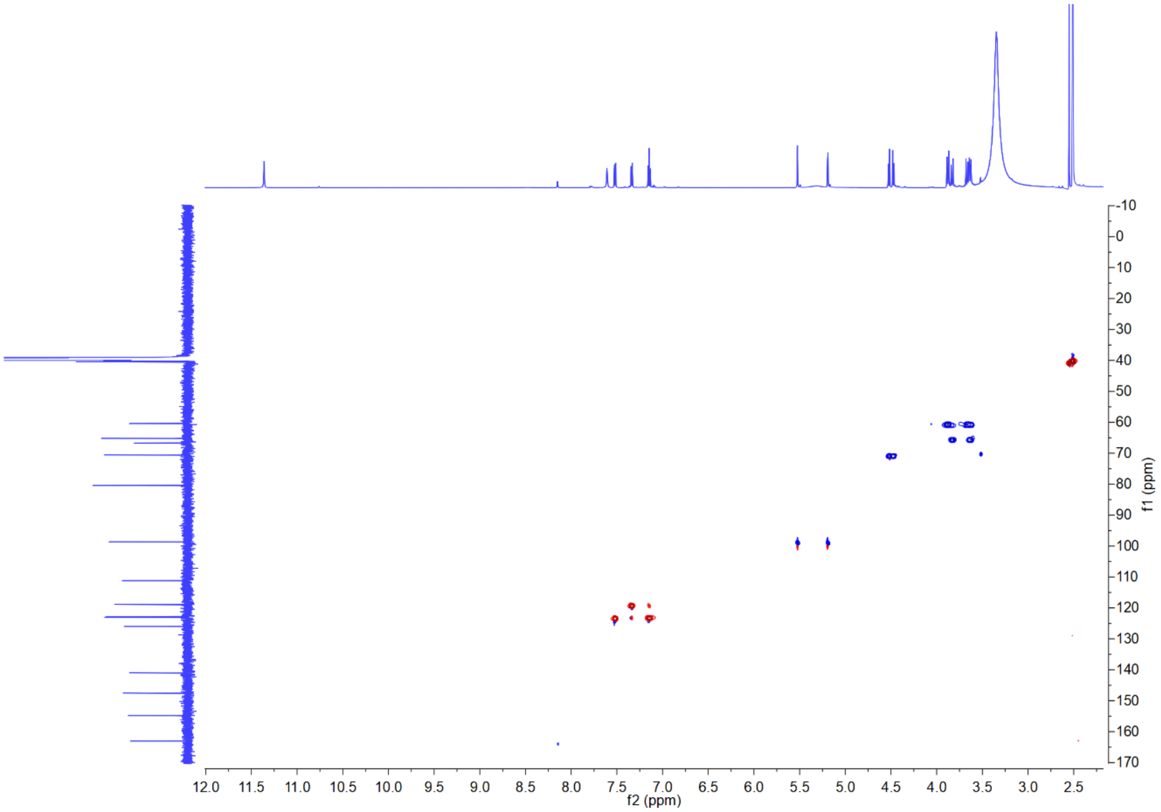

Supplementary Fig. 90 | HSQC spectrum of benzobactin A (28) in DMSO-*d*6.

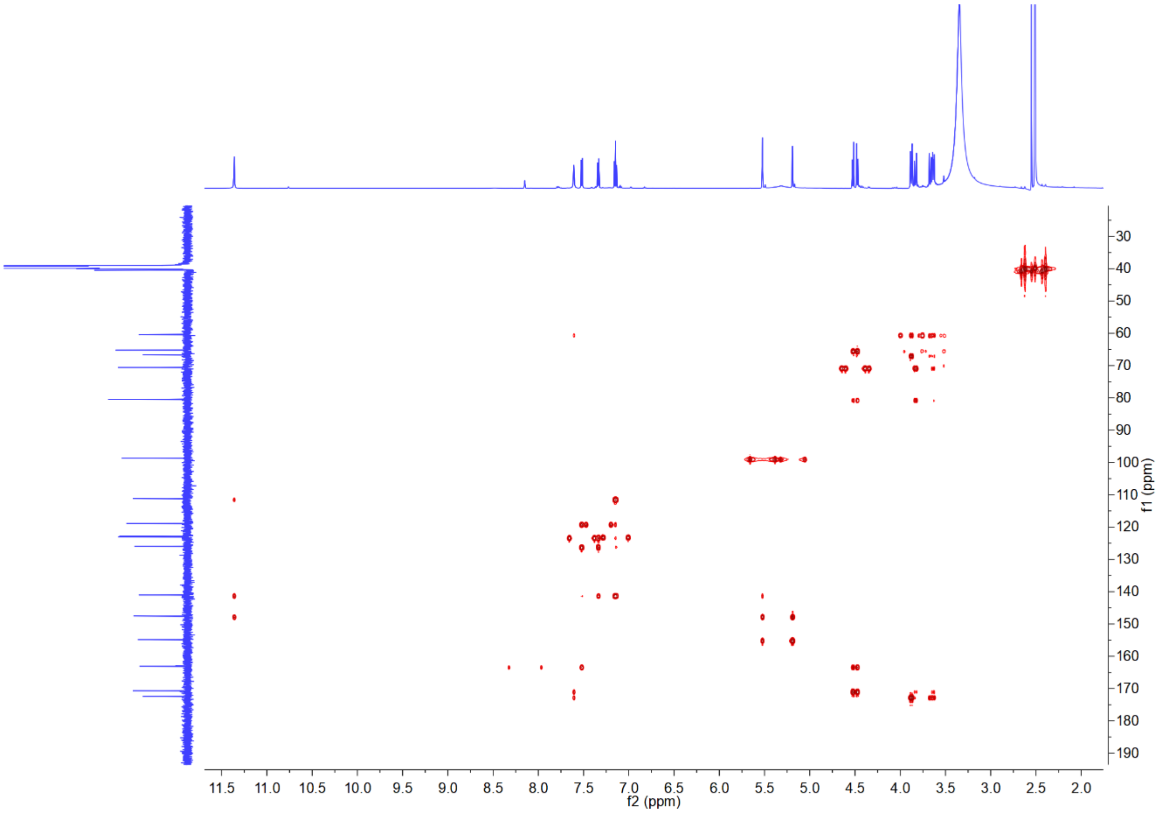

Supplementary Fig. 91 | HMBC spectrum of benzobactin A (28) in DMSO-*d*6.

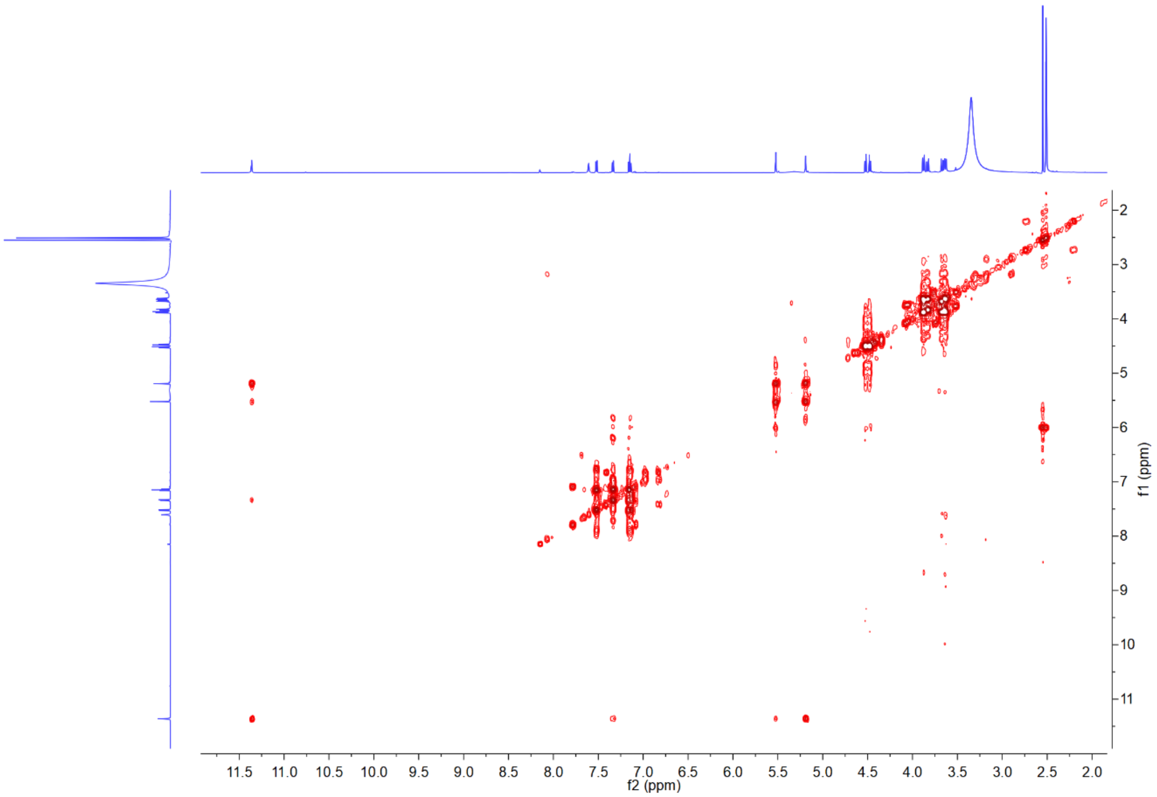

Supplementary Fig. 92 | ^{1}H-^{1}H COSY spectrum of benzobactin A (28) in DMSO-d_6.

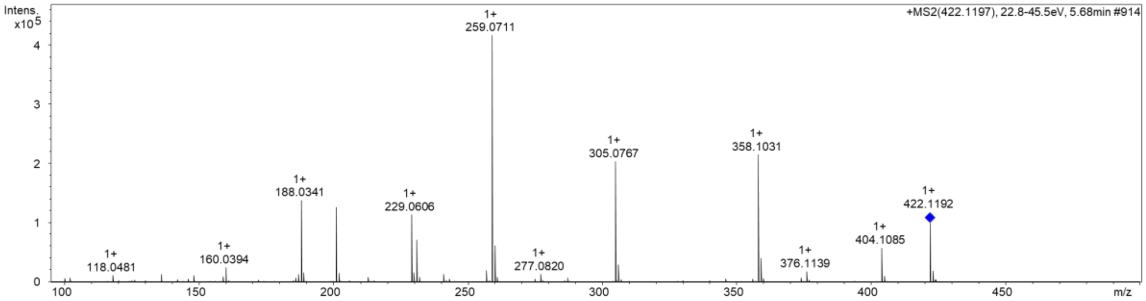

Supplementary Fig. 93 | HR-ESI-MS of benzobactin A (28).

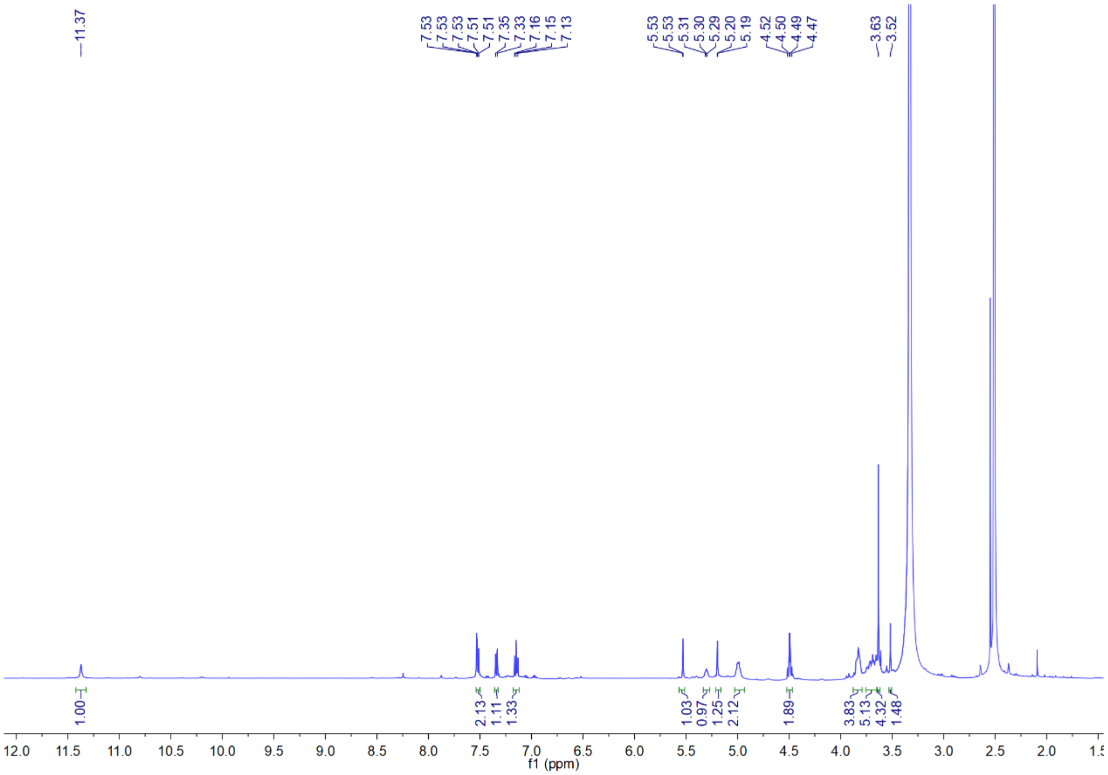

Supplementary Fig. 94 | ^{1}H NMR spectrum of benzobactin A methyl ester (29) in DMSO-*d*6.

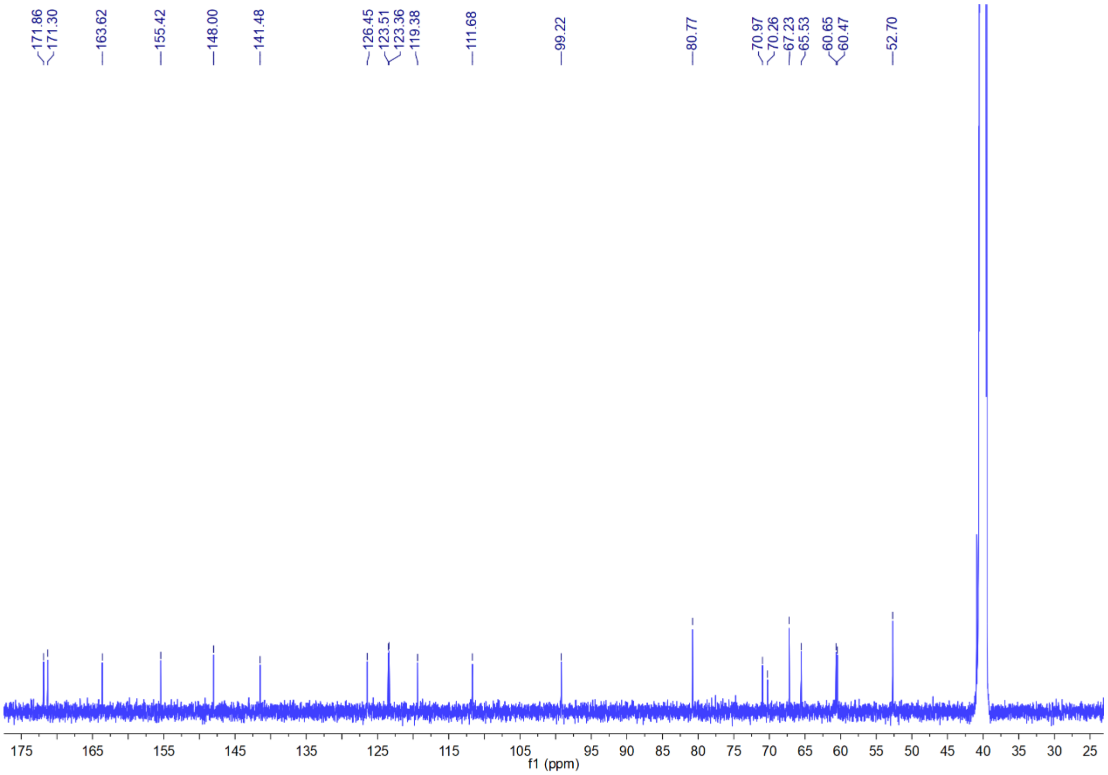

Supplementary Fig. 95 | ^{13}C NMR spectrum of benzobactin A methyl ester (29) in DMSO-*d*6.

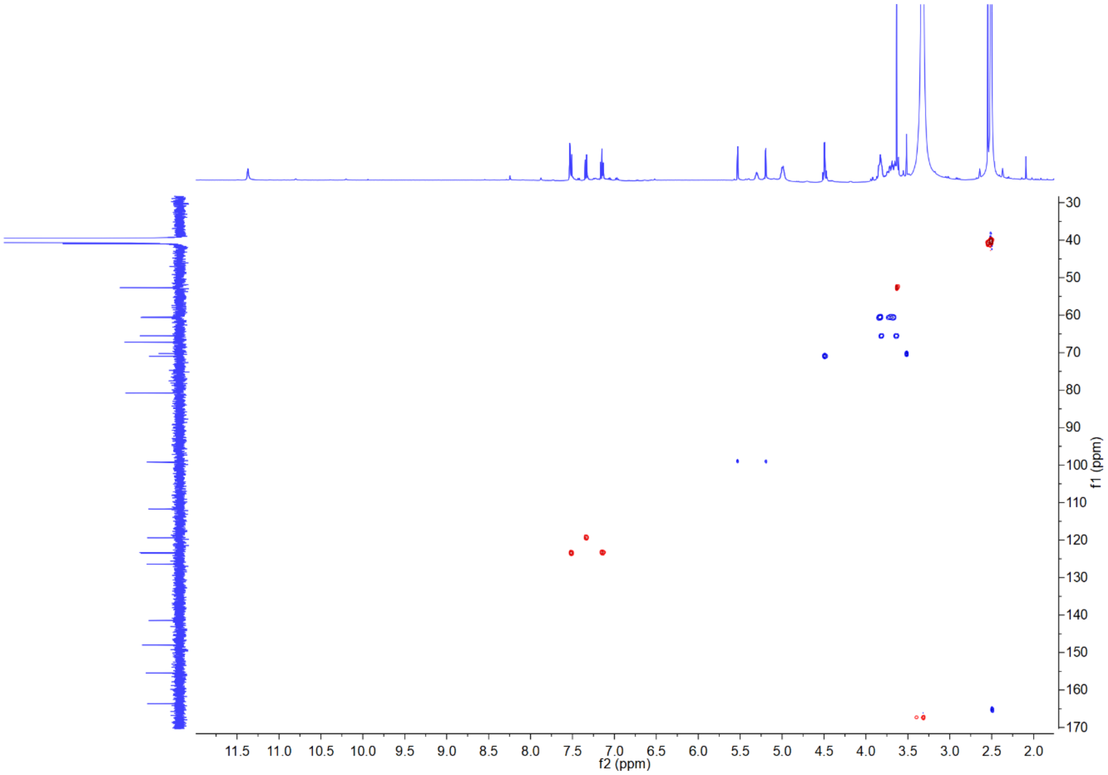

Supplementary Fig. 96 | HSQC spectrum of benzobactin A methyl ester (29) in DMSO-d_6.

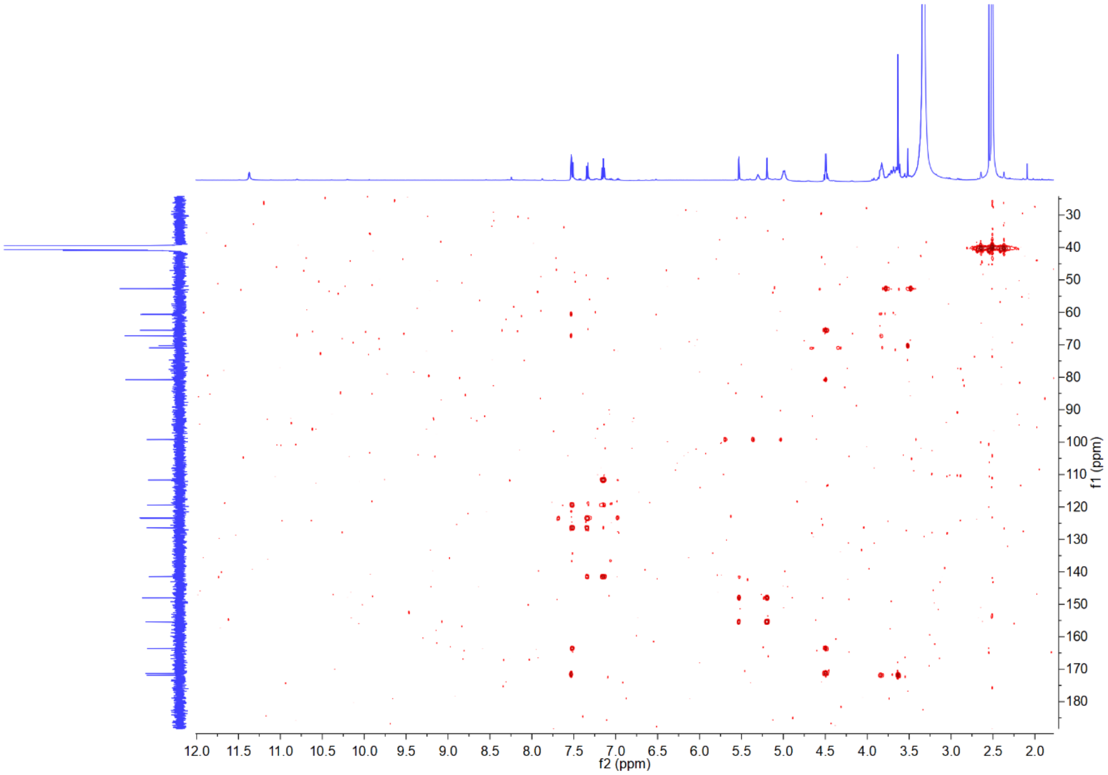

Supplementary Fig. 97 | HMBC spectrum of benzobactin A methyl ester (29) in DMSO-d_6.

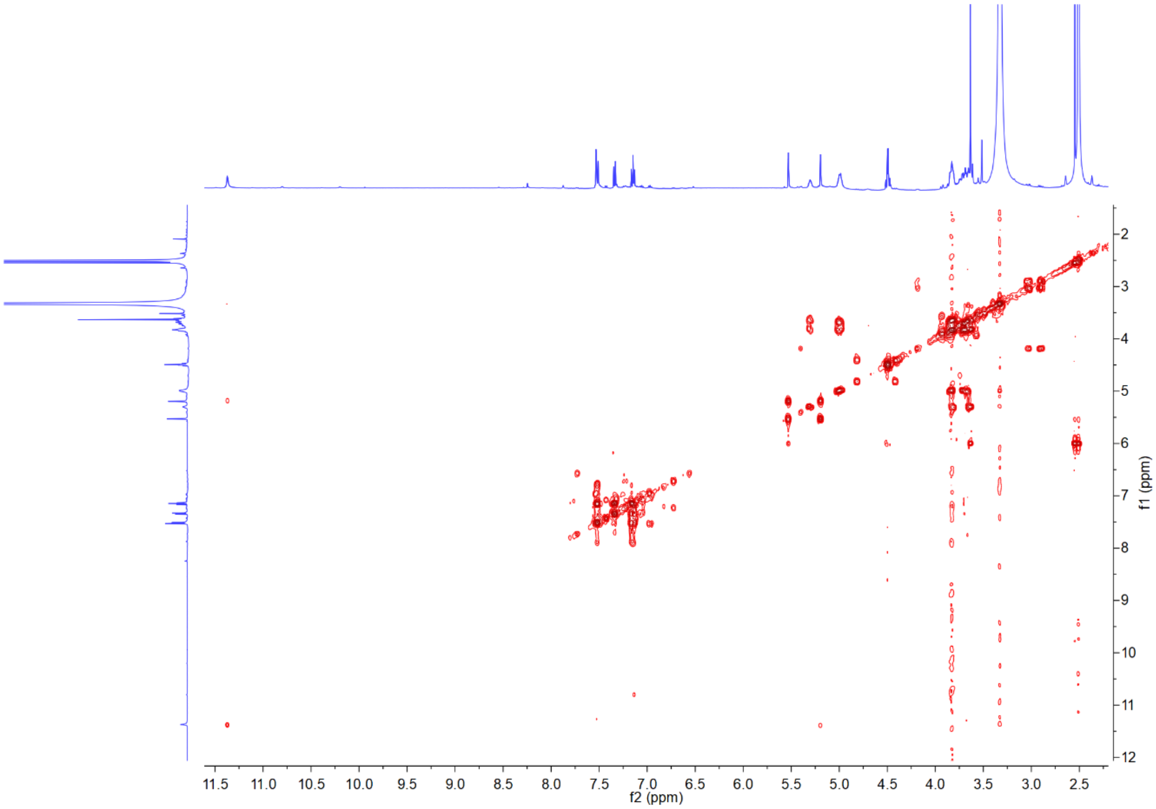

Supplementary Fig. 98 | ^{1}H-^{1}H COSY spectrum of benzobactin A methyl ester (29) in DMSO-*d*$_6$.

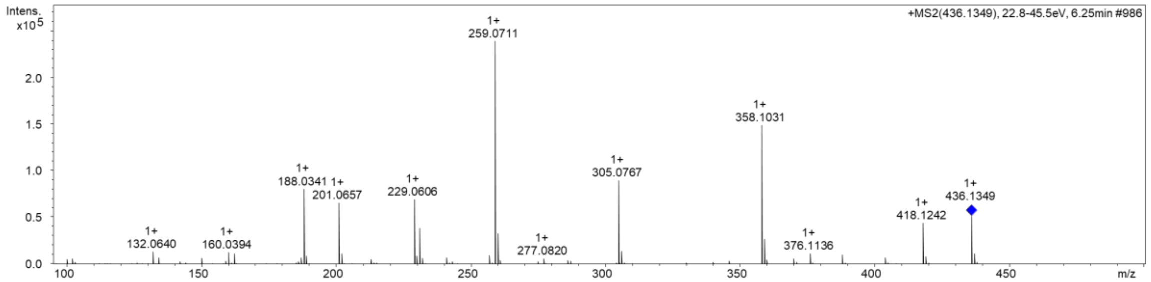

Supplementary Fig. 99 | HR-ESI-MS of benzobactin A methyl ester (29).

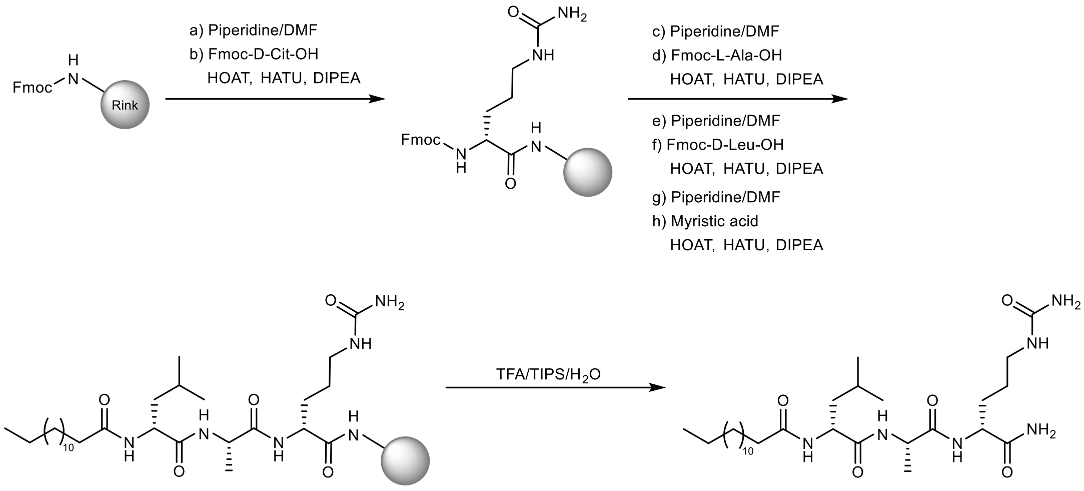

17

Supplementary Fig. 100 | Synthetic route of lipocitide A (17).

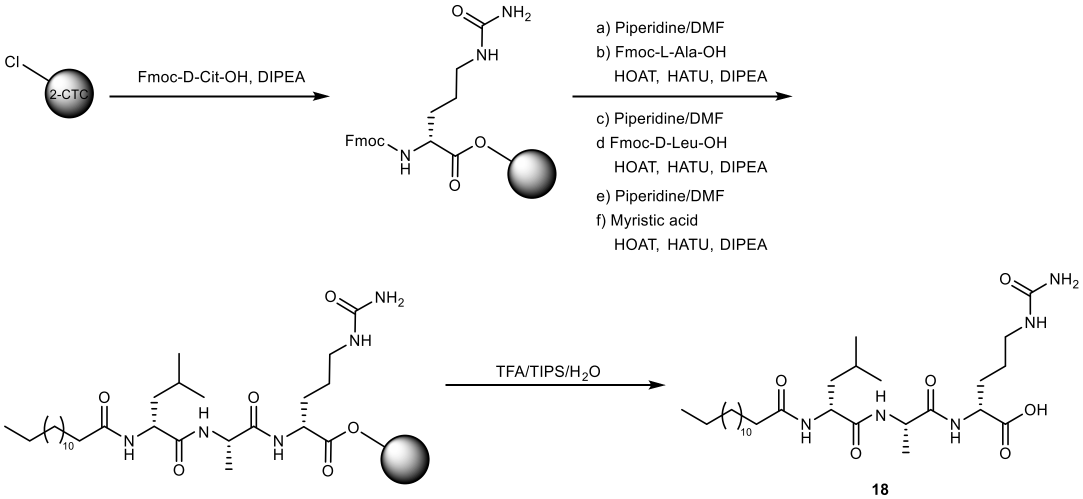

18

Supplementary Fig. 101 | Synthetic route of lipocitide B (18).

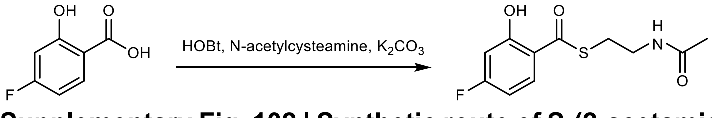

Supplementary Fig. 102 | Synthetic route of S-(2-acetamidoethyl)4-fluoro-2-hydroxybenzothioate (4-fluorosalicylate SNAC).

References

1. Eren, A. M. *et al.* Community-led, integrated, reproducible multi-omics with anvi'o. *Nat. Microbiol.* **6**, 3-6 (2021).
2. Navarro-Muñoz, J. C. *et al.* A computational framework to explore large-scale biosynthetic diversity. *Nat. Chem. Biol.* **16**, 60-68 (2019).
3. Chaturvedi, K. S. *et al.* Cupric yersiniabactin is a virulence-associated superoxide dismutase mimic. *ACS Chem. Biol.* **9**, 551-561 (2014).
4. Tobias, N. J. *et al.* Natural product diversity associated with the nematode symbionts *Photorhabdus* and *Xenorhabdus*. *Nat. Microbiol.* **2**, 1676-1685 (2017).
5. Johnston, I. *et al.* Identification of essential genes for *Escherichia coli* aryl polyene biosynthesis and function in biofilm formation. *NPJ Biofilms Microbiomes* **7**, 56 (2021).
6. Grammbitter, G. L. *et al.* The chemical structure of widespread microbial aryl polyene lipids. *bioRxiv doi 10.1101/2020.12.19.423268* (2020).
7. Cimermancic, P. *et al.* Insights into secondary metabolism from a global analysis of prokaryotic biosynthetic gene clusters. *Cell* **158**, 412-421 (2014).
8. Fiedor, J. & Burda, K. Potential role of carotenoids as antioxidants in human health and disease. *Nutrients* **6**, 466-488 (2014).
9. Hoffmann, T. *et al.* Correlating chemical diversity with taxonomic distance for discovery of natural products in myxobacteria. *Nat. Commun.* **9**, 803 (2018).
10. Sajnaga, E. & Kazimierczak, W. Evolution and taxonomy of nematode-associated entomopathogenic bacteria of the genera *Xenorhabdus* and *Photorhabdus*: an overview. *Symbiosis* **80**, 1-13 (2020).
11. Thanwisai, A. *et al.* Diversity of *Xenorhabdus* and *Photorhabdus* spp. and their symbiotic entomopathogenic nematodes from Thailand. *PLoS One* **7**, e43835 (2012).
12. Blin, K. *et al.* antiSMASH 5.0: updates to the secondary metabolite genome mining pipeline. *Nucleic Acids Res.* **47**, W81-W87 (2019).
13. Crawford, J. M., Portmann, C., Zhang, X., Roeffaers, M. B. J. & Clardy, J. Small molecule perimeter defense in entomopathogenic bacteria. *Proc. Natl. Acad. Sci. U. S. A.* **109**, 10821-10826 (2012).
14. Li, J.-H. *et al.* Making and breaking leupeptin protease inhibitors in pathogenic Gammaproteobacteria. *Angew. Chem. Int. Ed.* **59**, 17872-17880 (2020).
15. Bräuer, A. *et al.* Structural snapshots of the minimal PKS system responsible for octaketide biosynthesis. *Nat. Chem.* **12**, 755-763 (2020).
16. Medema, M. H. The year 2020 in natural product bioinformatics: an overview of the latest tools and databases. *Nat. Prod. Rep.* **38**, 301-306 (2021).
17. Montalbán-López, M. *et al.* New developments in RiPP discovery, enzymology and engineering. *Nat. Prod. Rep.* **38**, 130-239 (2020).
18. Oh, J., Kim, N. Y., Chen, H., Palm, N. W. & Crawford, J. M. An Ugi-like biosynthetic pathway encodes bombesin receptor subtype-3 agonists. *J. Am. Chem. Soc.* **141**, 16271-16278 (2019).
19. Park, H. B. *et al.* Bacterial autoimmune drug metabolism transforms an immunomodulator into structurally and functionally divergent antibiotics. *Angew. Chem. Int. Ed.* **59**, 7871-7880 (2020).
20. van der Hooft, J. J. J. *et al.* Linking genomics and metabolomics to chart specialized metabolic diversity. *Chem. Soc. Rev.* **49**, 3297-3314 (2020).
21. Schorn, M. A. *et al.* A community resource for paired genomic and metabolomic data mining. *Nat. Chem. Biol.* (2021).
22. Souto, A. *et al.* Structure and biosynthetic assembly of piscibactin, a siderophore from *Photobacterium damselae* subsp. *piscicida*, predicted from genome analysis. *Eur. J. Org. Chem.* **2012**, 5693-5700 (2012).
23. Drechsel, H. *et al.* Structure elucidation of yersiniabactin, a siderophore from highly virulent *Yersinia* strains. *Liebigs Ann.*, 1727-1733 (1995).
24. Fischbach, M. A. & Walsh, C. T. Assembly-line enzymology for polyketide and nonribosomal peptide antibiotics: logic, machinery, and mechanisms. *Chem. Rev.* **106**, 3468-3496 (2006).

25. Vizcaino, M. I., Engel, P., Trautman, E. & Crawford, J. M. Comparative metabolomics and structural characterizations illuminate colibactin pathway-dependent small molecules. *J. Am. Chem. Soc.* **136**, 9244-9247 (2014).
26. Cai, X. *et al.* Entomopathogenic bacteria use multiple mechanisms for bioactive peptide library design. *Nat. Chem.* **9**, 379-386 (2017).
27. Fuchs, S. W., Grundmann, F., Kurz, M., Kaiser, M. & Bode, H. B. Fabclavines: bioactive peptide-polyketide-polyamino hybrids from *Xenorhabdus*. *ChemBioChem* **15**, 512-516 (2014).
28. Grammbitter, G. L. C. *et al.* An uncommon type II PKS catalyzes biosynthesis of aryl polyene pigments. *J. Am. Chem. Soc.* **141**, 16615-16623 (2019).
29. Joyce, S. A. *et al.* Bacterial biosynthesis of a multipotent stilbene. *Angew. Chem. Int. Ed.* **47**, 1942-1945 (2008).
30. Wang, G. *et al.* CRAGE enables rapid activation of biosynthetic gene clusters in undomesticated bacteria. *Nat. Microbiol.* **4**, 2498-2510 (2019).
31. Stein, M. L. *et al.* One-shot NMR analysis of microbial secretions identifies highly potent proteasome inhibitor. *Proc. Natl. Acad. Sci. U. S. A.* **109**, 18367-18371 (2012).
32. Groll, M., Larionov, O. V., Huber, R. & de Meijere, A. Inhibitor-binding mode of homobelactosin C to proteasomes: New insights into class I MHC ligand generation. *Proc. Natl. Acad. Sci. U. S. A.* **103**, 4576-4579 (2006).
33. Groll, M., Korotkov, V. S., Huber, E. M., de Meijere, A. & Ludwig, A. A minimal β-lactone fragment for selective β5c or β5i proteasome inhibitors. *Angew. Chem. Int. Ed.* **54**, 7810-7814 (2015).
34. Fujii, K., Shimoya, T., Ikai, Y., Oka, H. & Harada, K.-i. Further application of advanced Marfey's method for determination of absolute configuration of primary amino compound. *Tetrahedron Lett.* **39**, 2579-2582 (1998).
