# Supplementary material for: Global analysis of biosynthetic gene clusters reveals conserved and unique natural products in entomopathogenic nematode-symbiotic bacteria
Source: Nat Chem. 2022 Apr 25;14(6):701–12. doi: 10.1038/s41557-022-00923-2 (PMC9177418; doi:10.1038/s41557-022-00923-2)
Supplement: Supplementary file 2 — Reporting Summary [file 41557_2022_923_MOESM2_ESM.pdf]

nature research

Corresponding author(s): Helge B. Bode
Yi-Ming Shi

Last updated by author(s): Nov 15, 2021

Reporting Summary

Nature Research wishes to improve the reproducibility of the work that we publish. This form provides structure for consistency and transparency in reporting. For further information on Nature Research policies, see our Editorial Policies and the Editorial Policy Checklist.

Statistics

For all statistical analyses, confirm that the following items are present in the figure legend, table legend, main text, or Methods section.

n/a	Confirmed	
☐	☒	The exact sample size (*n*) for each experimental group/condition, given as a discrete number and unit of measurement
☐	☒	A statement on whether measurements were taken from distinct samples or whether the same sample was measured repeatedly
☒	☐	The statistical test(s) used AND whether they are one- or two-sided<br>*Only common tests should be described solely by name; describe more complex techniques in the Methods section.*
☒	☐	A description of all covariates tested
☒	☐	A description of any assumptions or corrections, such as tests of normality and adjustment for multiple comparisons
☐	☒	A full description of the statistical parameters including central tendency (e.g. means) or other basic estimates (e.g. regression coefficient) AND variation (e.g. standard deviation) or associated estimates of uncertainty (e.g. confidence intervals)
☒	☐	For null hypothesis testing, the test statistic (e.g. *F*, *t*, *r*) with confidence intervals, effect sizes, degrees of freedom and *P* value noted<br>*Give P values as exact values whenever suitable.*
☒	☐	For Bayesian analysis, information on the choice of priors and Markov chain Monte Carlo settings
☒	☐	For hierarchical and complex designs, identification of the appropriate level for tests and full reporting of outcomes
☒	☐	Estimates of effect sizes (e.g. Cohen's *d*, Pearson's *r*), indicating how they were calculated

Our web collection on statistics for biologists contains articles on many of the points above.

Software and code

Policy information about availability of computer code

Data collection	Bruker Compass DataAnalysis 4.3 was used for chromatography and mass spectrometry. Bruker TopSpin 4.0 was used for NMR data. Geneious Prime 2021 was used for sequence data. Tecan iControl 2.0 was used for absorbance (cell viability) measurements. Collection of X-ray diffraction data was processed with the program package XDS version February 5, 2021.
Data analysis	Raw genome sequencing data were trimmed using Trimmomatic 0.39. Genomes were assembled with SPAdes 3.10.1 and annotated with Prokka 1.12. Completed genome sequences were analyzed and viewed in Geneious Prime 2021. Biosynthetic gene clusters were annotated by antiSMASH 5.0, exploration by BiG-FAM 1.0.0, and classification by BiG-SCAPE 1.0.0 with the MIBiG repository 2.0 reference and PFAM database 32.0. Cytoscape 3.7.2 was used to visualize the BiG-SCAPE network. Pangenome analysis and visualization were performed by anvi'o 6.1. Statistical data of biosynthetic gene clusters were analyzed and evaluated using Origin 2020b and Excel of Microsoft office 365. Compound production in a wild-type strain or mutant, as well as MS/MS were analyzed by Bruker Compass DataAnalysis 4.3 and MetabolicDetect 2.1. IC50 values (cytotoxicity and IOC proteasome inhibition) were calculated based on sigmoidal fitting in GraphPad Prism 8.4.3 or 9.0.2. Data from insect immunity bioassays were subjected to one-way ANOVA using PROC GLM of SAS program (SAS Institute, 1989) for continuous variables. All results were plotted by using Sigma plot 12.0. Conventional crystallographic rigid body, positional, and temperature factor refinements were carried out with REFMAC5 5.0.32. Protein crystal was determined and analyzed by CCP4 Program Suite 7.1.016. Model building was performed with Coot 0.8.7.

For manuscripts utilizing custom algorithms or software that are central to the research but not yet described in published literature, software must be made available to editors and reviewers. We strongly encourage code deposition in a community repository (e.g. GitHub). See the Nature Research guidelines for submitting code & software for further information.

Data

Policy information about availability of data

All manuscripts must include a data availability statement. This statement should provide the following information, where applicable:

- Accession codes, unique identifiers, or web links for publicly available datasets
- A list of figures that have associated raw data
- A description of any restrictions on data availability

The genome sequence data that support the findings of this study are available in NCBI GenBank database under accession nos. AYSJ00000000, CP011104.1, CP016176.1, FO704550, FOVO01000000, JADEUF000000000, JAGJDU000000000, JAGJJP000000000, JAGJJQ000000000, JAGJJR000000000, JAGJJS000000000, JAGJJT000000000, JAGJJU000000000, JAGJJV000000000, JAGJJW000000000, LOIC00000000, LOMY00000000, MKGQ00000000, MKGR00000000, MUBJ00000000, MUBK00000000, NC_005126.1, NC_013892.1, NC_014228.1, NIBS00000000, NIBT00000000, NIBU00000000, NIBV00000000, NITY00000000, NITZ00000000, NIUA00000000, NJAH00000000, NJAI00000000, NJAJ00000000, NJAK00000000, NJCW00000000, NJCX00000000, NJGH00000000, NKHP00000000, NKHQ00000000, NSCM00000000, VNHN00000000, WSEY00000000, WSFA00000000, WSFB00000000. For the corresponding genomes, see Supplementary Table 1. Crystallographic data have been deposited in the Protein Data Bank (https://www.rcsb.org) under the PDB ID 7O2L. All other data generated or analyzed in this study are available within the article and its Supplementary Information files.

Field-specific reporting

Please select the one below that is the best fit for your research. If you are not sure, read the appropriate sections before making your selection.

☒ Life sciences ☐ Behavioural & social sciences ☐ Ecological, evolutionary & environmental sciences

For a reference copy of the document with all sections, see nature.com/documents/nr-reporting-summary-flat.pdf

Life sciences study design

All studies must disclose on these points even when the disclosure is negative.

Sample size	For bacterial genomes, no sample-size calculation was performed. The involved strains (covering all strains in our collection and representing almost all Xenorhabdus and Photorhabdus taxonomy) for pangenome and sequence similarity network analysis were chosen in order to maximize our ability to obtain a comprehensive biosynthetic gene cluster atlas. Sample sizes of compound production in strains and bioassays were based on previous work (https://www.nature.com/articles/s41589-019-0246-1, https://www.nature.com/articles/ncomms10900, https://www.nature.com/articles/s41598-019-56892-z, https://sfamjournals.onlinelibrary.wiley.com/doi/10.1111/1462-2920.13845)
Data exclusions	A putative thiopeptide BGC (clusters: Xszus_1.region006, Xsze_2.region003, Xsto_4.region001, Xpb_30.3_21.region001, Xmir_10.region001, Xmau_6.region001, Xkoz_3.region001, Xjap_NZ_FOVO01000011.region001, Xish_1.region003, Xhom_ANU1.region005, Xhom_2.region003, Xets_11.region001, XenKK7.region002, XenDL20_c00108_NODE_12.region001, Xekj_19.region001, Xehl_28.region001, Xe30TX1_c0031_NODE_38.region001, Xdo_HBLC131_1.region001, Xdo_FRM16.1.region005, Xbov_NC_013892.1.region004, Ptem_HBLC135_17.region001, Ppb6_4.region001, Plum_TT01_1.region008, Pthr_PT1.1_23.region001, Plau_IT4.1_12.region001, Plum_IL9_35_scf0001.region001, Pbod_HU2.3_20.region001, Plau_HB1.3_105.region001, Plum_EN01_24_scf0009.region001, Pbod_DE6.1_24.region001, Plau_DE2.2_108.region001, Phpb_1.region001, Pbod_LJ_007.region001, Pbod_CN4_25_scf0020.region001, Paeg_BKT4.5_19.region001, P_tem_1.region017 et al.) that exists throughout 45 Xenorhabdus and Photorhabdus genomes was excluded in the analysis, since it turned out that its annotation by antiSMASH 5.0 is a false positive and early reports suggest that this cluster is responsible for ribosomal methylthiolation (doi: 10.1074/mcp.M110.005199 and doi: 10.1021/bi900939w). Two BGCs, Xdo_HBLC131_4.region001 encoding the biosynthesis of glidobactins in X. doucetiae HBLC131 and Ptem_HBLC135_2.region002 encoding the biosynthesis of ririwpeptides in P. temperata HBLC135 were artificially integrated into their respective genome by CRAGE previously (doi: 10.1038/s41564-019-0573-8), and thus the two BGCs were also excluded in our analysis. The exclusion criteria were pre-established at the outset of the study.
Replication	In general, all experiments were performed at least three independent times with representative data shown. All attempts to repeat compound determination and bioassays were successful. By comparing Xenorhabdus and Photorhabdus genome sequencing data available in NCBI, we were able to assess the reproducibility of the results (that is, the biosynthetic gene clusters) from each study (https://www.nature.com/articles/s41564-017-0039-9). Despite the different genome assemblies, the same biosynthetic gene clusters were recovered independently when using a consistent strain sample. Therefore, genome sequencing was not replicated.
Randomization	For all insect immunity assays, Spodoptera exigua fifth instar larvae (L5) aged day 1 (L5D1) were randomly allocated into experimental groups. For analysis of hemocyte-spreading behavior, a region with 100 cells was randomly chosen under the microscope for counting spread cells. For Galleria infection assays, the larvae were purchased and bred in-house to similar weight/size. Those deemed equivalents were randomly assigned to the experimental groups.
Blinding	Blinding was not relevant to this study, as we analyzed all sequenced genomes in our strain collection.

Reporting for specific materials, systems and methods

We require information from authors about some types of materials, experimental systems and methods used in many studies. Here, indicate whether each material, system or method listed is relevant to your study. If you are not sure if a list item applies to your research, read the appropriate section before selecting a response.

Materials & experimental systems

n/a	Involved in the study	
☒	☐	Antibodies
☐	☒	Eukaryotic cell lines
☒	☐	Palaeontology and archaeology
☐	☒	Animals and other organisms
☒	☐	Human research participants
☒	☐	Clinical data
☒	☐	Dual use research of concern

Methods

n/a	Involved in the study	
☒	☐	ChIP-seq
☒	☐	Flow cytometry
☒	☐	MRI-based neuroimaging

Eukaryotic cell lines

Policy information about cell lines

Cell line source(s)	DSMZ (ACC 180)
Authentication	HepG2 cells were obtained from DSMZ and did not undergo additional authentication procedures.
Mycoplasma contamination	negative
Commonly misidentified lines (See ICLAC register)	No commonly misidentified cell lines were used in the study.

Animals and other organisms

Policy information about studies involving animals; ARRIVE guidelines recommended for reporting animal research

Laboratory animals	The beet armyworm (Spodoptera exigua) was used in insect immunity assays. The wax moth (Galleria mellonella) was used for Xenorhabdus szentirmaii and the mutants thereof infection assays. Since we used only the larva of insects, the sex could not be determined until they become adults. The lepidopteran insect larvae of S. exigua were reared on an artificial diet (https://agris.fao.org/agris-search/search.do?recordID=KR19910051407) at 25 ± 2oC and relative humidity of 60 ± 5% with 16 h:8 h (L:D) photoperiod. Under these rearing conditions, S. exigua underwent five larval instars (L1-L5) before pupation. They continue being in the L5 phase for three days and then they become prepupa. In all insect immunity experiments, we used L5 day 1 (L5D1). Adults were provided with 10% sucrose for oviposition. G. mellonella larvae were purchased from Zoohaus Haindl, Frankfurt am Main.
Wild animals	The study did not involve wild animals.
Field-collected samples	S. exigua larvae were collected from Welsh onion (Allium fistulsum L.) field in Andong, Korea in 1994. The colony was reared for more than 26 years in the laboratory under the conditions described above. Insects were reared in the laboratory under conditions of 25 ± 2°C constant temperature, 16:8 h (L: D) photoperiod, and 60 ± 5 % relative humidity.
Ethics oversight	No ethical approval or guidance was required because no insect pests were used in this study.

Note that full information on the approval of the study protocol must also be provided in the manuscript.
